# Disease burden, risk factors, and trends of lip, oral cavity, pharyngeal cancers: A global analysis

**DOI:** 10.1002/cam4.6391

**Published:** 2023-07-30

**Authors:** Junjie Huang, Sze Chai Chan, Samantha Ko, Veeleah Lok, Lin Zhang, Xu Lin, Don E. Lucero‐Prisno, Wanghong Xu, Zhi‐Jie Zheng, Edmar Elcarte, Mellissa Withers, Martin C. S. Wong, NCD Global Health Research Group, Association of Pacific Rim Universities (APRU)

**Affiliations:** ^1^ Faculty of Medicine The Jockey Club School of Public Health and Primary Care, Chinese University of Hong Kong Hong Kong Special Administrative Region China; ^2^ Centre for Health Education and Health Promotion, Faculty of Medicine The Chinese University of Hong Kong Hong Kong Special Administrative Region China; ^3^ Department of Global Public Health Karolinska Institute, Karolinska University Hospital Stockholm Sweden; ^4^ Suzhou Industrial Park Monash Research Institute of Science and Technology Suzhou China; ^5^ The School of Public Health and Preventive Medicine Monash University Victoria Australia; ^6^ Department of Thoracic Surgery The First Affiliated Hospital, School of Medicine, Zhejiang University Hangzhou Zhejiang China; ^7^ Department of Global Health and Development London School of Hygiene and Tropical Medicine London UK; ^8^ School of Public Health, Fudan University Shanghai China; ^9^ Department of Global Health School of Public Health, Peking University Beijing China; ^10^ University of the Philippines Manila the Philippines; ^11^ Department of Population and Health Sciences Institute for Global Health, University of Southern California Los Angeles USA

**Keywords:** burden, lip, oral cavity, pharyngeal cancer, risk factors, temporal trends

## Abstract

**Background:**

Lip, oral and pharyngeal cancers make up a small percentage of total cancer cases worldwide and have reported lower rates of cancer‐related deaths globally in 2020, but their 5‐year survival rate in either early or advanced stages is different. The study evaluated the global incidence, mortality, risk factors, and temporal trends by age, gender, and geographical locations of lip, oral cavity, and pharyngeal cancer.

**Methods:**

Incidence and mortality rates were extracted from *Cancer Incidence in Five Continents* (*CI5*) volumes I‐XI, the *Nordic Cancer Registries* (*NORDCAN*), the *Surveillance, Epidemiology*, and *End Results* (*SEER*) *Program*, and the WHO IARC mortality database. Joinpoint regression was used to calculate the Average Annual Percentage Change to examine trends.

**Results:**

The highest incidence rates were found in Melanesia and South‐Central Asia and mortality rates were 8.2 and 7.5. Risk factors associated with incidence and mortality included HDI, tobacco use, alcohol consumption, poor diet, and chronic health conditions such as hypertension. Increasing trends of incidence and mortality were observed in females from Malta; males aged 50 and above from the United Kingdom, and females aged 50 and above from Slovakia reporting the largest increase.

**Conclusions:**

Although global incidence and mortality trends reported an overall decrease, significant increases were found for older age groups and female subjects. Incidence increase may be due to the growing prevalence of lifestyle, metabolic risk factors, and HPV infections, especially in developed countries.

## 1 INTRODUCTION

Lip, oral cavity, and pharyngeal cancers have been estimated to be responsible for about 4.1% of all cancer cases and 3.7% of cancer‐related deaths globally in 2020.^1^ The overall 5‐year survival rate worldwide is approximately 50%; there is an 80% 5‐year survival rate for stage I cancer which plummets to 20% for advanced stages (stages III/IV).^2^, ^3^, ^4^ In the early stages, oral cancers are often asymptomatic but may eventually present as pain caused by lesions. Lesions may appear as white and red patches, referred to as leukoplakia and erythroplakia, respectively. Additional symptoms at later stages may include bleeding, loose teeth, difficulty wearing dentures, dysphagia, dysarthria, odynophagia, and the development of a neck mass.

In developed and developing countries, cancers of the lip, oral cavity, and pharynx variety have been shown to be associated with exposure to risk factors such as tobacco use, including chewing or smoking tobacco^5^, ^6^; consumption of alcohol^7^; consumption of nitrosamine‐rich foods^8^; infection with human papillomavirus (HPV)^9^; environmental exposures, for example, ultraviolet radiation (UVR)^10^; dietary deficiencies due to a lack of fruit and non‐starchy vegetables.^11^

Though previous studies have examined the epidemiological trends of lip, oral cavity, and pharyngeal cancers, they utilized data limited to specific regions and countries,^12^, ^13^ of lower quality,^14^ or did not include analyses on age subgroups.^15^ An updated global assessment to examine the country‐specific, sex‐specific, and age‐specific incidence and mortality rates using high‐quality data would contribute toward a better understanding of the international differences in etiology, diagnosis, prognosis, and treatment. Additionally, this would inform the development of targeted interventions and disease prevention policies to reduce the overall burden of lip, oral cavity, and pharyngeal cancers. This study investigated the global incidence, mortality, risk factors, and temporal trends of lip, oral cavity, and pharyngeal cancers by age, sex, and geographical location.

## 2 MATERIALS AND METHODS

### 2.1 Data sources

To define the lip, oral cavity, and pharyngeal cancers, we used the International Classification of Diseases Version 10 (ICD‐10) C00‐14).^16^ Data were extracted from the Global Cancer Observatory (GLOBOCAN) database, developed by the International Agency for Research on Cancer, World Health Organization (IARC, WHO).^17^ The Gross Domestic Product (GDP) per capita and Human Development Index (HDI) for each country and region were retrieved from World Bank and United Nations. For the categorization of HDI rates, <0.550, 0.550–0.699, 0.700–0.700, and ≥0.800 are considered low, medium, high, and very high. The WHO Global Health Observatory data repository was employed for the age‐adjusted prevalence of smoking, alcohol drinking, unhealthy dietary, physical inactivity, hypertension, diabetes, and lipid disorders, at the country level.^18^

For the trend analysis on lip, oral cavity, and pharyngeal cancer incidence, data from the *Cancer Incidence in Five Continents* (*CI5*) volumes I‐XI, the *Nordic Cancer Registries* (*NORDCAN*), and the *Surveillance*, *Epidemiology*, and *End Results* (*SEER*) *Program* were accessed.^19^, ^20^, ^21^ The *CI5* database contains cancer incidence‐related data drawn from global, regional, and national cancer registries including the percentage of cases officially registered, the frequency of cases microscopically recorded, and cancer incidence by age, primary tumor year, and geographical locations. The *NORDCAN* database and *SEER* program contain cancer‐related statistics from the Nordic region and the United States, respectively. The WHO IARC mortality database contained cancer‐related death data for each country and region and was used for conducting a mortality trend analysis.^22^ Collecting data on cancer‐related deaths from national civil cancer registries on a local and national level with the registering system verified cancer deaths and their causes, which were then reported to the WHO annually. The weighted age‐standardized rate (ASR) was generated using the Segi‐Doll world reference population to transform all cancer incidence and mortality figures.^23^, ^24^ Weighting was proportionate to the individuals in the standard population's corresponding age groups.

### 2.2 Statistical analysis

Choropleth maps were constructed on the global incidence and mortality of lip, oral cavity, and pharyngeal cancers in 2020. To assess the relationships between GDP, HDI, risk factors, and lip and oral cavity incidence and mortality, a linear regression analysis was conducted using STATA 16.0 to generate the both beta coefficients (*β*) and the corresponding 95% CIs. The β estimates referred to the level of variation in ASR of incidence or mortality of lip, oral cavity, and pharyngeal cancers. To assess the temporal trend of incidence and mortality rates of all ages across gender (male and female) and geographical regions (Asia, Oceania, Northern America, Southern America, Northern Europe, Western Europe, Southern Europe, Eastern Europe), a logarithm transformation was performed on the incidence and mortality rates before carrying out the joinpoint regression analysis. The Average Annual Percentage Change (AAPC) and its 95% CI were calculated using joinpoint regression analysis software (Version 4.8.0.1–April 2020; Statistical Methodology and Applications Branch, Surveillance Research Program, National Cancer Institute). When conducting analysis on trends with transitions, AAPC is preferred over annual percentage change (APC) since the length of the time segment is considered and it does not assume linearity.^25^ This method has been used in previous studies to determine the epidemiological trend of other forms of cancer, with the outcome of positive or negative AAPC indicating an increasing or decreasing trend in cancer incidence or mortality, respectively.^26^ All *p* < 0.05 were considered statistically significant.

## 3 RESULTS

### 3.1 Global incidence of lip, oral cavity, and pharyngeal cancer in 2020

**FIGURE 1:**
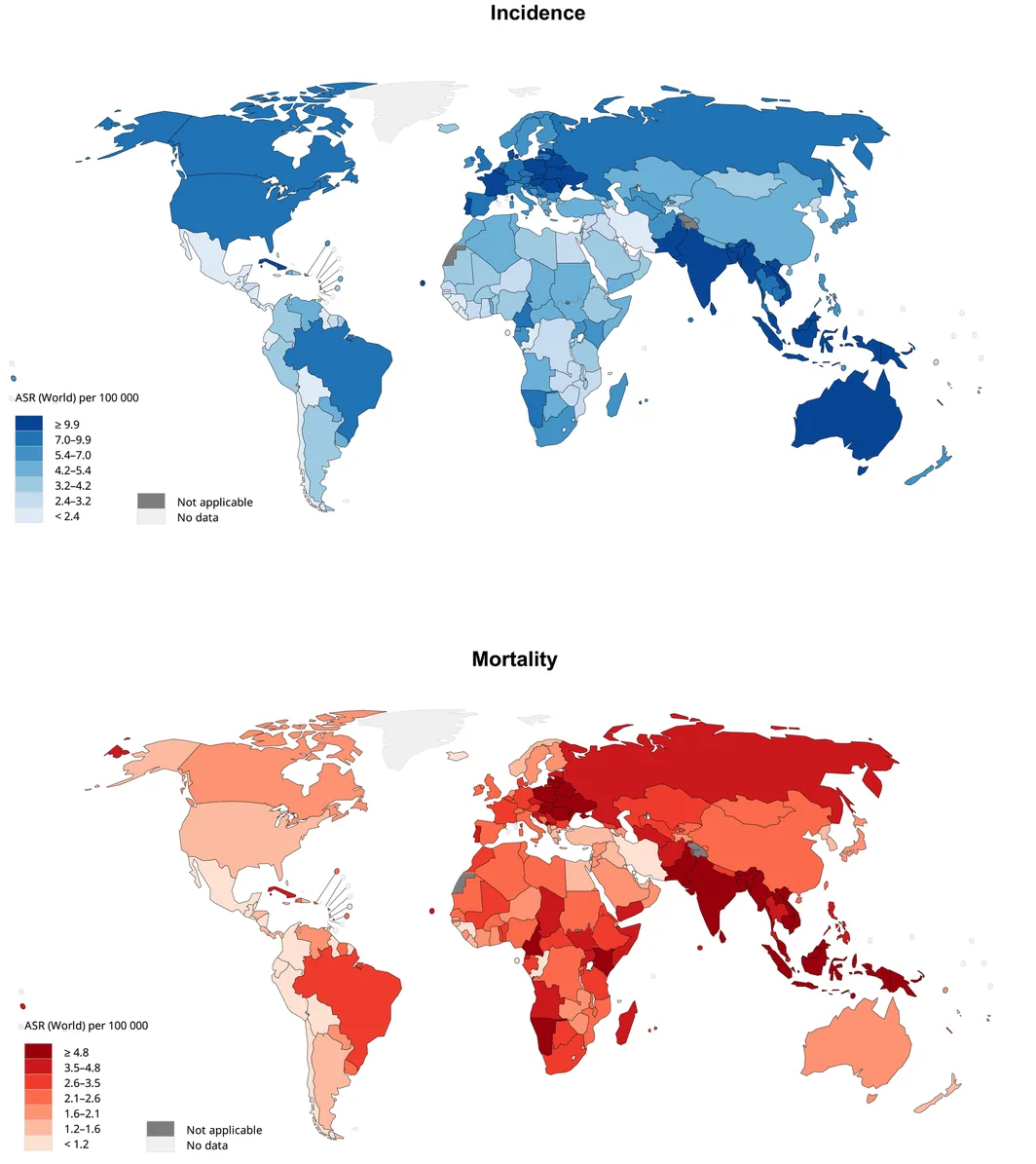
Global incidence and mortality of LOCP cancers, both sexes, all ages, in 2020. Data source: GLOBOCAN 2020 Graph production: IARC (http://gco.iarc.fr/today) World Health Organization.

In 2020, there were an estimated 747,316 newly reported cases of lip, oral cavity, and pharyngeal cancer with a global ASR of 8.1 new cases per 100,000 population (Figure 1). The region with the highest incidence was Melanesia (ASR = 19.8), followed by South‐Central Asia (13.5), Central and Eastern Europe (10.3), Western Europe (10.0), and Australia and New Zealand (10.0). On the contrary, the lowest incidence was found in Central America (2.0), Western Africa (3.1), Western Asia (3.7), Northern Africa (ASR = 3.9), and Eastern Africa (4.3). The incidence rate of males (12.2) was almost 3 times that of females (4.3). Countries with medium HDI had the highest incidence (19.5), followed by those with very high HDI (13.1), high HDI (8.3), and low HDI (5.2). The incidence of lip, oral cavity, and pharyngeal cancer increased with age, with the highest incidence found among the 70–85+ age group (37.8, Table S1).

As far as the subsite is concerned, lip and oral cavity cancer had the highest incidence (4.1), followed by cancers of the nasopharynx (1.5), oropharynx (1.1), hypopharynx (0.9) and salivary glands (0.6). The 70–85+ age group had the highest incidence of the cancers of lip and oral cavity (20.6), hypopharynx (5.0), and salivary glands (3.4); whereas the highest incidence in oropharyngeal (5.3) and nasopharyngeal cancer (4.8) was found among the 60–69 age group.

### 3.2 Global mortality of lip, oral cavity, and pharyngeal cancer in 2020

In 2020, it was estimated that 367,285 lip, oral cavity, and pharyngeal cancer‐related deaths were reported on a global scale (ASR = 3.9 death cases per 100,000 population). Similarly, Melanesia had the highest mortality (8.2), followed by South‐Central Asia (7.5), South‐Eastern Asia (5.8), Central and Eastern Europe (5.2), and Micronesia (4.1); while regions with the lowest mortality were Central America (0.90), Northern America (1.6), Western Asia (1.7), Australia and New Zealand (1.8), and Northern Africa (2.1). There was a threefold difference in the mortality between the two sexes (male ASR = 6.0, female ASR = 2.0). Populations with medium HDI had a significantly higher mortality of 7.3, while the other populations had mortality ranging from ASR 2.8 to 2.9. Similarly, the highest mortality was found among the 70–85+ age group (21.1).

Lip and oral cavity cancer had the highest mortality (1.9), followed by nasopharyngeal (0.88), oropharyngeal (0.51), hypopharyngeal (0.23), and salivary gland cancer (0.23). The age group of 70–85+ had the highest mortality for all cancers (ASR: 1.9–10.0) except nasopharyngeal cancer, for which the highest mortality was found among the 60–69 population.

### 3.3 Associations between risk factors and lip, oral cavity, and pharyngeal cancer incidence

**FIGURE 2:**
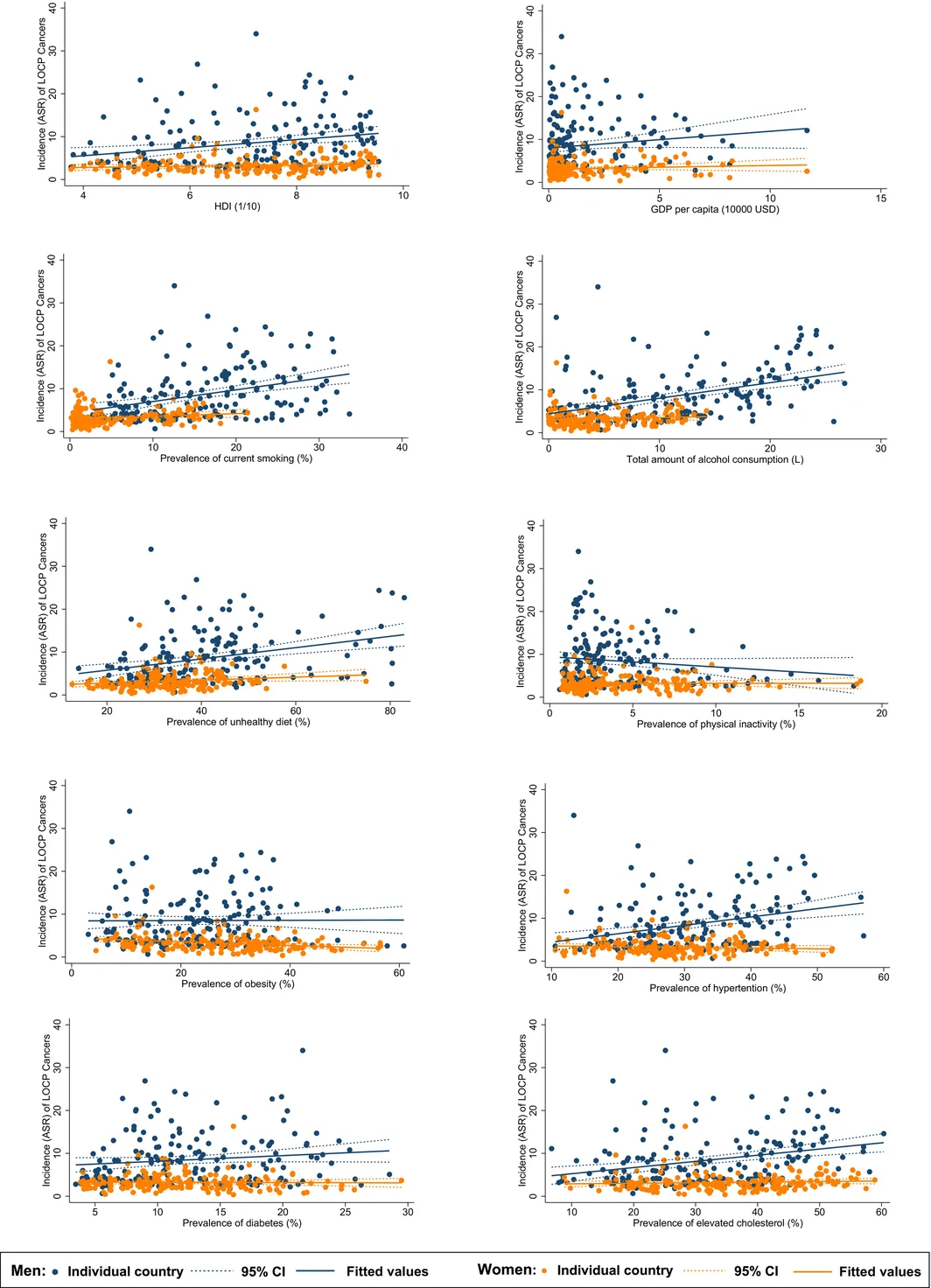
Associations between risk factors and LOCP cancers incidence.

Among males (Figure 2), incidence of lip, oral cavity, and pharyngeal cancer was associated with a higher HDI (*β* = 0.941, CI: 0.374–1.507, *p* = 0.001). Furthermore, it was associated with higher prevalence of risk factors including smoking (*β* = 0.272, CI: 0.164–0.379, *p* < 0.001), alcohol consumption (*β* = 0.363, CI: 0.253–0.473, *p* < 0.001), poor diet (*β* = 0.132, CI: 0.072–0.191, *p* < 0.001), hypertension (*β* = 0.195, CI: 0.102–0.289, *p* < 0.001), and lipid disorders (*β* = 0.144, CI: 0.074–0.214, *p* < 0.001), after adjusting for HDI and GDP per capita. Among females, incidence was associated with a higher prevalence of smoking (*β* = 0.063, CI: 0.006–0.119, *p* = 0.029) and poor diet (*β* = 0.035, CI: 0.003–0.066, *p* = 0.029), but a lower prevalence of obesity (*β* = −0.042, CI: −0.067 to −0.017, *p* < 0.001), after adjusting for HDI and GDP per capita.

### 3.4 Associations between risk factors and lip, oral cavity, and pharyngeal cancer mortality

**FIGURE 3:**
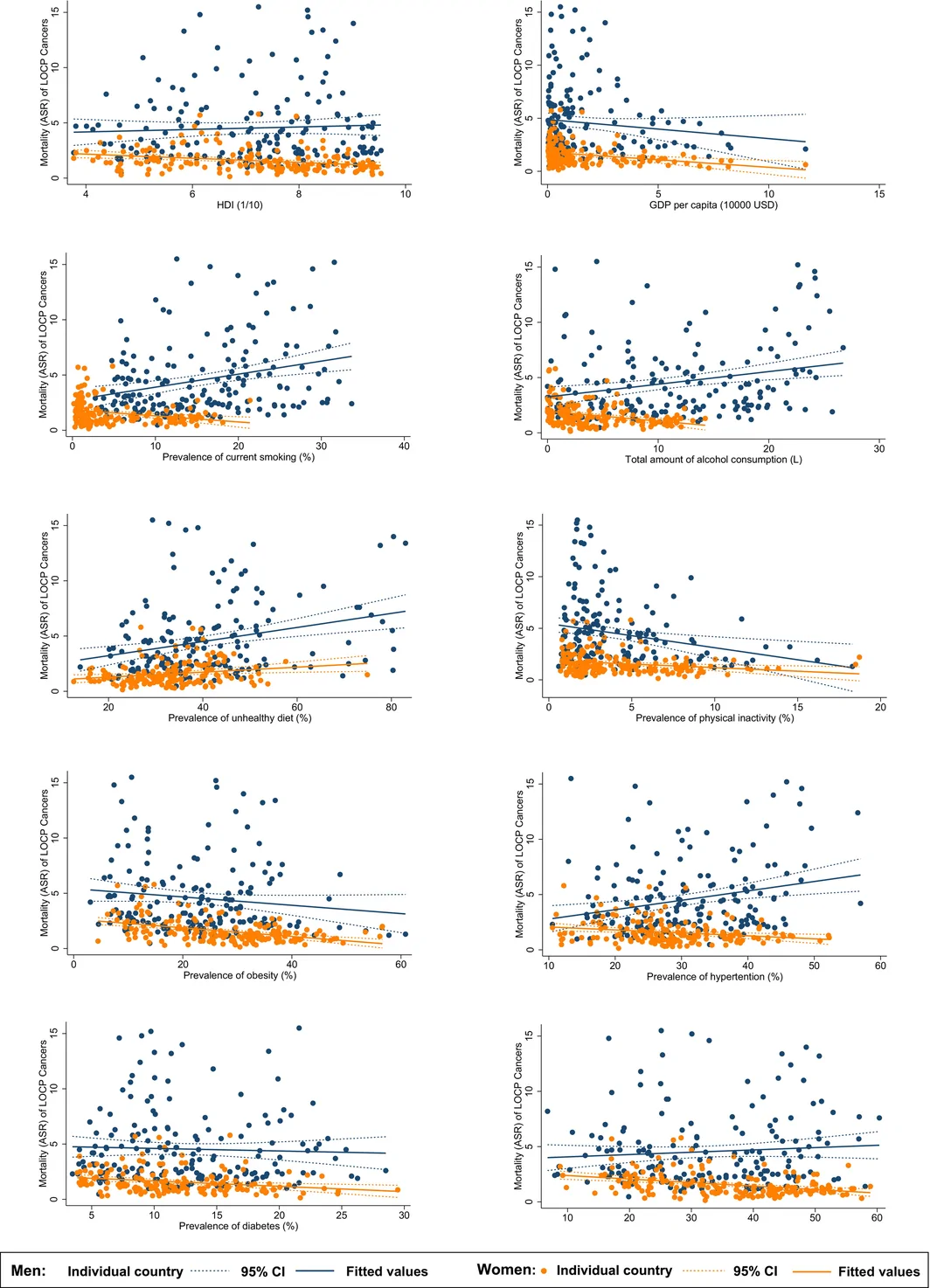
Associations between risk factors and LOCP cancers mortality.

Among males (Figure 3), mortality of lip, oral cavity, and pharyngeal cancer was associated with a higher prevalence of smoking (*β* = 0.118, CI: 0.056–0.179, *p* < 0.001), alcohol consumption (*β* = 0.115, CI: 0.049–0.181, *p* = 0.001), poor diet (*β* = 0.064, CI: 0.030–0.097, *p* < 0.001), and hypertension (*β* = 0.085, CI: 0.032–0.138, *p* = 0.002), but lower prevalence of physical inactivity (*β* = −0.234, CI: −0.388 to −0.079, *p* = 0.003). Among females, mortality was associated with a higher prevalence of poor diet (*β* = 0.023, CI: 0.006–0.039, *p* = 0.008), but lower HDI (*β* = −0.187, CI: −0.286 to −0.089, *p* < 0·001), GDP per capita (*β* = −0.143, CI: −0.218 to −0.067, *p* < 0.001), lower prevalence of smoking (*β* = −0.055, CI: −0.084 to −0.025, *p* < 0.001), alcohol consumption (*β* = −0.089, CI: −0.129 to −0.049, *p* < 0.001), physical inactivity (*β* = −0.069, CI: −0.115 to −0.023, *p* = 0.003), obesity (*β* = −0.039, CI: −0.052 to −0.027, *p* < 0.001), hypertension (*β* = −0.027, CI: −0.044 to −0.009, *p* = 0.003), diabetes (*β* = −0.048, CI; −0.078 to −0.018, *p* = 0.002), and lipid disorders (*β* = −0.032, CI: −0.044 to −0.019, *p* < 0.001).

### 3.5 Temporal trends of lip, oral cavity, and pharyngeal cancer

The incidence and mortality trends of lip, oral cavity, and pharyngeal cancer for each country between 1980 and 2019 are shown in alphabetical order according to their continents in Figure S1, and the trend regression is presented in Figure S2.

### 3.6 Incidence trends of all ages

**FIGURE 4:**
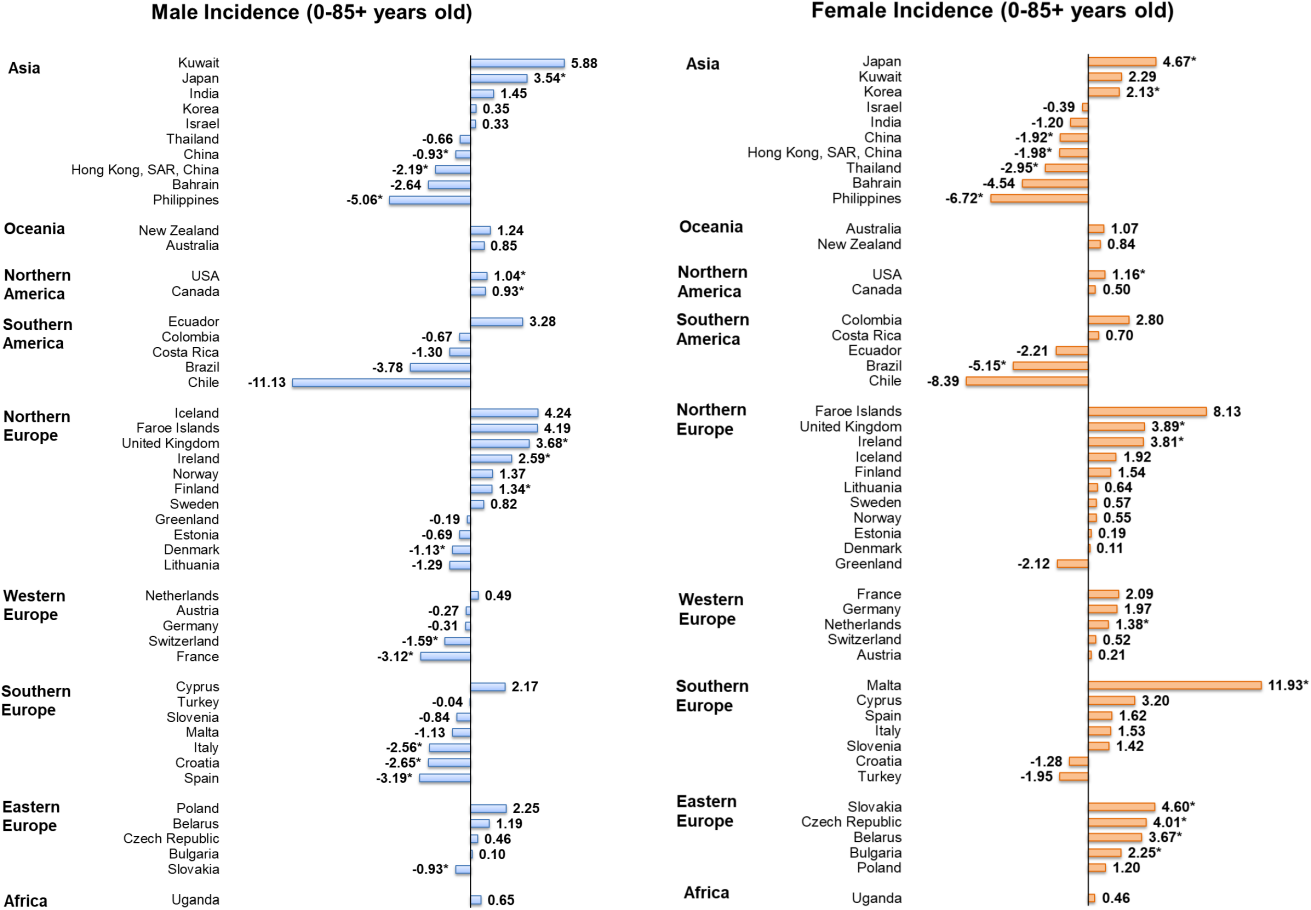
AAPC of LOCP cancers incidence for individuals of all ages. AAPC, annual percentage change; *p < 0.05.

For males, six countries showed evident increases, and the most remarkable increases were reported in United Kingdom (AAPC [in %] = 3.68; 95% CI = 3.12–4.24; *p* < 0.0001), Japan (AAPC = 3.54; 95% CI = 1.99–5.11; *p* = 0.0007), and Ireland (AAPC = 2.59; 95% CI = 1.27–3.92; *p* = 0.0018) (Figure 4). Remarkable decreases were reported in 10 countries, countries showing the highest decreases were the Philippines (AAPC = −5.06; 95% CI = −6.56 to −3.54; *p* = 0.0001), Spain (AAPC = −3.19; 95% CI = −4.58 to −1.77; *p* < 0.0001), and France (AAPC = −3.12; 95% CI = −4.13 to −2.09; *p* = 0.0001). For females, 10 countries showed significant increasing trends, with Malta (AAPC = 11.93; 95% CI = 0.85–24.22; *p* = 0.037), Japan (AAPC = 4.67; 95% CI = 3.11–6.26; *p* = 0.0001), and Slovakia (AAPC = 4.60; 95% CI = 2.25–7.01; *p* = 0.0019) reporting the greatest increases. Significant decreasing trends were found in 5 countries, as Philippines (AAPC = −6.72; 95% CI = −10.66 to −2.61; *p* = 0.0016), Brazil (AAPC = −5.15; 95% CI = −9.11 to −1.01; *p* = 0.021), and Thailand (AAPC = −2.95; 95% CI = −3.94 to −1.96; *p* = 0.0001) reported the largest decreases.

### 3.7 Mortality trend of all ages

**FIGURE 5:**
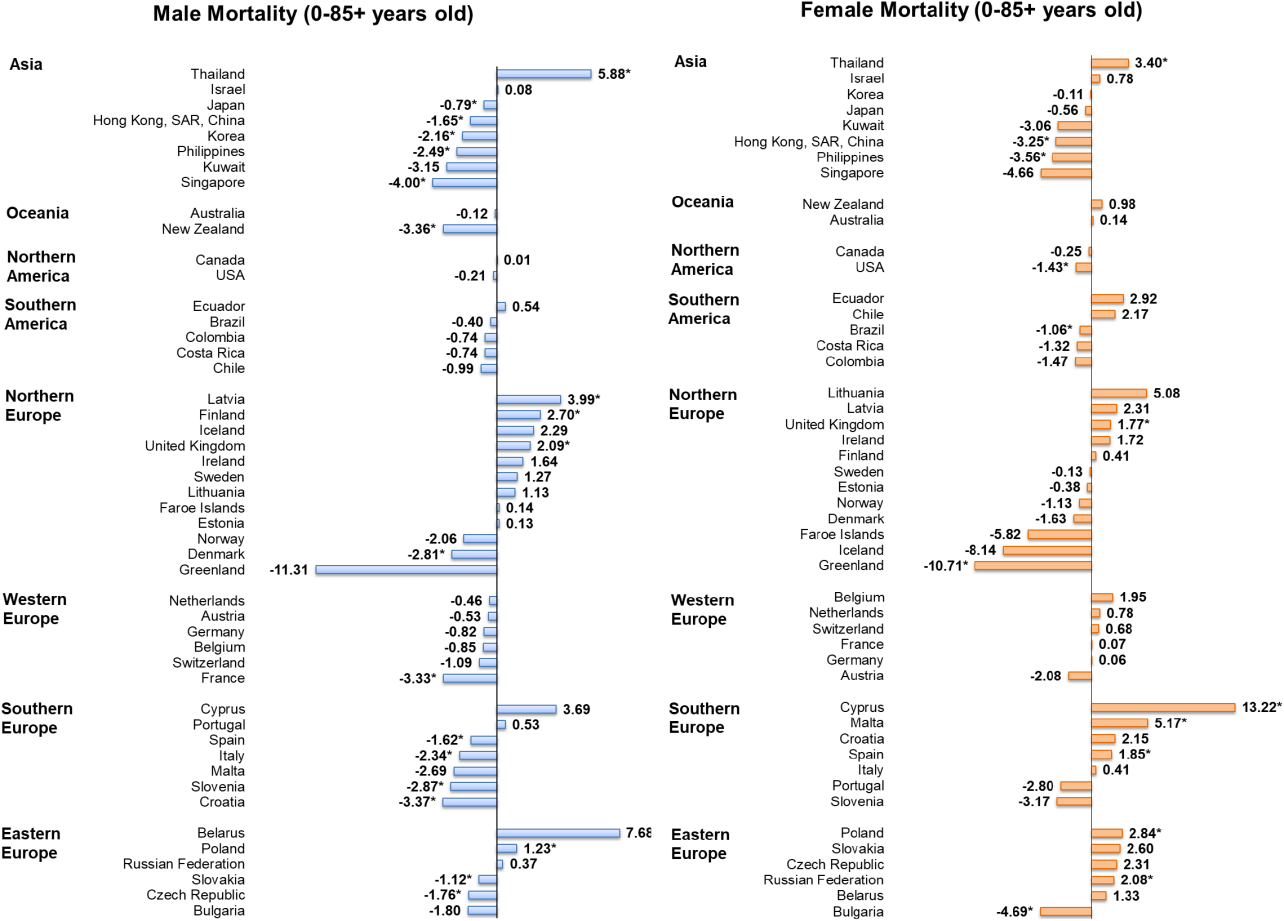
AAPC of LOCP cancers mortality for individuals of all ages. AAPC, annual percentage change; *p < 0.05.

For males, 4 countries reported significant increases, as Thailand (AAPC = 5.88; 95% CI = 4.65–7.13; *p* < 0.0001), Latvia (AAPC = 3.99; 95% CI = 1.12–6.96; *p* = 0.012), and United Kingdom (AAPC = 2.09; 95% CI = 1.28–2.9; *p* = 0.0003) reported the largest increases (Figure 5). In contrast, significant decreases were observed in 15 countries. The largest decreases were found in Singapore (AAPC = −4.00; 95% CI = −6.78 to −1.14; *p* = 0.0064), Croatia (AAPC = −3.37; 95% CI = −5.40 to −1.29; *p* = 0.0059), and New Zealand (AAPC = −3.36; 95% CI = −4.45 to −2.25; *p* = 0.0001). For females, significant increasing trends were reported in 7 countries, such as Cyprus (AAPC = 13.22; 95% CI = 1.67–26.08; *p* = 0.029), Malta (AAPC = 5.17; 95% CI = 0.22–10.37; *p* = 0.043), and Thailand (AAPC = 3.4; 95% CI = 2.52–4.28; *p* < 0.0001) reported the largest increases. Five countries reported significant decreasing trends, with Bulgaria (AAPC = −4.69; 95% CI = −8.80 to −0.40; *p* = 0.036), Philippines (AAPC = −3.56; 95% CI = −4.29 to −2.82; *p* < 0.0001), and Hong Kong, SAR, China (AAPC = −3.25; 95% CI = −6.22 to −0.19; *p* = 0.038) reporting the most remarkable decreases.

### 3.8 Incidence trend by age groups

For males aged 50 years or above, 11 countries showed evident increases, and the most remarkable increases were reported in United Kingdom (AAPC = 3.99; 95% CI = 3.29–4.69; *p* < 0.0001), Japan (AAPC = 3.50; 95% CI = 2.06–4.97; *p* = 0.0005), and Ireland (AAPC = 2.35; 95% CI = 0.70–4.02; *p* = 0.011) (Figure S3). Significant decreasing trends were observed in 6 countries, Philippines (AAPC = −5.22; 95% CI = −6.82 to −3.59; *p* = 0.0001), Spain (AAPC = −2.74; 95% CI = −3.74 to −1.73; *p* = 0.0003), and Croatia (AAPC = −2.17; 95% CI = −3.36 to −0.97; *p* = 0.0032) had reported the greatest decreases. For younger males aged below 50, evident increasing trends were found in Ecuador (AAPC = 23.56; 95% CI = 16.40–31.15; *p* < 0.0001), Thailand (AAPC = 3.55; 95% CI = 0.71–6.48; *p* = 0.020), and United Kingdom (AAPC = 2.54; 95% CI = 2.02–3.06; *p* < 0.0001); while significant decreases were observed in 15 countries, including Germany (AAPC = −8.3; 95% CI = −12.18 to −4.26; *p* = 0.0017), France (AAPC = −7.86; 95% CI = −10.35 to −5.29; *p* < 0.0001), and Costa Rica (AAPC = −6.67; 95% CI = −12.55 to −0.4; *p* = 0.040) (Figure S4). For the youngest age group below 40, Ecuador (AAPC = 21.83; 95% CI = 9.49–35.55; *p* = 0.0027), Japan (AAPC = 5.48; 95% CI = 2.04–9.03; *p* = 0.0059), and United Kingdom (AAPC = 2.35; 95% CI = 0.21–4.54; *p* = 0.035) reported evident increases, while remarkable decreases were found only in France (AAPC = −5.83; 95% CI = −10.89 to −0.48; *p* = 0.036) and Cyprus (AAPC = −5.61; 95% CI = −8.32 to −2.81; *p* = 0.0001) (Figure S5).

For females aged 50 or above, significant increases were reported in 12 countries, as Slovakia (AAPC = 5.21; 95% CI = 2.38–8.11; *p* = 0.0026), Czech Republic (AAPC = 5.02; 95% CI = 3.50–6.56; *p* = 0.0001), and Japan (AAPC = 4.31; 95% CI = 2.42–6.24; *p* = 0.0007) reported the most remarkable increases. Significant decreases were reported in Philippines (AAPC = −7.63; 95% CI = −12.21 to −2.81; *p* = 0.0022), Thailand (AAPC = −4.51; 95% CI = −6.43 to −2.54; *p* = 0.0008), and Chile (AAPC = −2.1; 95% CI = −3.24 to −0.94; *p* = 0.0032). For the age group below 50, For females, 4 countries reported significant increasing trends, with Malta (AAPC = 13.83; 95% CI = 7.69–20.32; *p* = 0.0007) reporting the most remarkable increase, followed by Japan (AAPC = 5.76; 95% CI = 1.68–10.01; *p* = 0.011), and United Kingdom (AAPC = 3.03; 95% CI = 1.36–4.73; *p* = 0.0029). In contrast, Hong Kong, SAR, China (AAPC = −2.53; 95% CI = −3.93 to −1.10; *p* = 0.0036), Chile (AAPC = −1.69; 95% CI = −2.82 to −0.55; *p* = 0.0094) reported significant decreases. For the youngest female age group under 40 years old, five countries reported evident increases, Estonia (AAPC = 11.98; 95% CI = 2–22.93; *p* = 0.023) had the greatest increase, followed by the Netherlands (AAPC = 6.07; 95% CI = 0.41–12.06; *p* = 0.038), and United Kingdom (AAPC = 4.29; 95% CI = 2.26–6.36; *p* = 0.0011), whereas significant decreases were reported in Brazil (AAPC = −19.07; 95% CI = −30.37 to −5.94; *p* = 0.012), Hong Kong, SAR, China (AAPC = −4.4; 95% CI = −7.98 to −0.68; *p* = 0.026), and China (AAPC = −3.93; 95% CI = −5.95 to −1.85; *p* = 0.0025).

## 4 DISCUSSION

### 4.1 Summary of major findings

This study comprehensively analyses the disease burden, risk factors, and temporal epidemiological trends of lip, oral cavity, and pharyngeal cancer, using the most updated data retrieved from databases. Subgroups of regions, countries, sexes, and age groups have been included in a robust statistical analysis. We noted a significant geographical disparity in the incidence of the cancer. Higher lip, oral cavity, and pharyngeal cancer incidence and mortality were associated with higher HDI as well as prevalence of smoking, alcohol consumption, poor diet, and hypertension in males, while mortality was associated with a lower HDI, GDP, and prevalence of lifestyle and metabolic risk factors in females. There has been an overall decreasing trend in incidence for both male and female among Asian countries, while a general increasing trend in incidence was found in European countries, such trends were particularly evident in female aged 50 years old or above. For mortality, a decreasing trend was found among the male population in general while an overall increasing trend was observed for female.

### 4.2 Explanations and comparisons with past literature

Lip, oral cavity, and pharyngeal incidence were found to be highest in Melanesia, South‐Central Asia, Australia, and New Zealand, which supports past findings where overall incidence rates ranged from 0.5 to 21.2 in males and 0.5 to 12.0 per 100,000 persons in females.^27^ The increasing number of cases in European countries, with significant rises in developed countries, may be due to advancements in early cancer detection and increased exposure to risk factors. For instance, central and Eastern Europe and Western Europe incidence was significantly higher than the global average. This further reinforces evidence where the region previously reported a majority of newly diagnosed cases (19.2% worldwide) in 2012.^15^ In the present study, the highest mortality rates were found in the region of Melanesia, South‐Central Asia, South‐Eastern Asia, Central and Eastern Europe, and Micronesia, all of which are locations where high lip, oral cavity, and pharyngeal incidence rates have been reported.^28^

Risk factor associations, such as high HDI, substance use, and chronic health conditions, reinforce findings from previous studies. The highest incidence and mortality of lip, oral cavity, and pharyngeal cancer were observed in medium HDI countries with the ASR being 19.5 and 7.3, respectively. Previously in 2012, countries with low HDI had a higher ASR of 4.4 for incidence compared to medium HDI countries (ASR = 2.2), and higher age‐standardized mortality rates in low HDI countries (ASR = 3.3) than in medium HDI countries. (ASR = 2).^29^ Incidence and mortality rates are usually pronounced in low and medium HDI settings as rapid socio‐economic development brings about the adoption of adverse lifestyle, behavioral, and environmental factors. Moreover, the existing healthcare infrastructure and medical interventions are ill‐equipped to elevate the increasing burden of cancer.^30^ A review of the prevalence of substance usage in different regions found that tobacco smoking and alcohol consumption were primary risk factors for oral cavity cancer in Europe, North, and Latin America.^31^ Additionally, Melanesia, South‐Central Asia, and South‐Eastern Asia may be partially explained by the prevalence of betel quid chewing^32^ while solar radiation in parts of Oceania, namely Australia and New Zealand, is the most pertinent risk factor associated with these regions.^15^ Non‐starchy vegetables and fruit intake may act as a protective factor as dietary deficiencies and increased red meat consumption have been associated with 11%–15% of oral cavity and pharyngeal cancer cases.^31^, ^33^, ^34^ Hypertension is one of the most frequently reported comorbidities found for lip, oral cavity, and pharyngeal cancer, as both diseases share the same risk factors including sedentary lifestyle, unhealthy diet, obesity, alcohol use, and smoking.^35^, ^36^

The global variation in lip, oral cavity, and pharyngeal cancer incidence and mortality trends may be reflective of the changing risk factor prevalence and population lifestyle of each region. The gradual decreasing trend in global incidence, particularly in males, mirrors the overall decline in tobacco use since 1990 (−27.2%, 95% CI: −26.0% to −28.3%).^37^ This is indicative of the effectiveness of interventions centered around substance use cessation and its importance in preventing the development of cancers.^38^ Although majority of cases are found in people aged 50 and older, rising incidence trends in younger age groups within developed regions—Australia, Europe, and North America—have been thought to be associated with the spread of human papillomavirus (HPV) infection.^2^, ^15^, ^39^ For instance, HPV‐associated oropharyngeal cancer has been increasing from 16% in 1984 to more than 70% in 2000 in the USA.^40^ An upward trend of cancer incidence and mortality in women was accompanied by a growing prevalence of smoking among females, with female smokers shown to be at an increased risk of developing oral cavity cancer compared to males.^2^, ^28^

### 4.3 Limitations

There were some limitations in this study. First, the estimates of GLOBOCAN 2020, which were based on past data on incidence and mortality trends, did not take into account the influence of the COVID‐19 pandemic. The incidence might have been overestimated as cancer diagnoses were expected to decline due to the pandemic. On the contrary, there might be an underestimation of the mortality because of late diagnosis and co‐infection of COVID‐19. Further, there is a possibility of the under‐reporting of incidence and mortality of lip, oral cavity, and pharyngeal cancers in developing countries due to the absence of well‐established infrastructure and mechanisms for cancer reporting. Lastly, an analysis of the trends of different stages, subtypes, and categories of lip, oral cavity, and pharyngeal cancers was not conducted due to the limited availability of data.

## 5 CONCLUSION

The incidence of lip, pharynx, and oral has been steadily increasing, especially in developed countries, female subjects, and older populations. While mortality decreased in males in the majority of the regions, overall rates have been increasing in female population. The rise in cases may be due to the improvement in disease detection and increased prevalence of its related lifestyle and metabolic risk factors. Lifestyle modifications are highly recommended to combat the risk posed by such factors by means of alcohol control, weight control, and increasing physical activity. As diagnosis in advanced stages worsens prognosis significantly, methods to enhance early detection and disease surveillance may improve overall treatment outcomes. Future research may look to explore the precipitating variables behind the epidemiologic trends which may provide more insight into the etiology and prognosis of lip, oral, and pharyngeal cancers.

## AUTHOR CONTRIBUTIONS

**Junjie Huang:** Conceptualization (lead); supervision (lead); writing – original draft (equal). **Sze Chai Chan:** Data curation (equal); formal analysis (equal); writing – original draft (equal). **Samantha Ko:** Writing – original draft (equal). **Veeleah Lok:** Writing – review and editing (equal). **Lin Zhang:** Writing – review and editing (equal). **Xu Lin:** Writing – review and editing (equal). **Don E. Lucero‐Prisno III:** Writing – review and editing (equal). **Wang‐Hong Xu:** Writing – review and editing (equal). **Zhi‐Jie Zheng:** Writing – review and editing (equal). **Edmar Elcarte:** Writing – review and editing (equal). **Mellissa Withers:** Writing – review and editing (equal). **Martin C. S. Wong:** Conceptualization (lead); supervision (lead); writing – review and editing (equal).

## Supporting information


Appendix S1.

## Data Availability

The data that supports the findings of this study are available from the corresponding author, upon reasonable request.
