# Supplementary material for: Disease burden, risk factors, and trends of lip, oral cavity, pharyngeal cancers: A global analysis
Source: Cancer Med. 2023 Jul 30;12(17):18153–64. doi: 10.1002/cam4.6391 (PMC10524054; doi:10.1002/cam4.6391)
Supplement: Supplementary file 1 — Appendix S1. [file CAM4-12-18153-s001.pdf]

Supplementary Legends

Supplementary Figure 1. Incidence and mortality trends for individual countries

Supplementary Figure 2. Results of joinpoint regression for individual countries

Supplementary Figure 3. AAPC of LOCP cancers incidence for individuals aged 50 years or above

Supplementary Figure 4. AAPC of LOCP cancers incidence for individuals aged < 50 years

Supplementary Figure 5. AAPC of LOCP cancers incidence for individuals aged < 40 years

Supplementary Table 1. ASR of LOCP cancers incidence and mortality by age group and subsite

Supplementary Figure 1: The plots of incidence and mortality trends for each country

Male

Asia

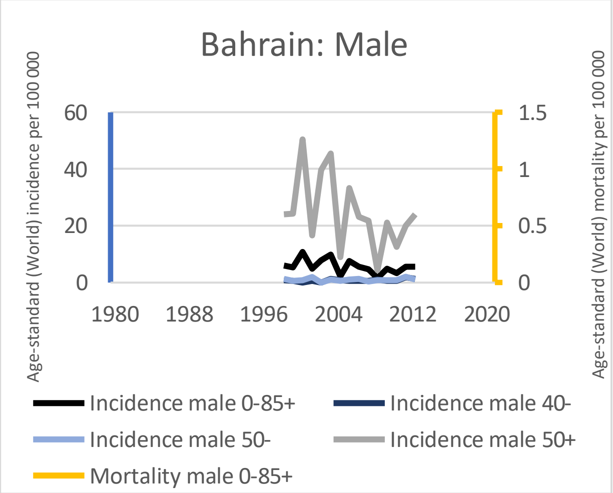

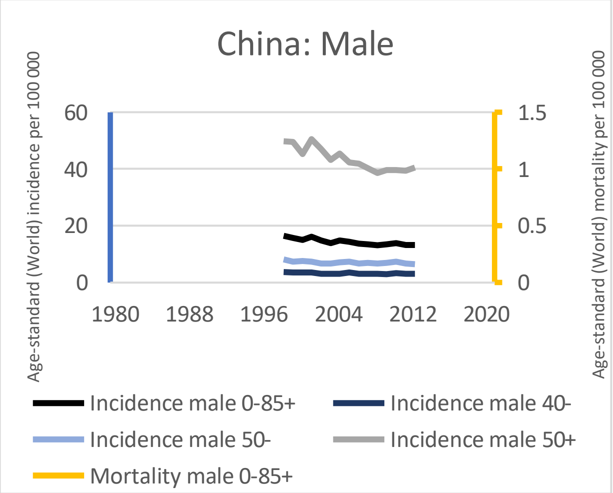

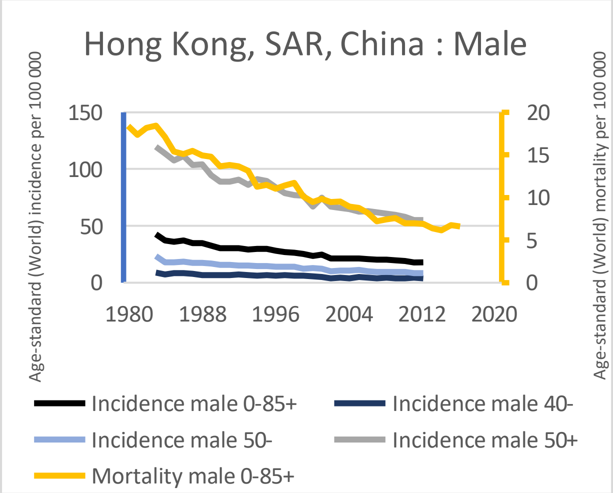

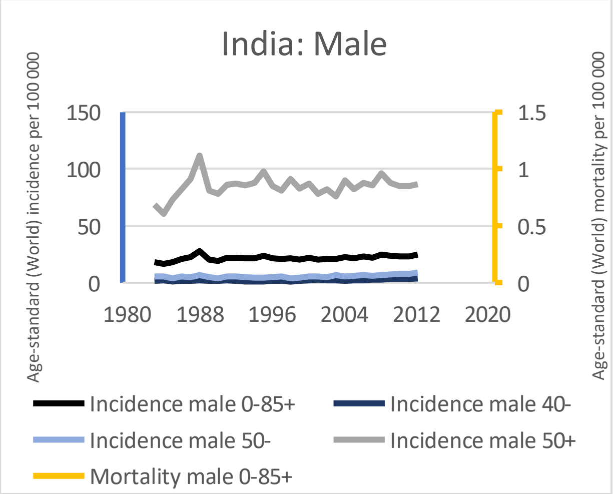

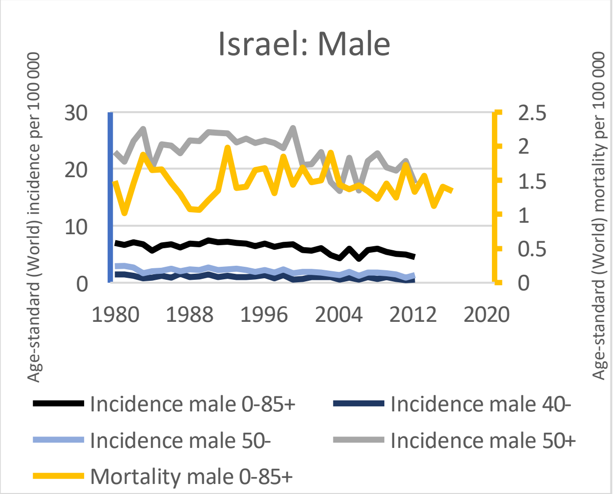

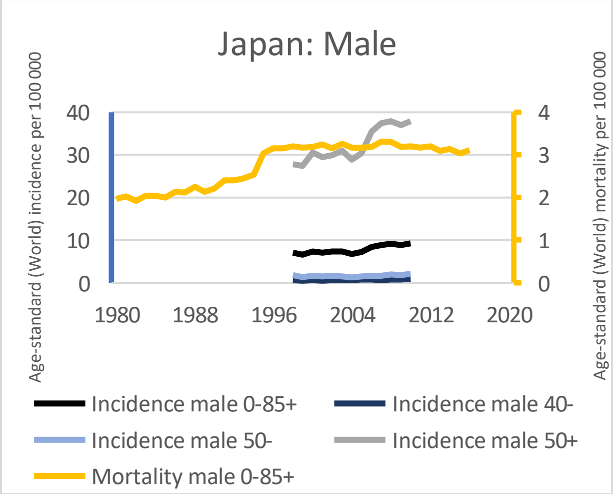

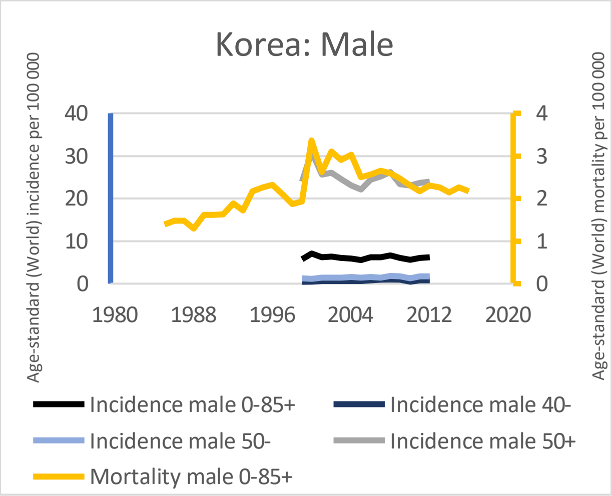
Korea: Male
Age-standard (World) incidence per 100 000
Age-standard (World) mortality per 100 000
40 30 20 10 0
4 3 2 1 0
1980 1988 1996 2004 2012 2020
Incidence male 0-85+
Incidence male 40-
Incidence male 50-
Incidence male 50+
Mortality male 0-85+

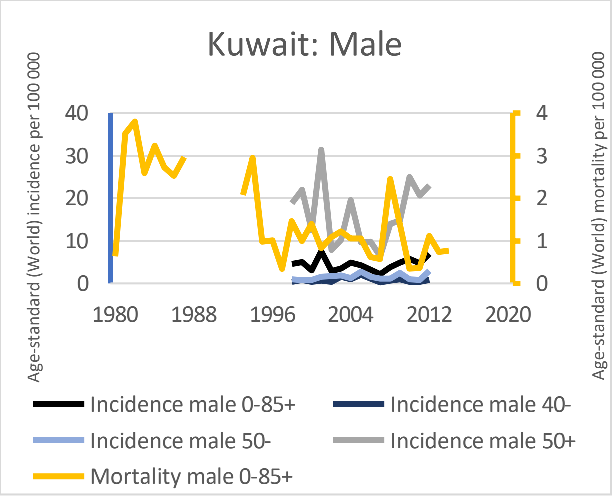
Kuwait: Male
Age-standard (World) incidence per 100 000
Age-standard (World) mortality per 100 000
40 30 20 10 0
4 3 2 1 0
1980 1988 1996 2004 2012 2020
Incidence male 0-85+
Incidence male 40-
Incidence male 50-
Incidence male 50+
Mortality male 0-85+

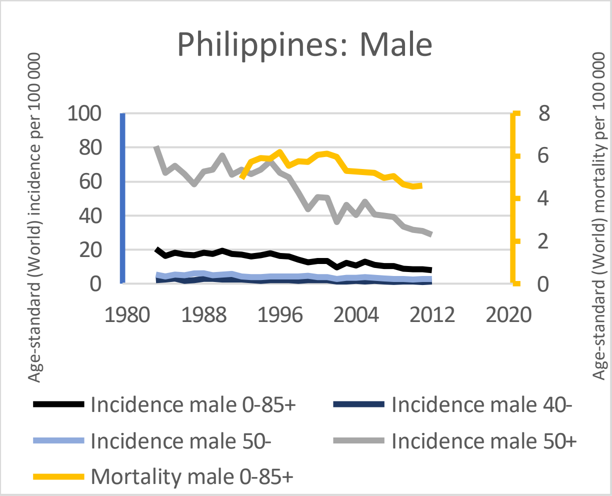
Philippines: Male
Age-standard (World) incidence per 100 000
Age-standard (World) mortality per 100 000
100 80 60 40 20 0
8 6 4 2 0
1980 1988 1996 2004 2012 2020
Incidence male 0-85+
Incidence male 40-
Incidence male 50-
Incidence male 50+
Mortality male 0-85+

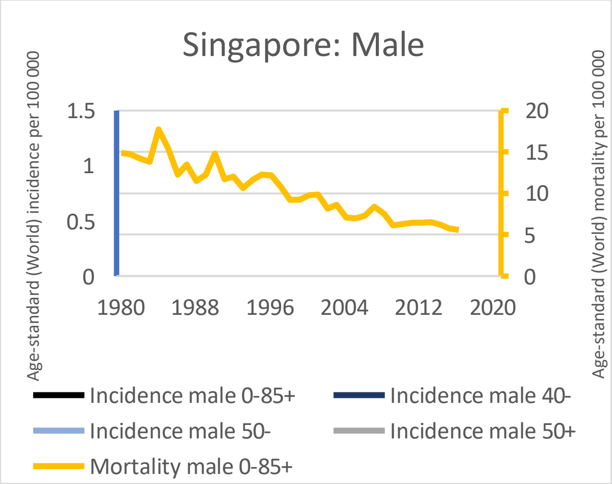
Singapore: Male
Age-standard (World) incidence per 100 000
Age-standard (World) mortality per 100 000
1.5 1 0.5 0
20 15 10 5 0
1980 1988 1996 2004 2012 2020
Incidence male 0-85+
Incidence male 40-
Incidence male 50-
Incidence male 50+
Mortality male 0-85+

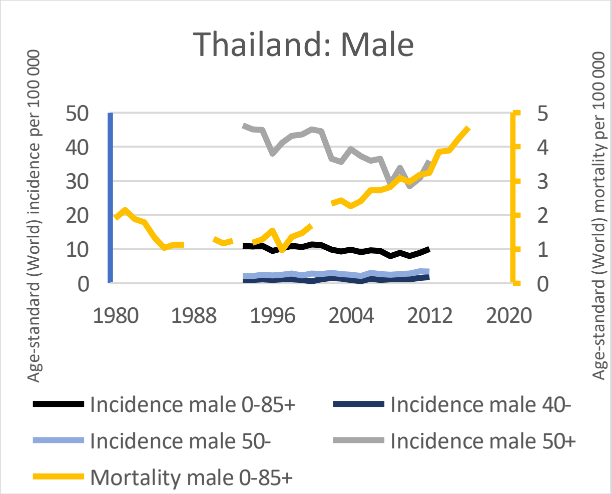
Thailand: Male
Age-standard (World) incidence per 100 000
Age-standard (World) mortality per 100 000
50 40 30 20 10 0
5 4 3 2 1 0
1980 1988 1996 2004 2012 2020
Incidence male 0-85+
Incidence male 40-
Incidence male 50-
Incidence male 50+
Mortality male 0-85+

Oceania

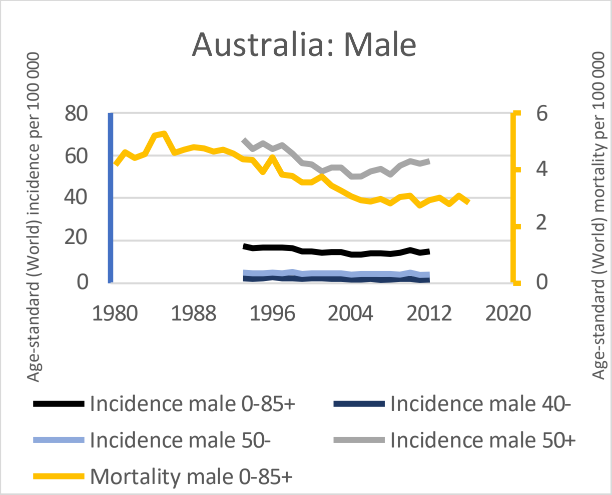

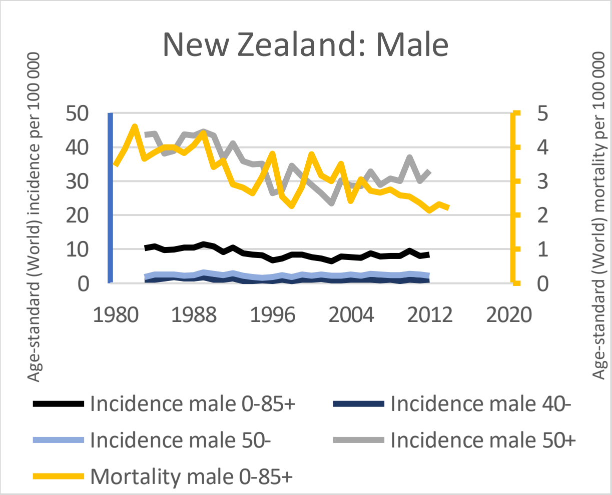

Northern America

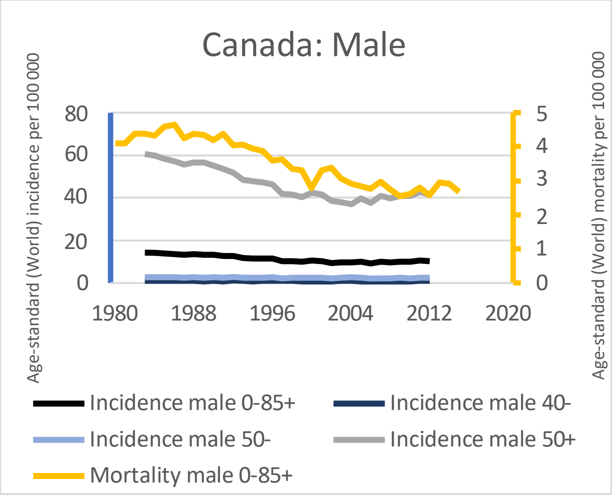

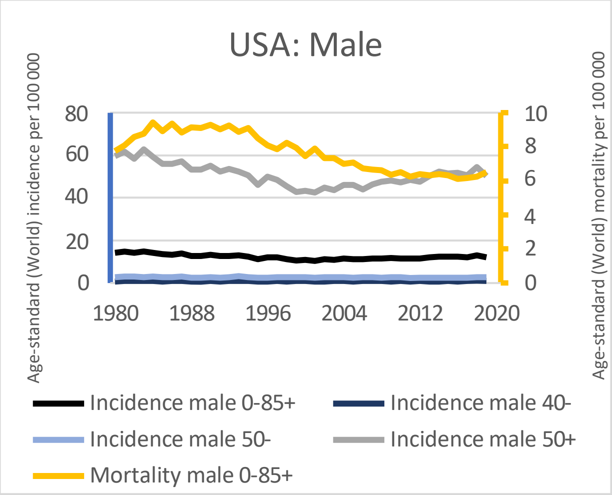

Southern America

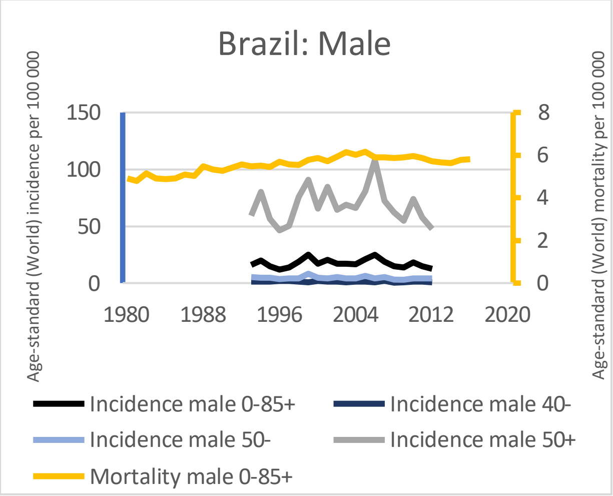

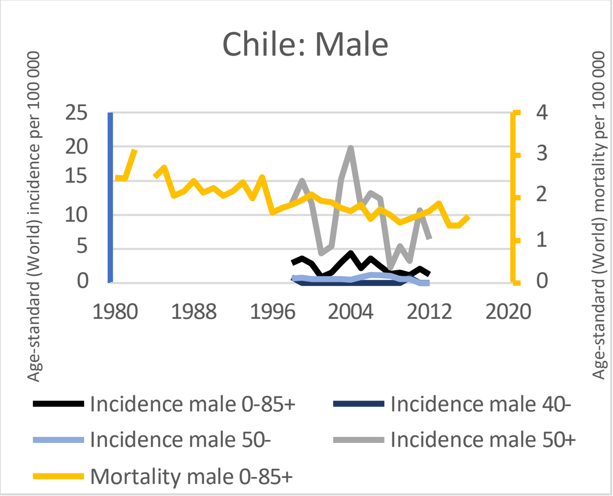

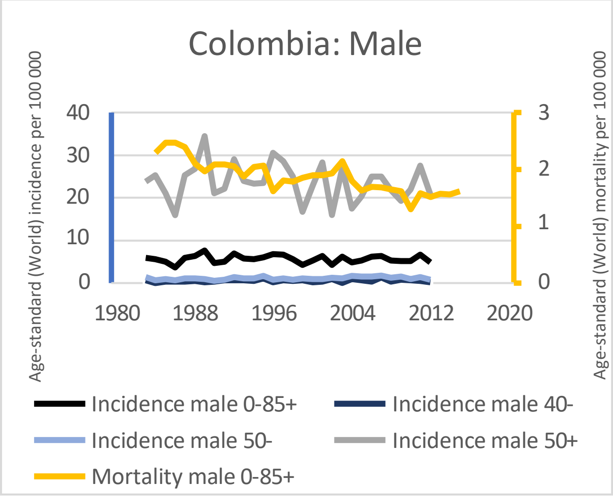

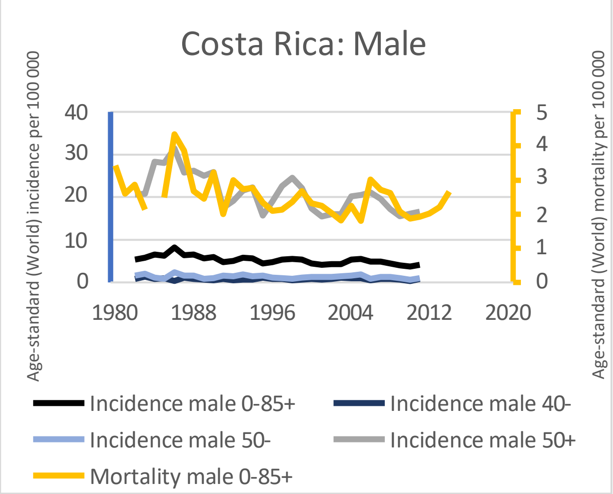

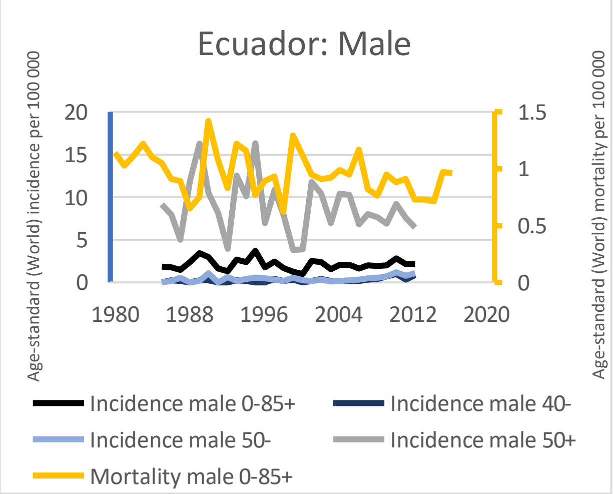

Northern Europe

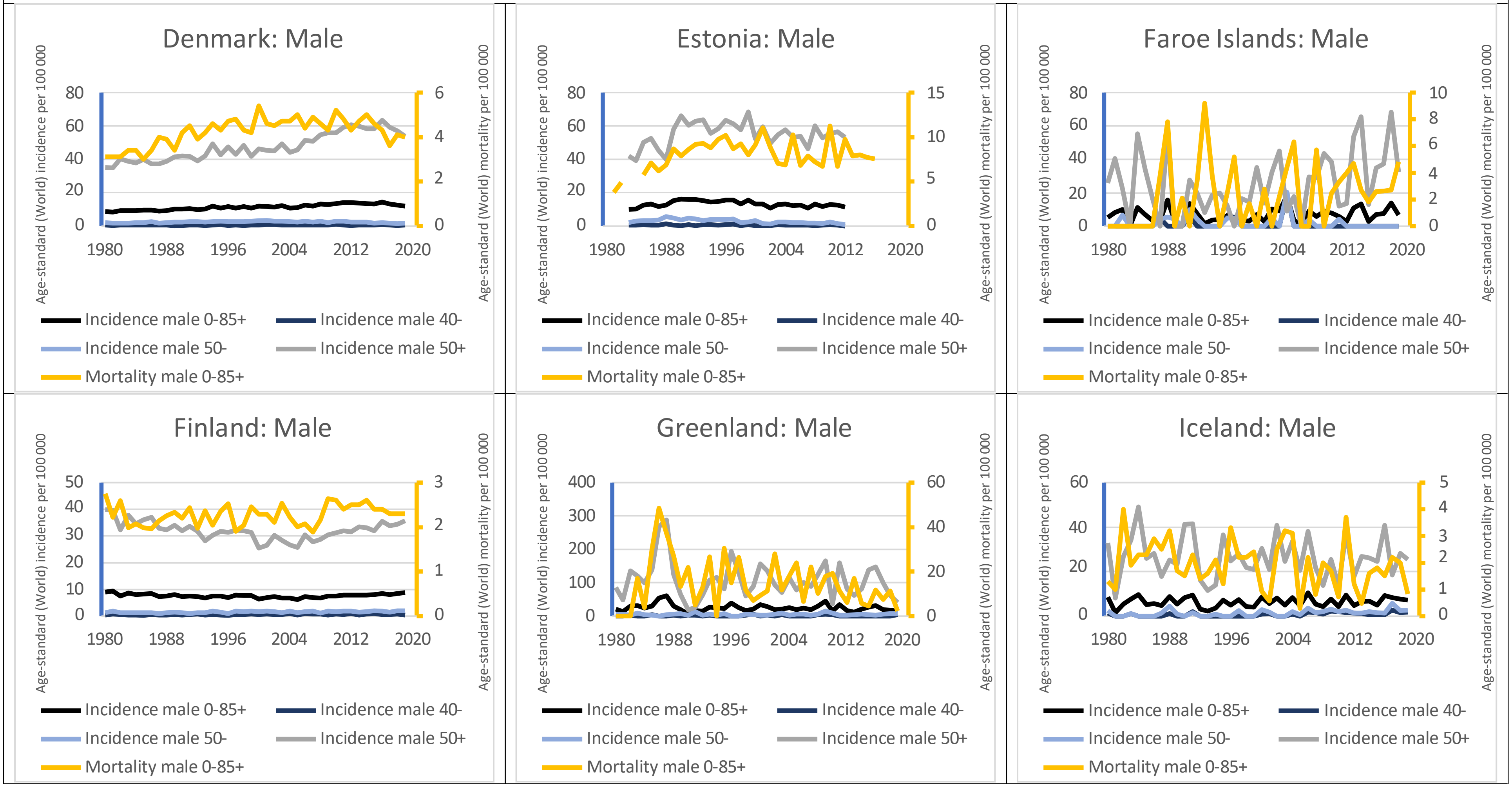

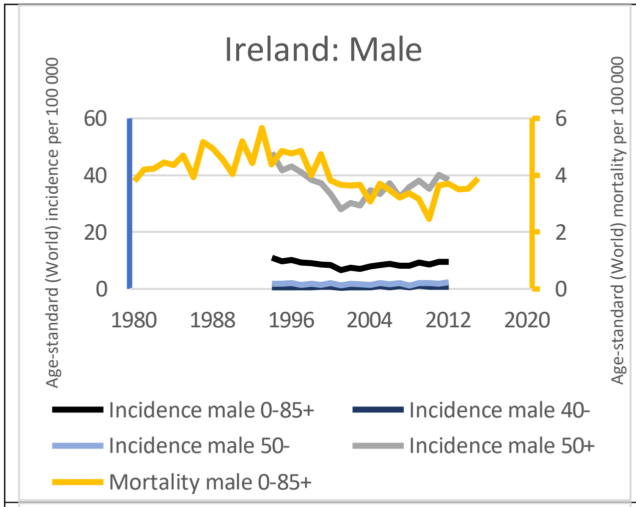
Ireland: Male
Age-standard (World) incidence per 100 000
Age-standard (World) mortality per 100 000
0
20
40
60
0
2
4
6
1980
1988
1996
2004
2012
2020
Incidence male 0-85+
Incidence male 40-
Incidence male 50-
Incidence male 50+
Mortality male 0-85+

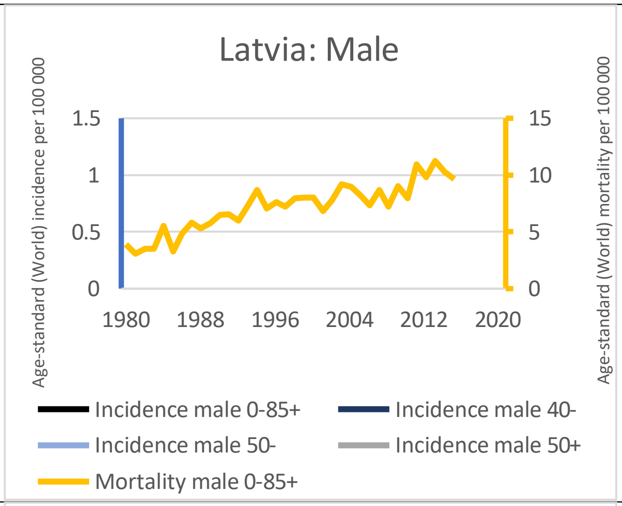
Latvia: Male
Age-standard (World) incidence per 100 000
Age-standard (World) mortality per 100 000
0
0.5
1
1.5
0
5
10
15
1980
1988
1996
2004
2012
2020
Incidence male 0-85+
Incidence male 40-
Incidence male 50-
Incidence male 50+
Mortality male 0-85+

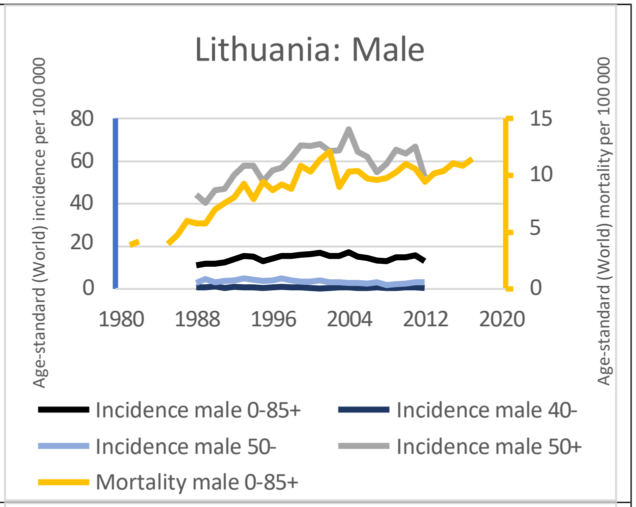
Lithuania: Male
Age-standard (World) incidence per 100 000
Age-standard (World) mortality per 100 000
0
20
40
60
80
0
5
10
15
1980
1988
1996
2004
2012
2020
Incidence male 0-85+
Incidence male 40-
Incidence male 50-
Incidence male 50+
Mortality male 0-85+

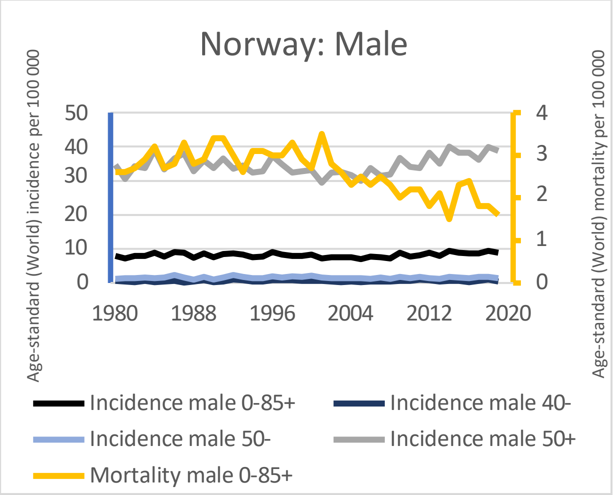
Norway: Male
Age-standard (World) incidence per 100 000
Age-standard (World) mortality per 100 000
0
10
20
30
40
50
0
1
2
3
4
1980
1988
1996
2004
2012
2020
Incidence male 0-85+
Incidence male 40-
Incidence male 50-
Incidence male 50+
Mortality male 0-85+

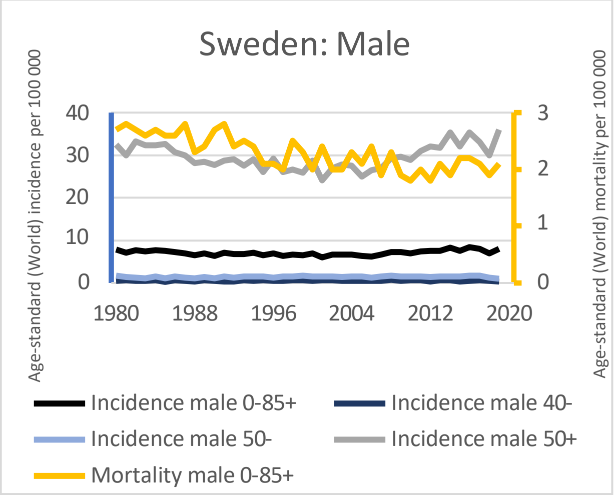
Sweden: Male
Age-standard (World) incidence per 100 000
Age-standard (World) mortality per 100 000
0
10
20
30
40
0
1
2
3
1980
1988
1996
2004
2012
2020
Incidence male 0-85+
Incidence male 40-
Incidence male 50-
Incidence male 50+
Mortality male 0-85+

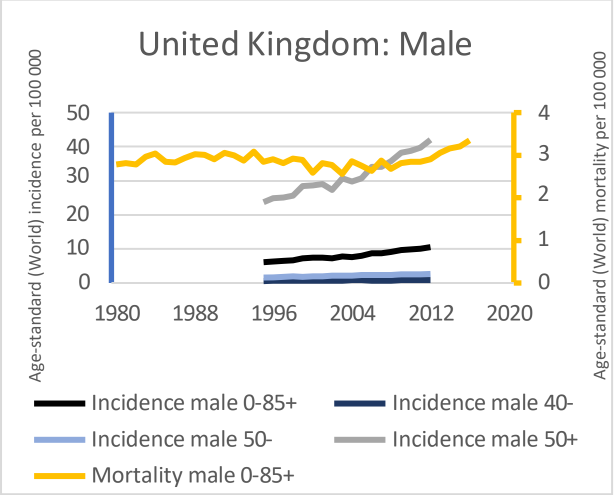
United Kingdom: Male
Age-standard (World) incidence per 100 000
Age-standard (World) mortality per 100 000
0
10
20
30
40
50
0
1
2
3
4
1980
1988
1996
2004
2012
2020
Incidence male 0-85+
Incidence male 40-
Incidence male 50-
Incidence male 50+
Mortality male 0-85+

Western Europe

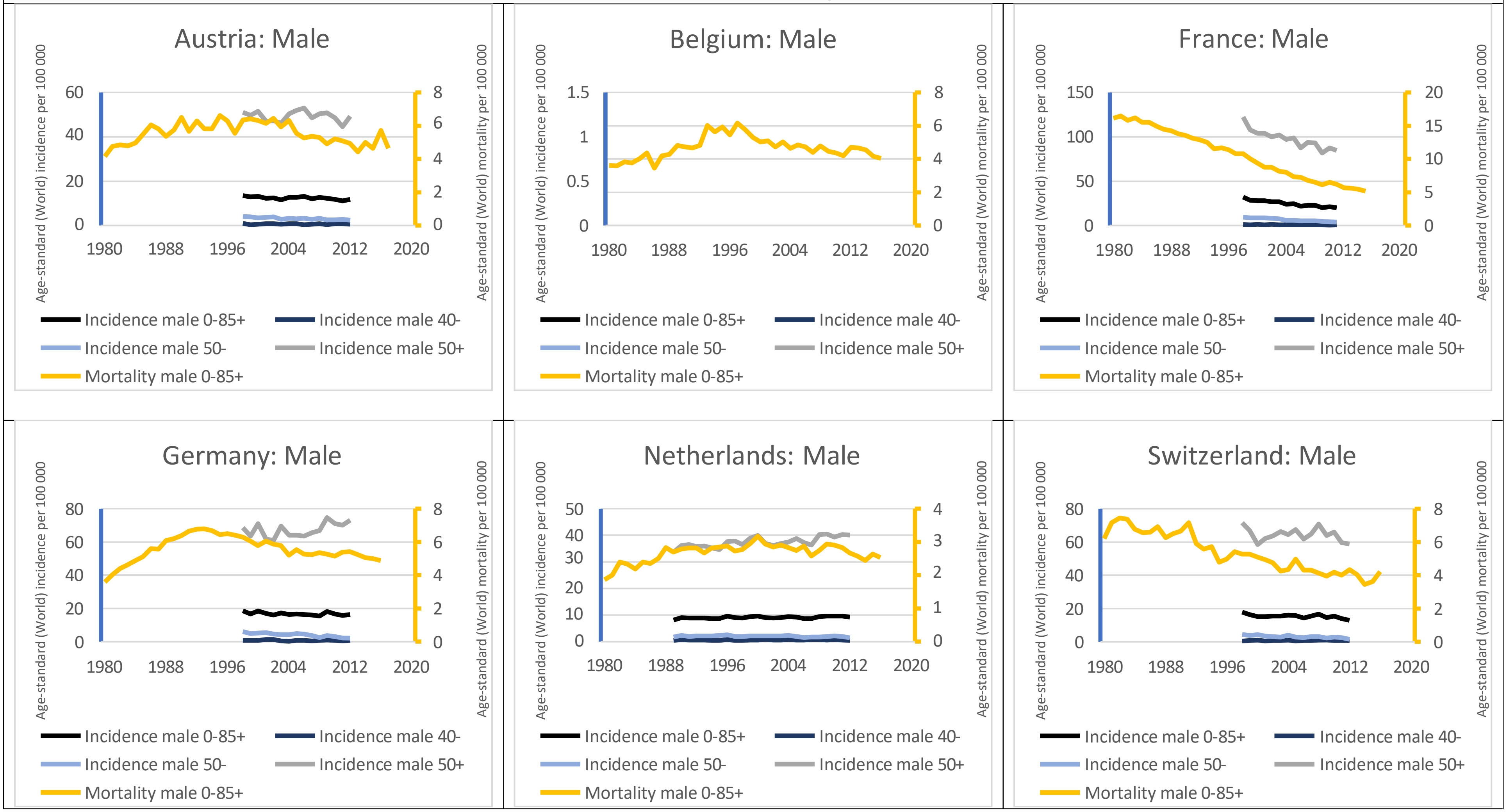

Southern Europe

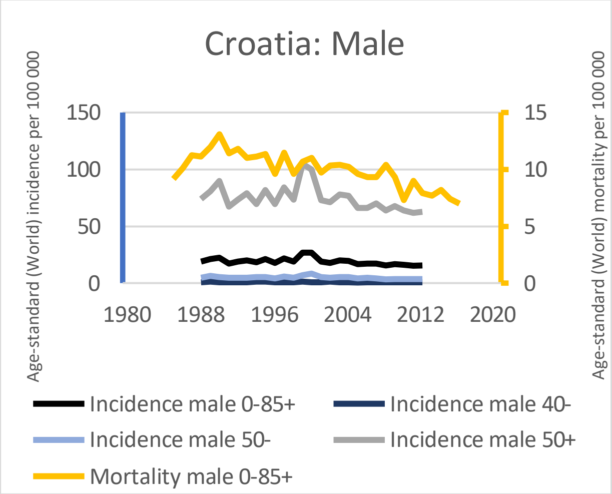

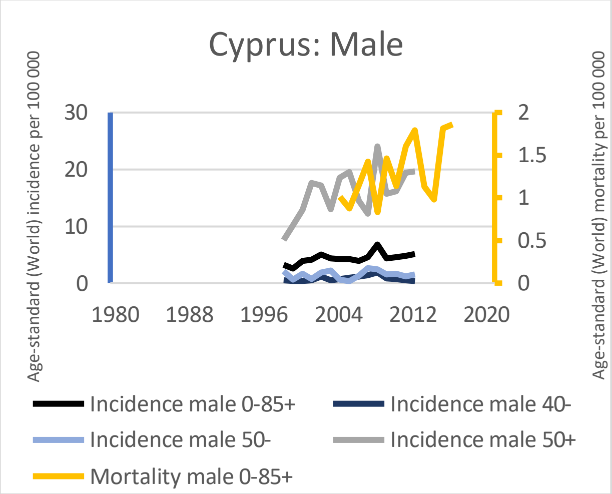

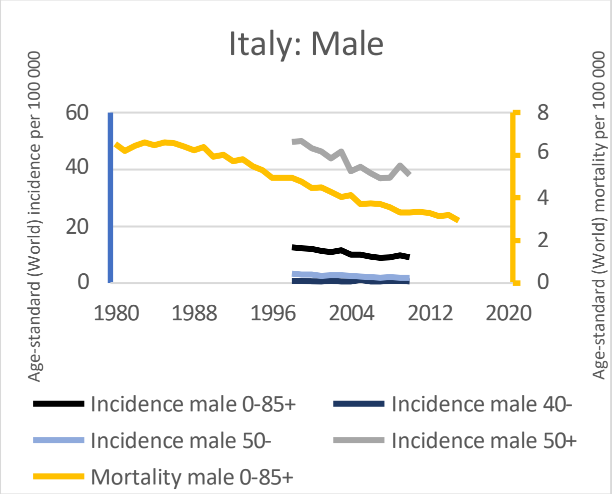

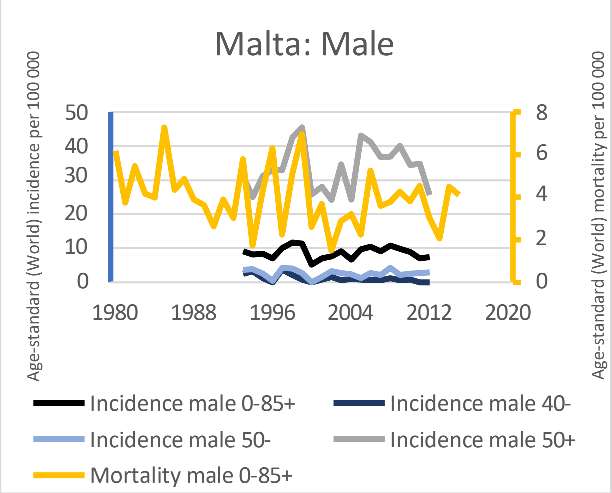

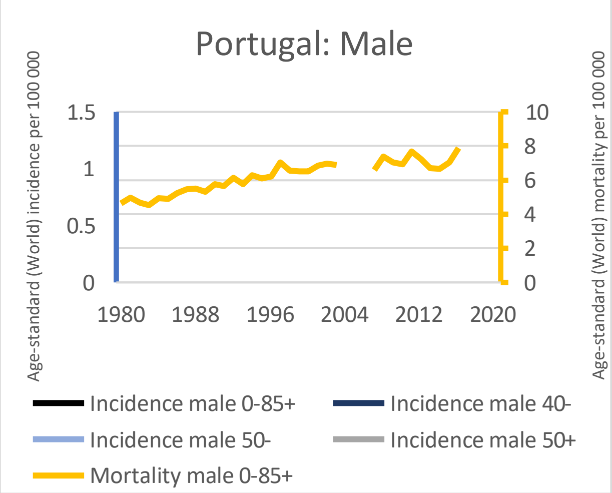

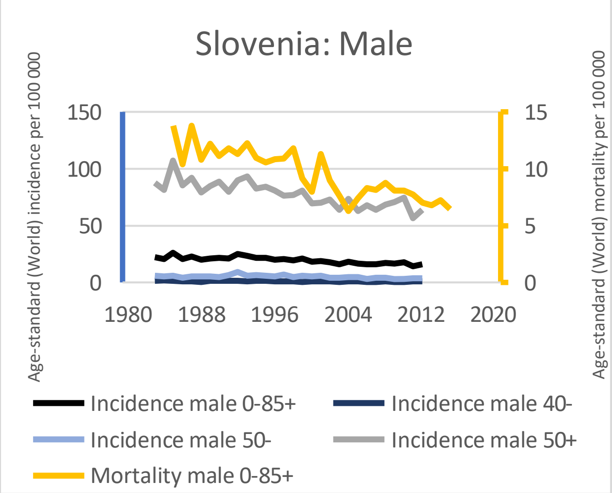

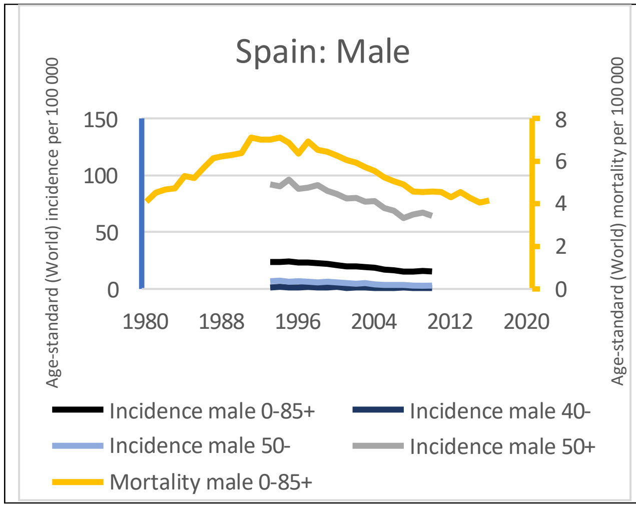
Spain: Male
Age-standard (World) incidence per 100 000
Age-standard (World) mortality per 100 000
150
100
50
0
8
6
4
2
0
1980
1988
1996
2004
2012
2020
Incidence male 0-85+
Incidence male 40-
Incidence male 50-
Incidence male 50+
Mortality male 0-85+

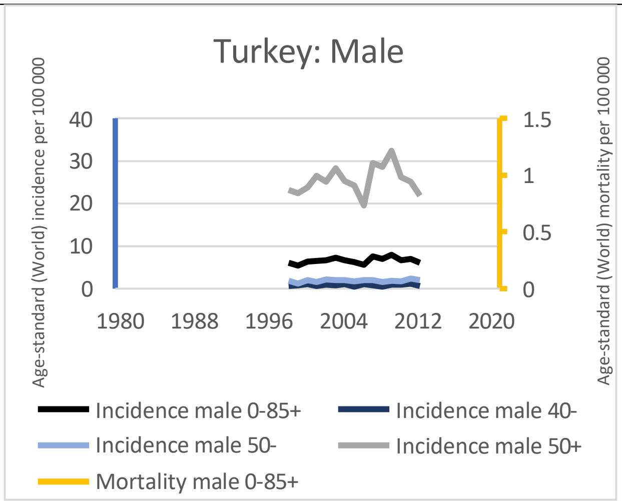
Turkey: Male
Age-standard (World) incidence per 100 000
Age-standard (World) mortality per 100 000
40
30
20
10
0
1.5
1
0.5
0
1980
1988
1996
2004
2012
2020
Incidence male 0-85+
Incidence male 40-
Incidence male 50-
Incidence male 50+
Mortality male 0-85+

Eastern Europe

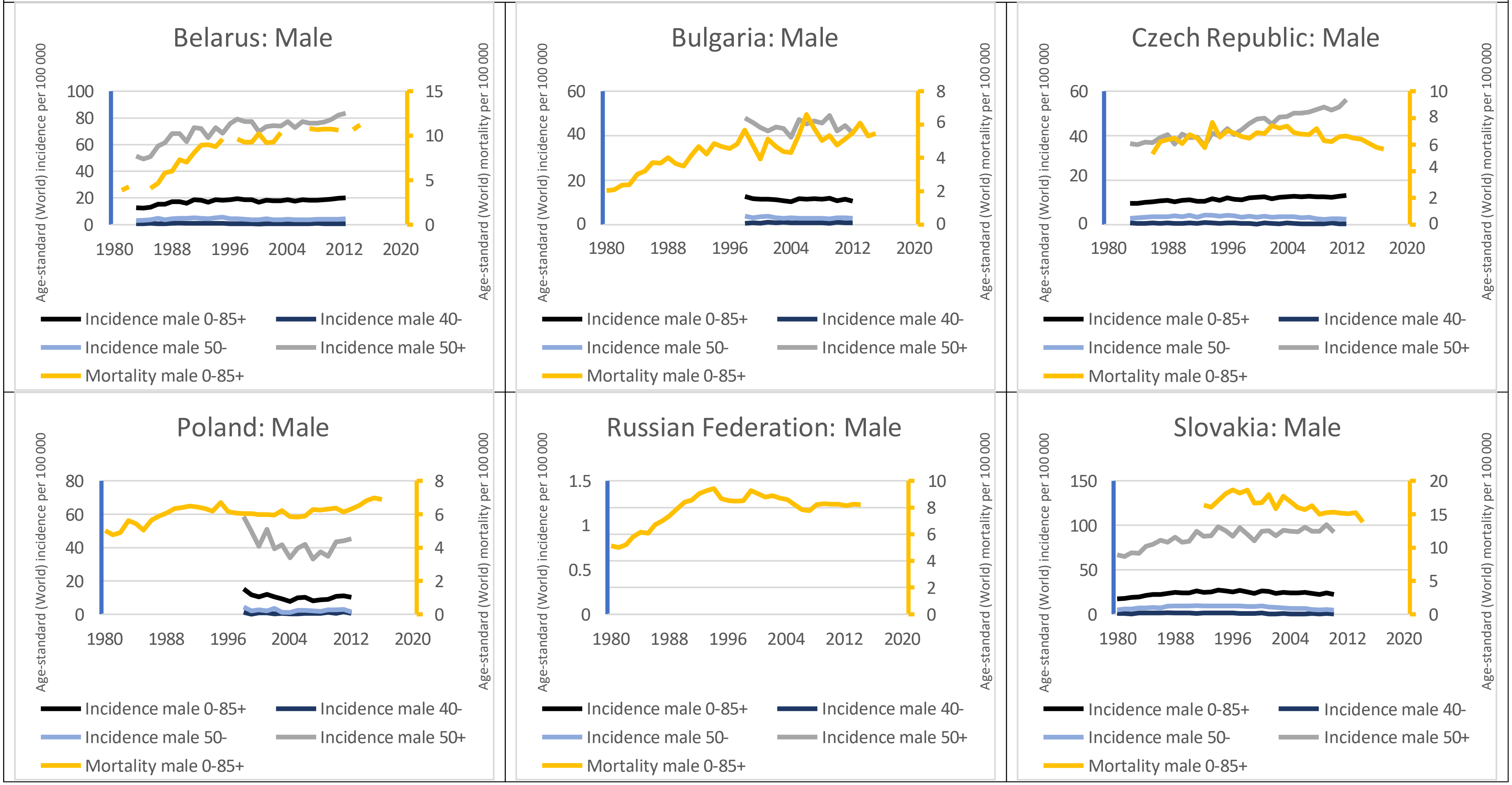

Africa		
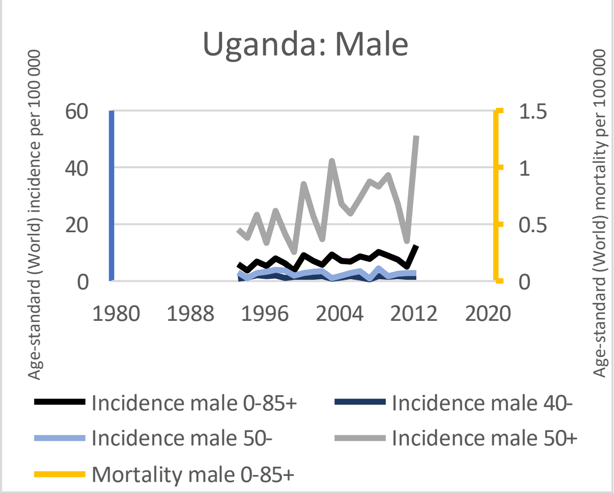Uganda: Male<br>Age-standard (World) incidence per 100 000<br>Age-standard (World) mortality per 100 000<br>60<br>40<br>20<br>0<br>1.5<br>1<br>0.5<br>0<br>1980<br>1988<br>1996<br>2004<br>2012<br>2020<br>Incidence male 0-85+<br>Incidence male 40-<br>Incidence male 50-<br>Incidence male 50+<br>Mortality male 0-85+		

Female

Asia

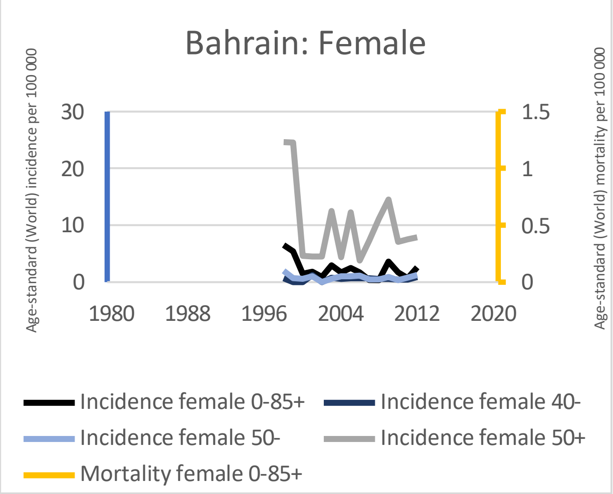
Bahrain: Female
Age-standard (World) incidence per 100 000
Age-standard (World) mortality per 100 000
30
20
10
0
1.5
1
0.5
0
1980
1988
1996
2004
2012
2020
Incidence female 0-85+
Incidence female 40-
Incidence female 50-
Incidence female 50+
Mortality female 0-85+

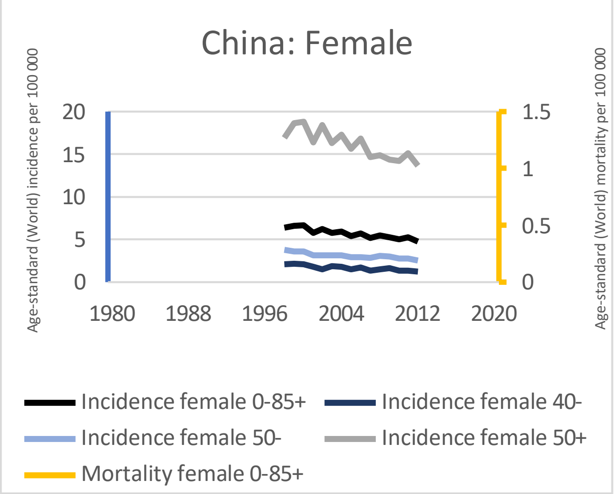
China: Female
Age-standard (World) incidence per 100 000
Age-standard (World) mortality per 100 000
20
15
10
5
0
1.5
1
0.5
0
1980
1988
1996
2004
2012
2020
Incidence female 0-85+
Incidence female 40-
Incidence female 50-
Incidence female 50+
Mortality female 0-85+

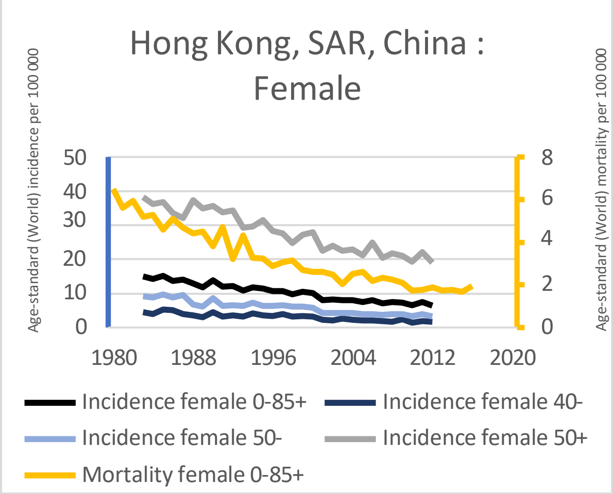
Hong Kong, SAR, China : Female
Age-standard (World) incidence per 100 000
Age-standard (World) mortality per 100 000
50
40
30
20
10
0
8
6
4
2
0
1980
1988
1996
2004
2012
2020
Incidence female 0-85+
Incidence female 40-
Incidence female 50-
Incidence female 50+
Mortality female 0-85+

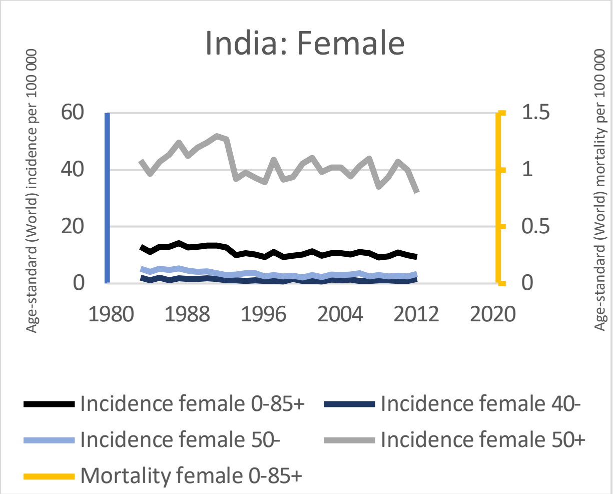
India: Female
Age-standard (World) incidence per 100 000
Age-standard (World) mortality per 100 000
60
40
20
0
1.5
1
0.5
0
1980
1988
1996
2004
2012
2020
Incidence female 0-85+
Incidence female 40-
Incidence female 50-
Incidence female 50+
Mortality female 0-85+

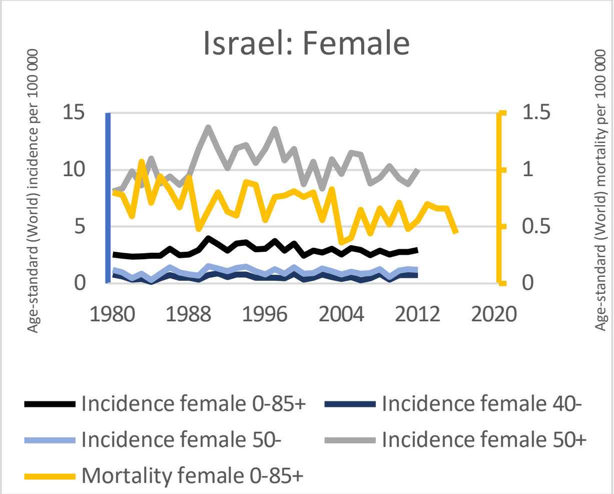
Israel: Female
Age-standard (World) incidence per 100 000
Age-standard (World) mortality per 100 000
15
10
5
0
1.5
1
0.5
0
1980
1988
1996
2004
2012
2020
Incidence female 0-85+
Incidence female 40-
Incidence female 50-
Incidence female 50+
Mortality female 0-85+

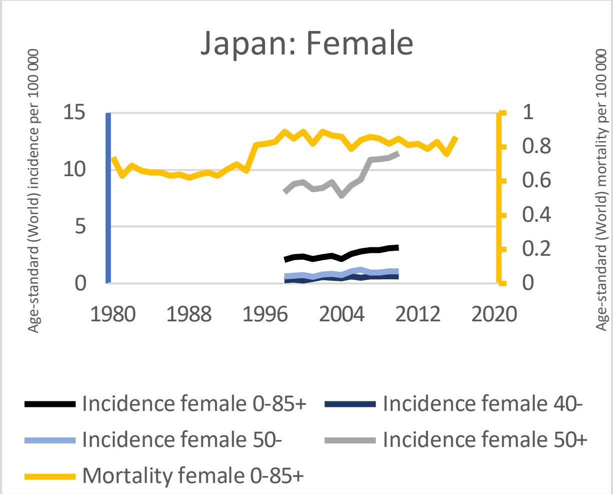
Japan: Female
Age-standard (World) incidence per 100 000
Age-standard (World) mortality per 100 000
15
10
5
0
1
0.8
0.6
0.4
0.2
0
1980
1988
1996
2004
2012
2020
Incidence female 0-85+
Incidence female 40-
Incidence female 50-
Incidence female 50+
Mortality female 0-85+

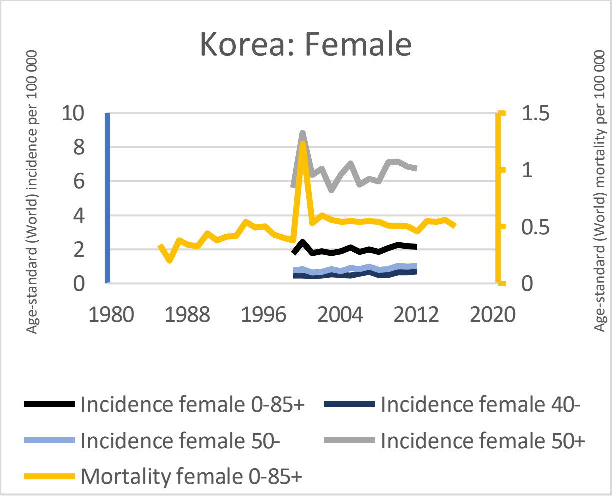
Korea: Female
Age-standard (World) incidence per 100 000
Age-standard (World) mortality per 100 000
10 8 6 4 2 0
1.5 1 0.5 0
1980 1988 1996 2004 2012 2020
Incidence female 0-85+
Incidence female 40-
Incidence female 50-
Incidence female 50+
Mortality female 0-85+

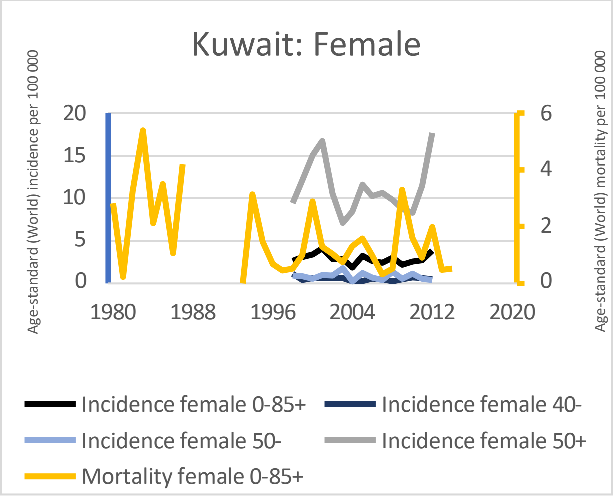
Kuwait: Female
Age-standard (World) incidence per 100 000
Age-standard (World) mortality per 100 000
20 15 10 5 0
6 4 2 0
1980 1988 1996 2004 2012 2020
Incidence female 0-85+
Incidence female 40-
Incidence female 50-
Incidence female 50+
Mortality female 0-85+

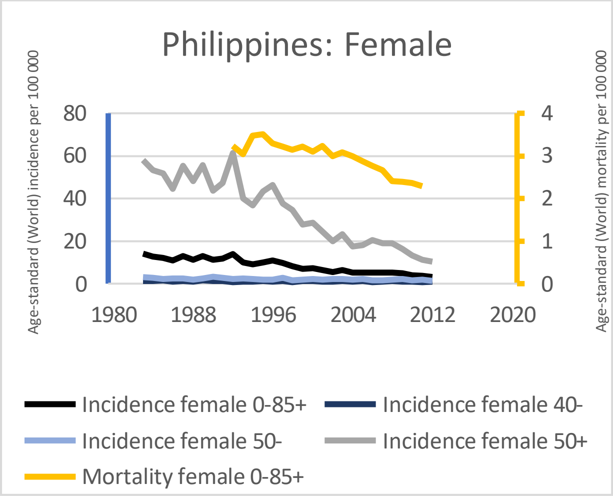
Philippines: Female
Age-standard (World) incidence per 100 000
Age-standard (World) mortality per 100 000
80 60 40 20 0
4 3 2 1 0
1980 1988 1996 2004 2012 2020
Incidence female 0-85+
Incidence female 40-
Incidence female 50-
Incidence female 50+
Mortality female 0-85+

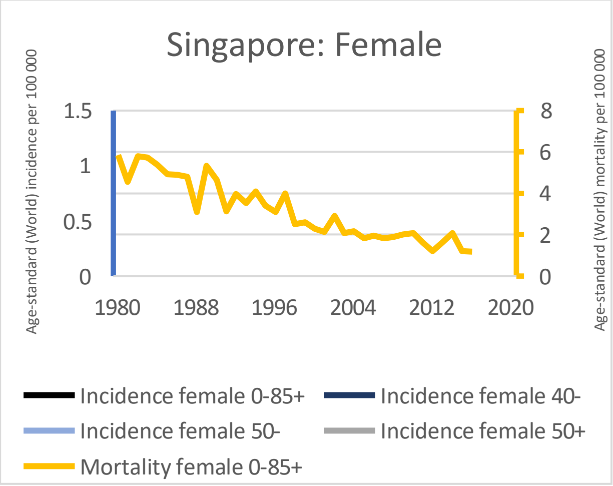
Singapore: Female
Age-standard (World) incidence per 100 000
Age-standard (World) mortality per 100 000
1.5 1 0.5 0
8 6 4 2 0
1980 1988 1996 2004 2012 2020
Incidence female 0-85+
Incidence female 40-
Incidence female 50-
Incidence female 50+
Mortality female 0-85+

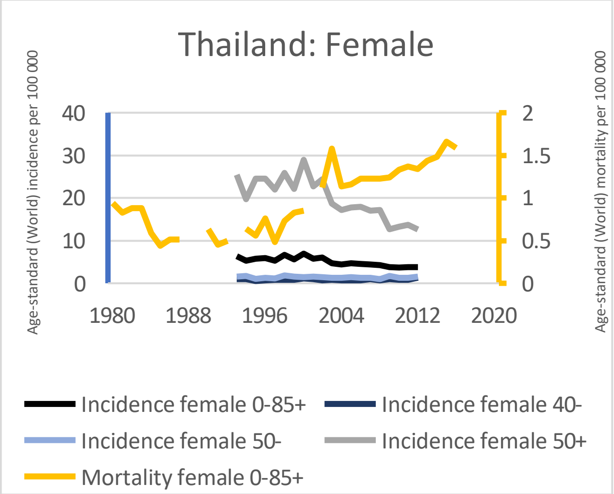
Thailand: Female
Age-standard (World) incidence per 100 000
Age-standard (World) mortality per 100 000
40 30 20 10 0
2 1.5 1 0.5 0
1980 1988 1996 2004 2012 2020
Incidence female 0-85+
Incidence female 40-
Incidence female 50-
Incidence female 50+
Mortality female 0-85+

Oceania

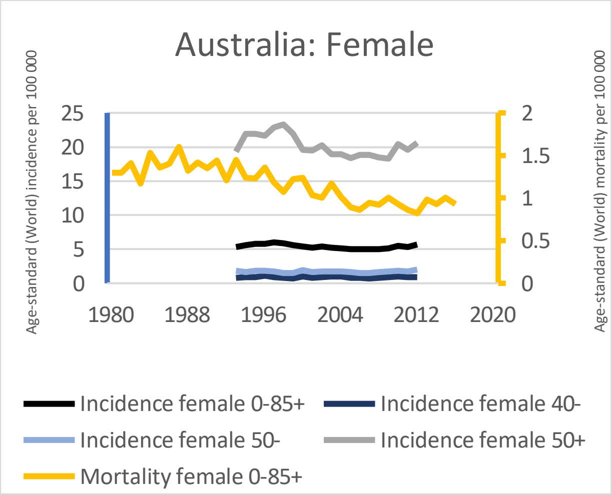

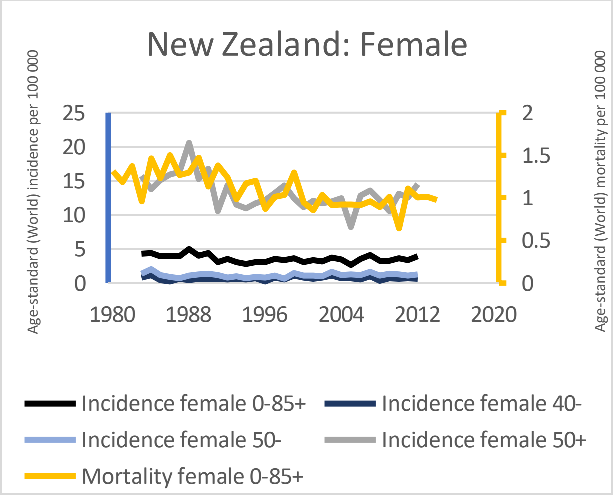

Northern America

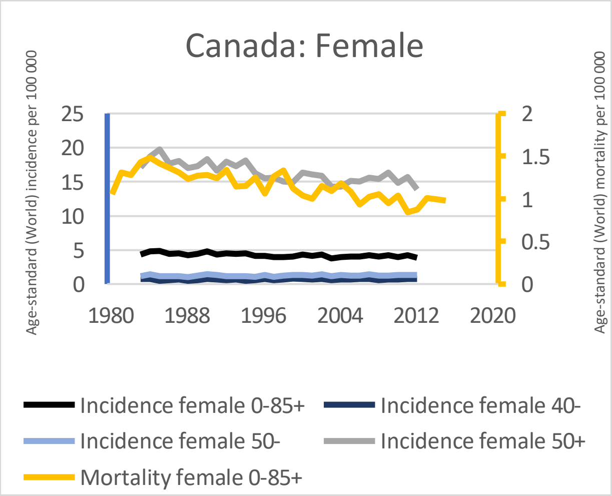

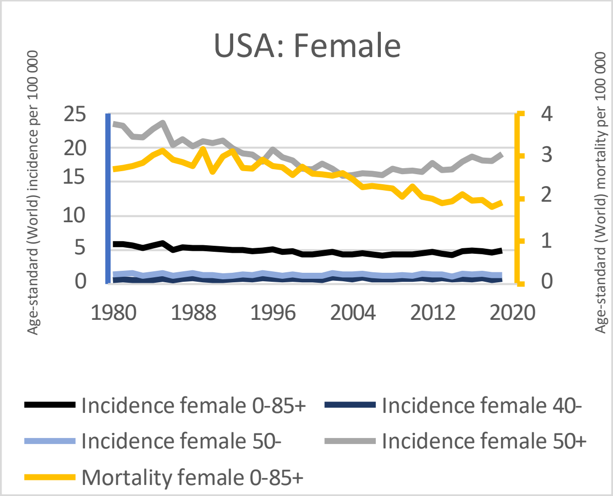

Southern America

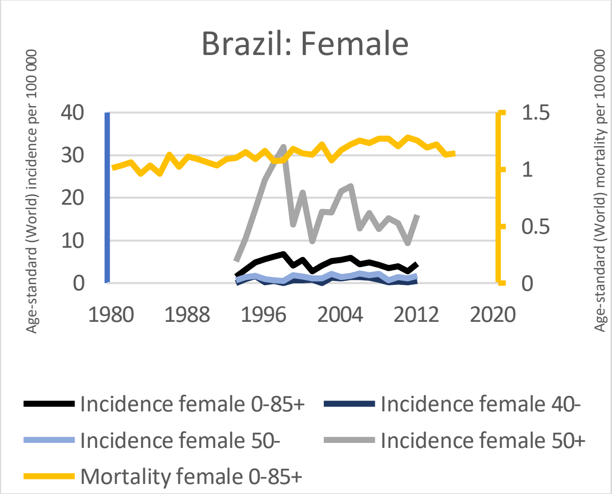

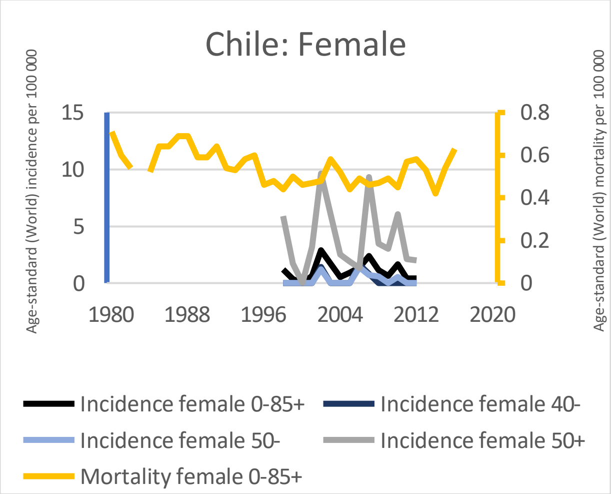

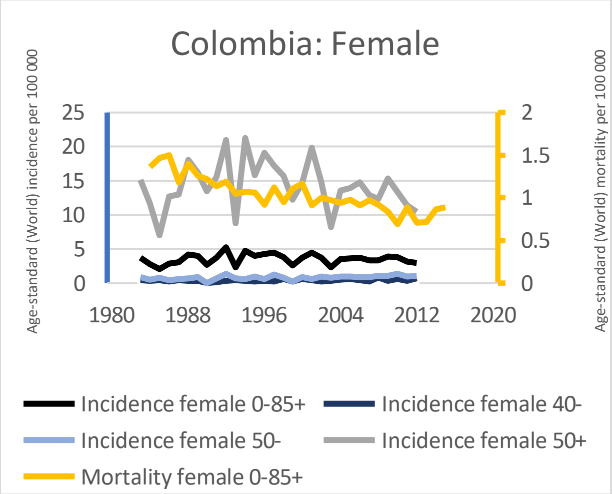

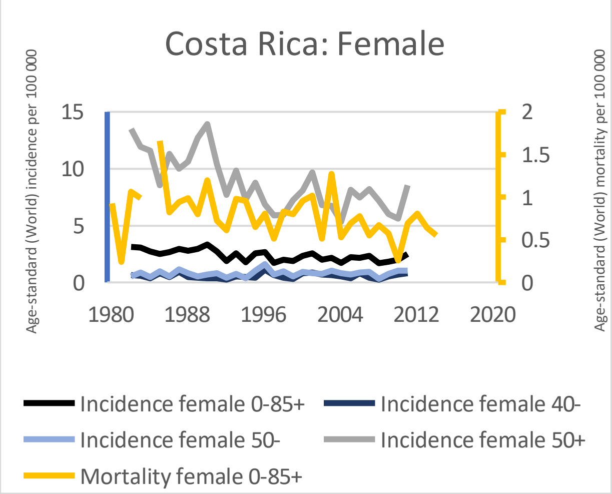

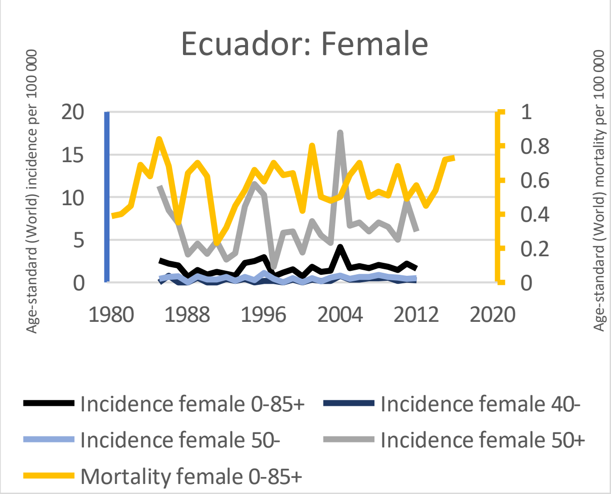

Northern Europe

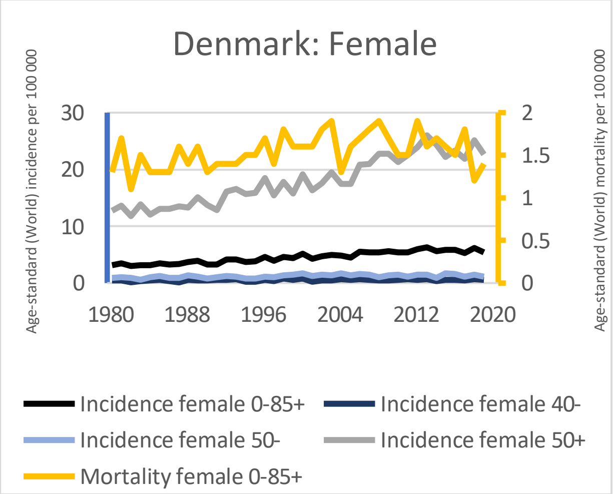

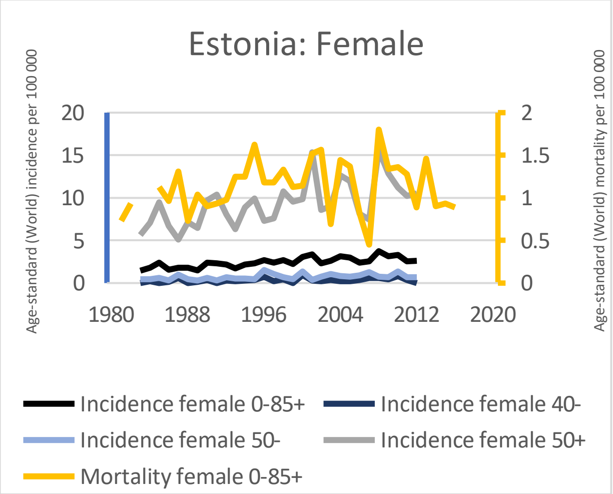

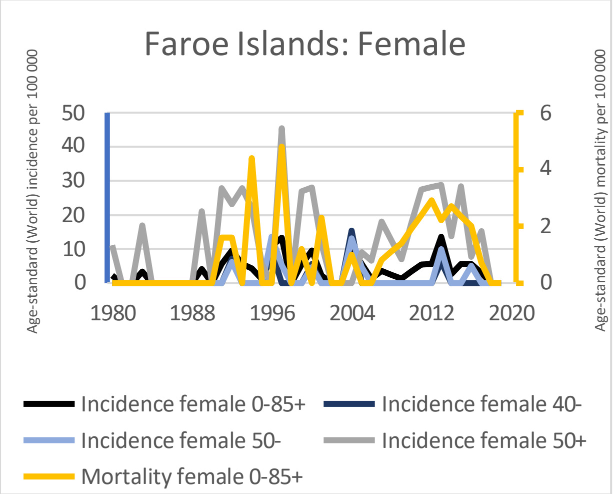

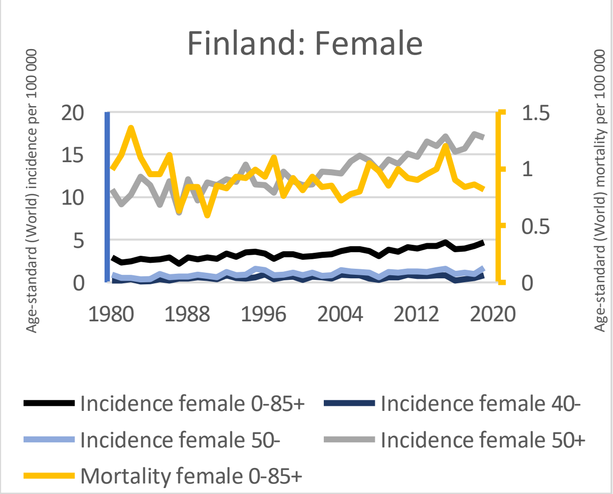

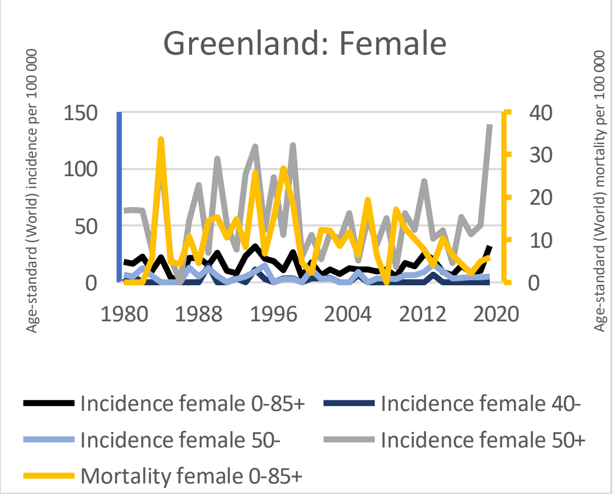

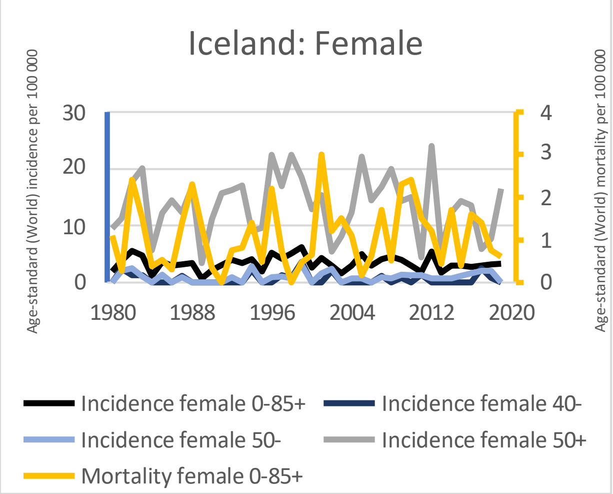

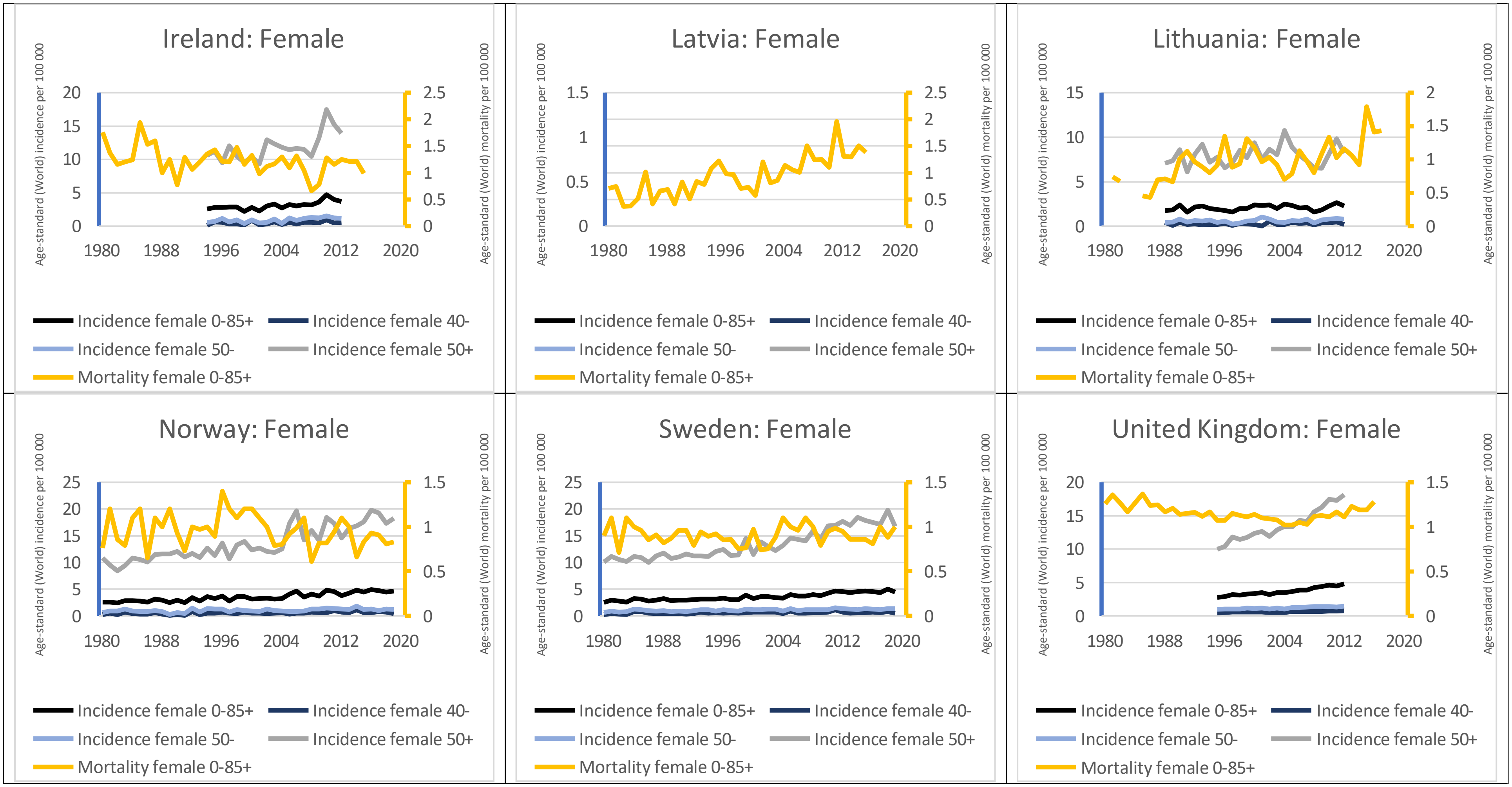

Ireland: Female
Latvia: Female
Lithuania: Female
Norway: Female
Sweden: Female
United Kingdom: Female
Age-standard (World) incidence per 100 000
Age-standard (World) mortality per 100 000
1980
1988
1996
2004
2012
2020
Incidence female 0-85+
Incidence female 40-
Incidence female 50-
Incidence female 50+
Mortality female 0-85+

Western Europe

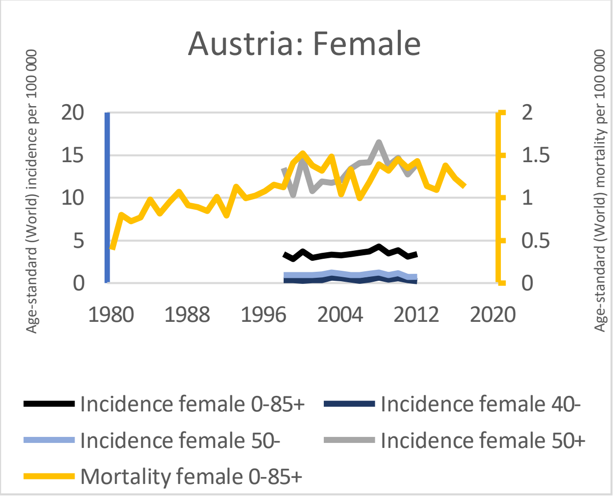

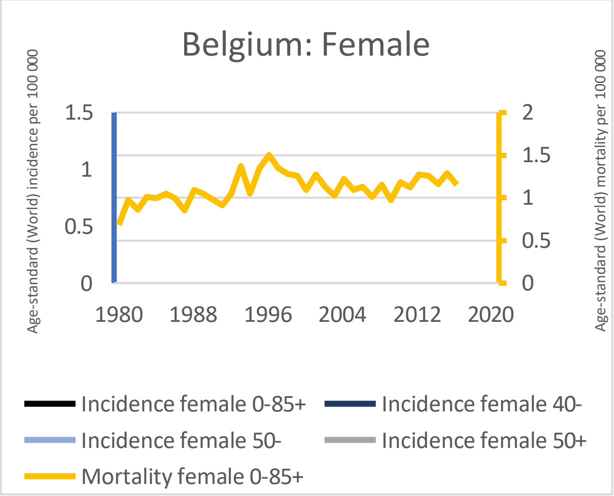

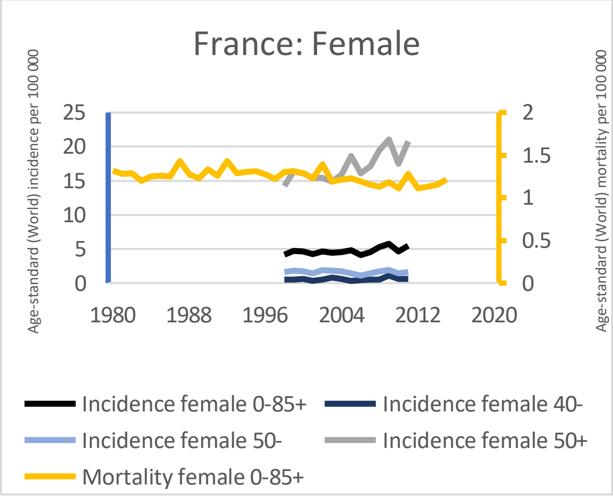

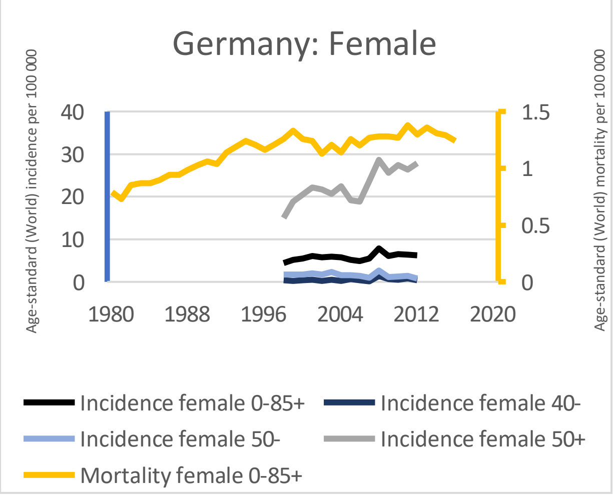

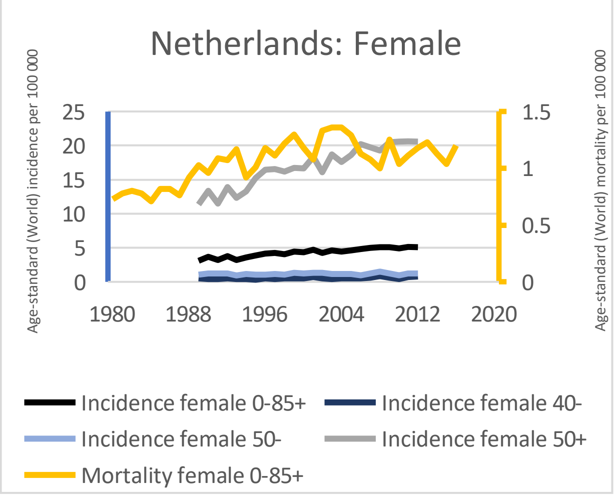

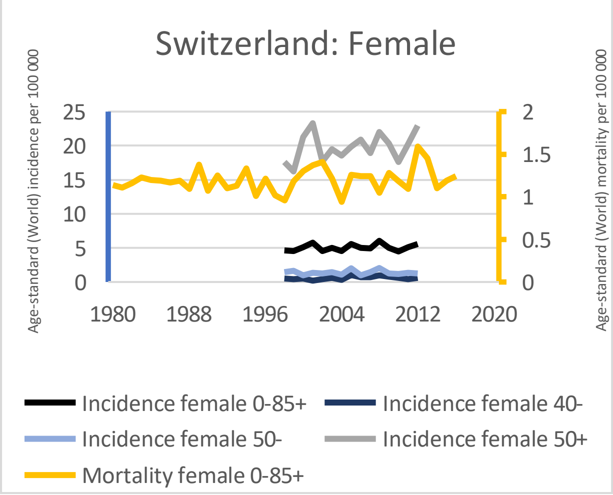

Southern Europe

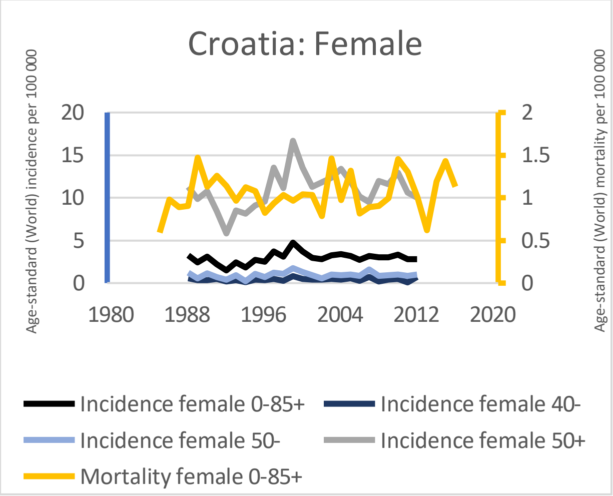

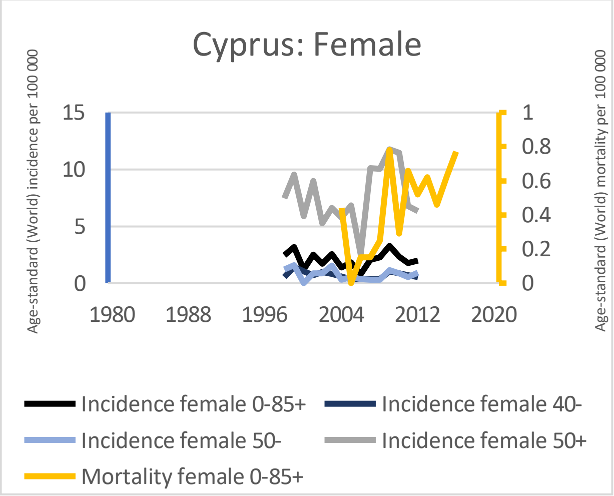

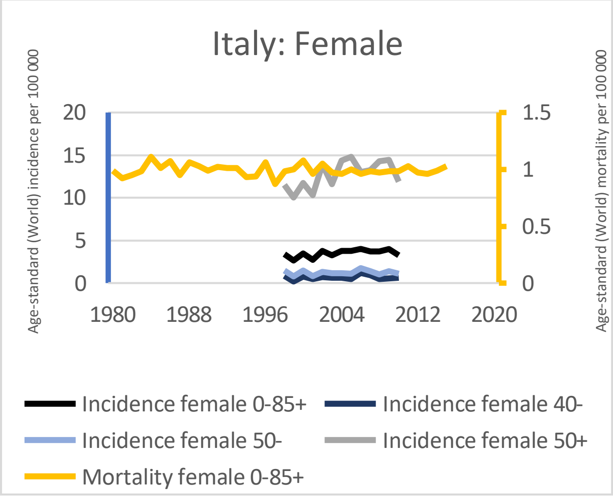

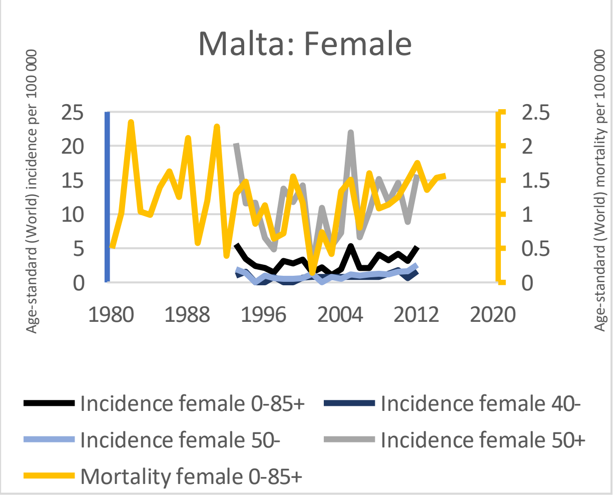

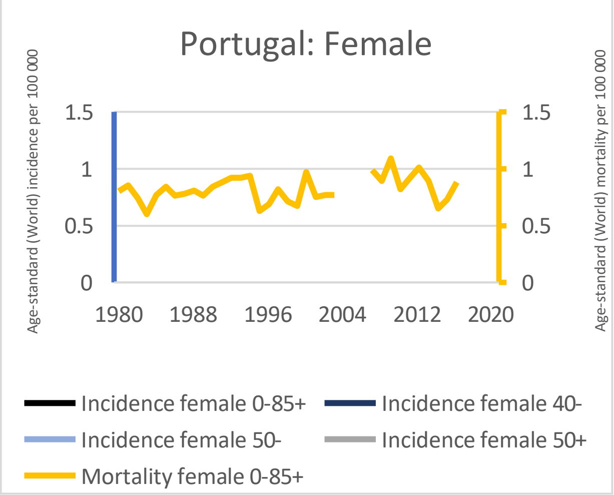

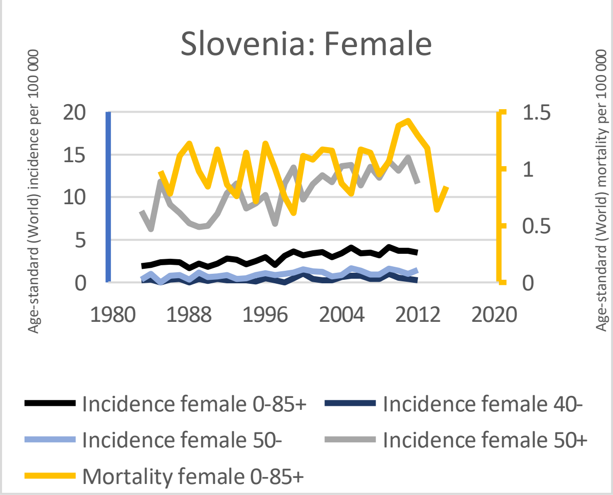

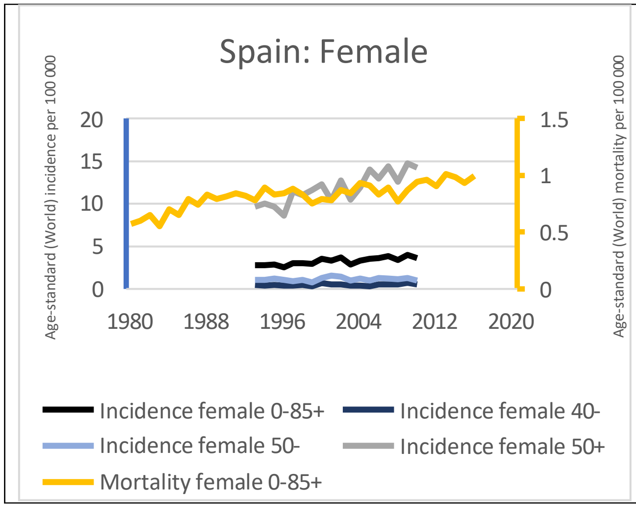
Spain: Female
Age-standard (World) incidence per 100 000
Age-standard (World) mortality per 100 000
20
15
10
5
0
1.5
1
0.5
0
1980
1988
1996
2004
2012
2020
Incidence female 0-85+
Incidence female 40-
Incidence female 50-
Incidence female 50+
Mortality female 0-85+

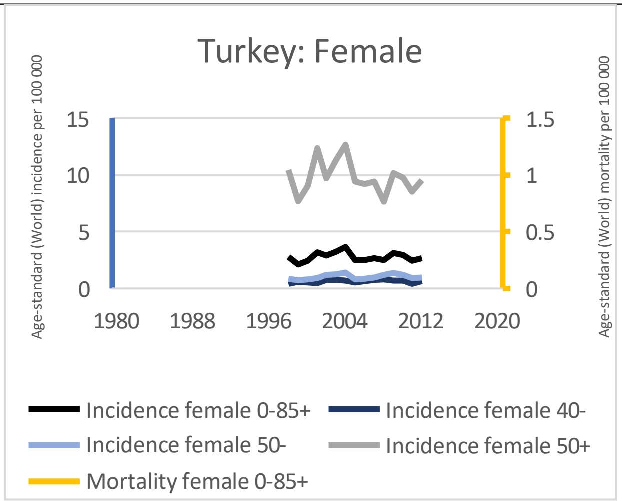
Turkey: Female
Age-standard (World) incidence per 100 000
Age-standard (World) mortality per 100 000
15
10
5
0
1.5
1
0.5
0
1980
1988
1996
2004
2012
2020
Incidence female 0-85+
Incidence female 40-
Incidence female 50-
Incidence female 50+
Mortality female 0-85+

Eastern Europe

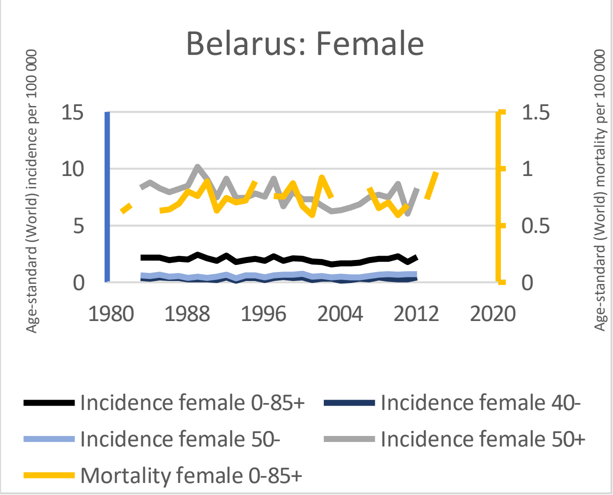

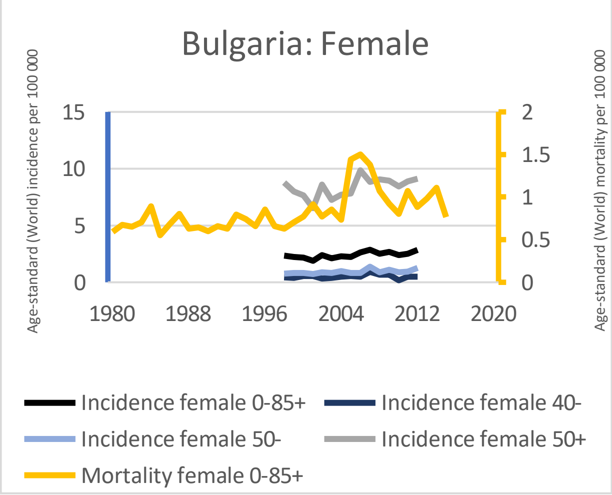

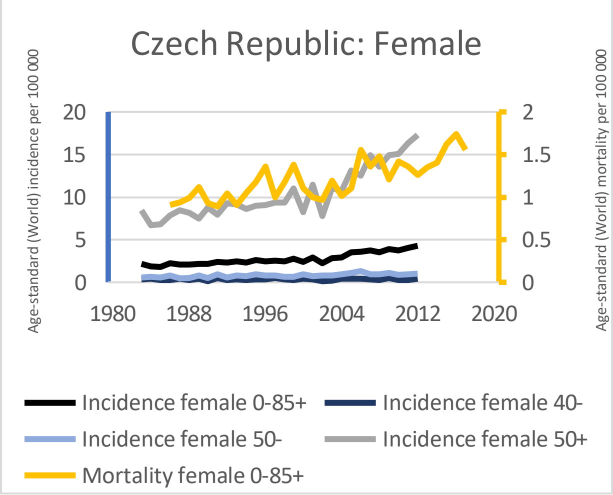

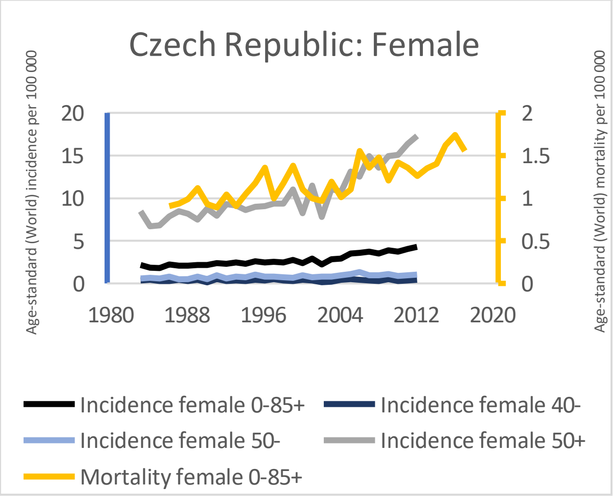

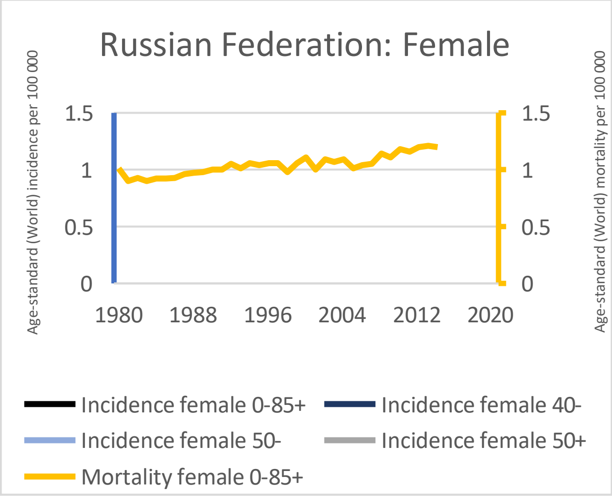

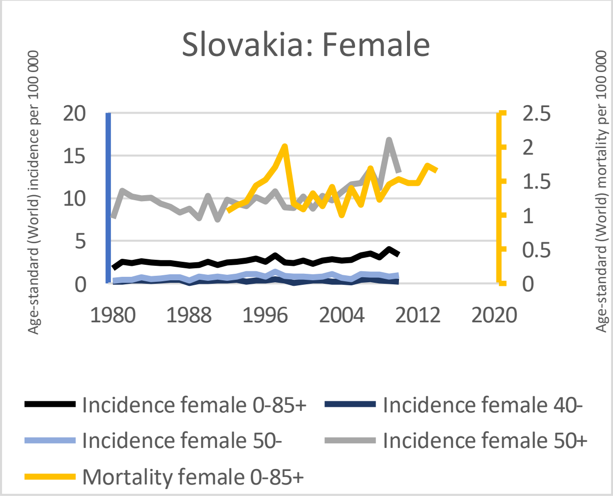

Africa		
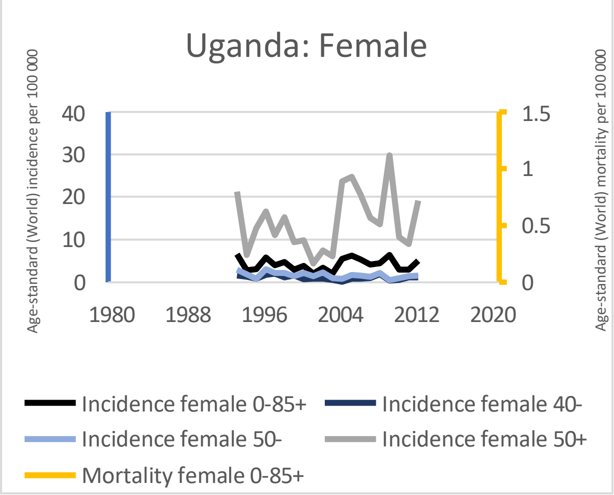Uganda: Female<br>Age-standard (World) incidence per 100 000<br>40<br>30<br>20<br>10<br>0<br>1980<br>1988<br>1996<br>2004<br>2012<br>2020<br>1.5<br>1<br>0.5<br>0<br>Age-standard (World) mortality per 100 000<br>Incidence female 0-85+<br>Incidence female 40-<br>Incidence female 50-<br>Incidence female 50+<br>Mortality female 0-85+		

Supplementary Figure 2: The graphs of the Joinpoint regression output

a.) Incidence male all ages

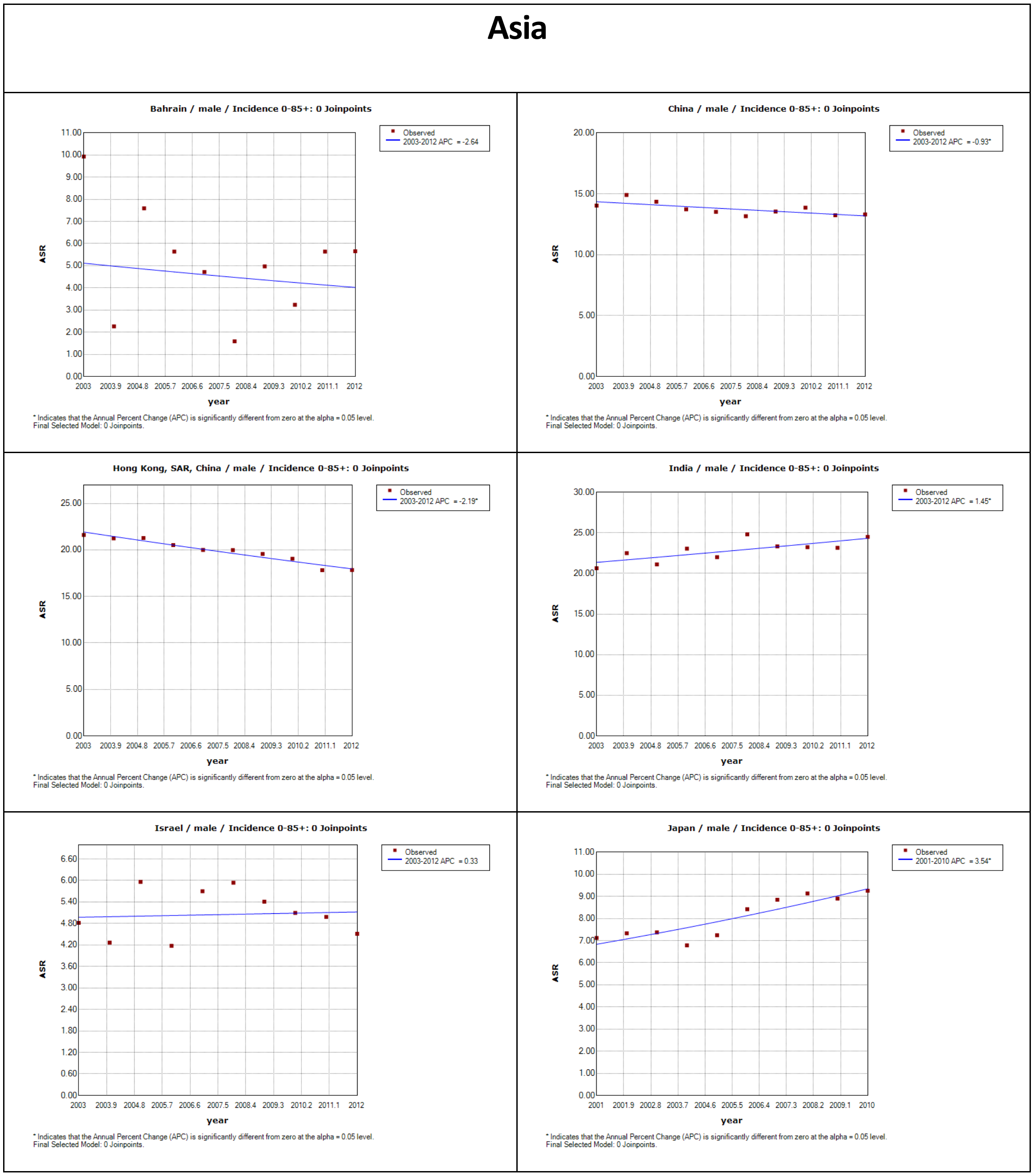

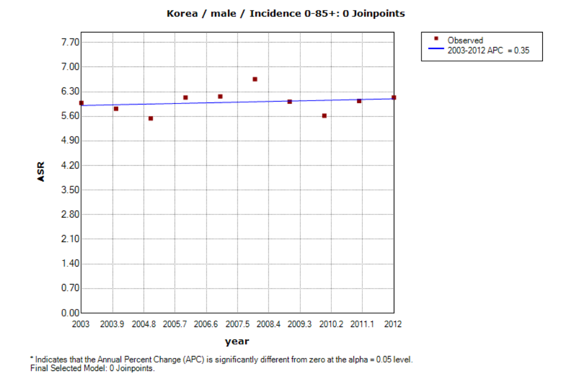
Korea / male / Incidence 0-85+: 0 Joinpoints
Observed
2003-2012 APC = 0.35
ASR
year
* Indicates that the Annual Percent Change (APC) is significantly different from zero at the alpha = 0.05 level.
Final Selected Model: 0 Joinpoints.

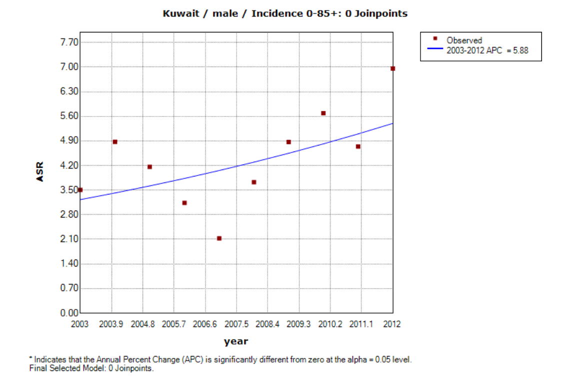
Kuwait / male / Incidence 0-85+: 0 Joinpoints
Observed
2003-2012 APC = 5.88
ASR
year
* Indicates that the Annual Percent Change (APC) is significantly different from zero at the alpha = 0.05 level.
Final Selected Model: 0 Joinpoints.

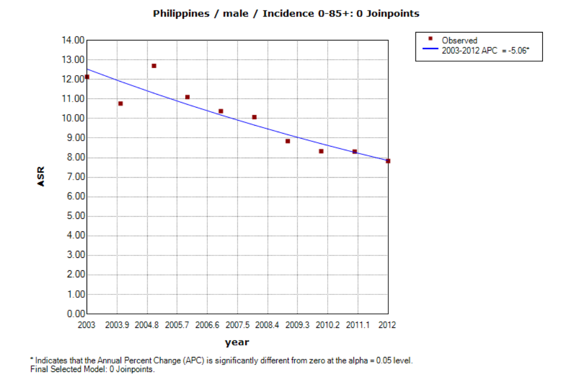
Philippines / male / Incidence 0-85+: 0 Joinpoints
Observed
2003-2012 APC = -5.06*
ASR
year
* Indicates that the Annual Percent Change (APC) is significantly different from zero at the alpha = 0.05 level.
Final Selected Model: 0 Joinpoints.

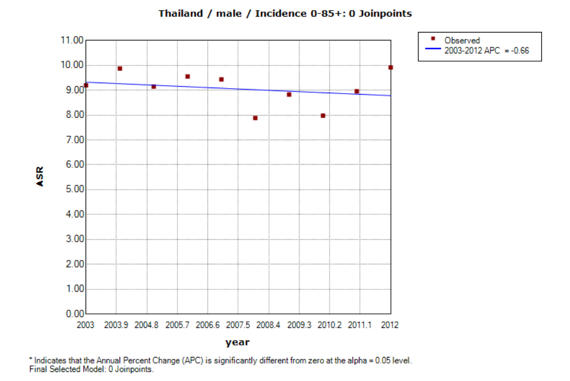
Thailand / male / Incidence 0-85+: 0 Joinpoints
Observed
2003-2012 APC = -0.66
ASR
year
* Indicates that the Annual Percent Change (APC) is significantly different from zero at the alpha = 0.05 level.
Final Selected Model: 0 Joinpoints.

Oceania

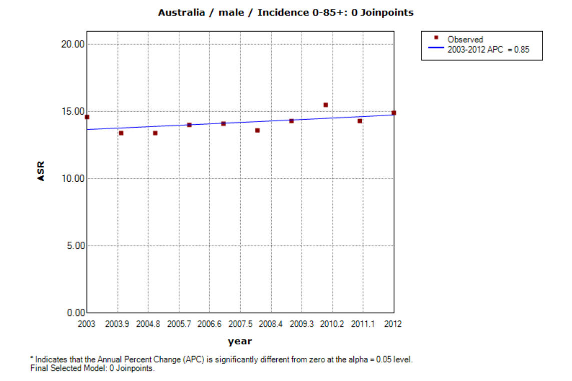
Australia / male / Incidence 0-85+: 0 Joinpoints
Observed
2003-2012 APC = 0.85
ASR
year
* Indicates that the Annual Percent Change (APC) is significantly different from zero at the alpha = 0.05 level.
Final Selected Model: 0 Joinpoints.

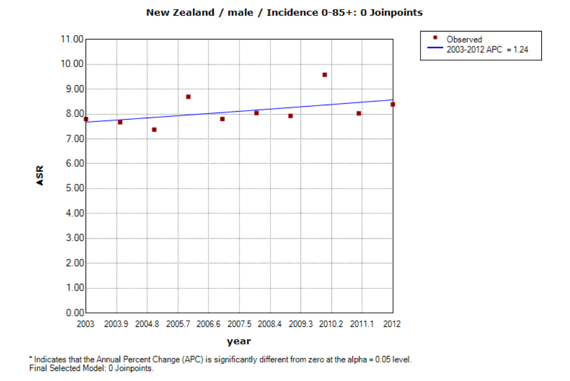
New Zealand / male / Incidence 0-85+: 0 Joinpoints
Observed
2003-2012 APC = 1.24
ASR
year
* Indicates that the Annual Percent Change (APC) is significantly different from zero at the alpha = 0.05 level.
Final Selected Model: 0 Joinpoints.

Northern America

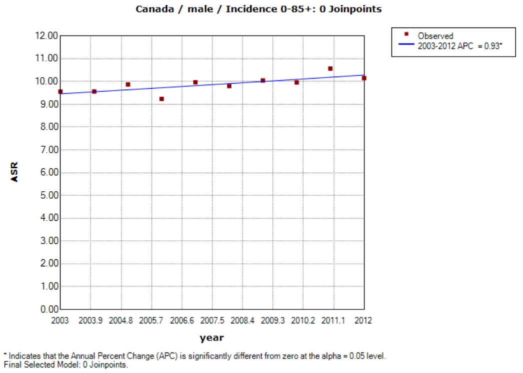

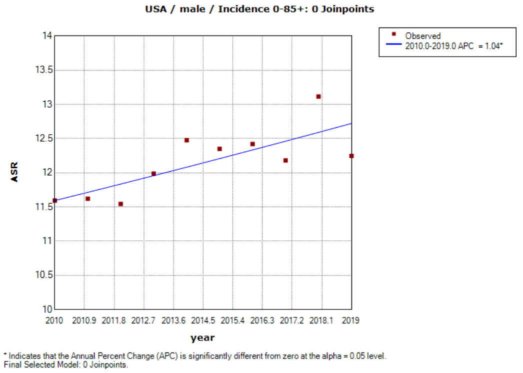

Southern America

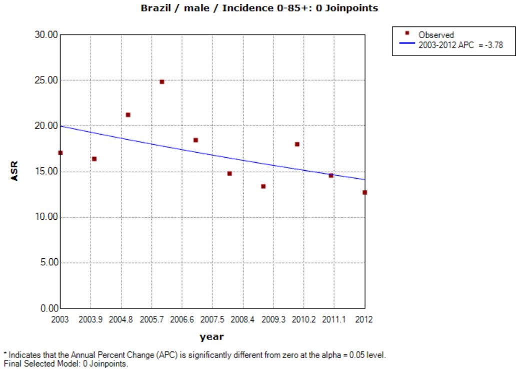

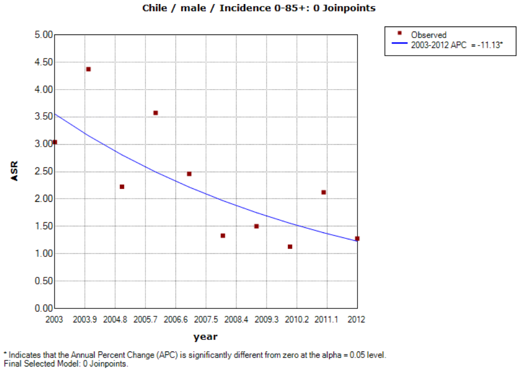

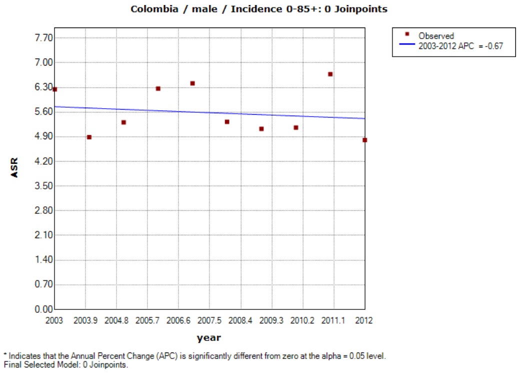

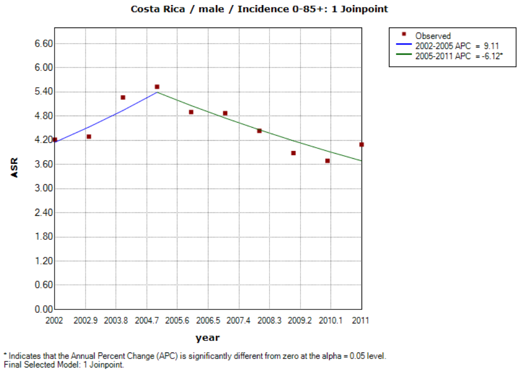

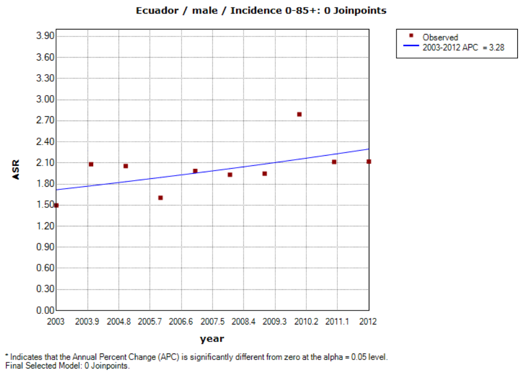

Northern Europe

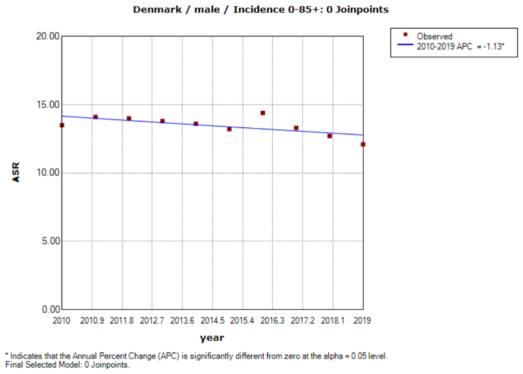

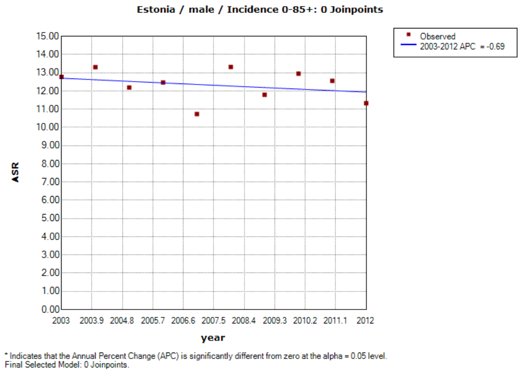

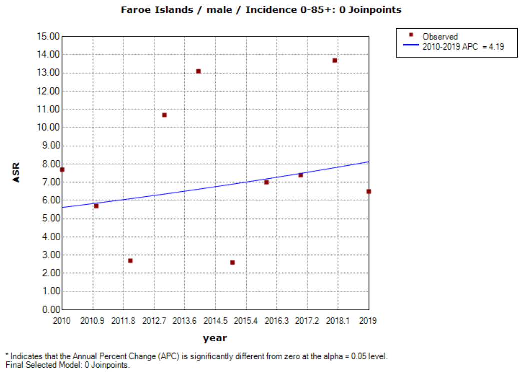

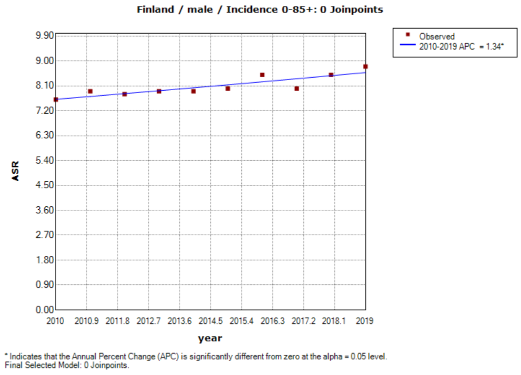

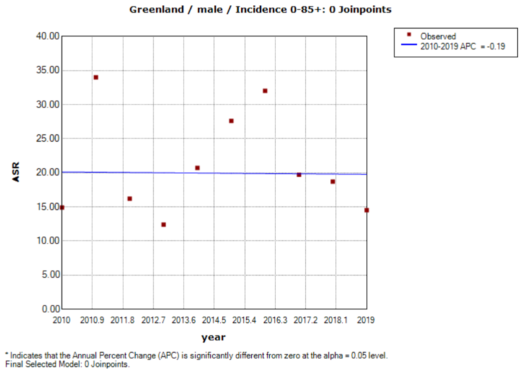

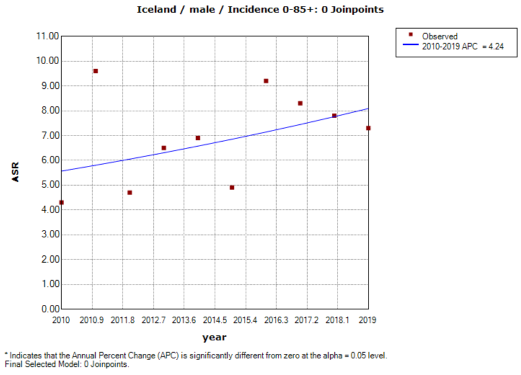

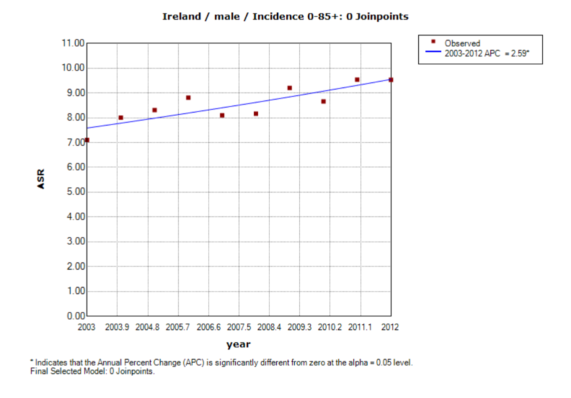

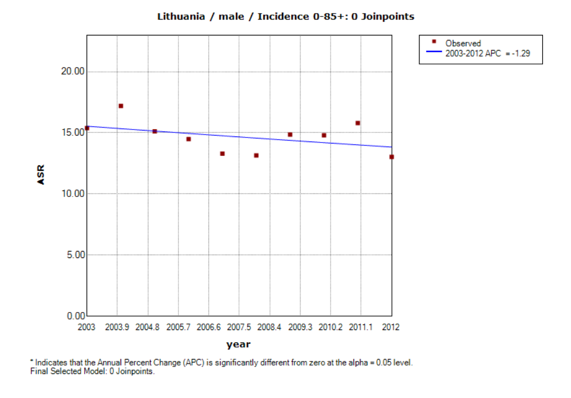

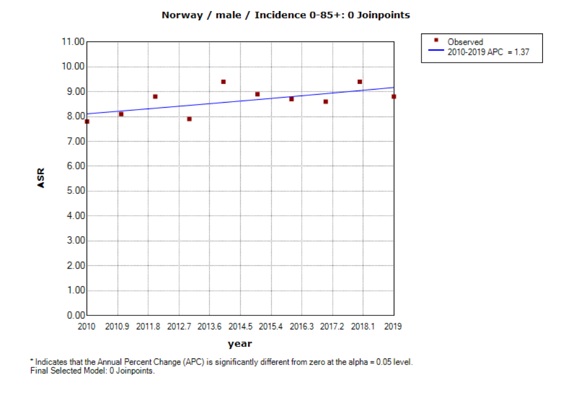

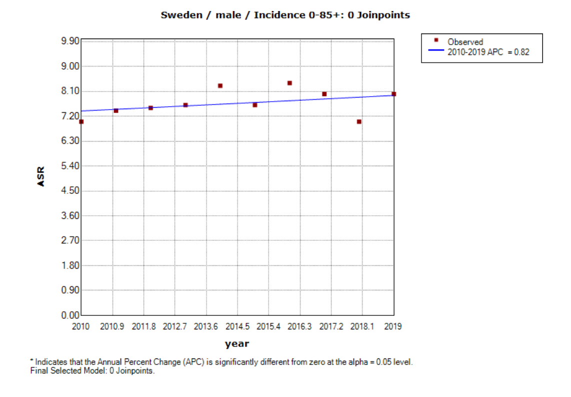

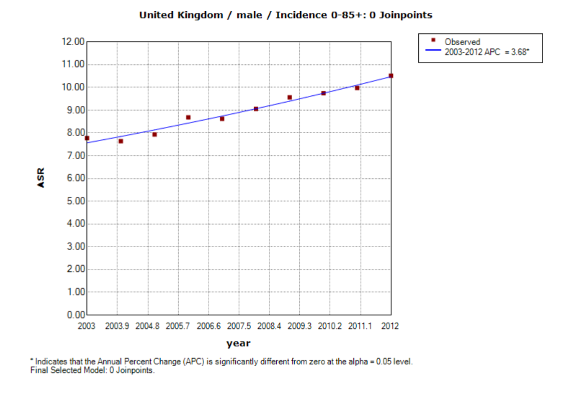

Western Europe

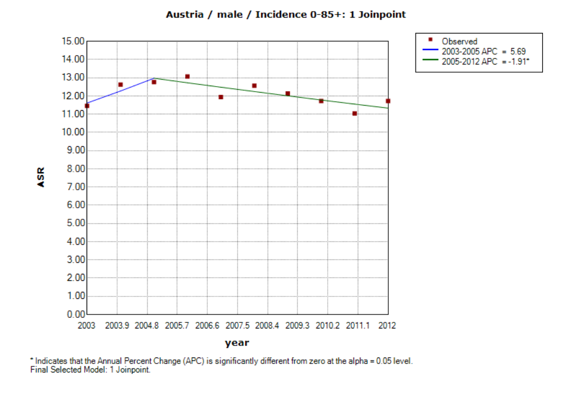

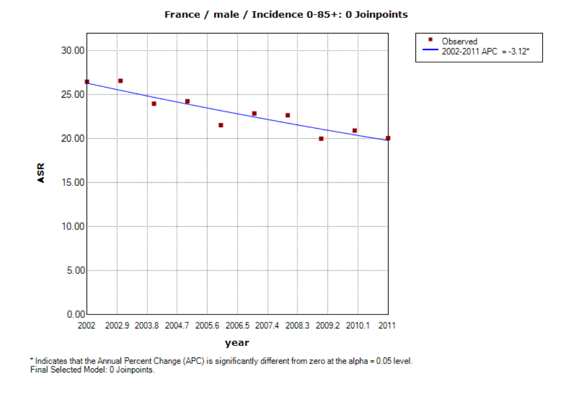

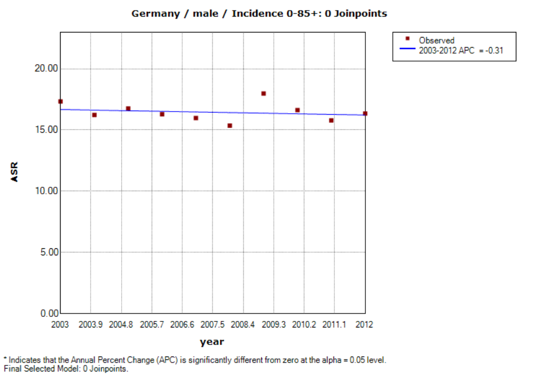

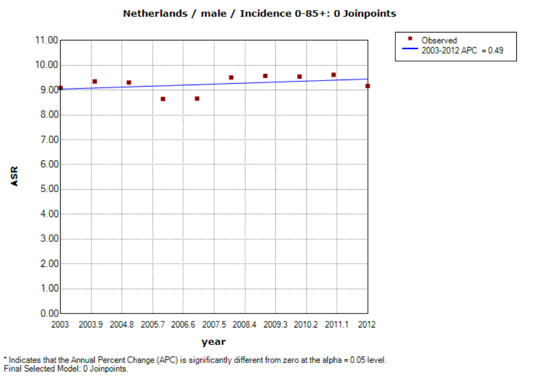

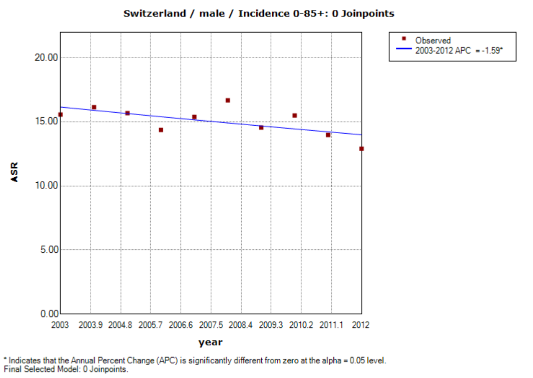

Southern Europe

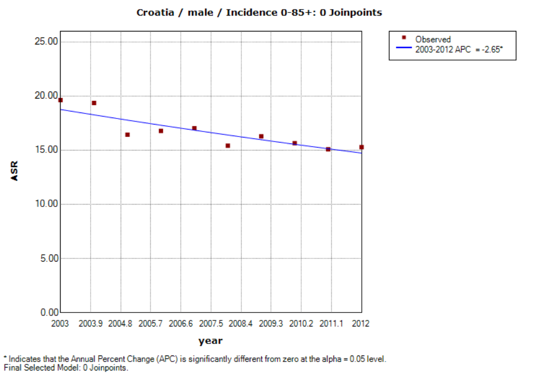

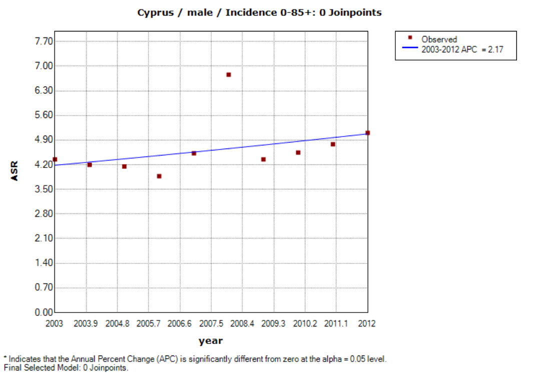

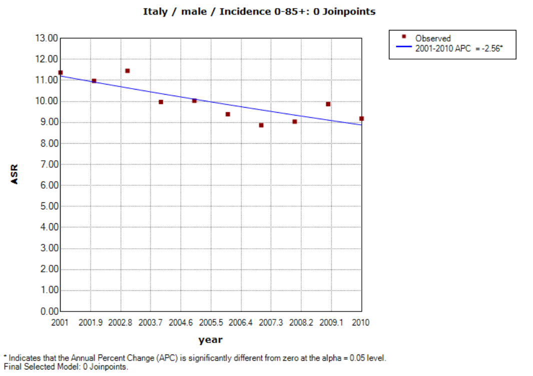

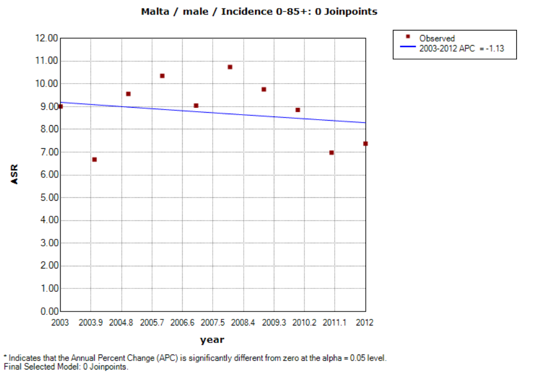

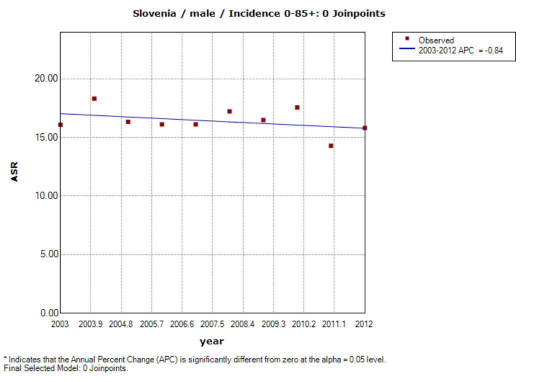

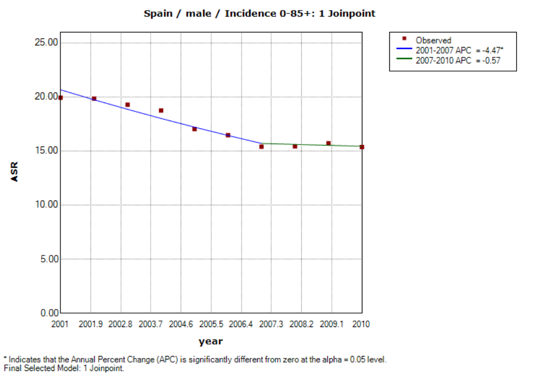

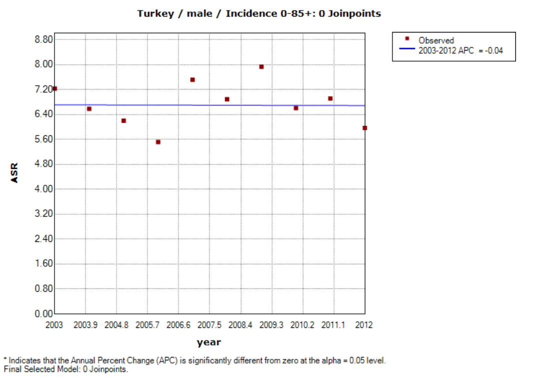

Eastern Europe

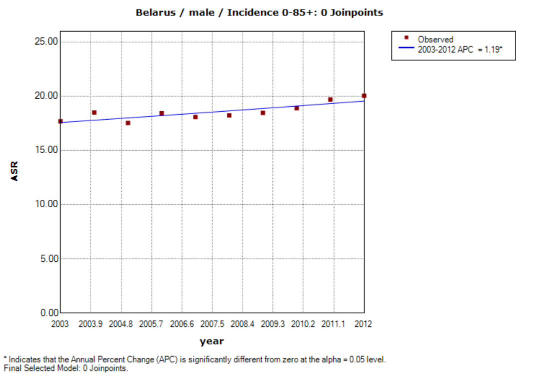

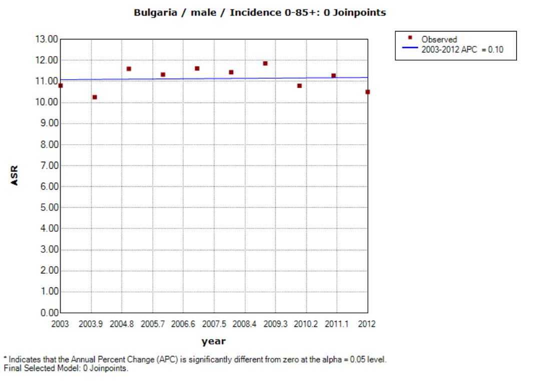

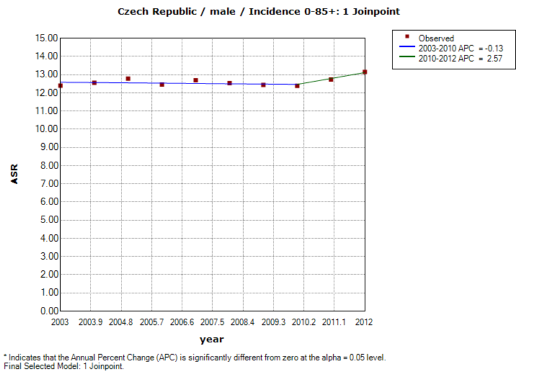

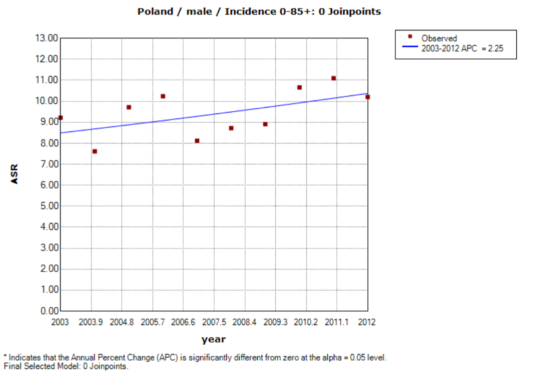

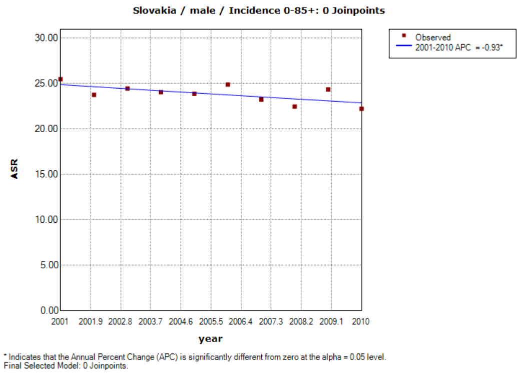
Slovakia / male / Incidence 0-85+: 0 Joinpoints
Observed
2001-2010 APC = -0.93*
ASR
30.00
25.00
20.00
15.00
10.00
5.00
0.00
2001
2001.9
2002.8
2003.7
2004.6
2005.5
2006.4
2007.3
2008.2
2009.1
2010
year
* Indicates that the Annual Percent Change (APC) is significantly different from zero at the alpha = 0.05 level.
Final Selected Model: 0 Joinpoints.

Africa

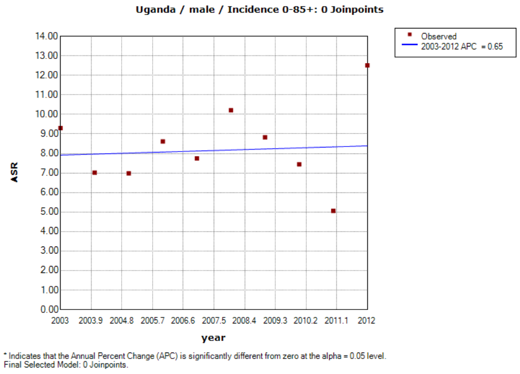
Uganda / male / Incidence 0-85+: 0 Joinpoints
Observed
2003-2012 APC = 0.65
ASR
14.00
13.00
12.00
11.00
10.00
9.00
8.00
7.00
6.00
5.00
4.00
3.00
2.00
1.00
0.00
2003
2003.9
2004.8
2005.7
2006.6
2007.5
2008.4
2009.3
2010.2
2011.1
2012
year
* Indicates that the Annual Percent Change (APC) is significantly different from zero at the alpha = 0.05 level.
Final Selected Model: 0 Joinpoints.

b.) Incidence female all ages

Asia

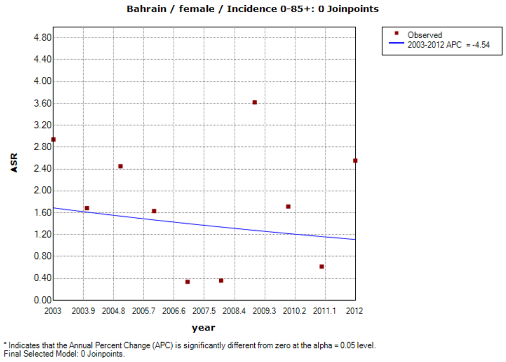

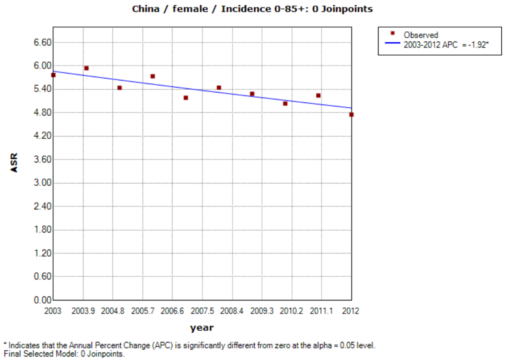

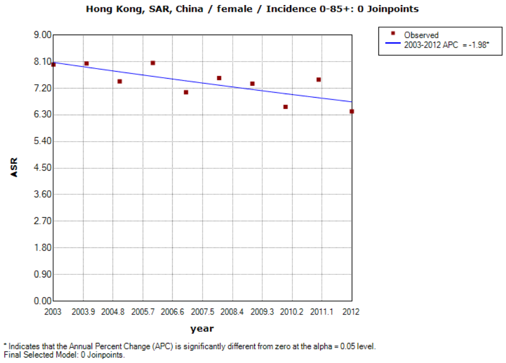

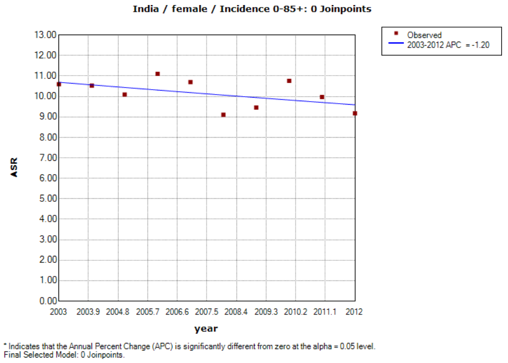

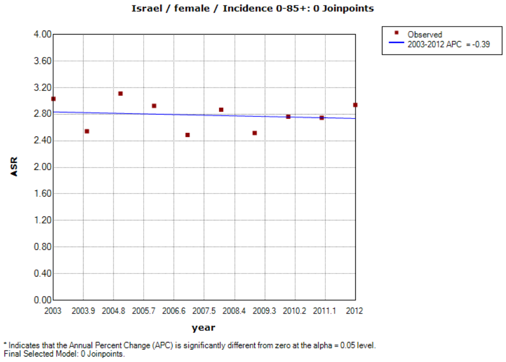

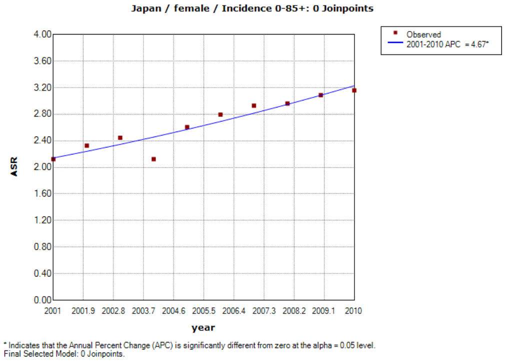

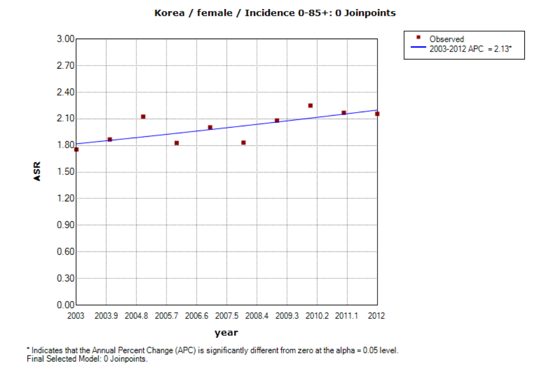
Korea / female / Incidence 0-85+: 0 Joinpoints
Observed
2003-2012 APC = 2.13*
ASR
year
* Indicates that the Annual Percent Change (APC) is significantly different from zero at the alpha = 0.05 level.
Final Selected Model: 0 Joinpoints.

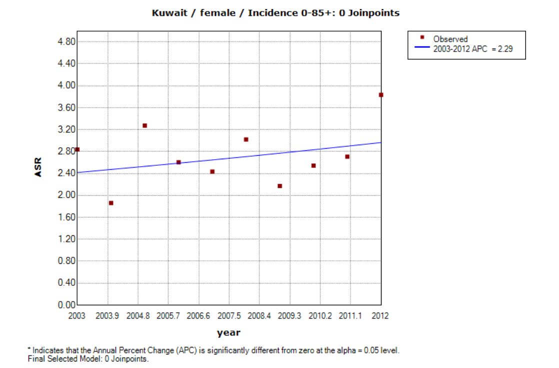
Kuwait / female / Incidence 0-85+: 0 Joinpoints
Observed
2003-2012 APC = 2.29
ASR
year
* Indicates that the Annual Percent Change (APC) is significantly different from zero at the alpha = 0.05 level.
Final Selected Model: 0 Joinpoints.

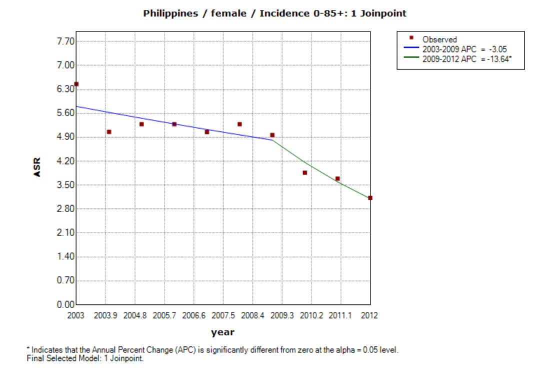
Philippines / female / Incidence 0-85+: 1 Joinpoint
Observed
2003-2009 APC = -3.05
2009-2012 APC = -13.64*
ASR
year
* Indicates that the Annual Percent Change (APC) is significantly different from zero at the alpha = 0.05 level.
Final Selected Model: 1 Joinpoint.

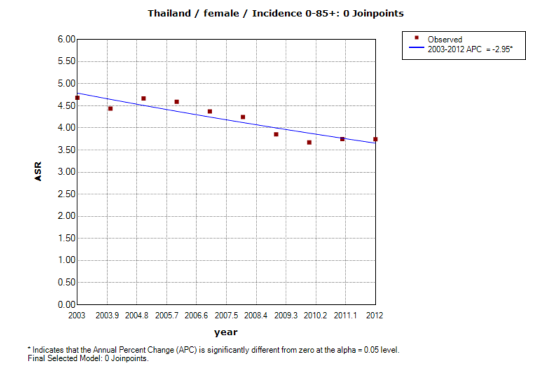
Thailand / female / Incidence 0-85+: 0 Joinpoints
Observed
2003-2012 APC = -2.95*
ASR
year
* Indicates that the Annual Percent Change (APC) is significantly different from zero at the alpha = 0.05 level.
Final Selected Model: 0 Joinpoints.

Oceania

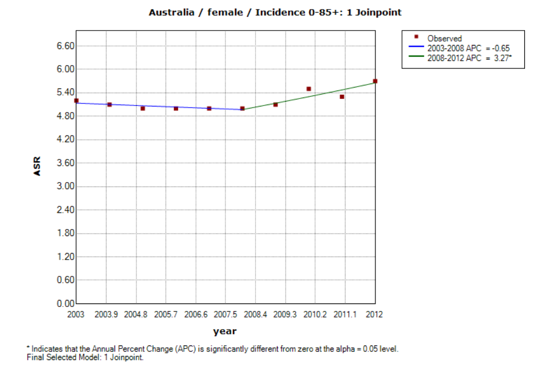
Australia / female / Incidence 0-85+: 1 Joinpoint
Observed
2003-2008 APC = -0.65
2008-2012 APC = 3.27*
ASR
year
* Indicates that the Annual Percent Change (APC) is significantly different from zero at the alpha = 0.05 level.
Final Selected Model: 1 Joinpoint.

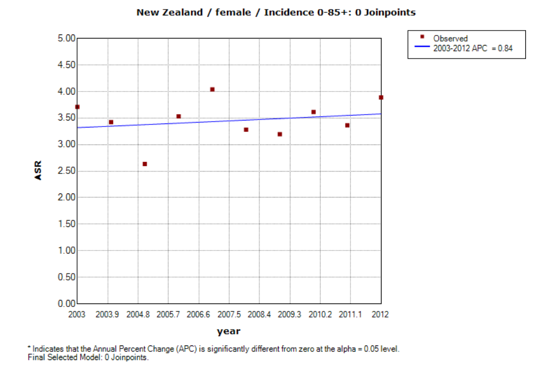
New Zealand / female / Incidence 0-85+: 0 Joinpoints
Observed
2003-2012 APC = 0.84
ASR
year
* Indicates that the Annual Percent Change (APC) is significantly different from zero at the alpha = 0.05 level.
Final Selected Model: 0 Joinpoints.

Northern America

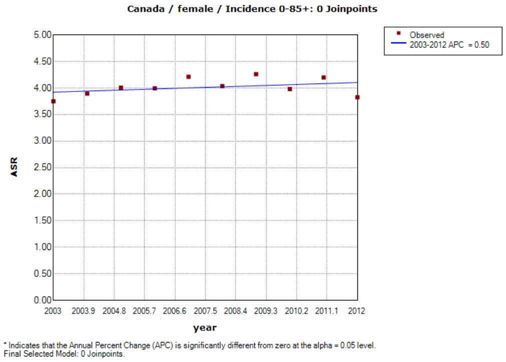

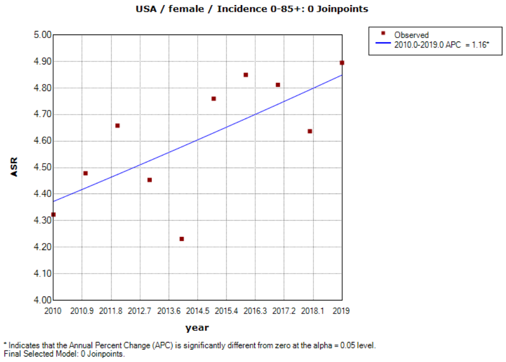

Southern America

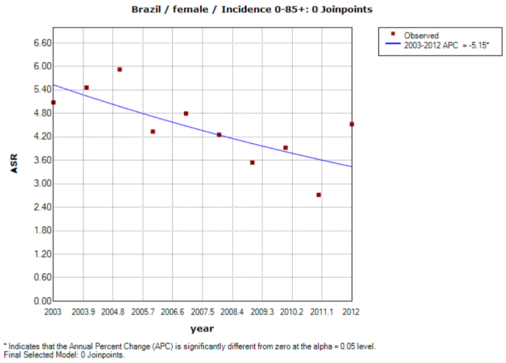

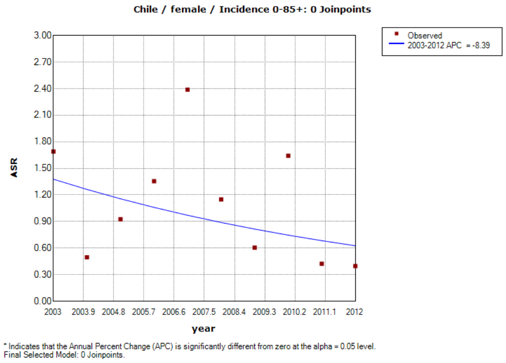

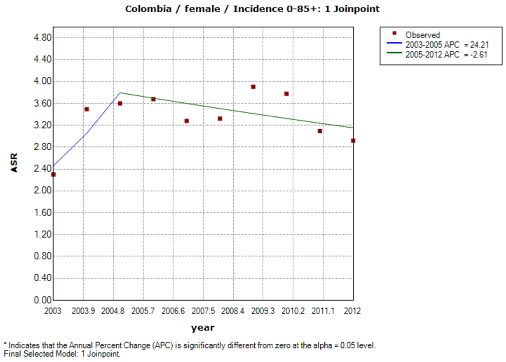

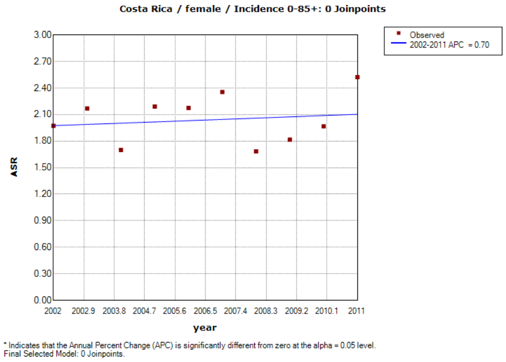

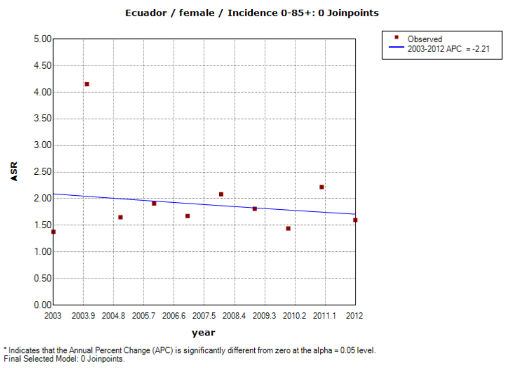

Northern Europe

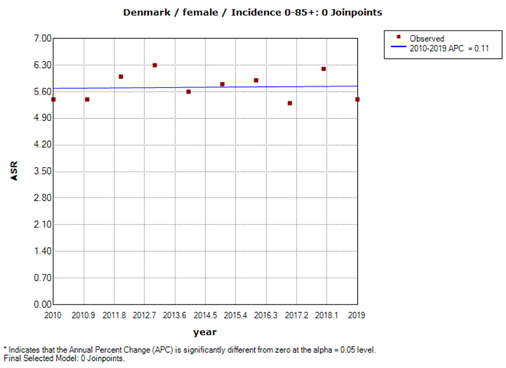

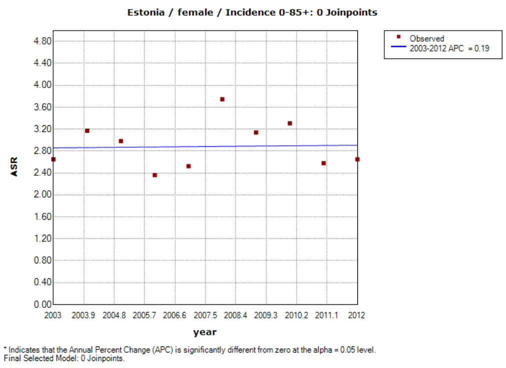

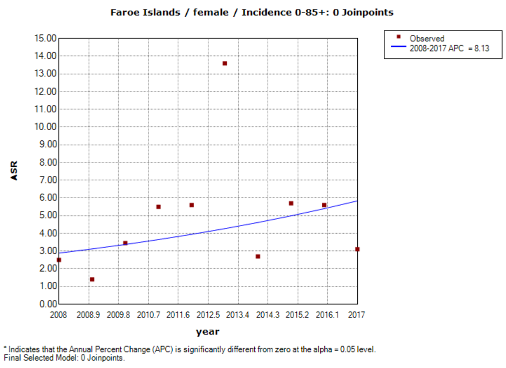

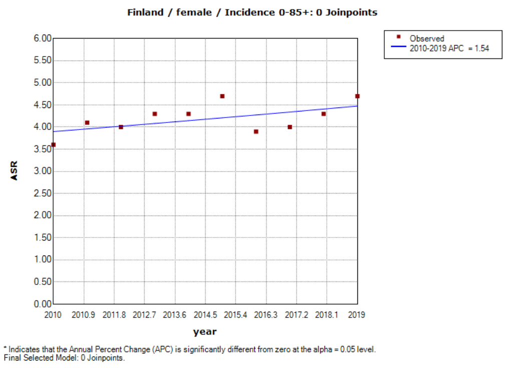

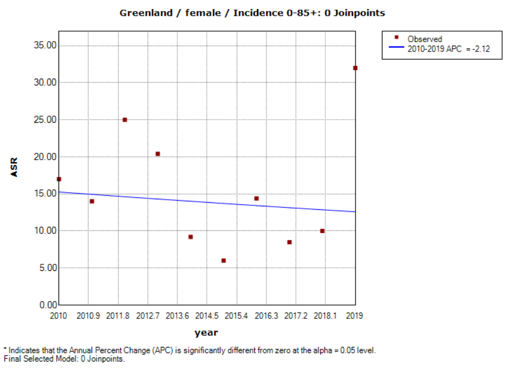

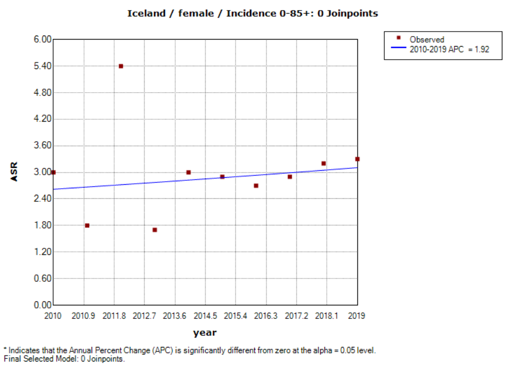

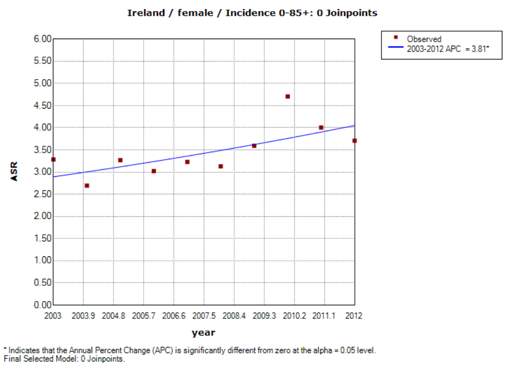

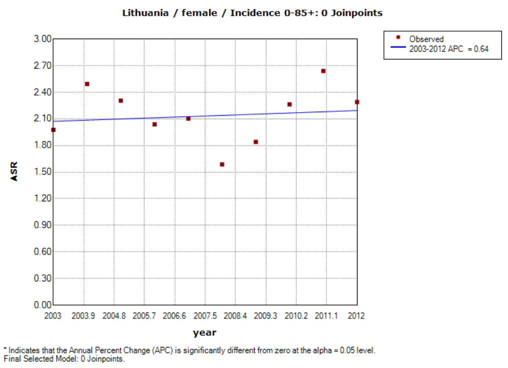

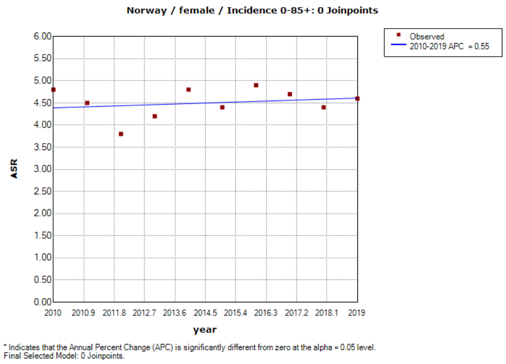

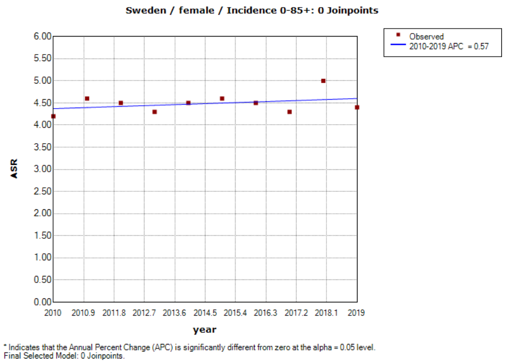

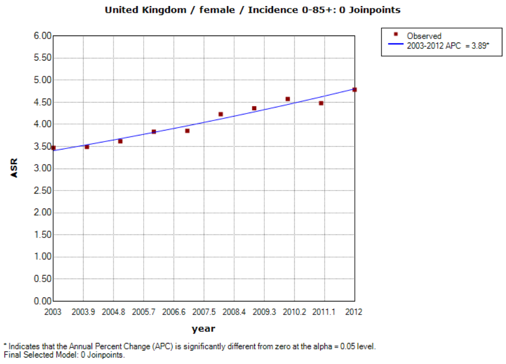

Western Europe

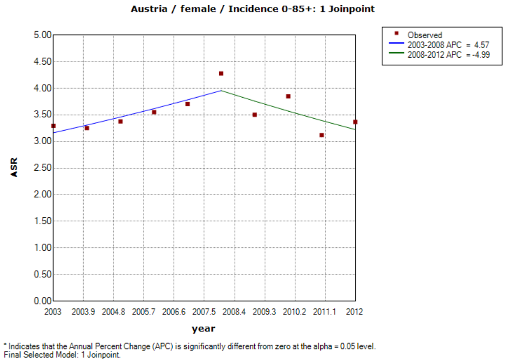

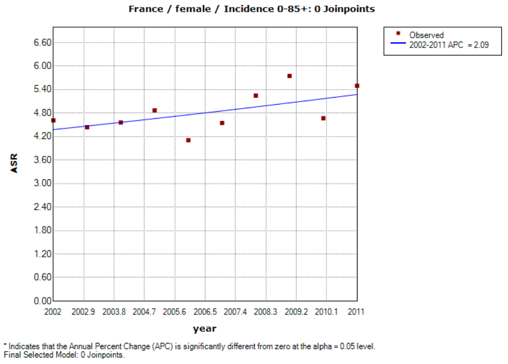

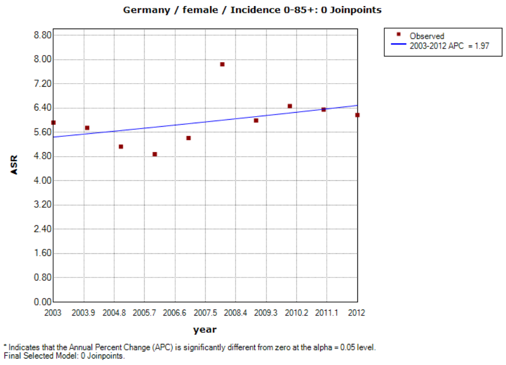

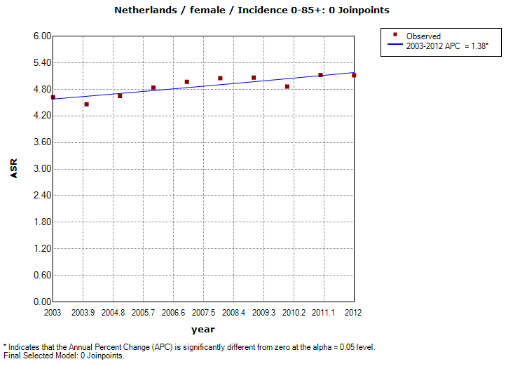

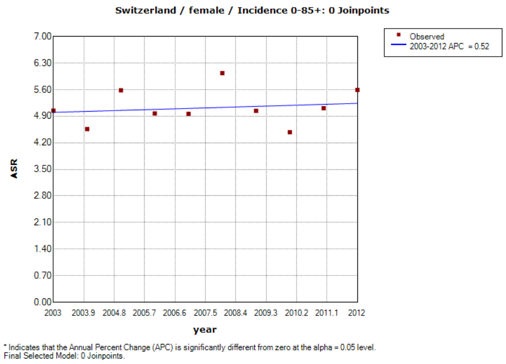

Southern Europe

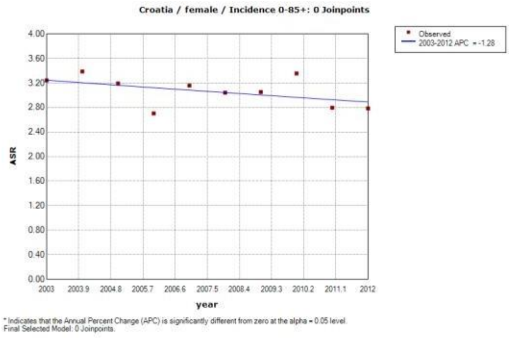

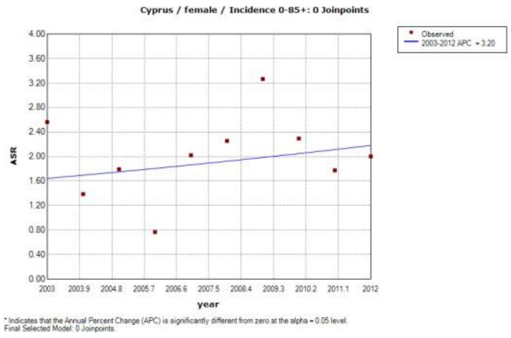

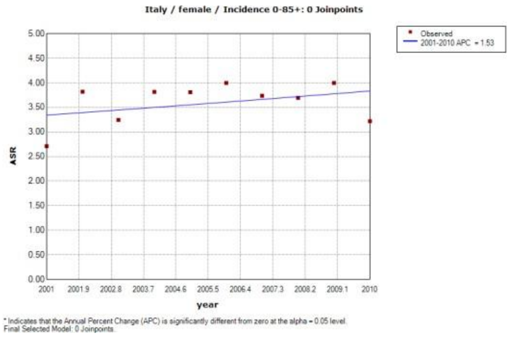

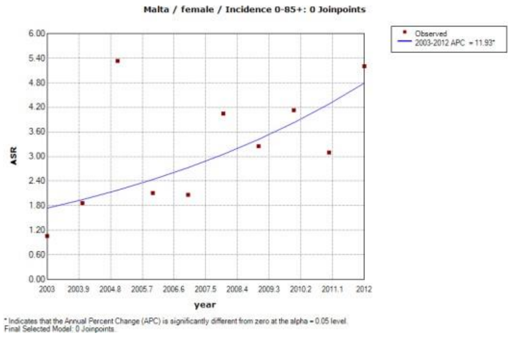

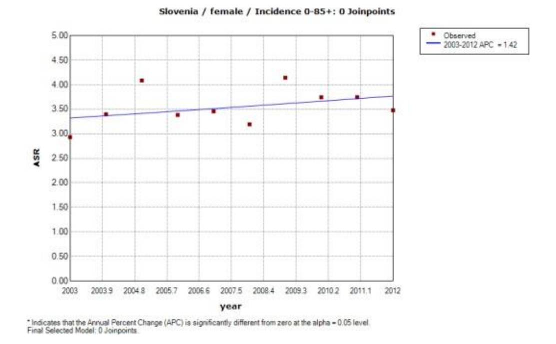

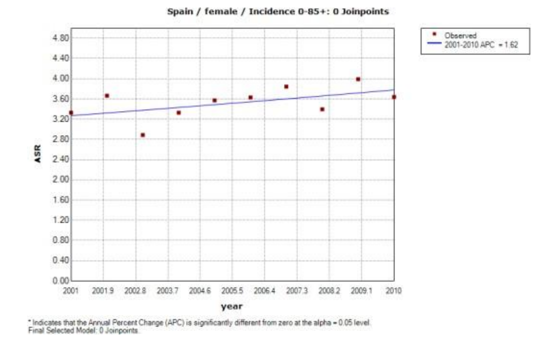

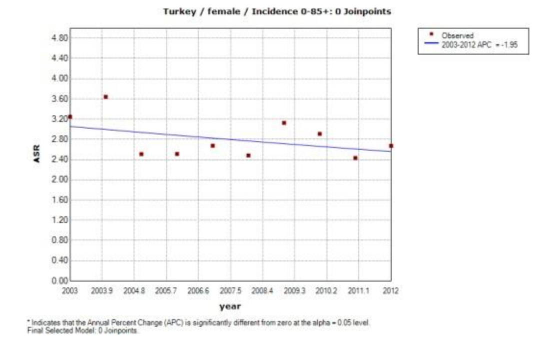

Eastern Europe

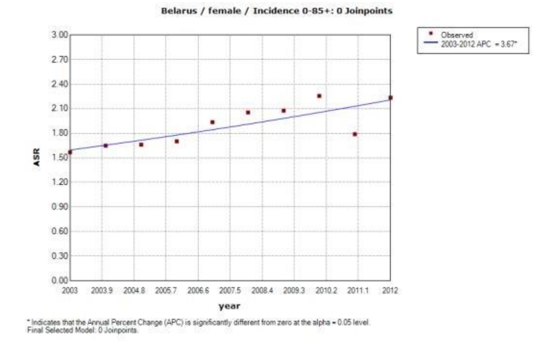

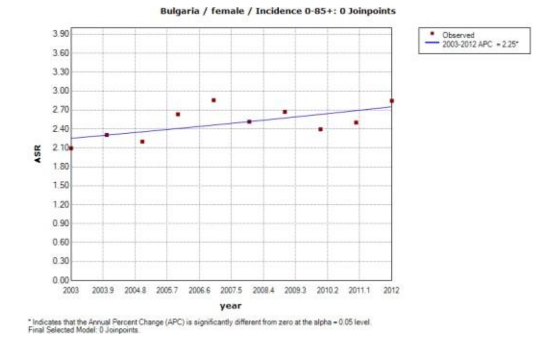

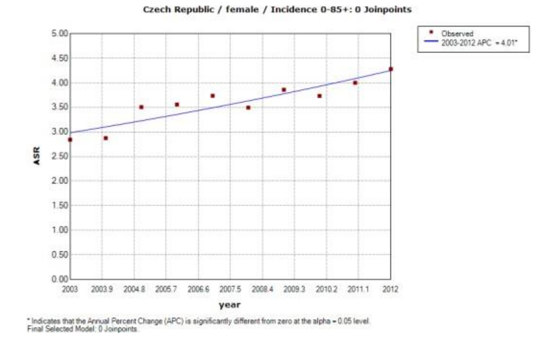

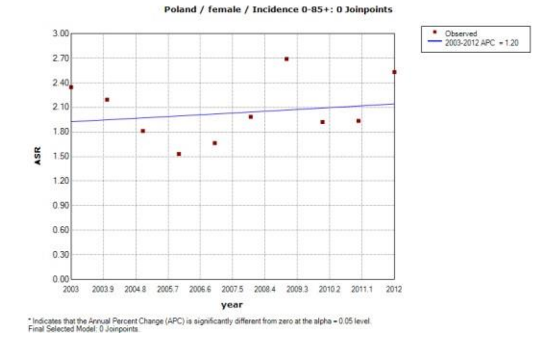

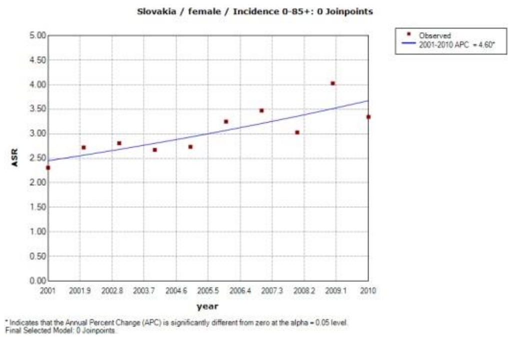
Slovakia / female / Incidence 0-85+: 0 Joinpoints
Observed
2001-2010 APC = 4.60*
ASR
year
* Indicates that the Annual Percent Change (APC) is significantly different from zero at the alpha = 0.05 level.
Final Selected Model: 0 Joinpoints.

Africa

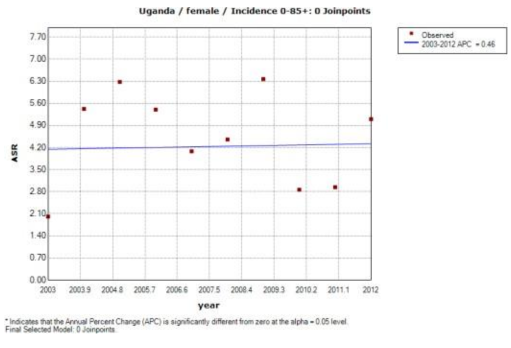
Uganda / female / Incidence 0-85+: 0 Joinpoints
Observed
2003-2012 APC = 0.46
ASR
year
* Indicates that the Annual Percent Change (APC) is significantly different from zero at the alpha = 0.05 level.
Final Selected Model: 0 Joinpoints.

c.) Incidence male below 40 years old

Asia

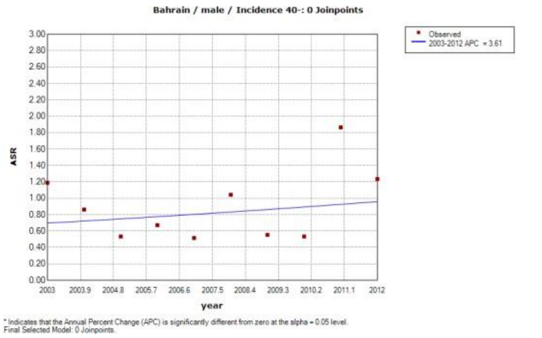

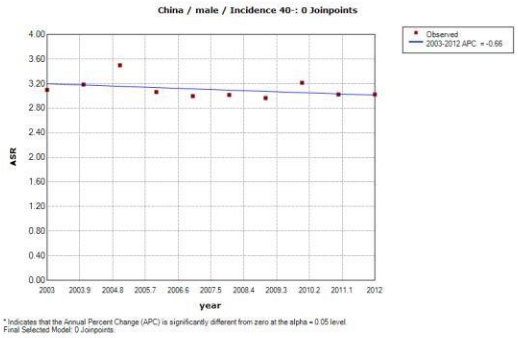

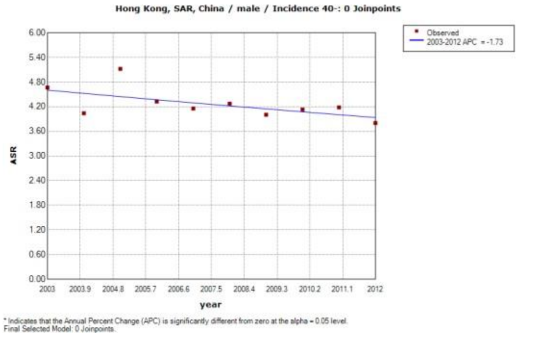

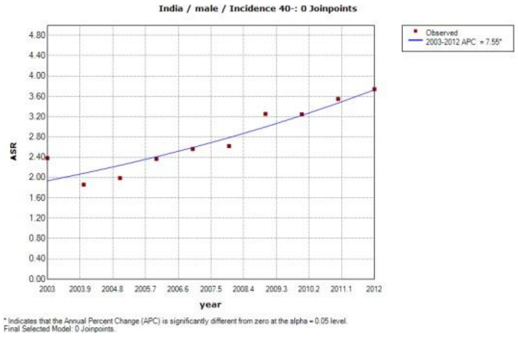

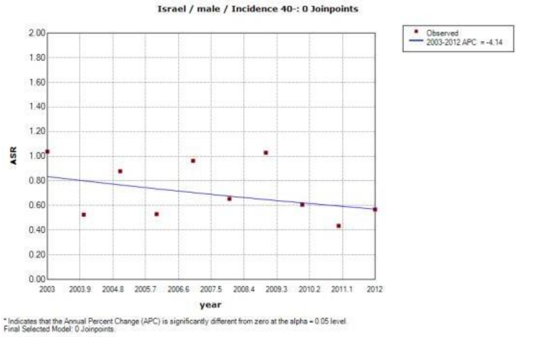

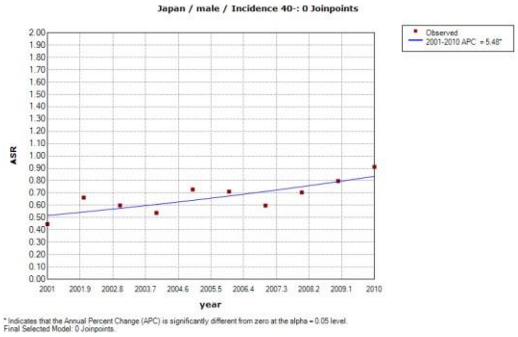

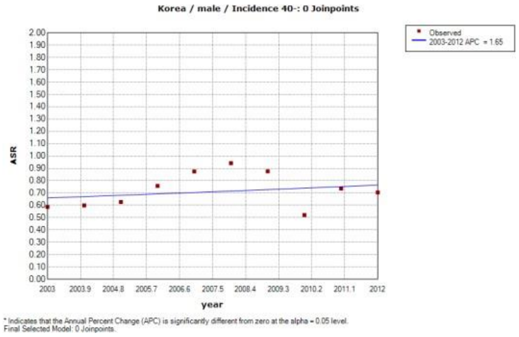
Korea / male / Incidence 40-: 0 Joinpoints
Observed
2003-2012 APC = 1.65
ASR
year
* Indicates that the Annual Percent Change (APC) is significantly different from zero at the alpha = 0.05 level.
Final Selected Model: 0 Joinpoints.

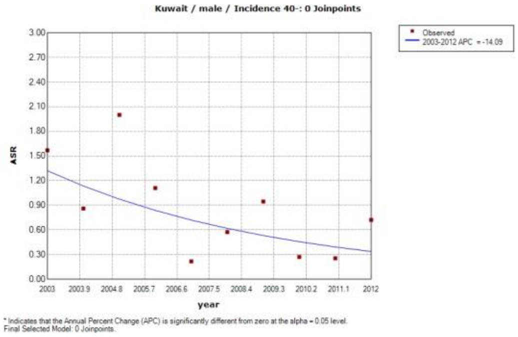
Kuwait / male / Incidence 40-: 0 Joinpoints
Observed
2003-2012 APC = -14.09
ASR
year
* Indicates that the Annual Percent Change (APC) is significantly different from zero at the alpha = 0.05 level.
Final Selected Model: 0 Joinpoints.

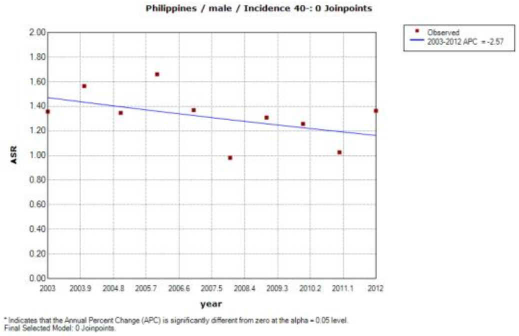
Philippines / male / Incidence 40-: 0 Joinpoints
Observed
2003-2012 APC = -2.57
ASR
year
* Indicates that the Annual Percent Change (APC) is significantly different from zero at the alpha = 0.05 level.
Final Selected Model: 0 Joinpoints.

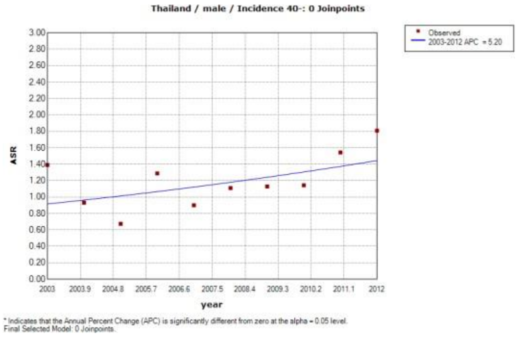
Thailand / male / Incidence 40-: 0 Joinpoints
Observed
2003-2012 APC = 5.20
ASR
year
* Indicates that the Annual Percent Change (APC) is significantly different from zero at the alpha = 0.05 level.
Final Selected Model: 0 Joinpoints.

Oceania

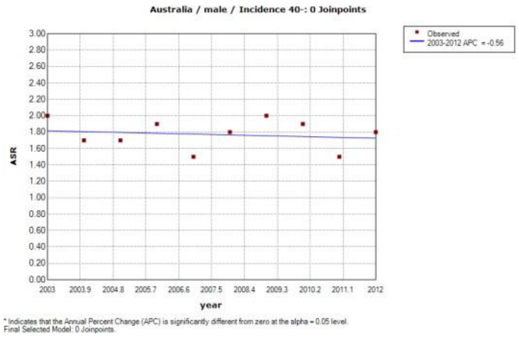
Australia / male / Incidence 40-: 0 Joinpoints
Observed
2003-2012 APC = -0.56
ASR
year
* Indicates that the Annual Percent Change (APC) is significantly different from zero at the alpha = 0.05 level.
Final Selected Model: 0 Joinpoints.

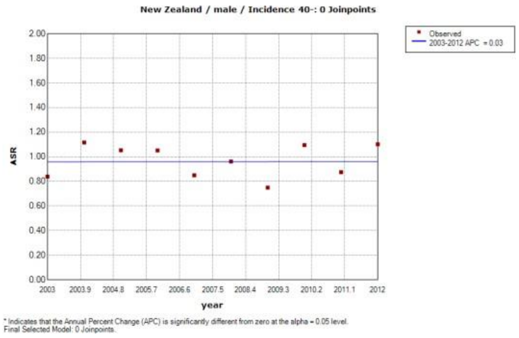
New Zealand / male / Incidence 40-: 0 Joinpoints
Observed
2003-2012 APC = 0.03
ASR
year
* Indicates that the Annual Percent Change (APC) is significantly different from zero at the alpha = 0.05 level.
Final Selected Model: 0 Joinpoints.

Northern America

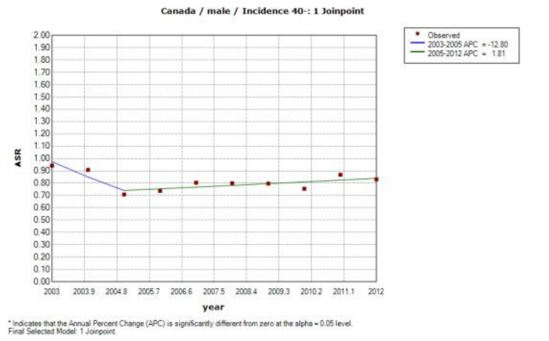

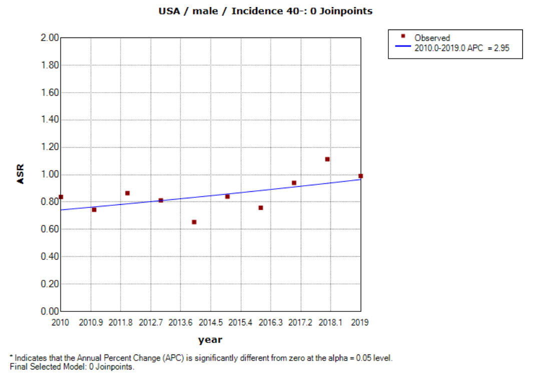

Southern America

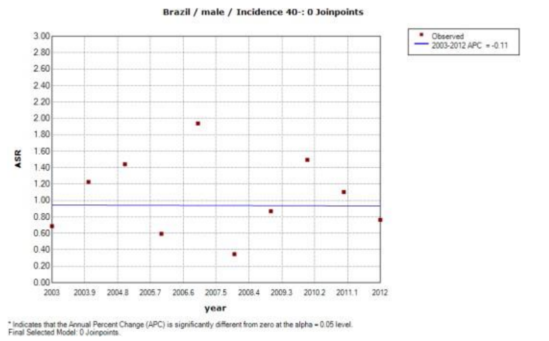

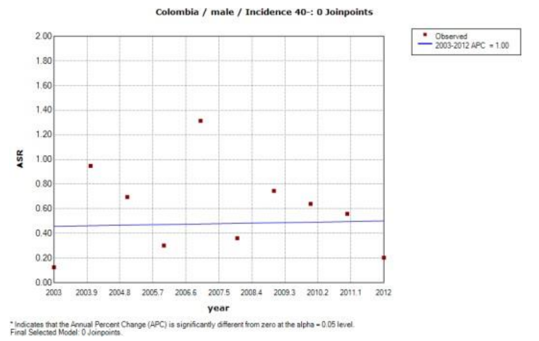

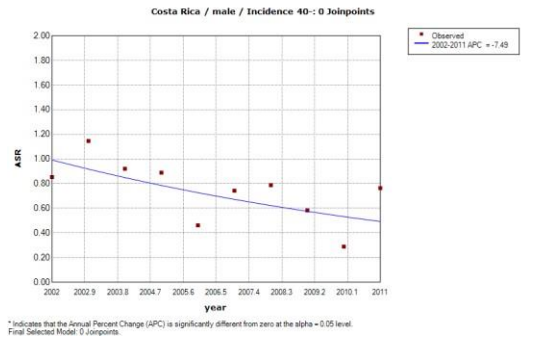

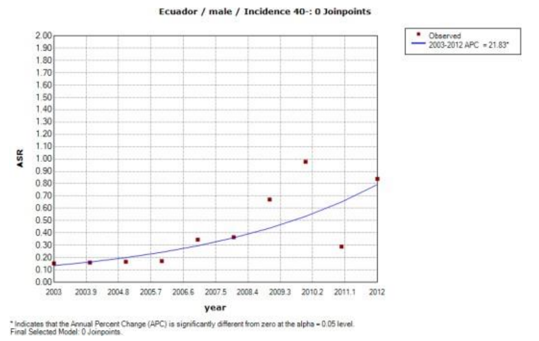

Northern Europe

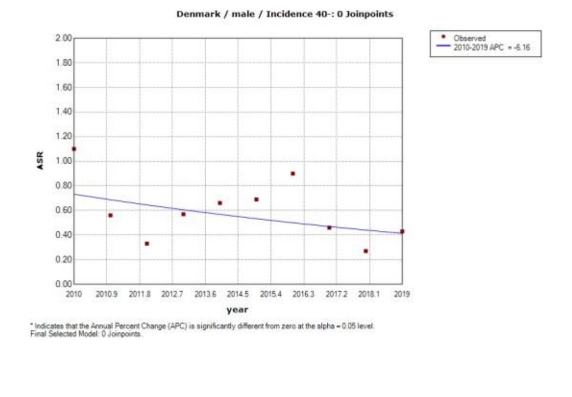

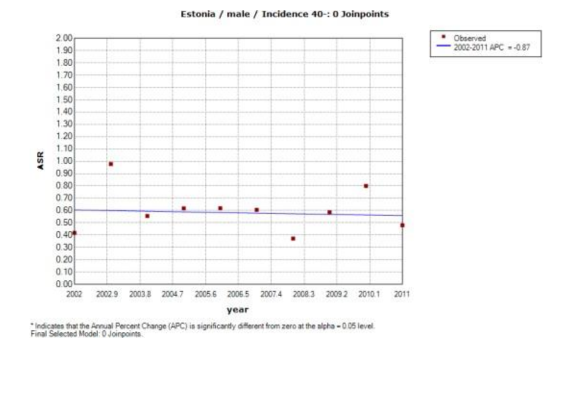

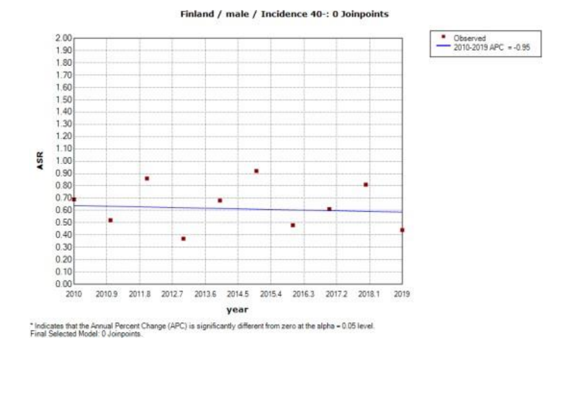

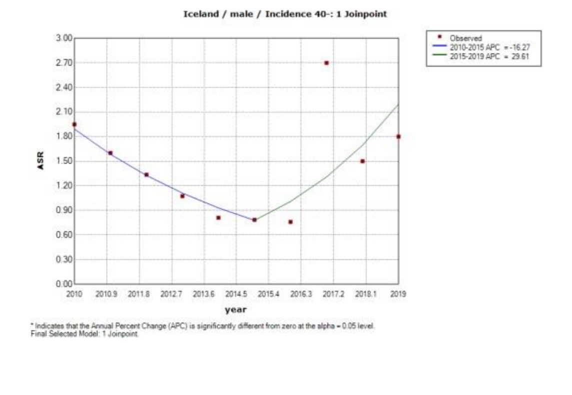

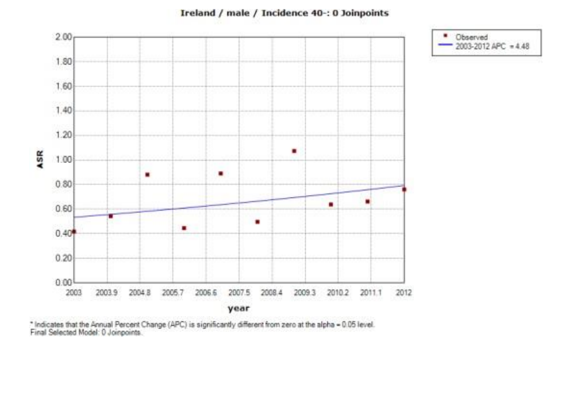

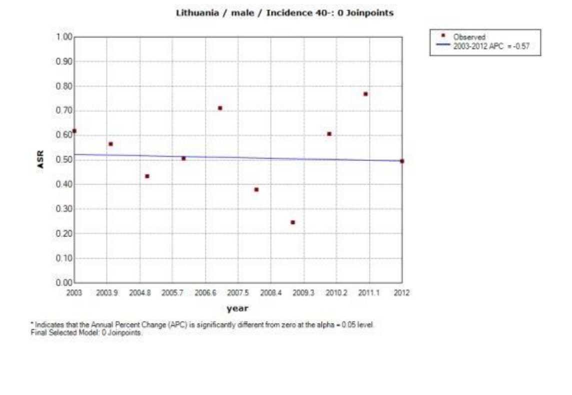

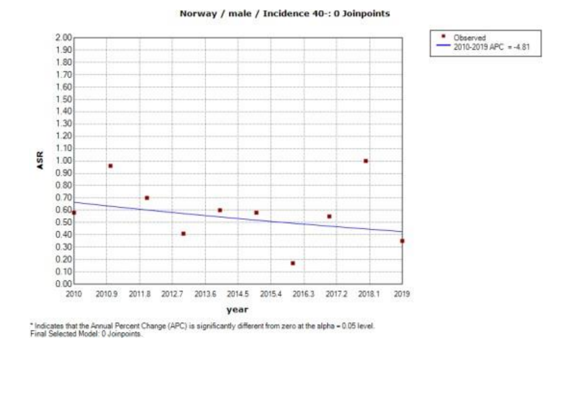

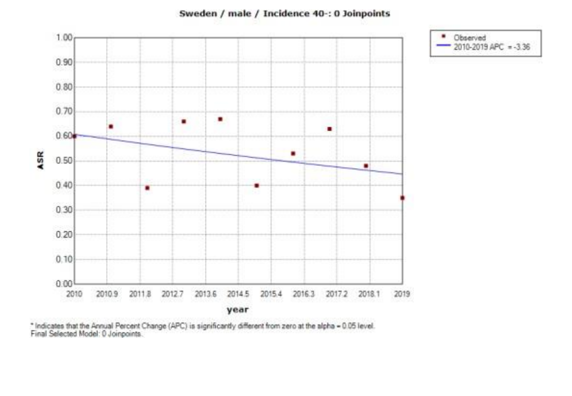

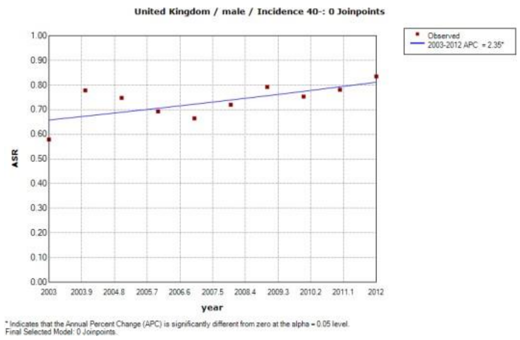

Western Europe

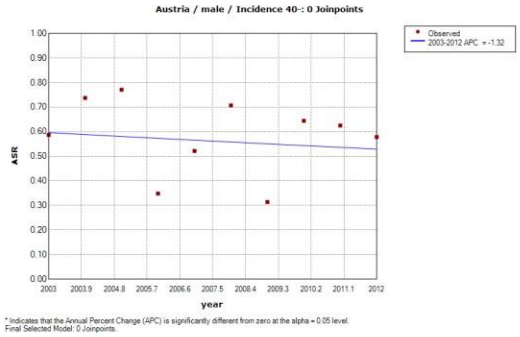

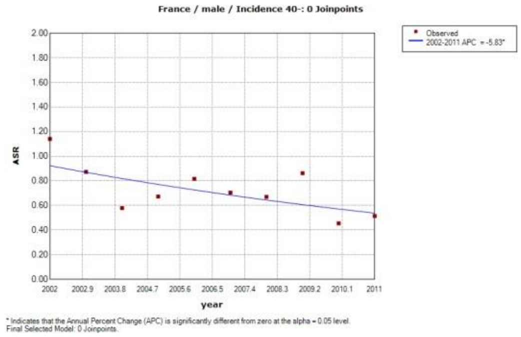

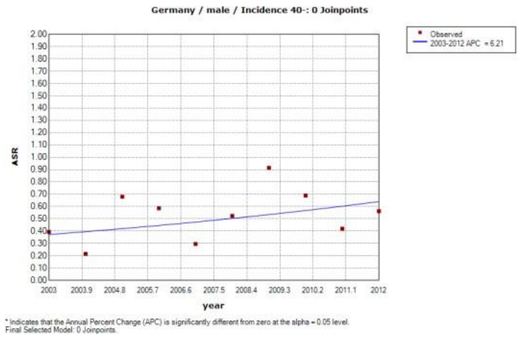

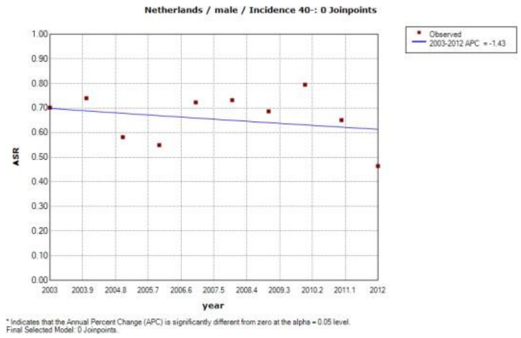

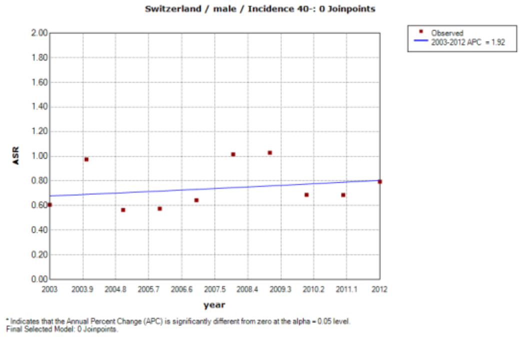

Southern Europe

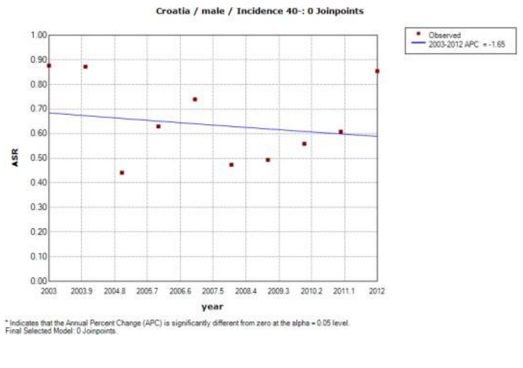

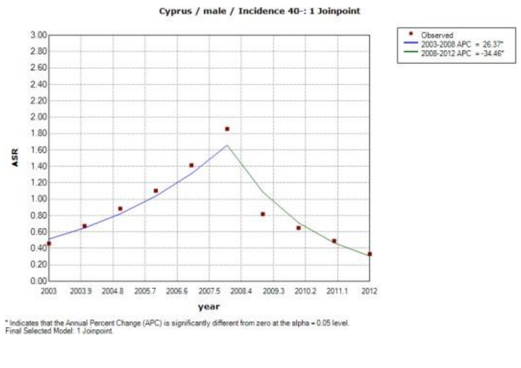

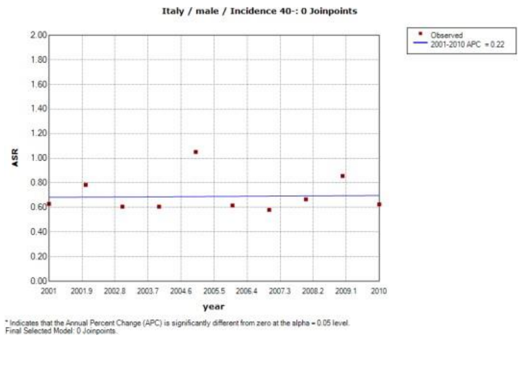

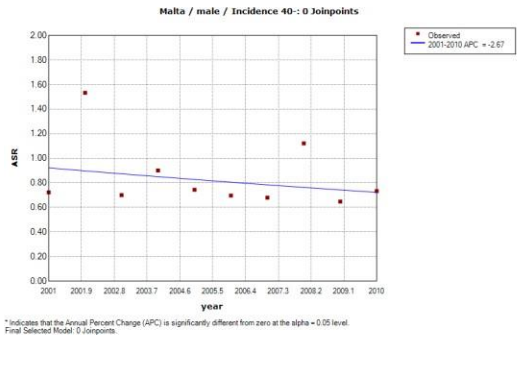

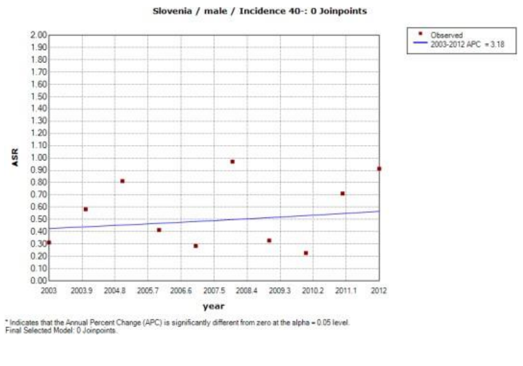

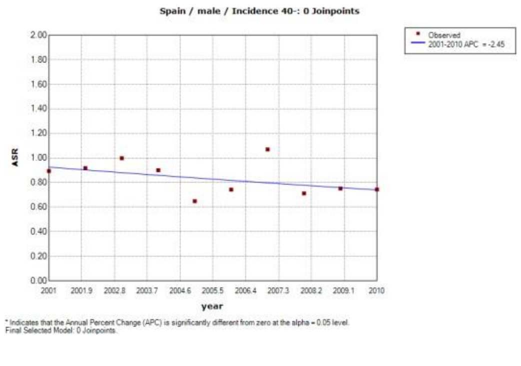

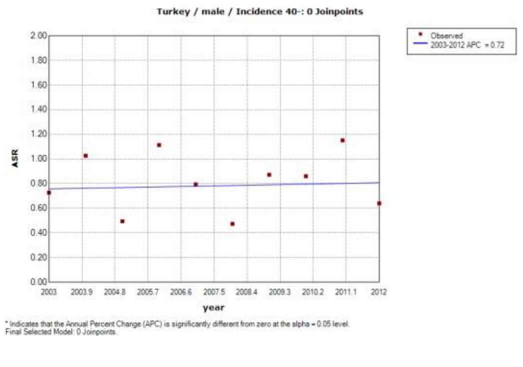

Eastern Europe

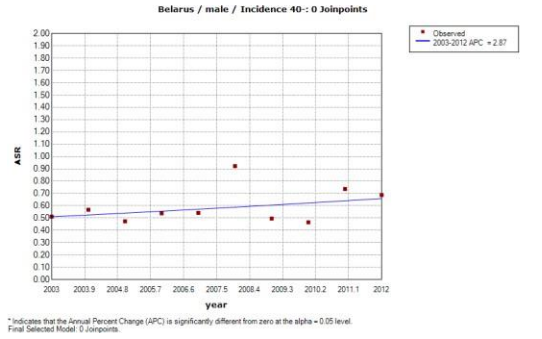

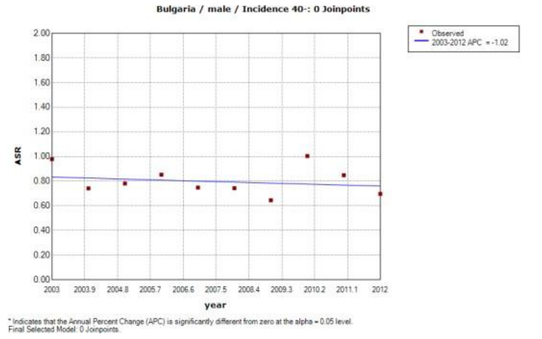

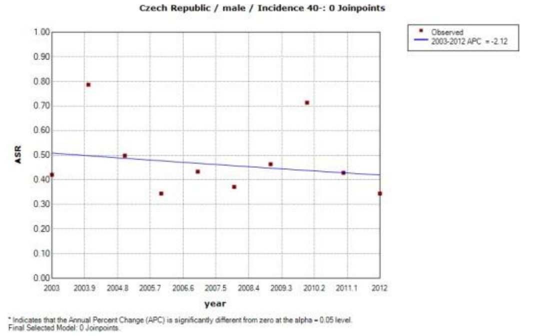

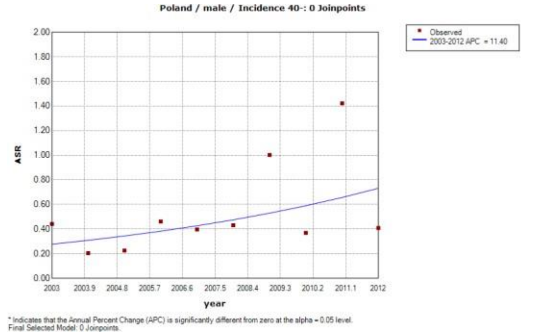

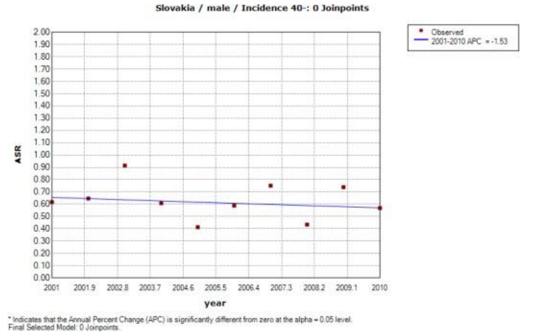

Africa	
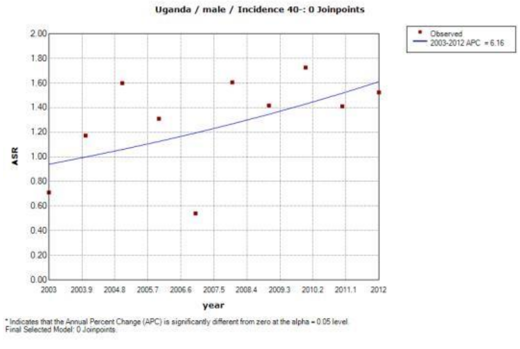Uganda / male / Incidence 40-: 0 Joinpoints<br>Observed<br>2003-2012 APC = 6.16<br>ASR<br>2.00<br>1.80<br>1.60<br>1.40<br>1.20<br>1.00<br>0.80<br>0.60<br>0.40<br>0.20<br>0.00<br>2003<br>2003.9<br>2004.8<br>2005.7<br>2006.6<br>2007.5<br>2008.4<br>2009.3<br>2010.2<br>2011.1<br>2012<br>year<br>* Indicates that the Annual Percent Change (APC) is significantly different from zero at the alpha = 0.05 level.<br>Final Selected Model: 0 Joinpoints.	

d.) Incidence female below 40 years old

Asia

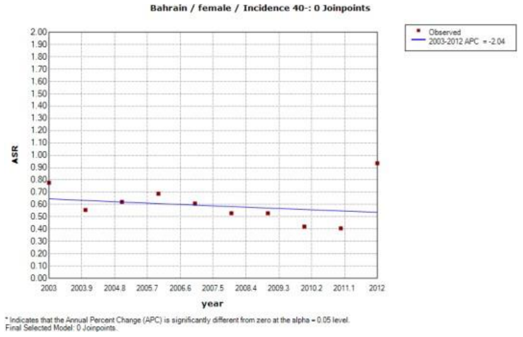

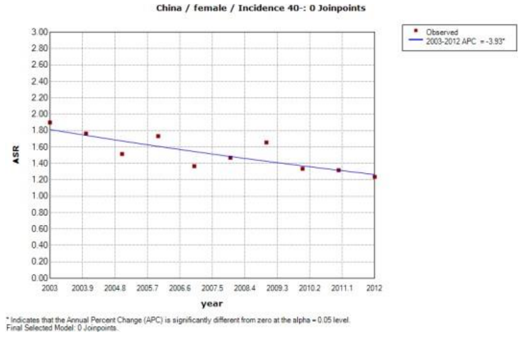

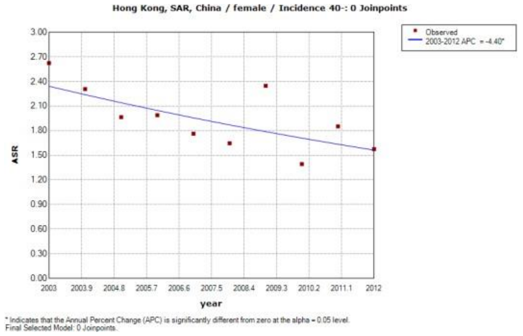

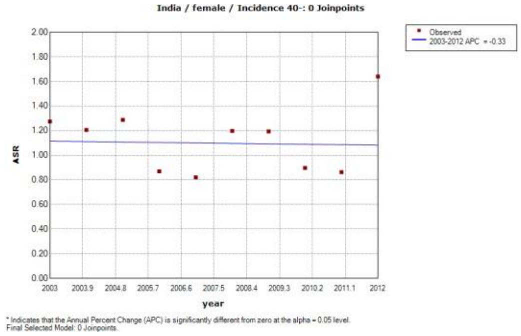

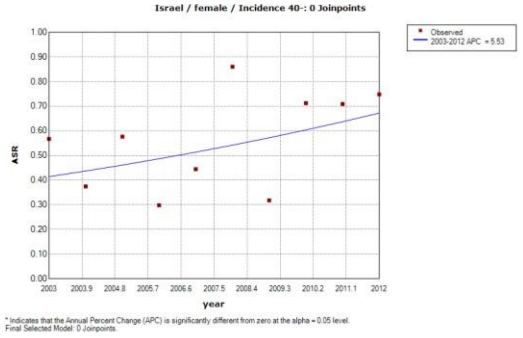

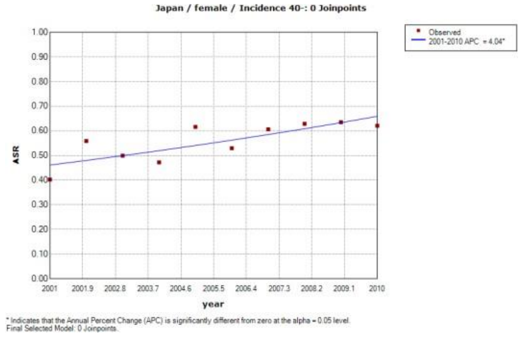

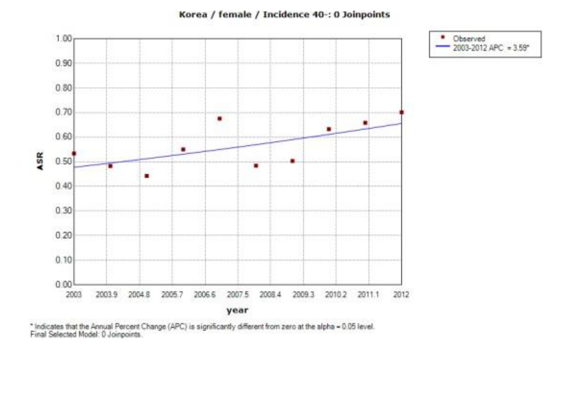
Korea / female / Incidence 40-: 0 Joinpoints
Observed
2003-2012 APC = 3.59*
ASR
year
* Indicates that the Annual Percent Change (APC) is significantly different from zero at the alpha = 0.05 level.
Final Selected Model: 0 Joinpoints.

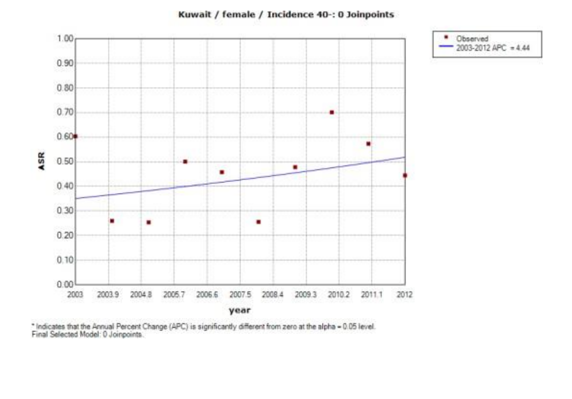
Kuwait / female / Incidence 40-: 0 Joinpoints
Observed
2003-2012 APC = 4.44
ASR
year
* Indicates that the Annual Percent Change (APC) is significantly different from zero at the alpha = 0.05 level.
Final Selected Model: 0 Joinpoints.

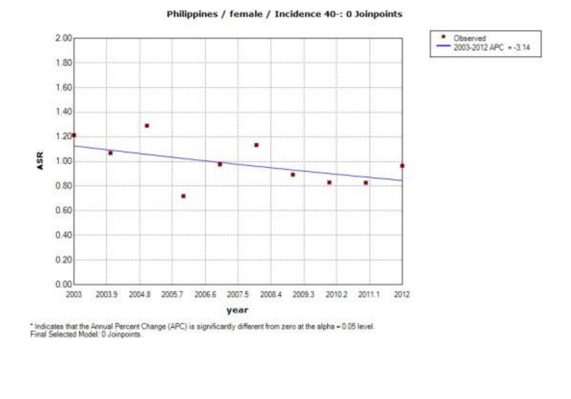
Philippines / female / Incidence 40-: 0 Joinpoints
Observed
2003-2012 APC = -3.14
ASR
year
* Indicates that the Annual Percent Change (APC) is significantly different from zero at the alpha = 0.05 level.
Final Selected Model: 0 Joinpoints.

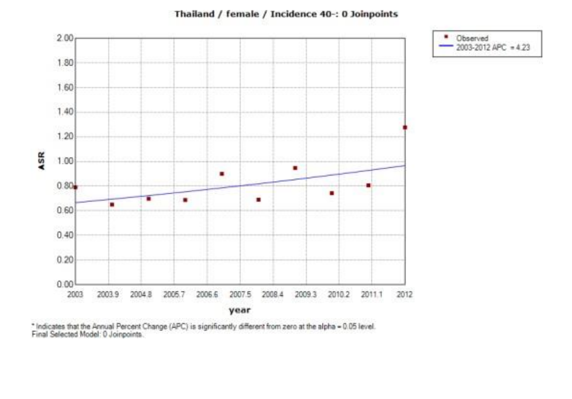
Thailand / female / Incidence 40-: 0 Joinpoints
Observed
2003-2012 APC = 4.23
ASR
year
* Indicates that the Annual Percent Change (APC) is significantly different from zero at the alpha = 0.05 level.
Final Selected Model: 0 Joinpoints.

Oceania

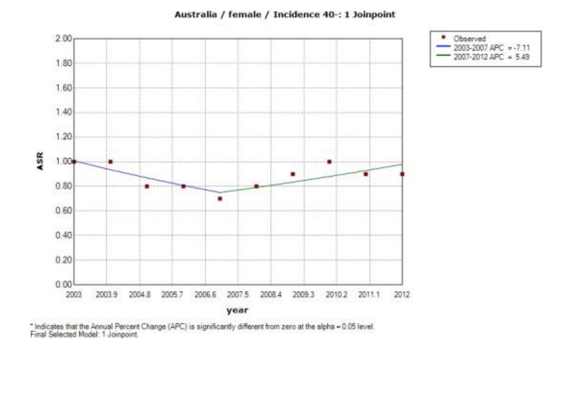
Australia / female / Incidence 40-: 1 Joinpoint
Observed
2003-2007 APC = -7.11
2007-2012 APC = 5.49
ASR
year
* Indicates that the Annual Percent Change (APC) is significantly different from zero at the alpha = 0.05 level.
Final Selected Model: 1 Joinpoint.

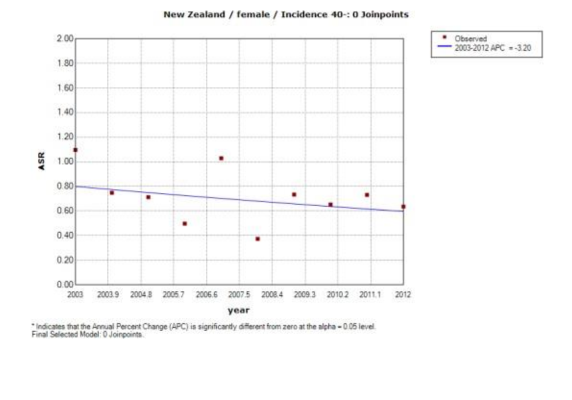
New Zealand / female / Incidence 40-: 0 Joinpoints
Observed
2003-2012 APC = -3.20
ASR
year
* Indicates that the Annual Percent Change (APC) is significantly different from zero at the alpha = 0.05 level.
Final Selected Model: 0 Joinpoints.

Northern America

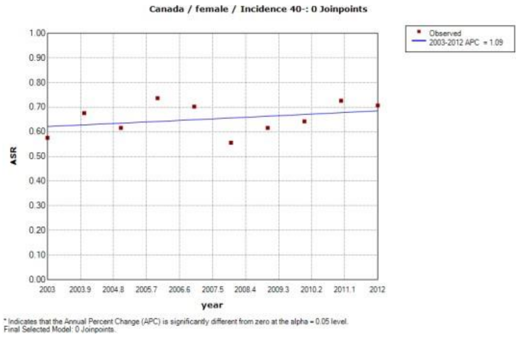

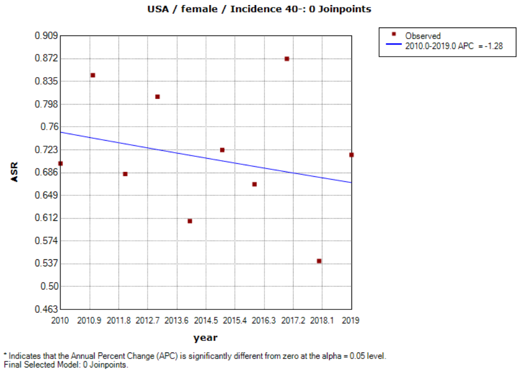

Southern America

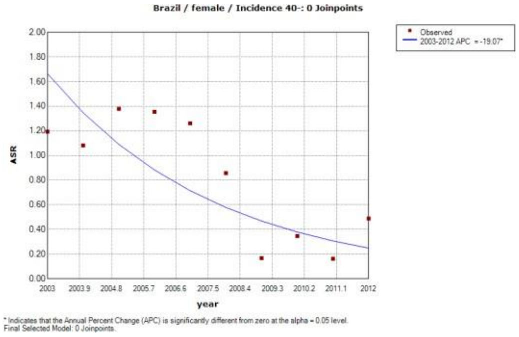

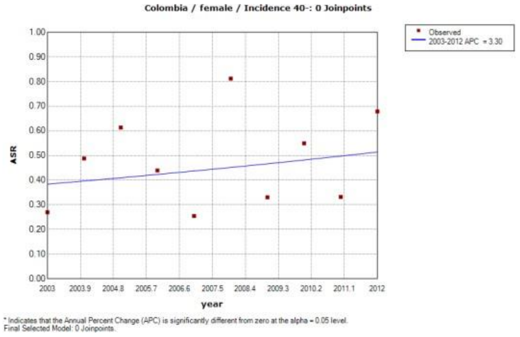

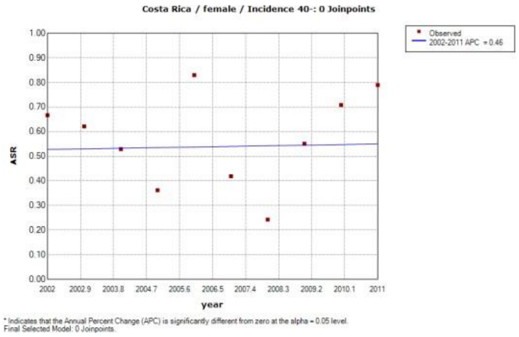

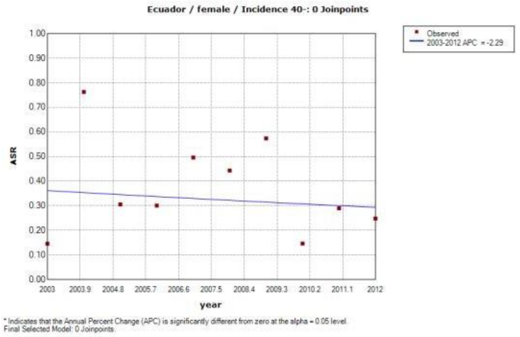

Northern Europe

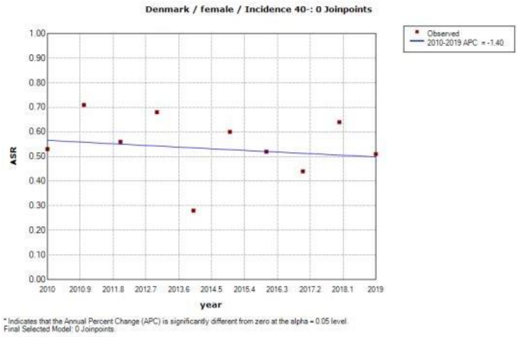

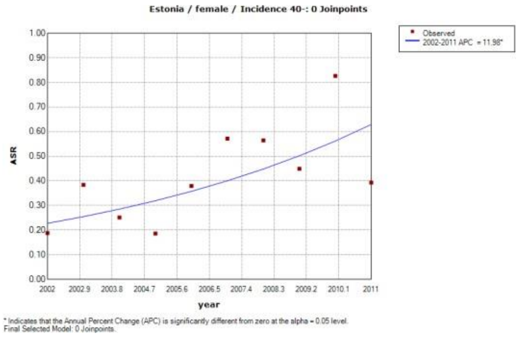

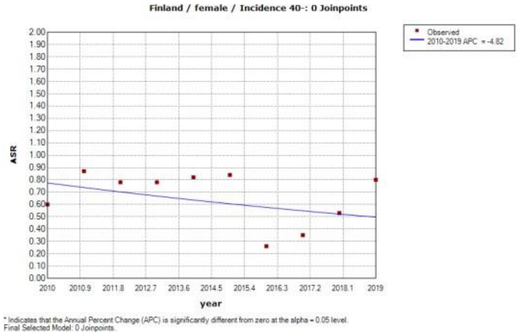

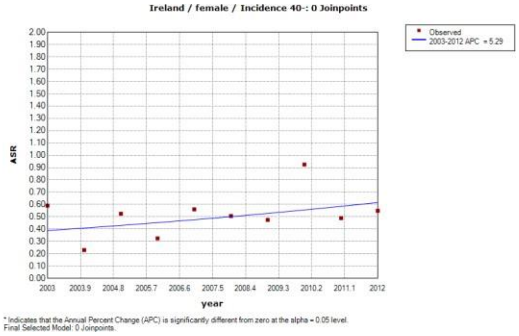

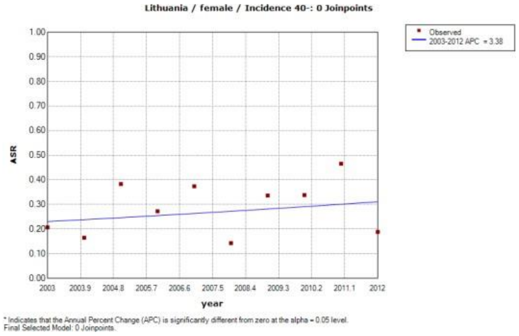

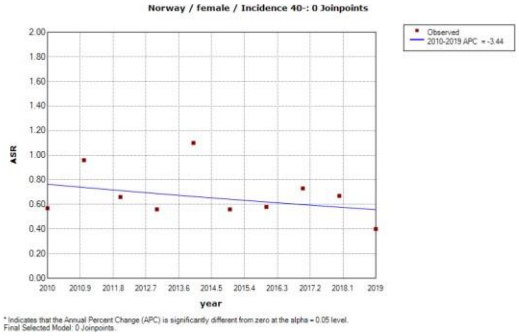

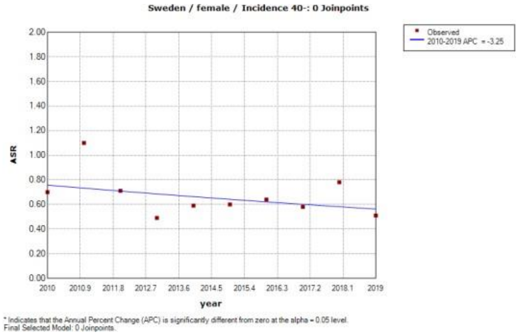

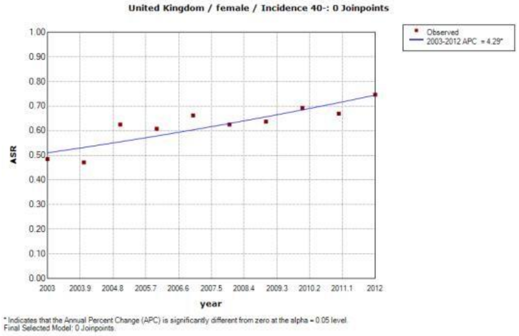

Western Europe

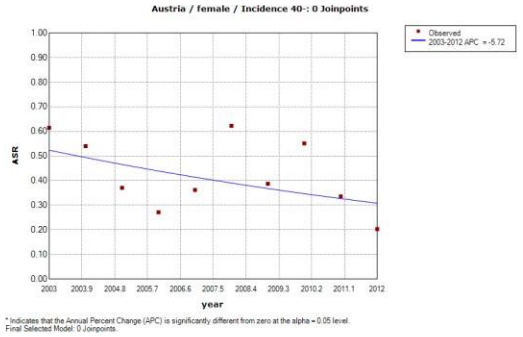

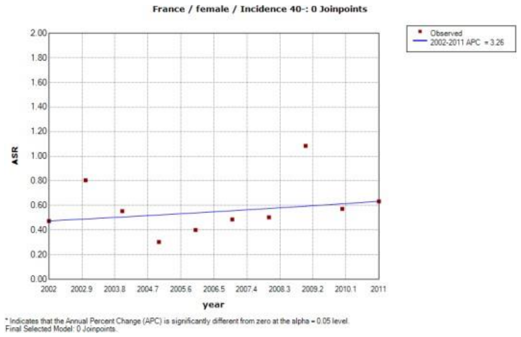

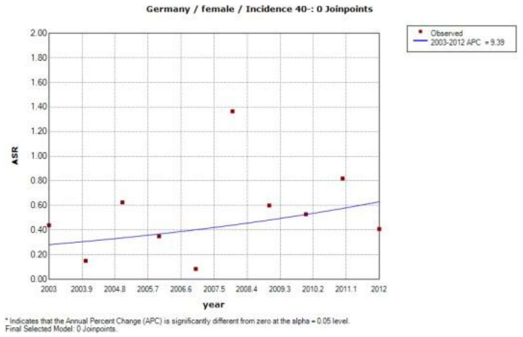

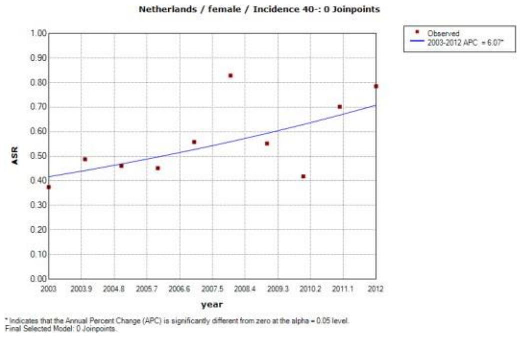

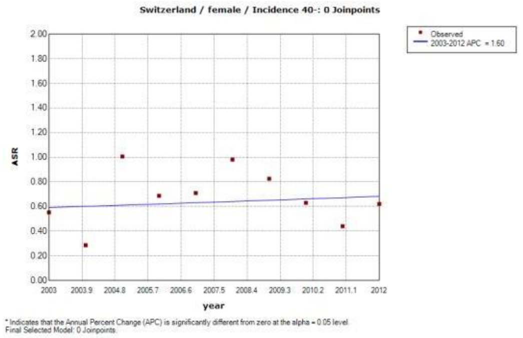

Southern Europe

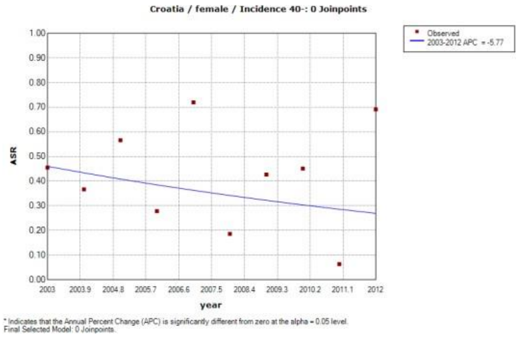

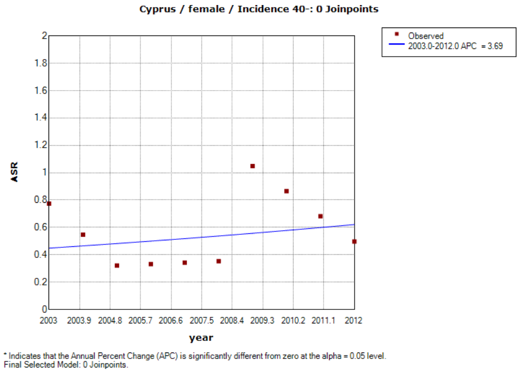

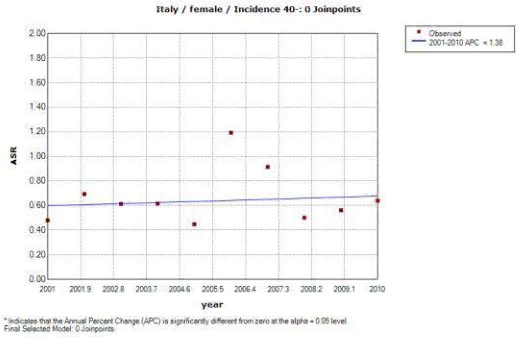

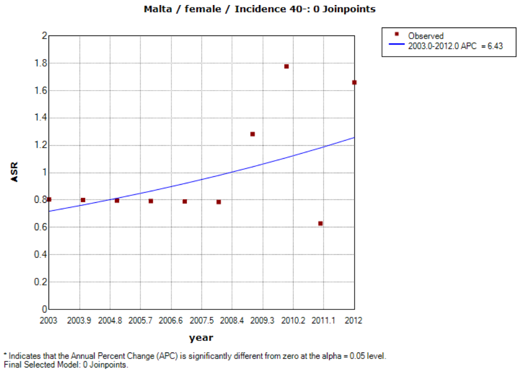

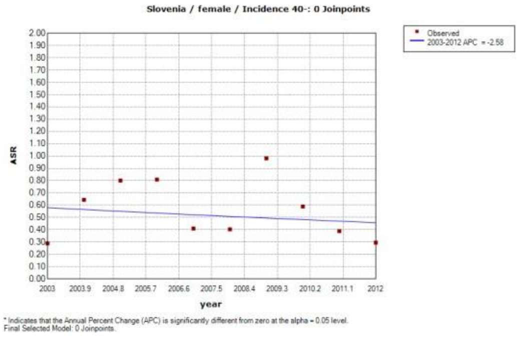

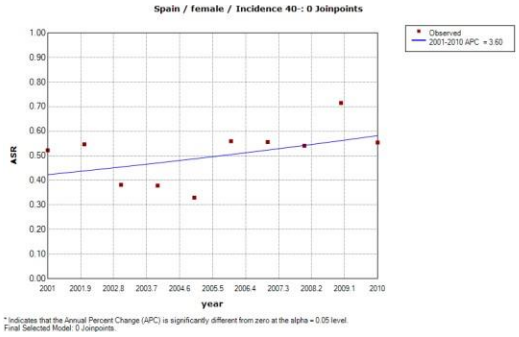

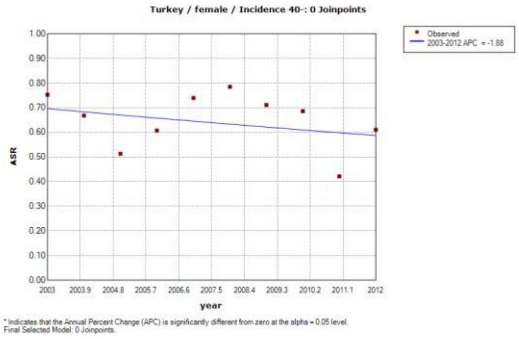

Eastern Europe

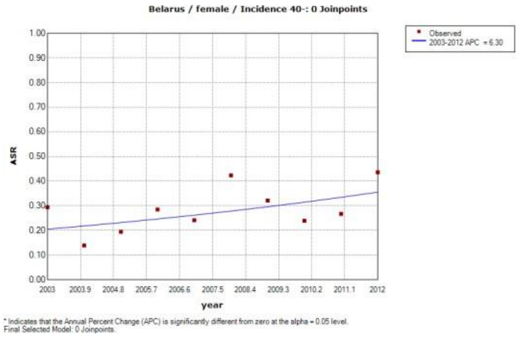

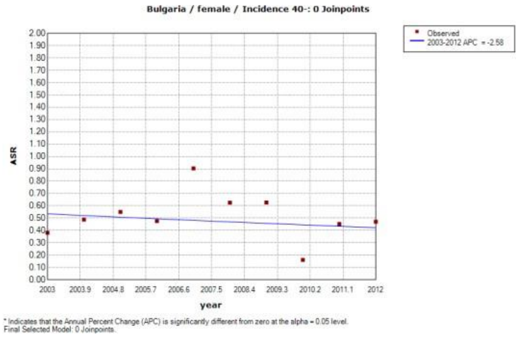

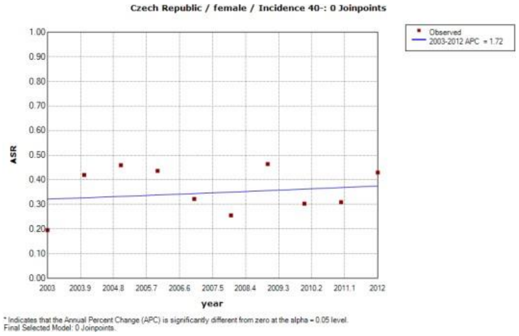

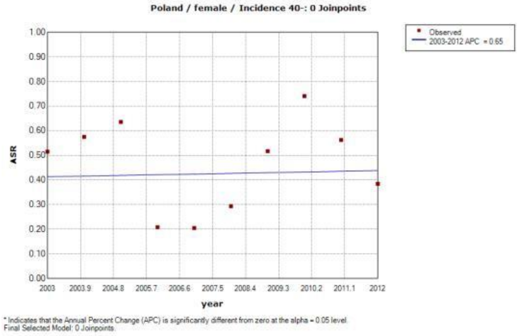

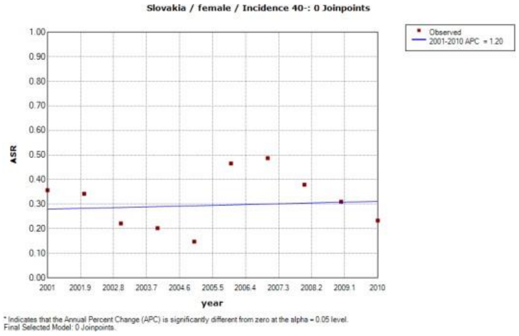

Africa

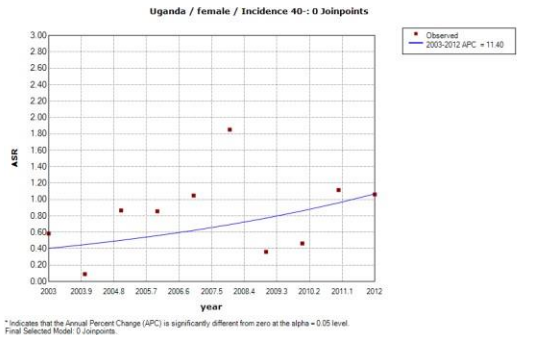
Uganda / female / Incidence 40-: 0 Joinpoints
Observed
2003-2012 APC = 11.40
ASR
3.00
2.80
2.60
2.40
2.20
2.00
1.80
1.60
1.40
1.20
1.00
0.80
0.60
0.40
0.20
0.00
2003
2003.9
2004.8
2005.7
2006.6
2007.5
2008.4
2009.3
2010.2
2011.1
2012
year
* Indicates that the Annual Percent Change (APC) is significantly different from zero at the alpha = 0.05 level.
Final Selected Model: 0 Joinpoints.

e.) Incidence male below 50 years old

Asia

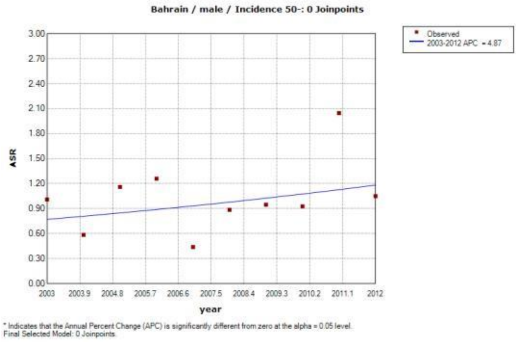

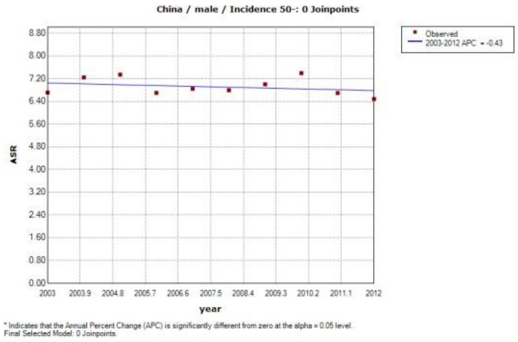

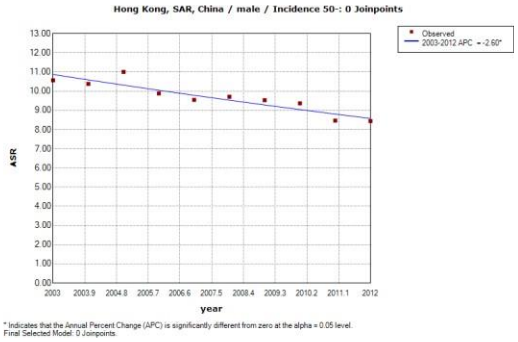

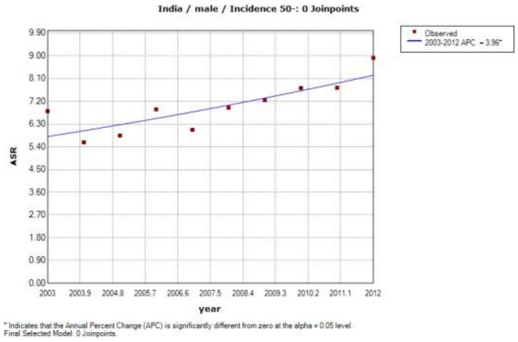

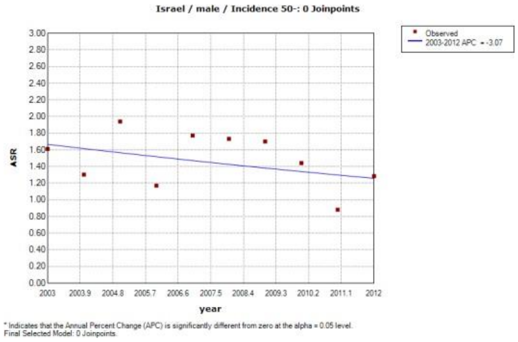

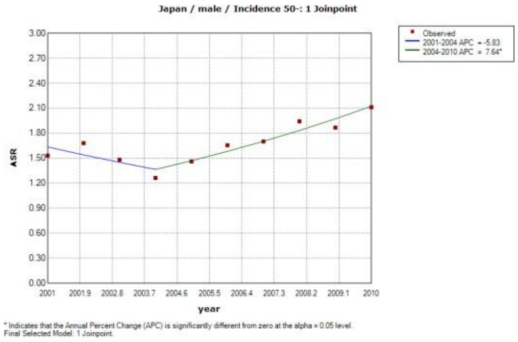

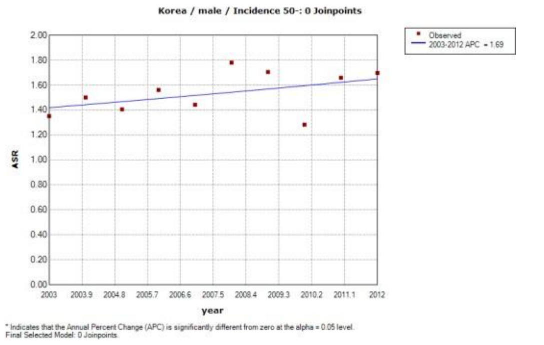
Korea / male / Incidence 50-: 0 Joinpoints
Observed
2003-2012 APC = 1.69
ASR
year
* Indicates that the Annual Percent Change (APC) is significantly different from zero at the alpha = 0.05 level.
Final Selected Model: 0 Joinpoints.

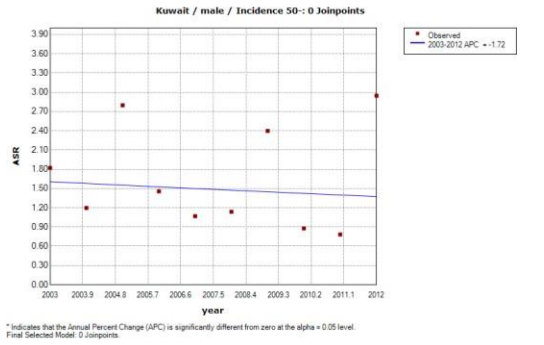
Kuwait / male / Incidence 50-: 0 Joinpoints
Observed
2003-2012 APC = -1.72
ASR
year
* Indicates that the Annual Percent Change (APC) is significantly different from zero at the alpha = 0.05 level.
Final Selected Model: 0 Joinpoints.

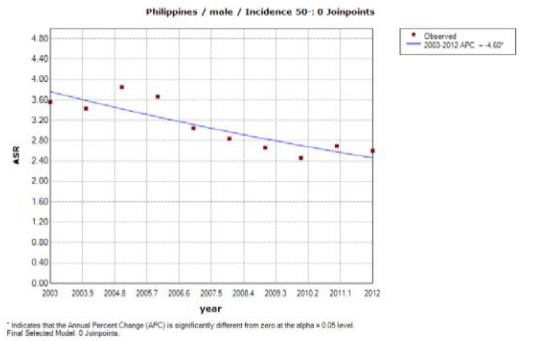
Philippines / male / Incidence 50-: 0 Joinpoints
Observed
2003-2012 APC = -4.60*
ASR
year
* Indicates that the Annual Percent Change (APC) is significantly different from zero at the alpha = 0.05 level.
Final Selected Model: 0 Joinpoints.

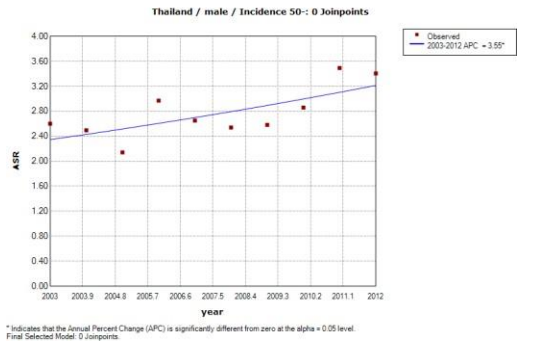
Thailand / male / Incidence 50-: 0 Joinpoints
Observed
2003-2012 APC = 3.55*
ASR
year
* Indicates that the Annual Percent Change (APC) is significantly different from zero at the alpha = 0.05 level.
Final Selected Model: 0 Joinpoints.

Oceania

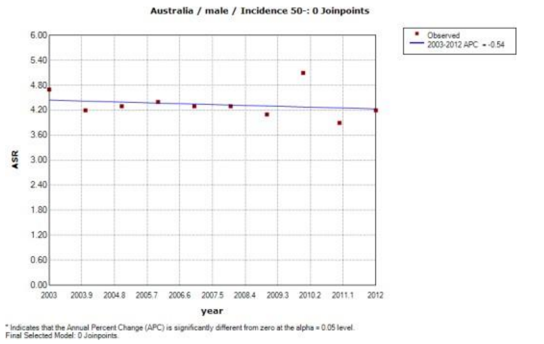
Australia / male / Incidence 50-: 0 Joinpoints
Observed
2003-2012 APC = -0.54
ASR
year
* Indicates that the Annual Percent Change (APC) is significantly different from zero at the alpha = 0.05 level.
Final Selected Model: 0 Joinpoints.

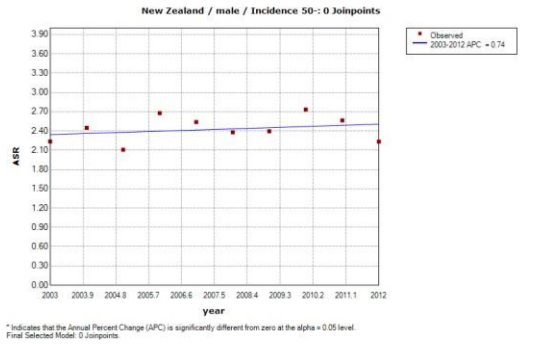
New Zealand / male / Incidence 50-: 0 Joinpoints
Observed
2003-2012 APC = 0.74
ASR
year
* Indicates that the Annual Percent Change (APC) is significantly different from zero at the alpha = 0.05 level.
Final Selected Model: 0 Joinpoints.

Northern America

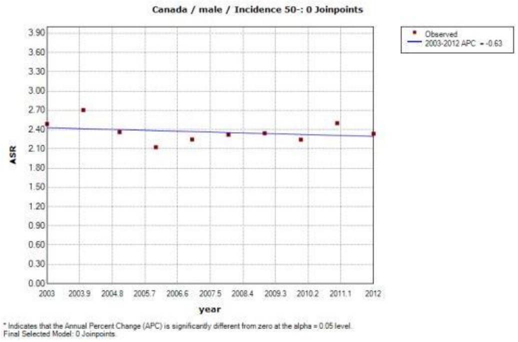

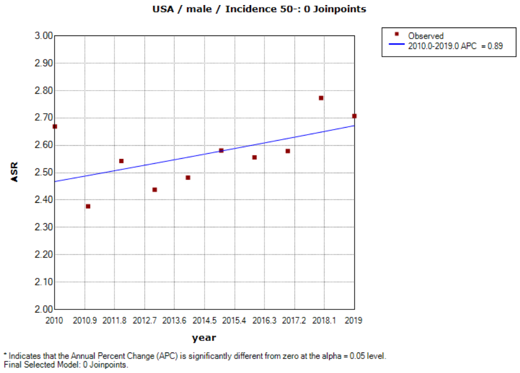

Southern America

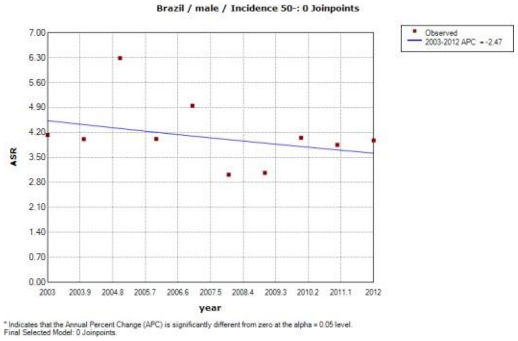

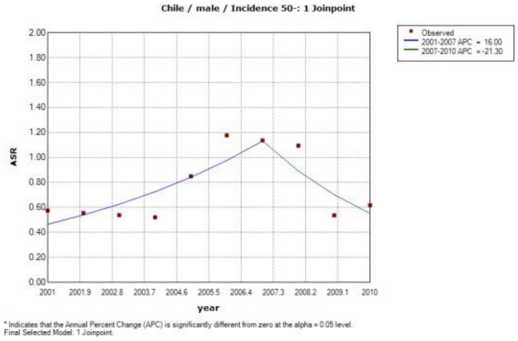

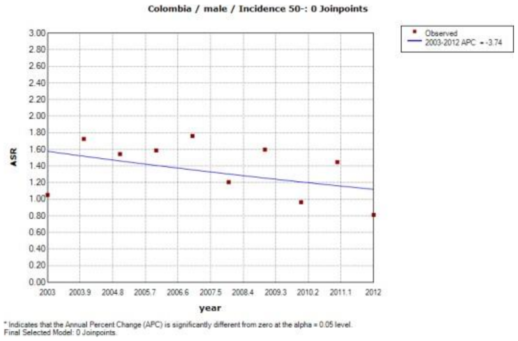

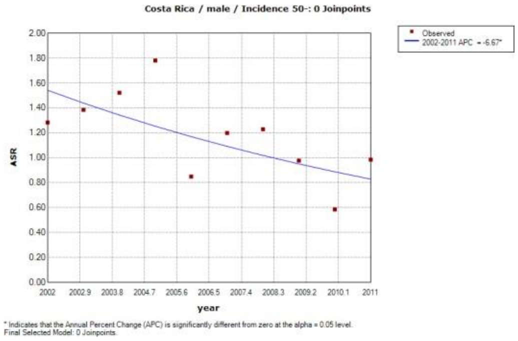

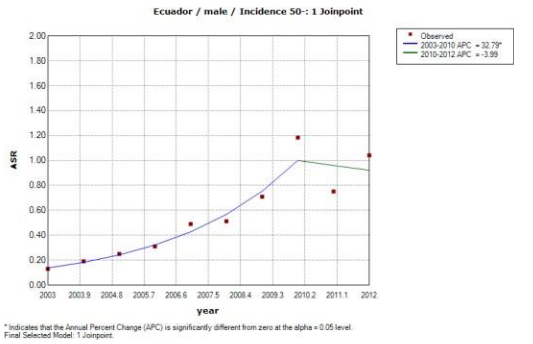

Northern Europe

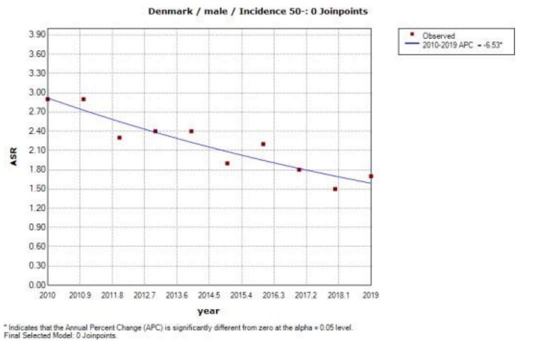

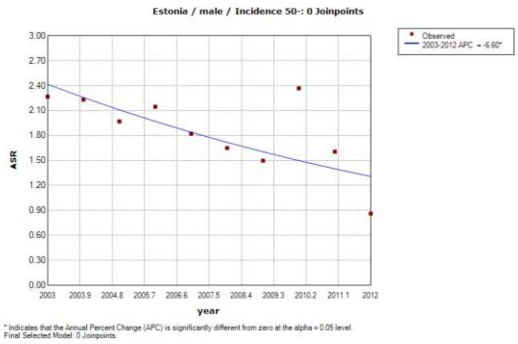

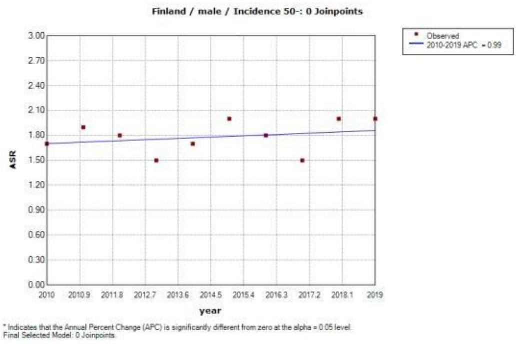

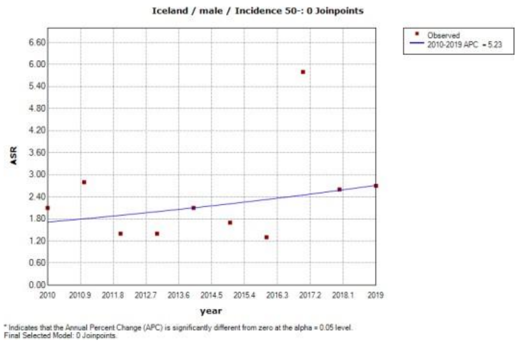

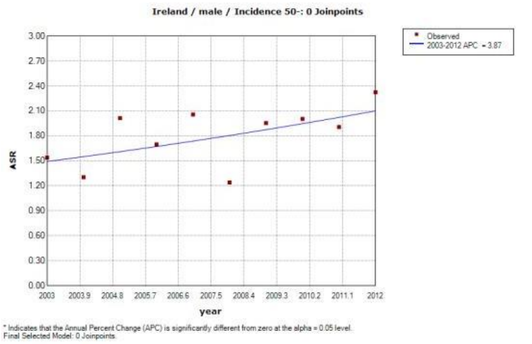

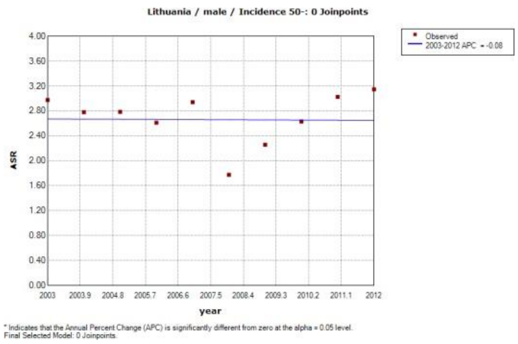

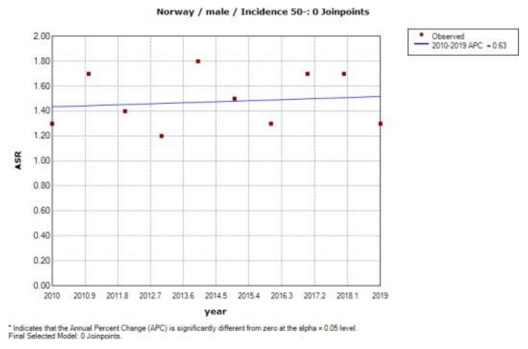

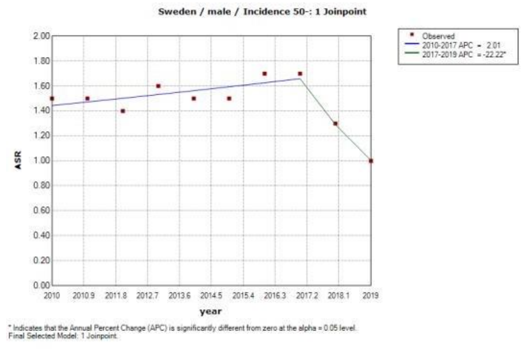

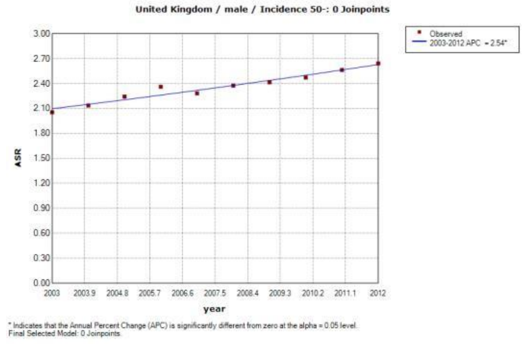

Western Europe

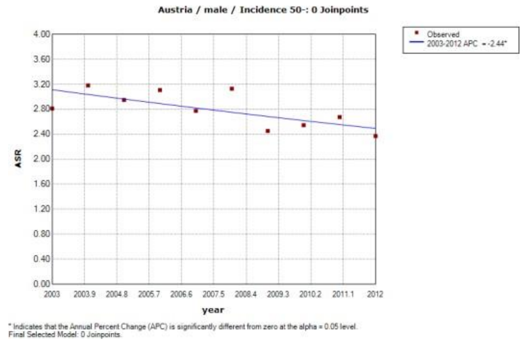

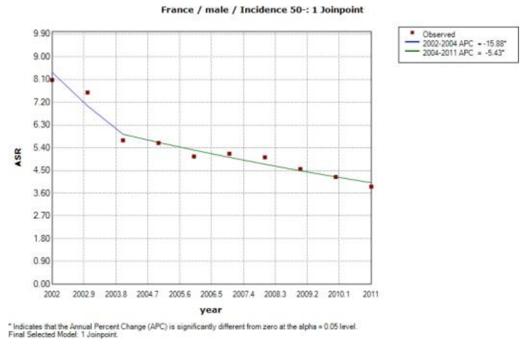

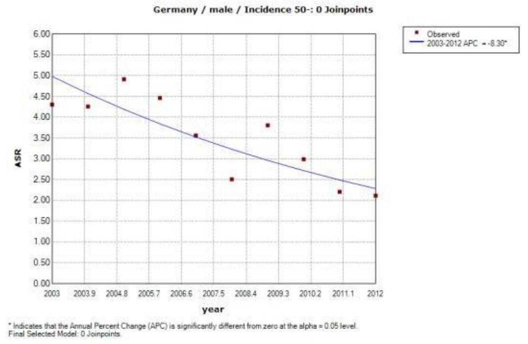

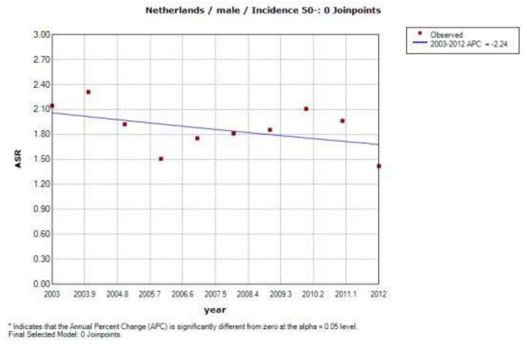

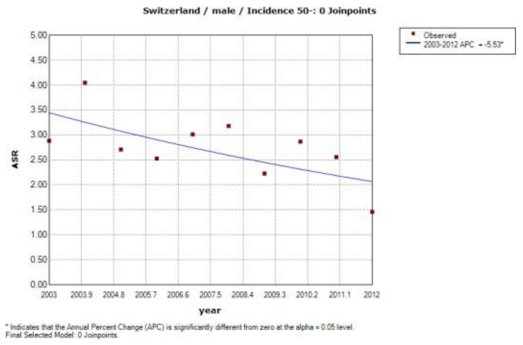

Southern Europe

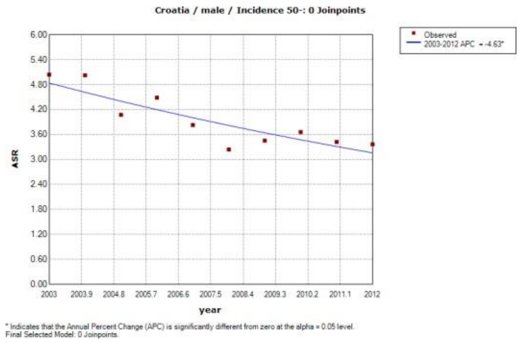

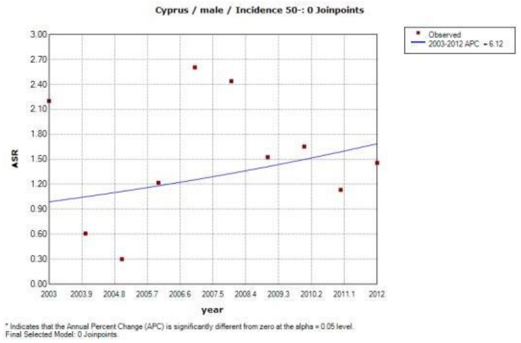

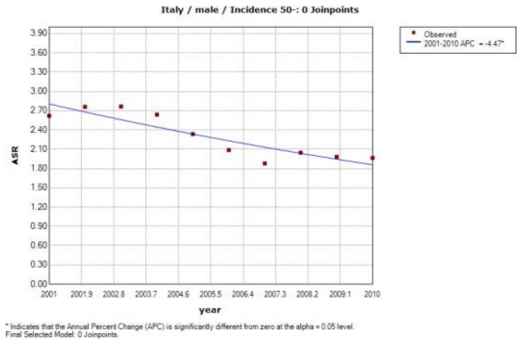

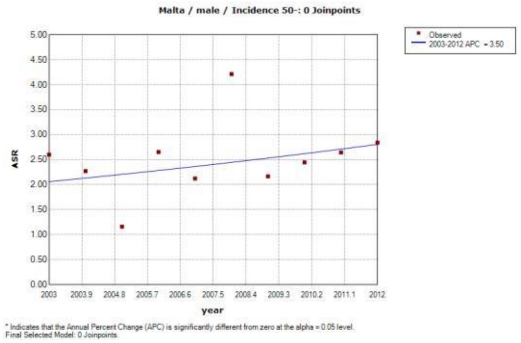

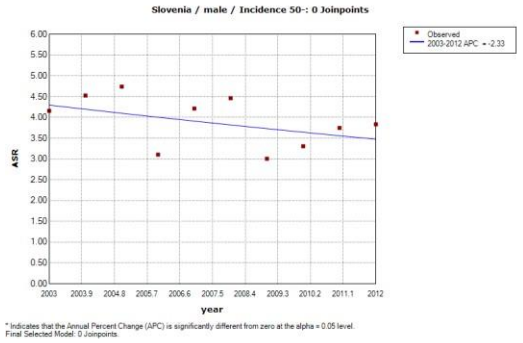

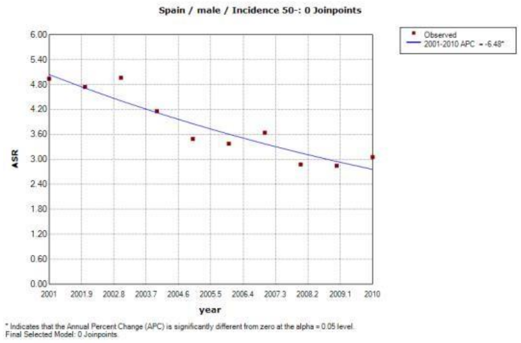

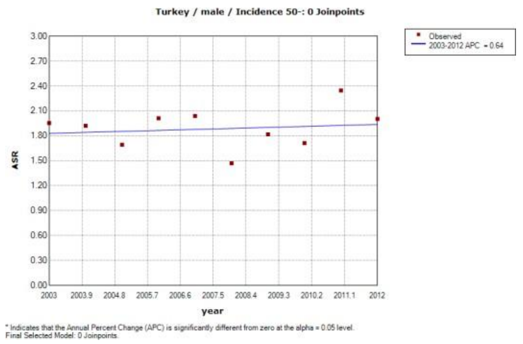

Eastern Europe

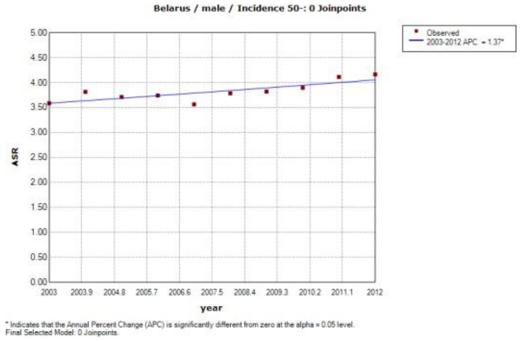

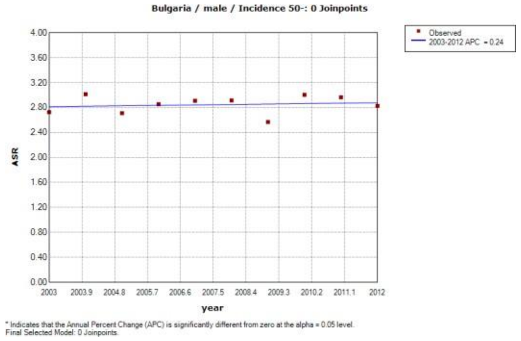

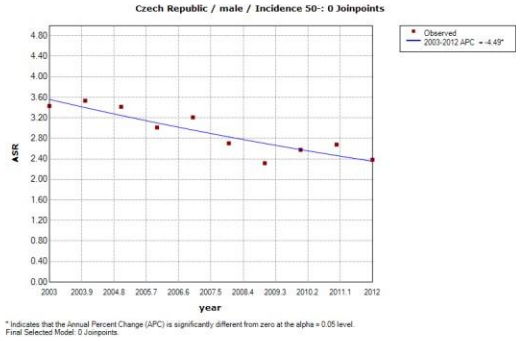

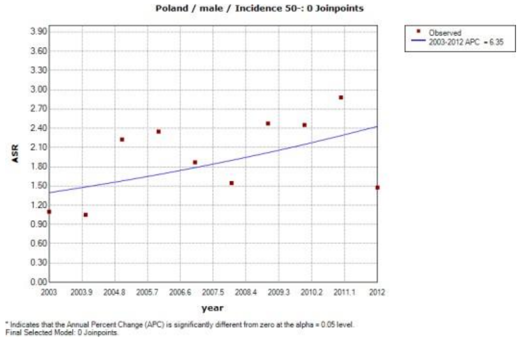

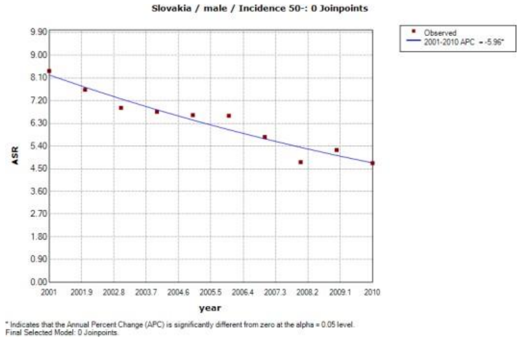

Africa	
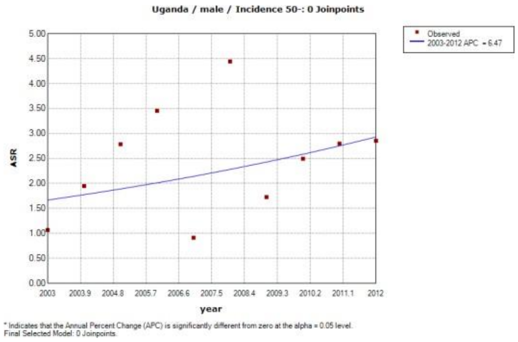Uganda / male / Incidence 50-: 0 Joinpoints
Observed
2003-2012 APC = 6.47
ASR
5.00
4.50
4.00
3.50
3.00
2.50
2.00
1.50
1.00
0.50
0.00
2003
2003.9
2004.8
2005.7
2006.6
2007.5
2008.4
2009.3
2010.2
2011.1
2012
year
* Indicates that the Annual Percent Change (APC) is significantly different from zero at the alpha = 0.05 level.
Final Selected Model: 0 Joinpoints.	

F .) Incidence female below 50 years old

Asia

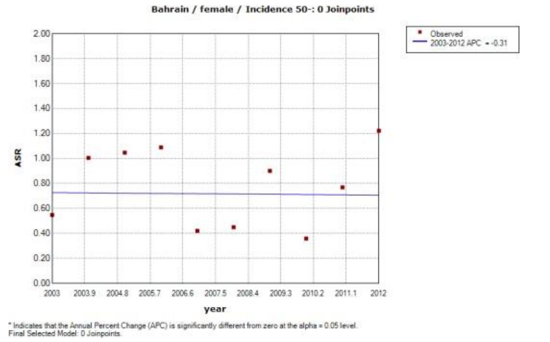

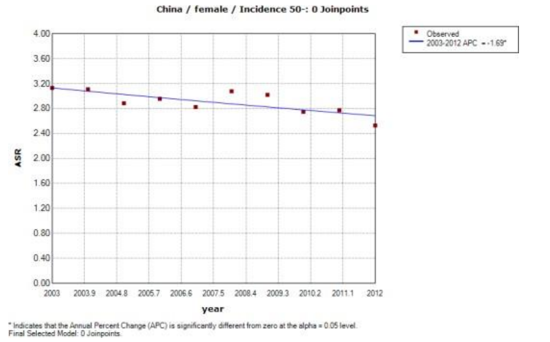

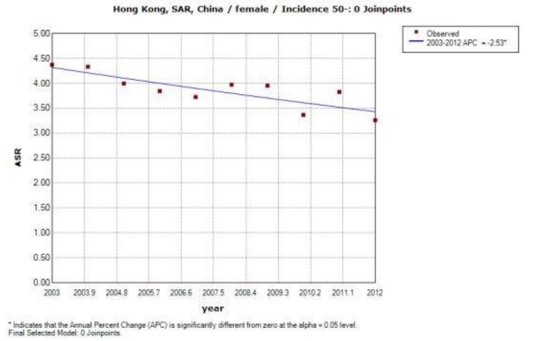

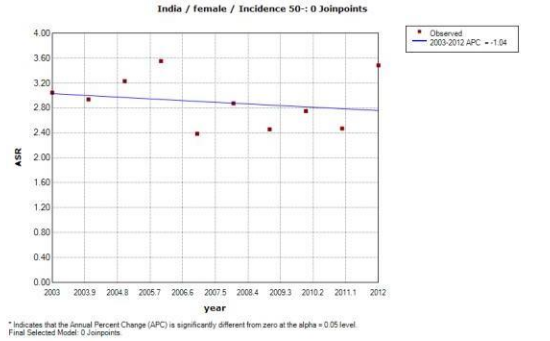

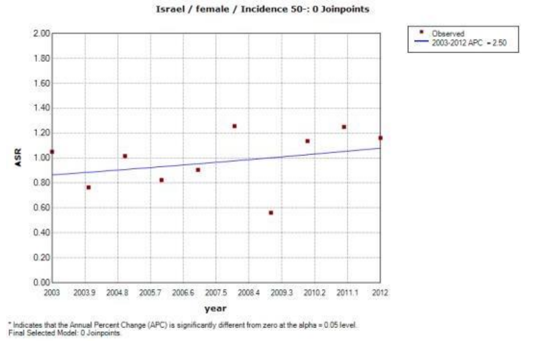

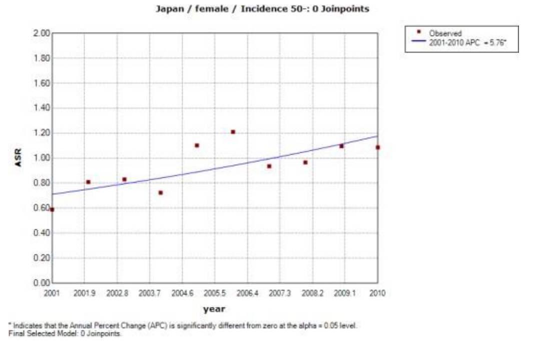

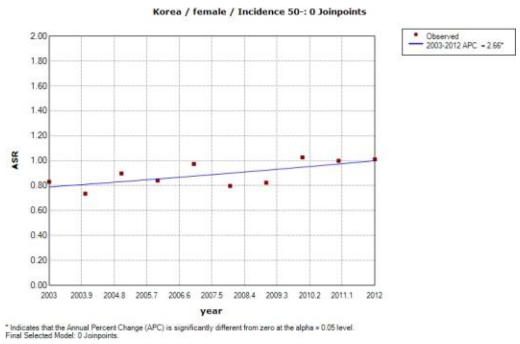
Korea / female / Incidence 50-: 0 Joinpoints
Observed
2003-2012 APC = 2.66*
ASR
year
* Indicates that the Annual Percent Change (APC) is significantly different from zero at the alpha = 0.05 level.
Final Selected Model: 0 Joinpoints.

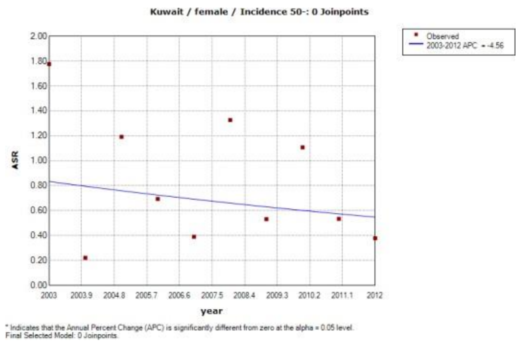
Kuwait / female / Incidence 50-: 0 Joinpoints
Observed
2003-2012 APC = -4.56
ASR
year
* Indicates that the Annual Percent Change (APC) is significantly different from zero at the alpha = 0.05 level.
Final Selected Model: 0 Joinpoints.

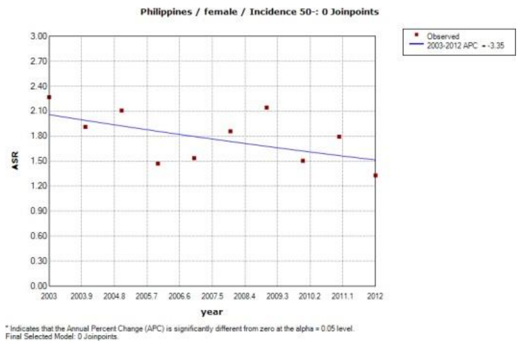
Philippines / female / Incidence 50-: 0 Joinpoints
Observed
2003-2012 APC = -3.35
ASR
year
* Indicates that the Annual Percent Change (APC) is significantly different from zero at the alpha = 0.05 level.
Final Selected Model: 0 Joinpoints.

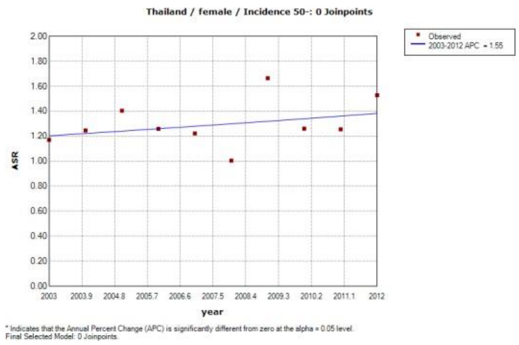
Thailand / female / Incidence 50-: 0 Joinpoints
Observed
2003-2012 APC = 1.55
ASR
year
* Indicates that the Annual Percent Change (APC) is significantly different from zero at the alpha = 0.05 level.
Final Selected Model: 0 Joinpoints.

Oceania

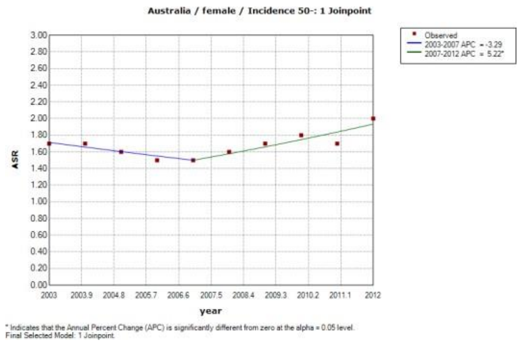
Australia / female / Incidence 50-: 1 Joinpoint
Observed
2003-2007 APC = -3.29
2007-2012 APC = 5.22*
ASR
year
* Indicates that the Annual Percent Change (APC) is significantly different from zero at the alpha = 0.05 level.
Final Selected Model: 1 Joinpoint.

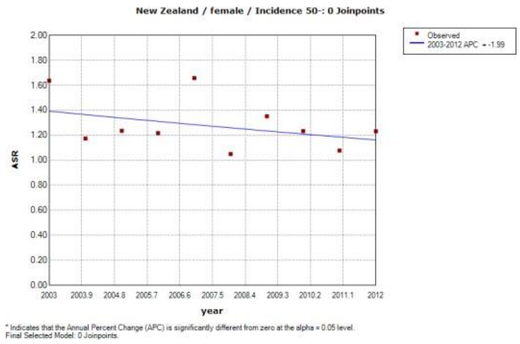
New Zealand / female / Incidence 50-: 0 Joinpoints
Observed
2003-2012 APC = -1.99
ASR
year
* Indicates that the Annual Percent Change (APC) is significantly different from zero at the alpha = 0.05 level.
Final Selected Model: 0 Joinpoints.

Northern America

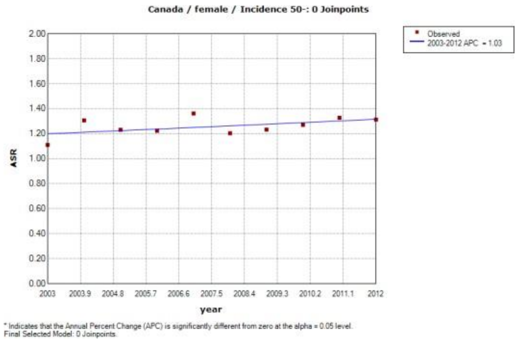

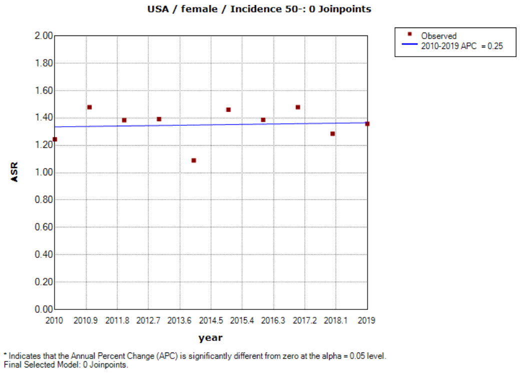

Southern America

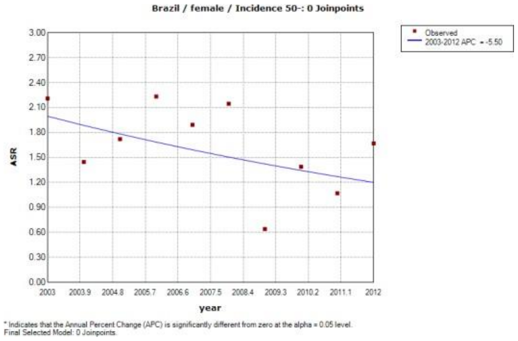

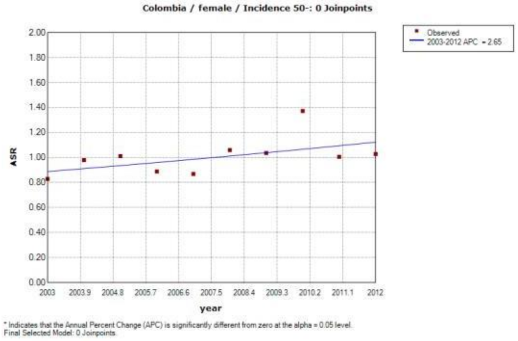

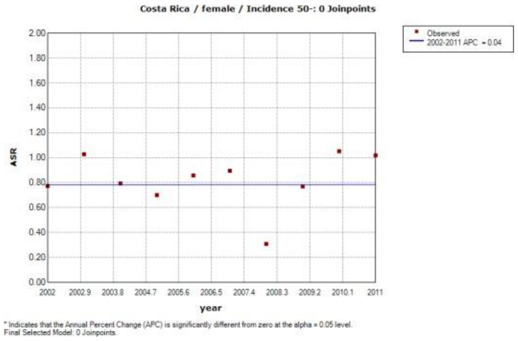

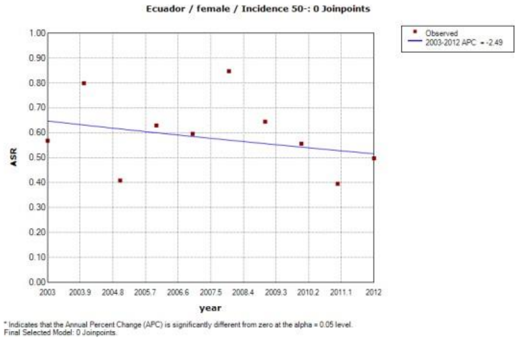

Northern Europe

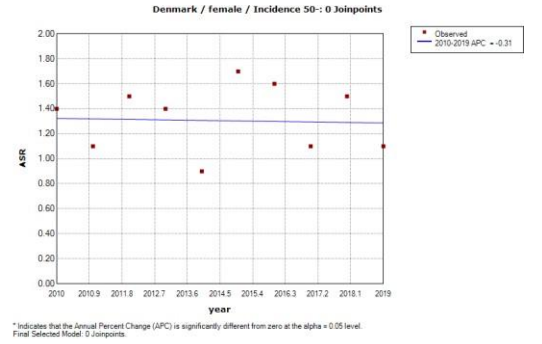

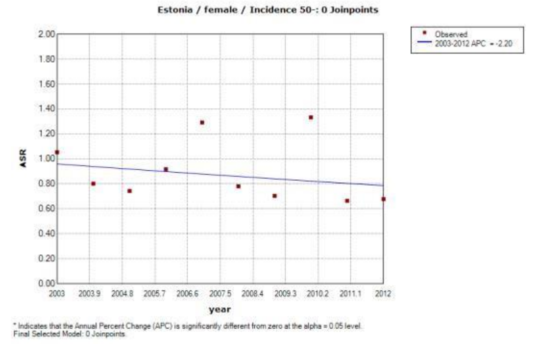

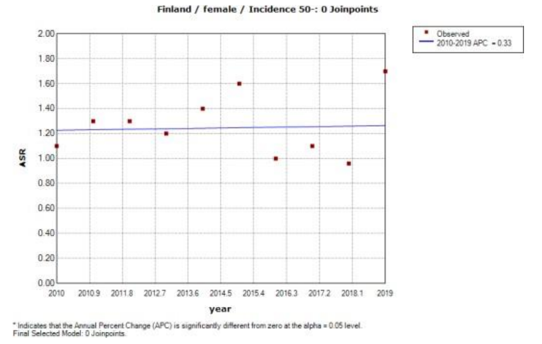

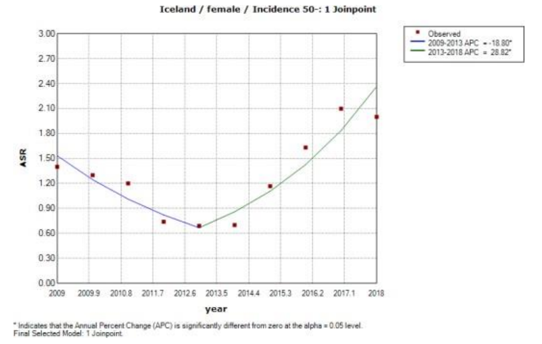

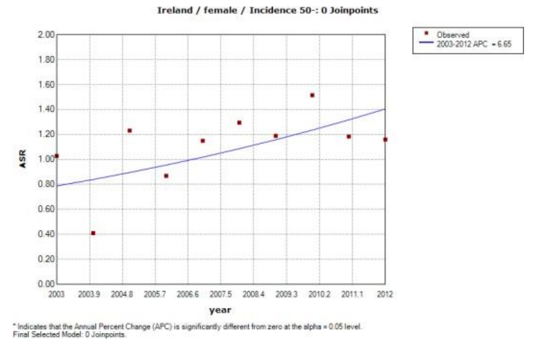

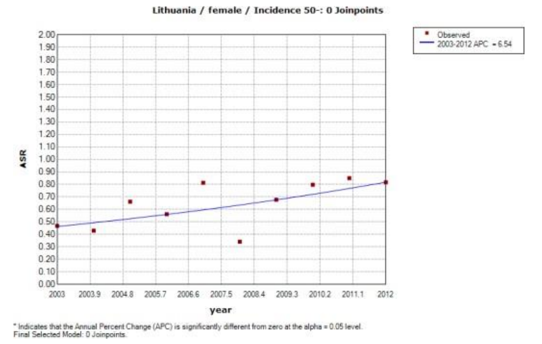

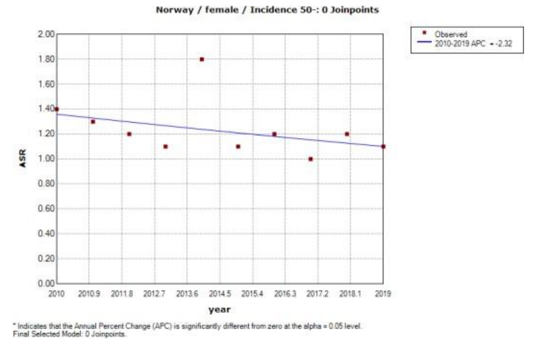

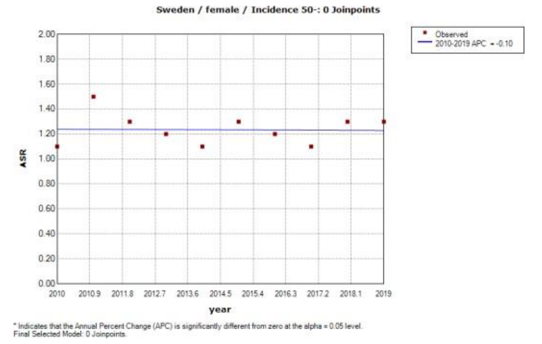

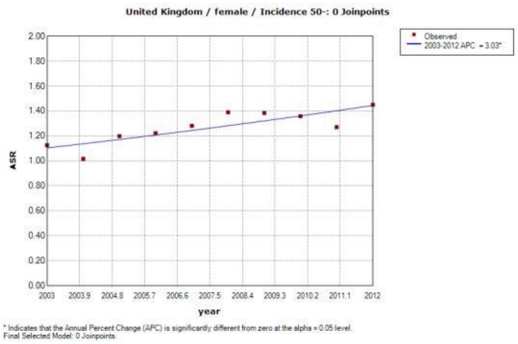

Western Europe

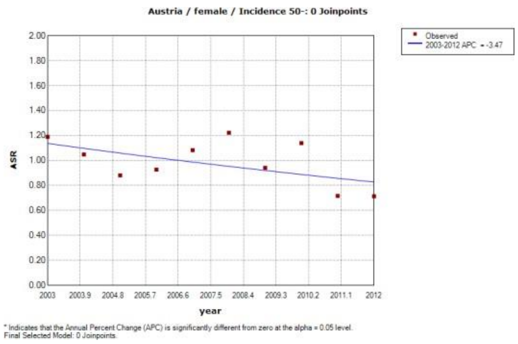

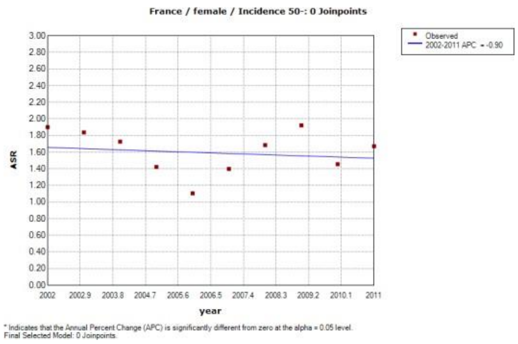

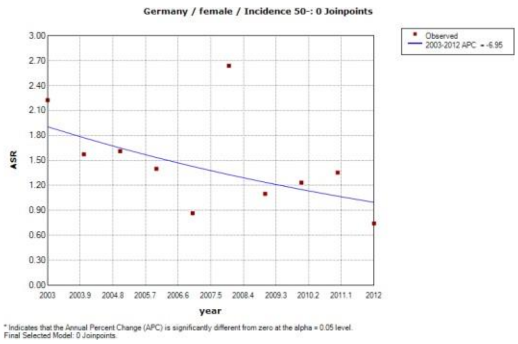

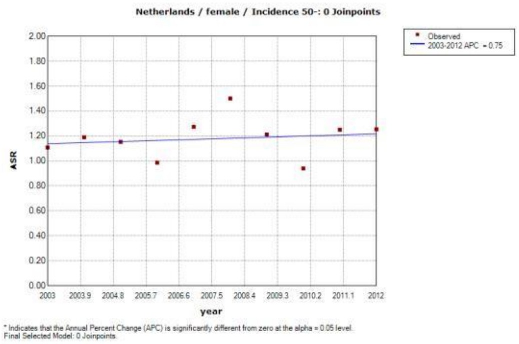

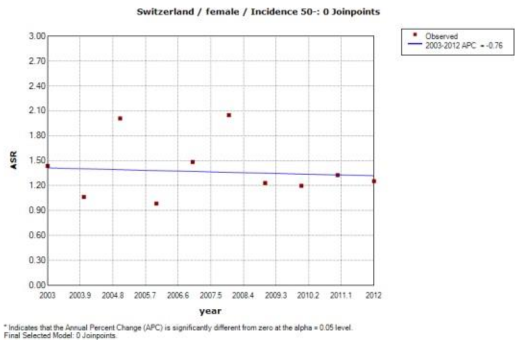

Southern Europe

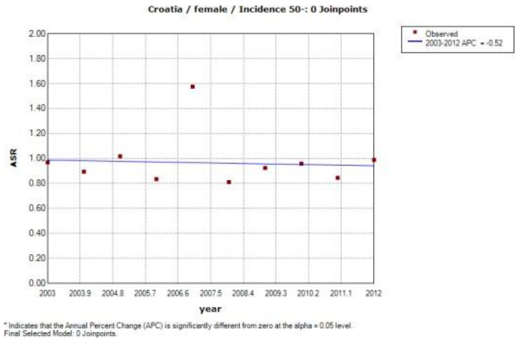

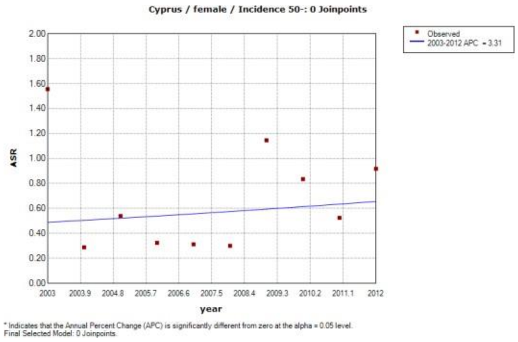

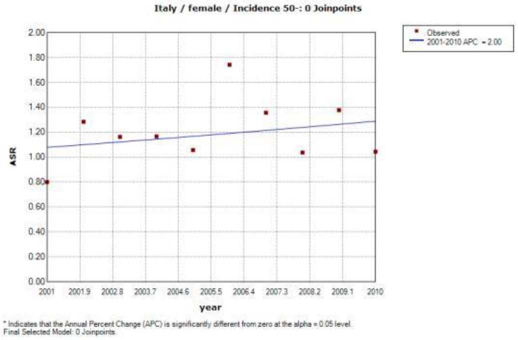

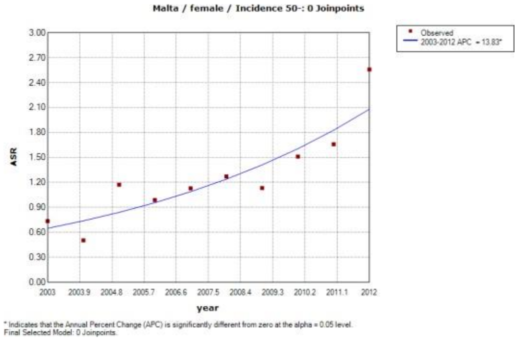

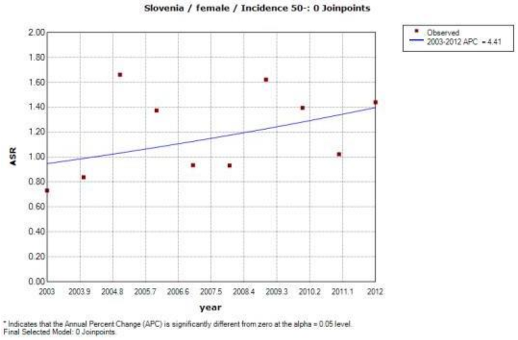

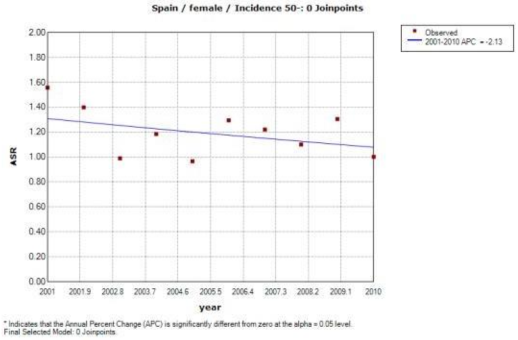

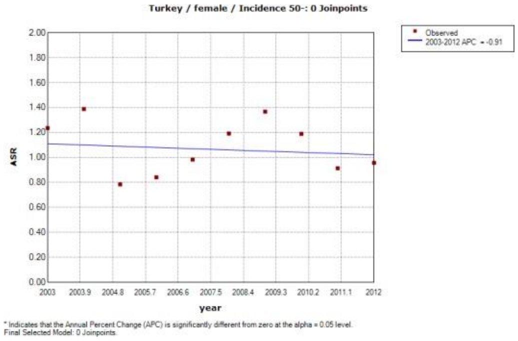

Eastern Europe

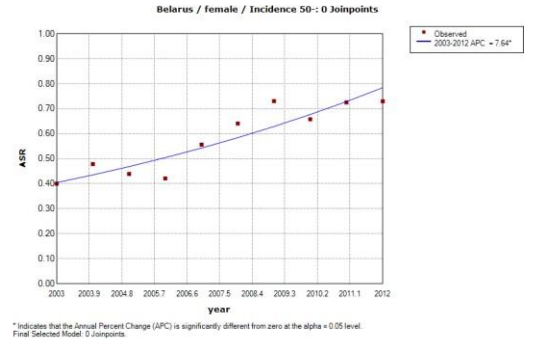

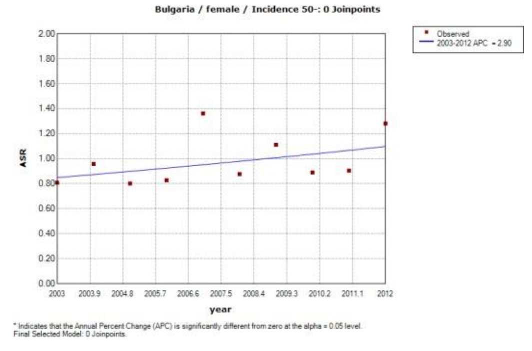

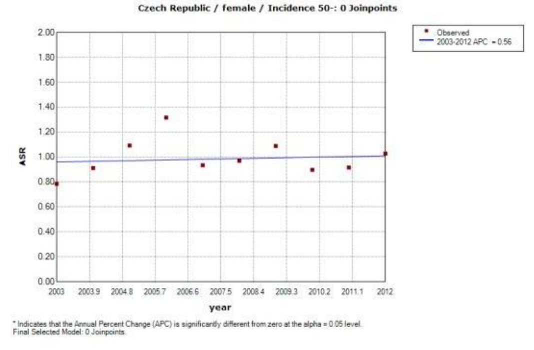

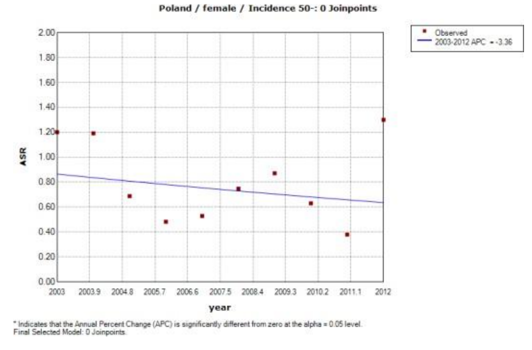

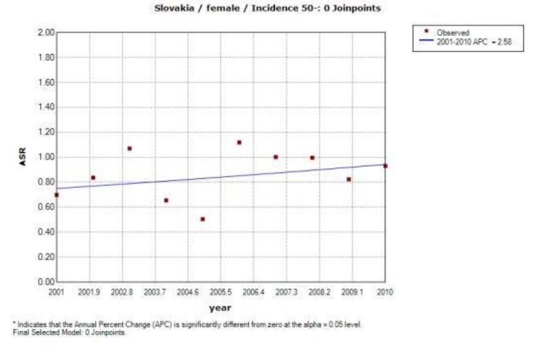

Africa

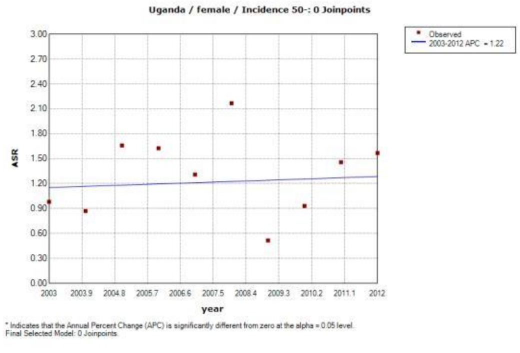
Uganda / female / Incidence 50-: 0 Joinpoints
Observed
2003-2012 APC = 1.22
3.00
2.70
2.40
2.10
1.80
1.50
1.20
0.90
0.60
0.30
0.00
ASR
2003
2003.9
2004.8
2005.7
2006.6
2007.5
2008.4
2009.3
2010.2
2011.1
2012
year
* Indicates that the Annual Percent Change (APC) is significantly different from zero at the alpha = 0.05 level.
Final Selected Model: 0 Joinpoints.

g.) Incidence male above 50 years old

Asia

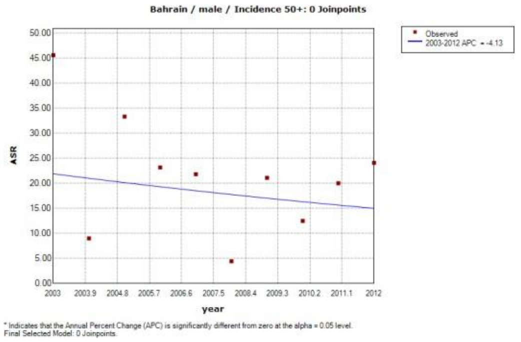

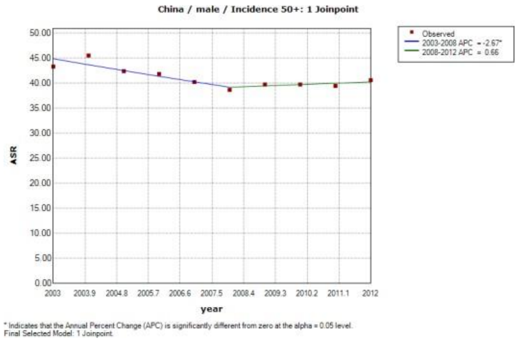

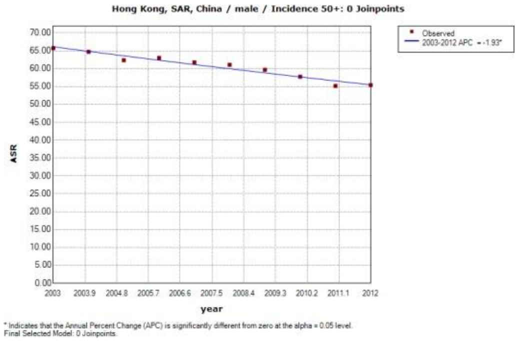

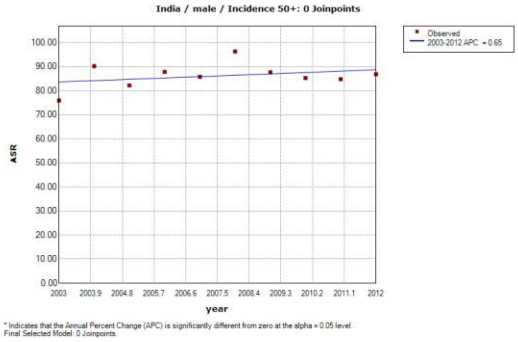

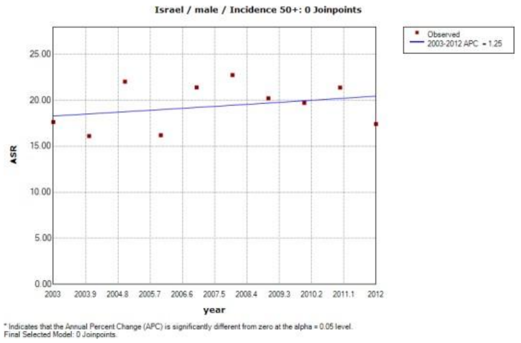

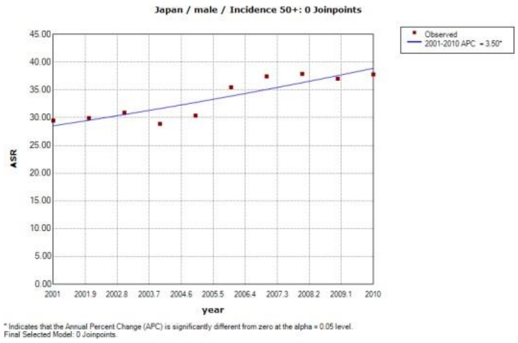

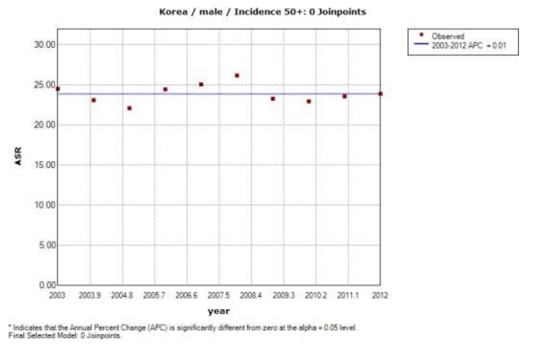
Korea / male / Incidence 50+: 0 Joinpoints
Observed
2003-2012 APC = 0.01
ASR
year
* Indicates that the Annual Percent Change (APC) is significantly different from zero at the alpha = 0.05 level.
Final Selected Model: 0 Joinpoints.

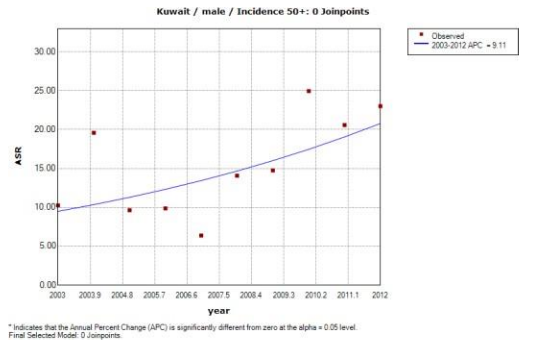
Kuwait / male / Incidence 50+: 0 Joinpoints
Observed
2003-2012 APC = 9.11
ASR
year
* Indicates that the Annual Percent Change (APC) is significantly different from zero at the alpha = 0.05 level.
Final Selected Model: 0 Joinpoints.

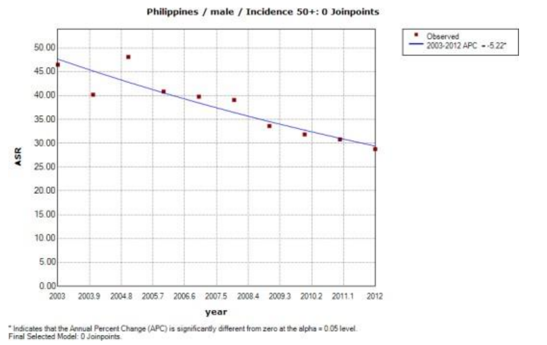
Philippines / male / Incidence 50+: 0 Joinpoints
Observed
2003-2012 APC = -5.22*
ASR
year
* Indicates that the Annual Percent Change (APC) is significantly different from zero at the alpha = 0.05 level.
Final Selected Model: 0 Joinpoints.

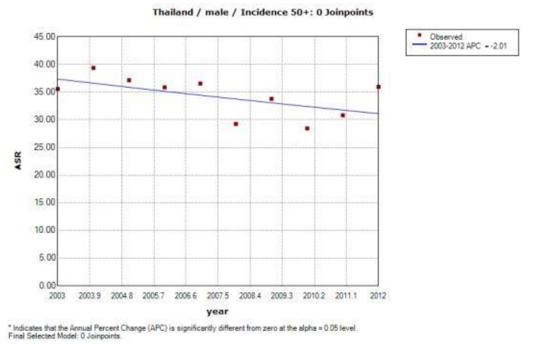
Thailand / male / Incidence 50+: 0 Joinpoints
Observed
2003-2012 APC = -2.01
ASR
year
* Indicates that the Annual Percent Change (APC) is significantly different from zero at the alpha = 0.05 level.
Final Selected Model: 0 Joinpoints.

Oceania

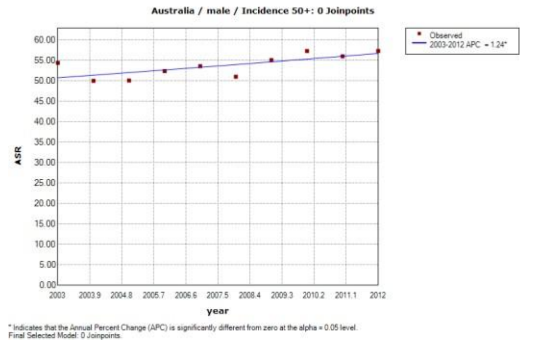
Australia / male / Incidence 50+: 0 Joinpoints
Observed
2003-2012 APC = 1.24*
ASR
year
* Indicates that the Annual Percent Change (APC) is significantly different from zero at the alpha = 0.05 level.
Final Selected Model: 0 Joinpoints.

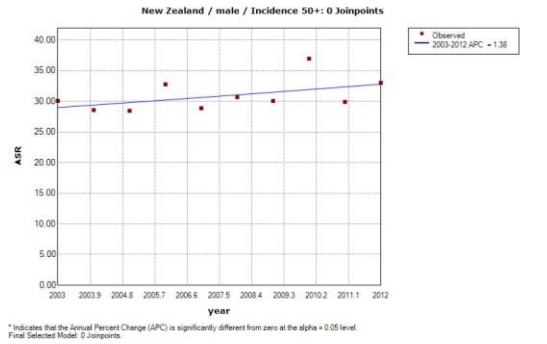
New Zealand / male / Incidence 50+: 0 Joinpoints
Observed
2003-2012 APC = 1.38
ASR
year
* Indicates that the Annual Percent Change (APC) is significantly different from zero at the alpha = 0.05 level.
Final Selected Model: 0 Joinpoints.

Northern America

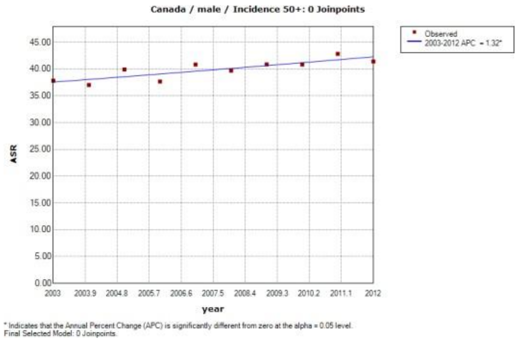

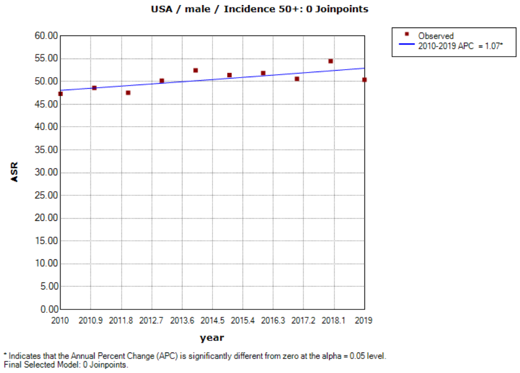

Southern America

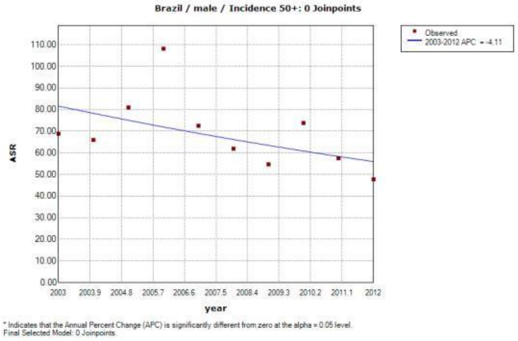

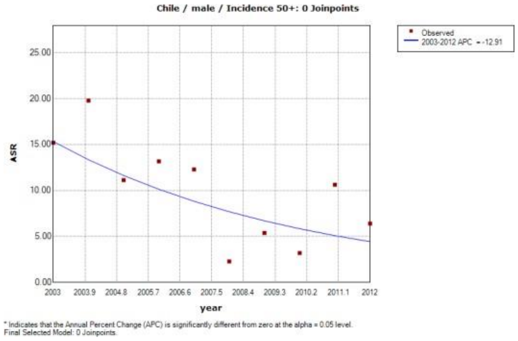

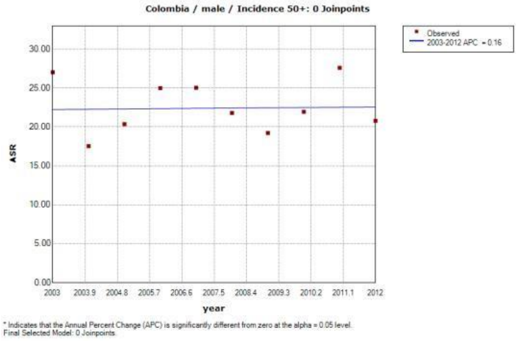

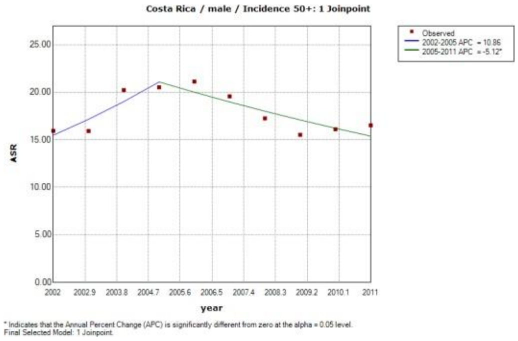

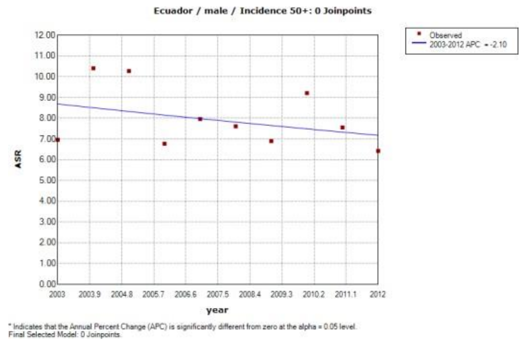

Northern Europe

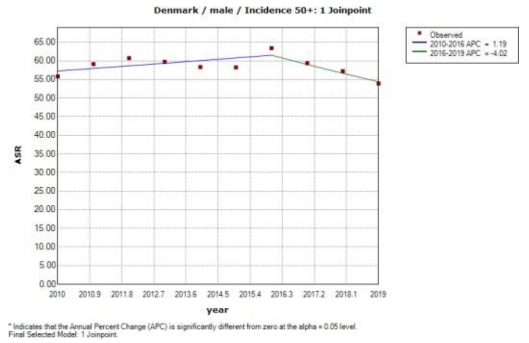

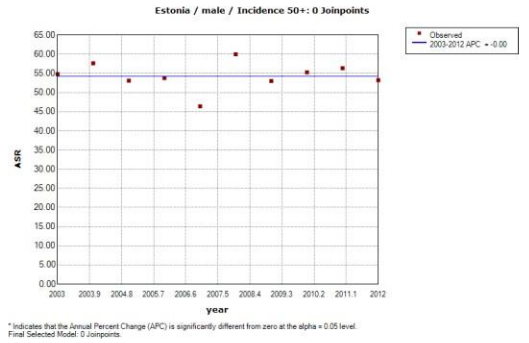

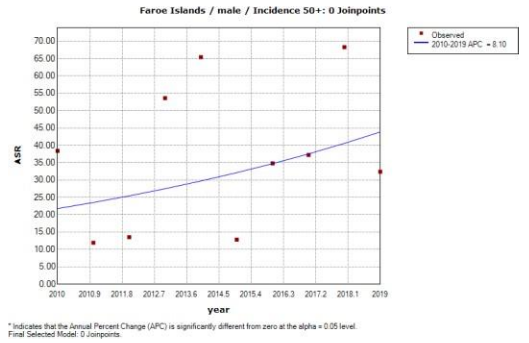

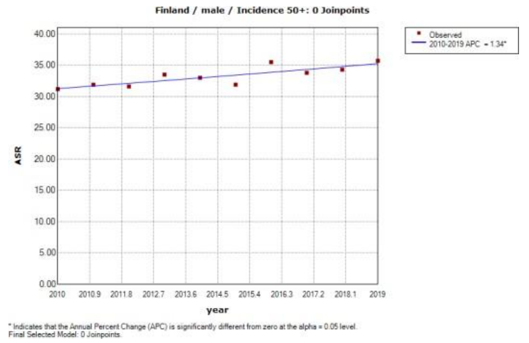

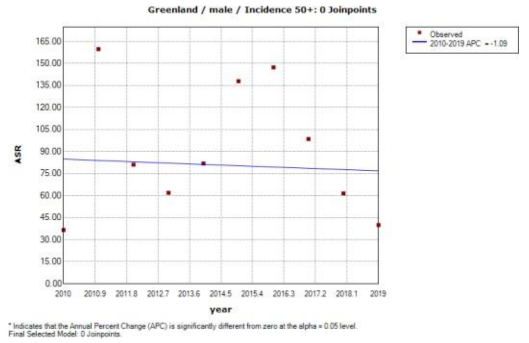

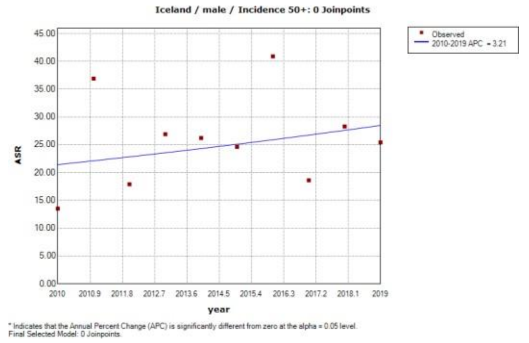

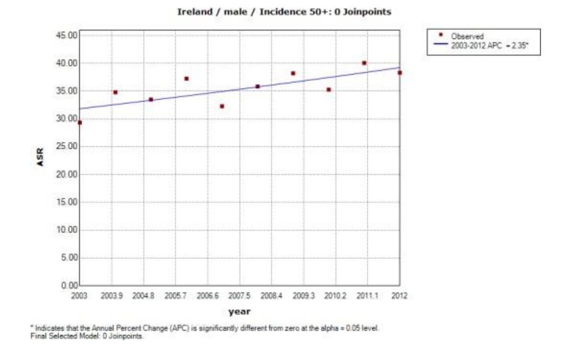

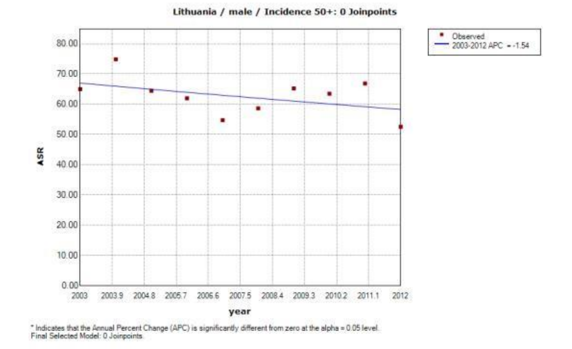

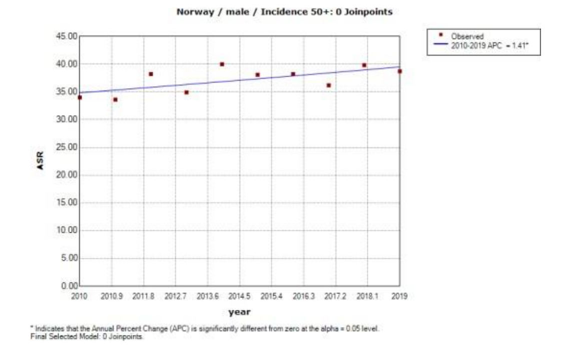

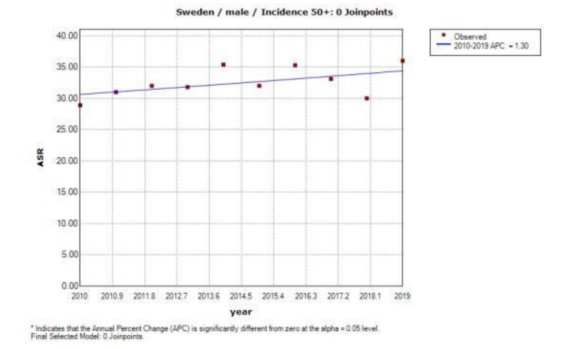

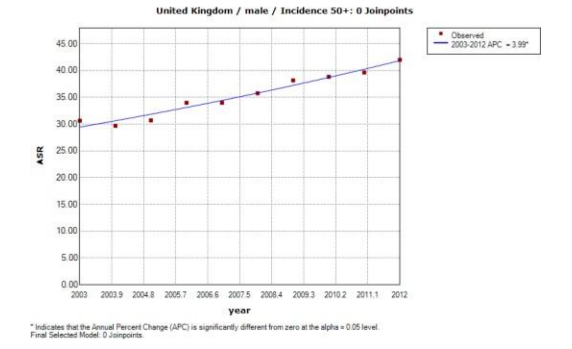

Western Europe

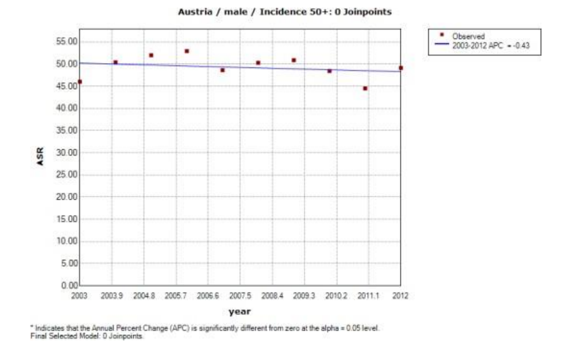

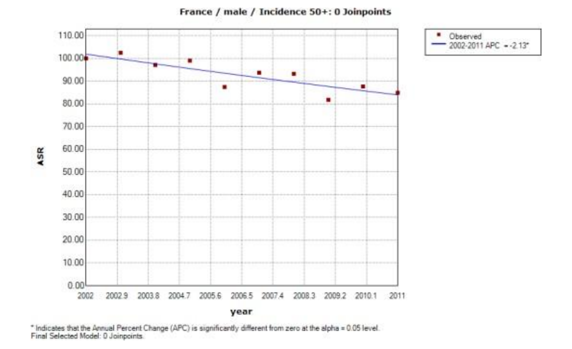

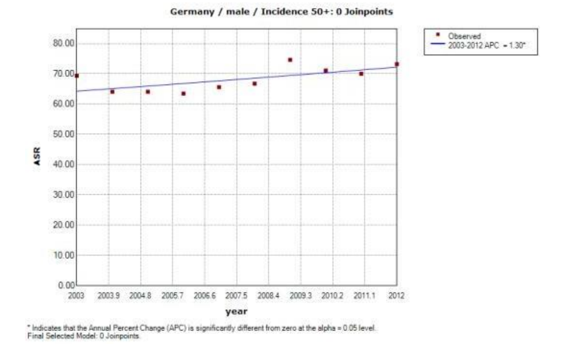

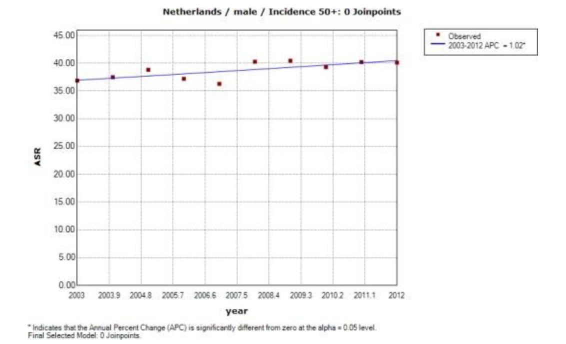

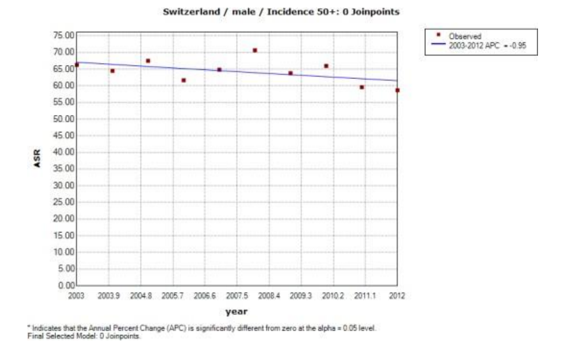

Southern Europe

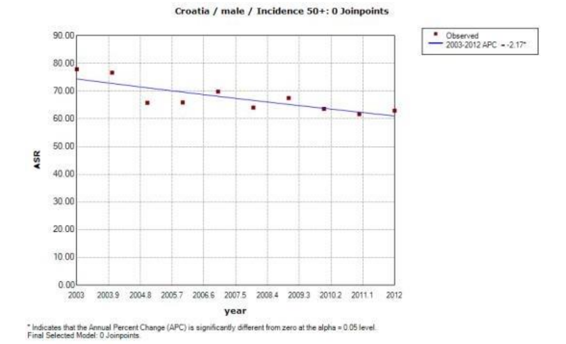

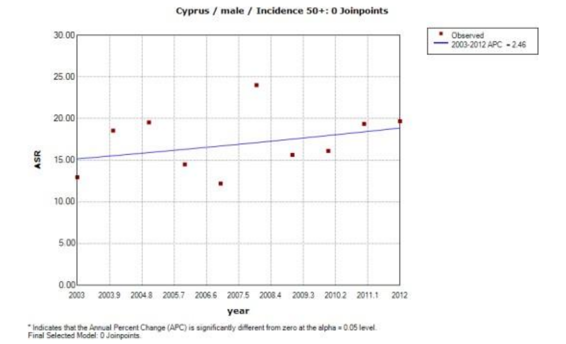

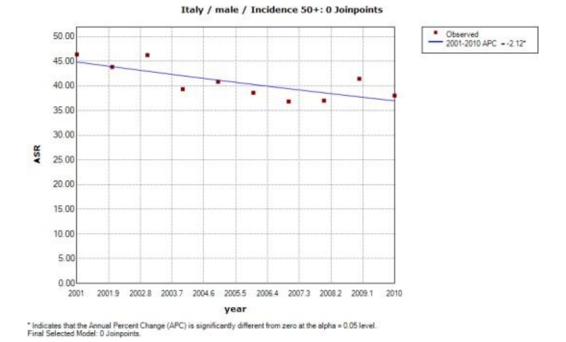

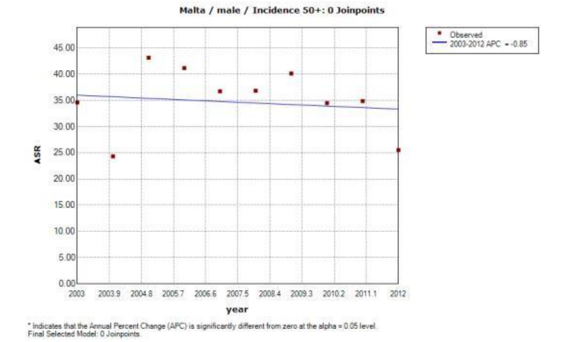

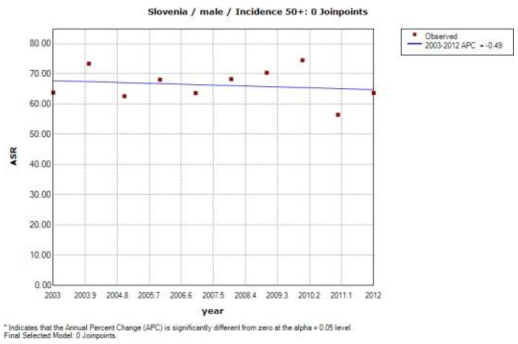

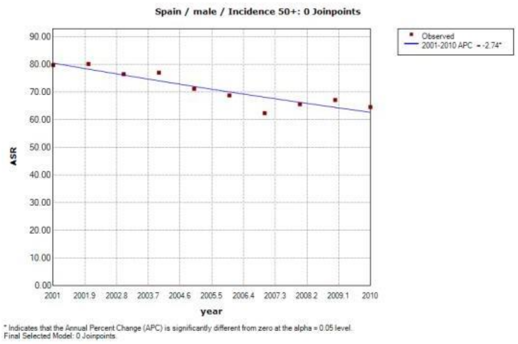

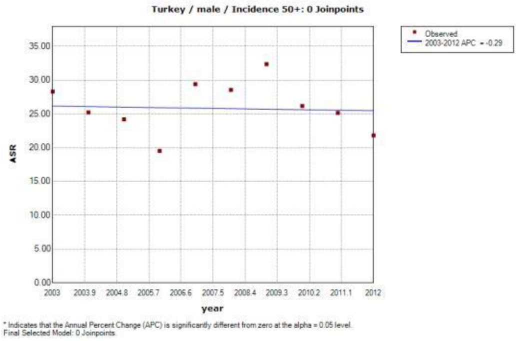

Eastern Europe

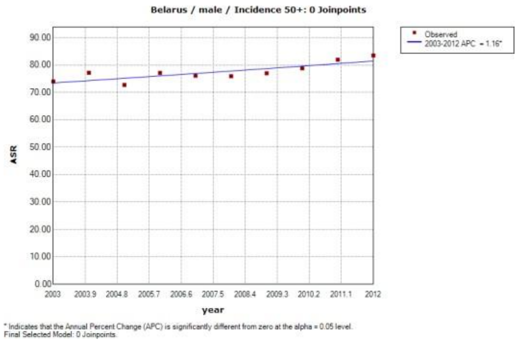

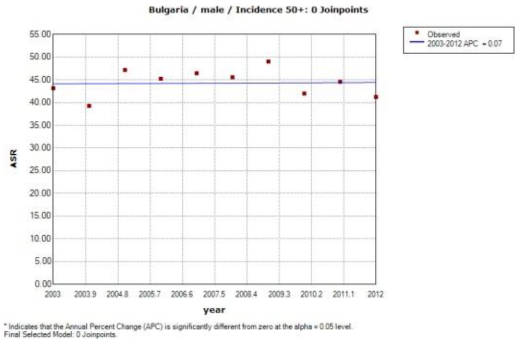

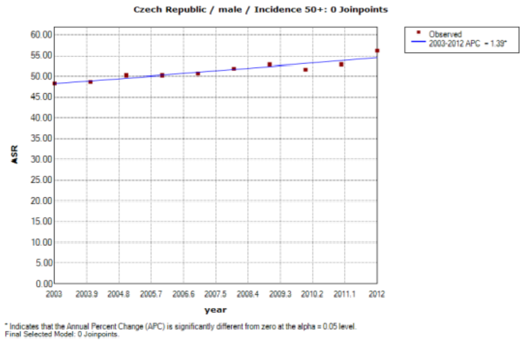

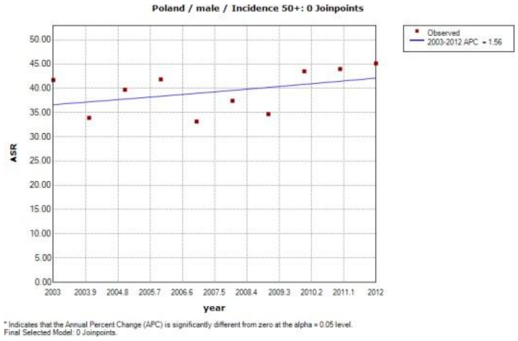

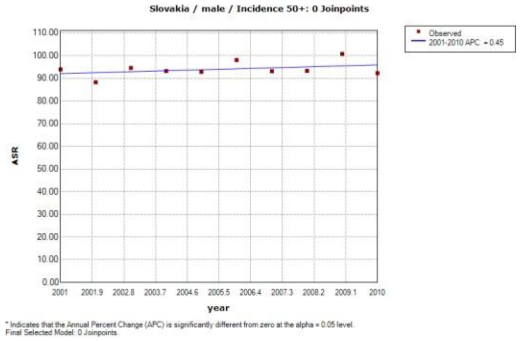
Slovakia / male / Incidence 50+: 0 Joinpoints
Observed
2001-2010 APC = 0.45
110.00
100.00
90.00
80.00
70.00
60.00
50.00
40.00
30.00
20.00
10.00
0.00
ASR
2001
2001.9
2002.8
2003.7
2004.6
2005.5
2006.4
2007.3
2008.2
2009.1
2010
year
* Indicates that the Annual Percent Change (APC) is significantly different from zero at the alpha = 0.05 level.
Final Selected Model: 0 Joinpoints.

Africa

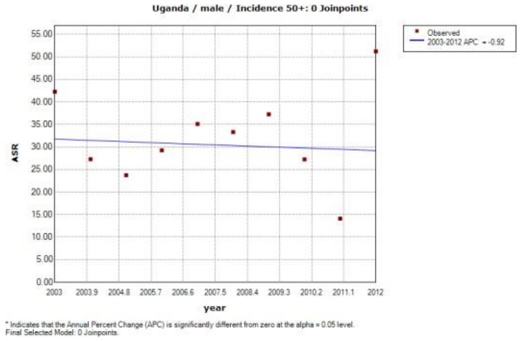
Uganda / male / Incidence 50+: 0 Joinpoints
Observed
2003-2012 APC = -0.92
55.00
50.00
45.00
40.00
35.00
30.00
25.00
20.00
15.00
10.00
5.00
0.00
ASR
2003
2003.9
2004.8
2005.7
2006.6
2007.5
2008.4
2009.3
2010.2
2011.1
2012
year
* Indicates that the Annual Percent Change (APC) is significantly different from zero at the alpha = 0.05 level.
Final Selected Model: 0 Joinpoints.

h.) Incidence female above 50 years old

Asia

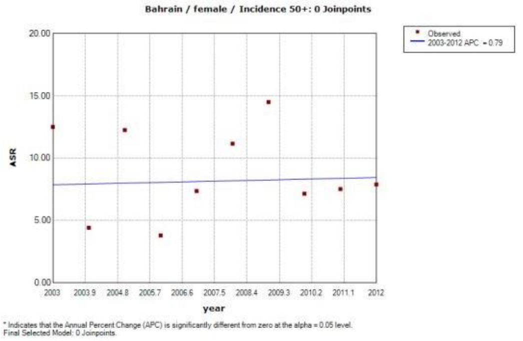

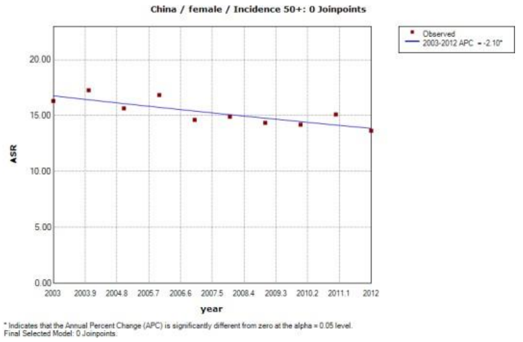

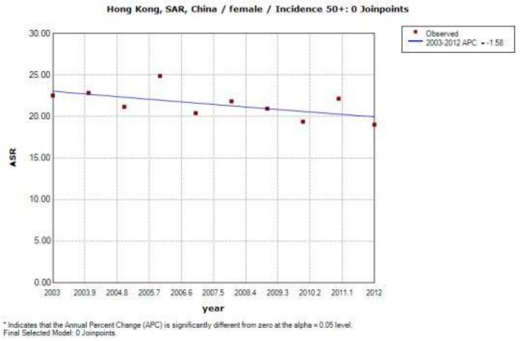

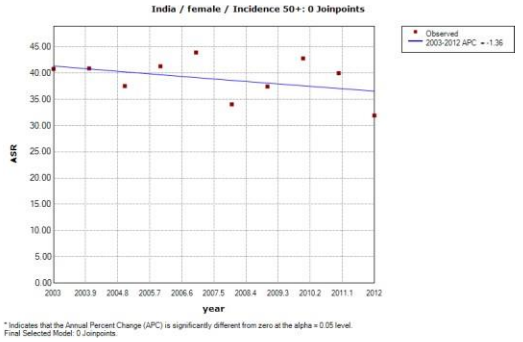

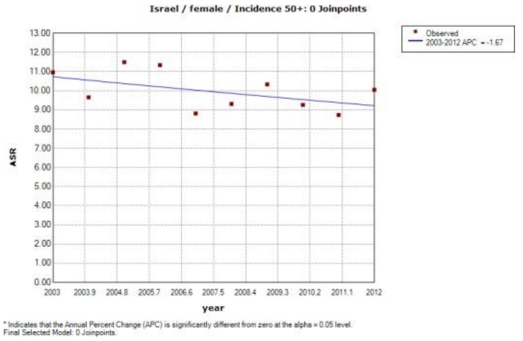

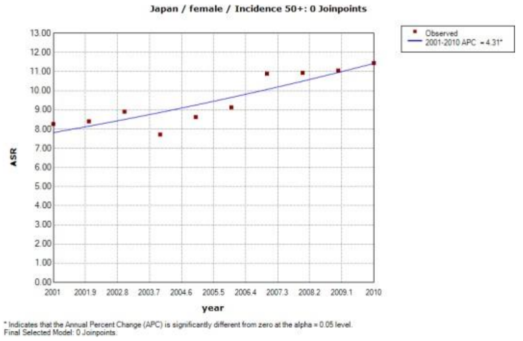

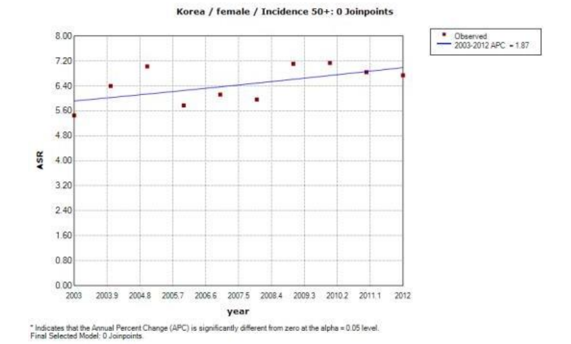
Korea / female / Incidence 50+: 0 Joinpoints
Observed
2003-2012 APC = 1.87
ASR
year
* Indicates that the Annual Percent Change (APC) is significantly different from zero at the alpha = 0.05 level.
Final Selected Model: 0 Joinpoints.

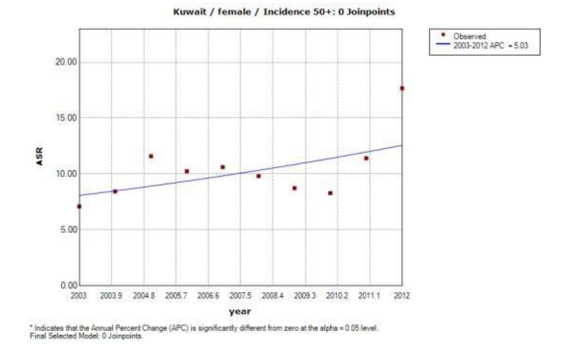
Kuwait / female / Incidence 50+: 0 Joinpoints
Observed
2003-2012 APC = 5.03
ASR
year
* Indicates that the Annual Percent Change (APC) is significantly different from zero at the alpha = 0.05 level.
Final Selected Model: 0 Joinpoints.

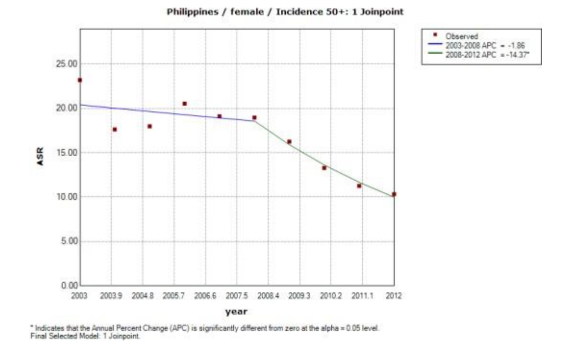
Philippines / female / Incidence 50+: 1 Joinpoint
Observed
2003-2008 APC = -1.86
2008-2012 APC = -14.37*
ASR
year
* Indicates that the Annual Percent Change (APC) is significantly different from zero at the alpha = 0.05 level.
Final Selected Model: 1 Joinpoint.

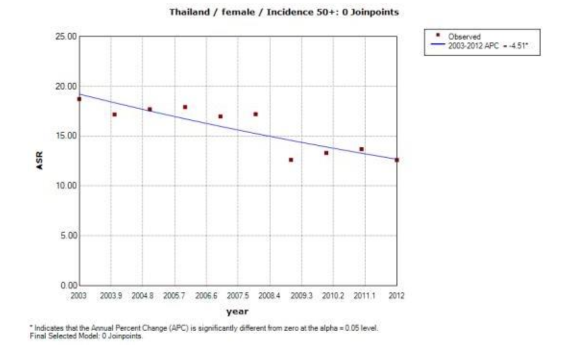
Thailand / female / Incidence 50+: 0 Joinpoints
Observed
2003-2012 APC = -4.51*
ASR
year
* Indicates that the Annual Percent Change (APC) is significantly different from zero at the alpha = 0.05 level.
Final Selected Model: 0 Joinpoints.

Oceania

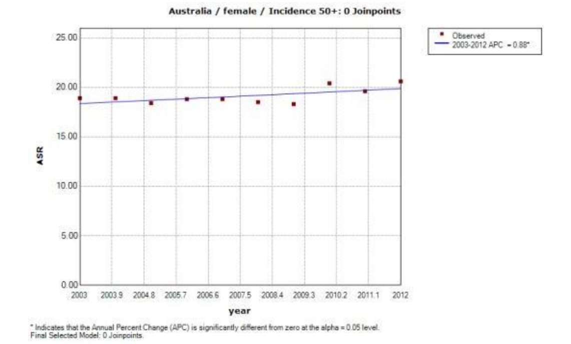
Australia / female / Incidence 50+: 0 Joinpoints
Observed
2003-2012 APC = 0.88*
ASR
year
* Indicates that the Annual Percent Change (APC) is significantly different from zero at the alpha = 0.05 level.
Final Selected Model: 0 Joinpoints.

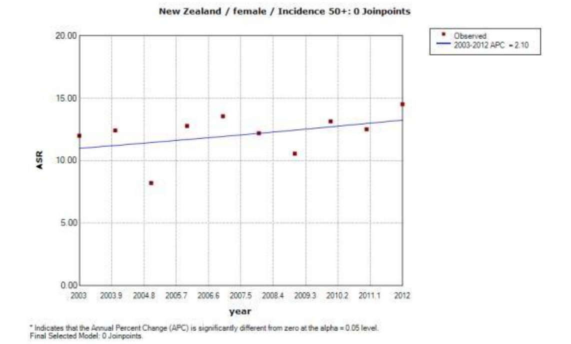
New Zealand / female / Incidence 50+: 0 Joinpoints
Observed
2003-2012 APC = 2.10
ASR
year
* Indicates that the Annual Percent Change (APC) is significantly different from zero at the alpha = 0.05 level.
Final Selected Model: 0 Joinpoints.

Northern America

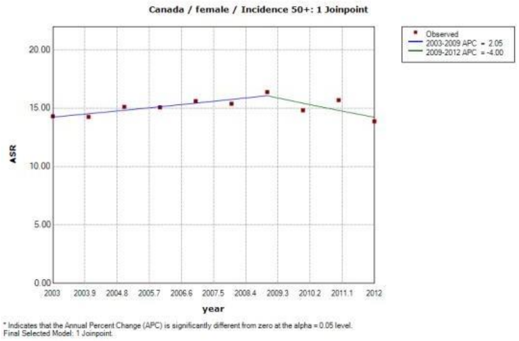

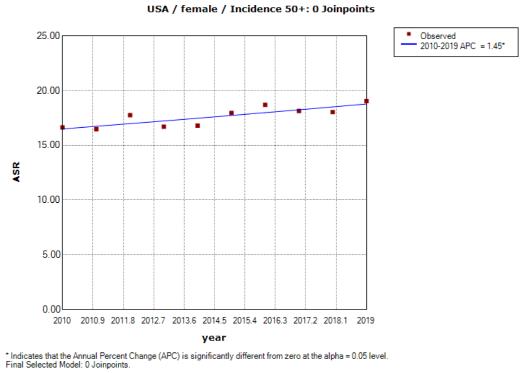

Southern America

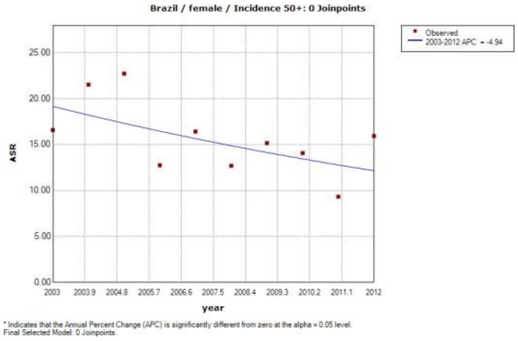

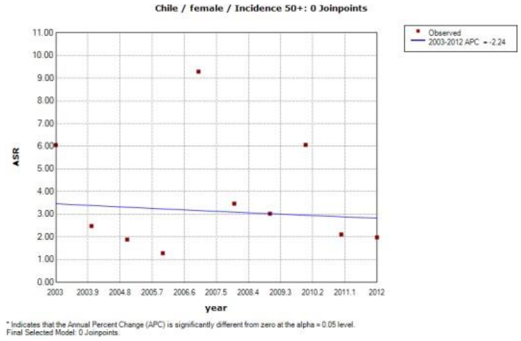

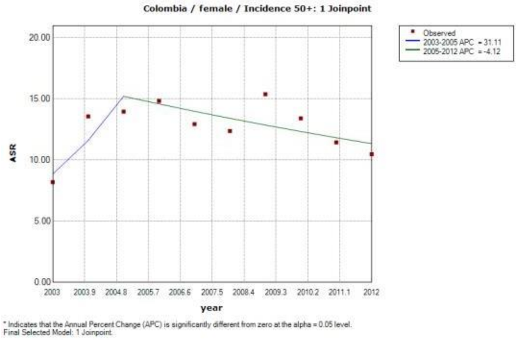

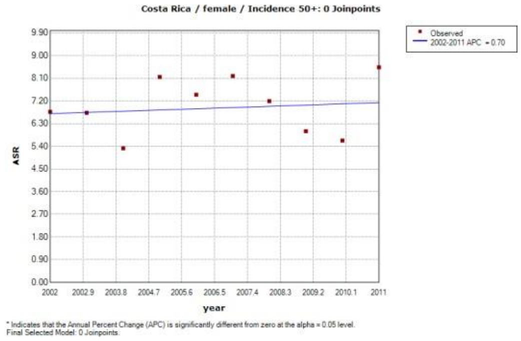

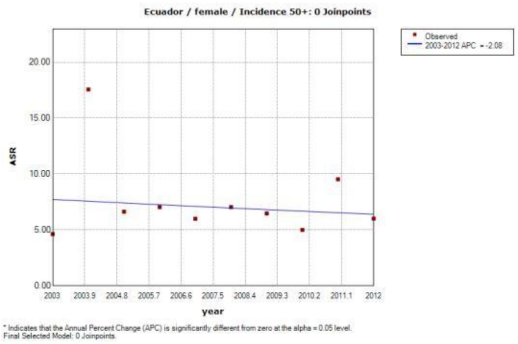

Northern Europe

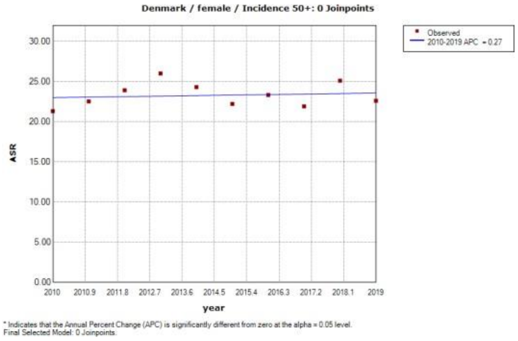

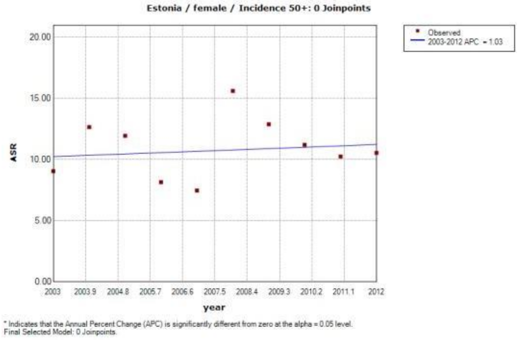

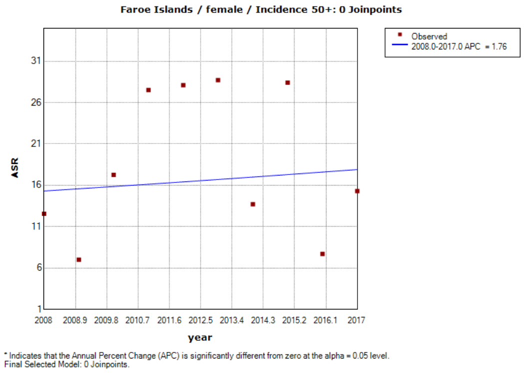

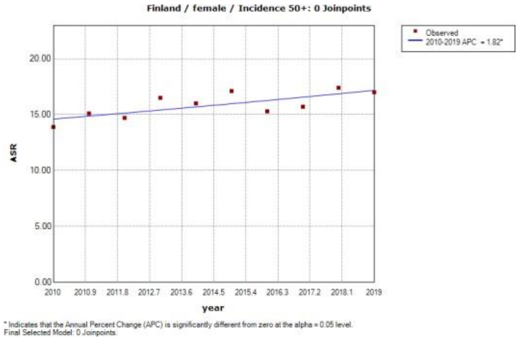

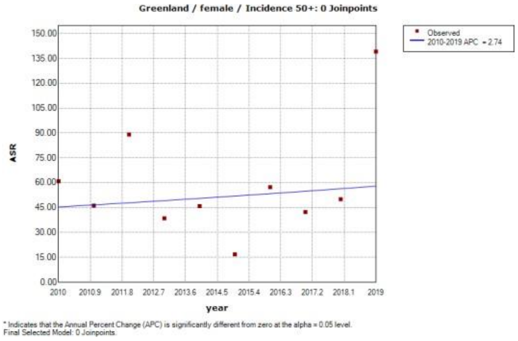

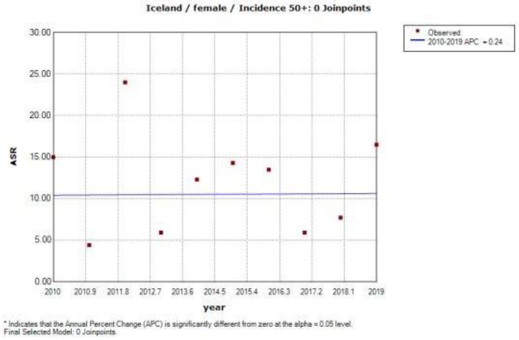

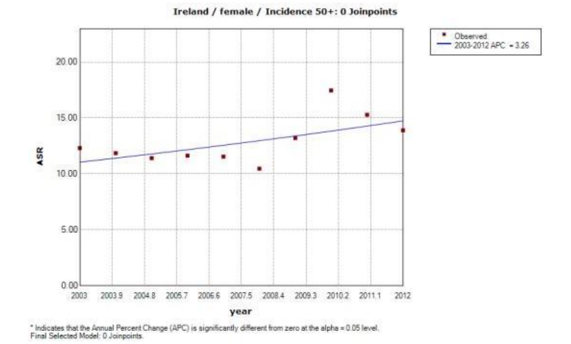

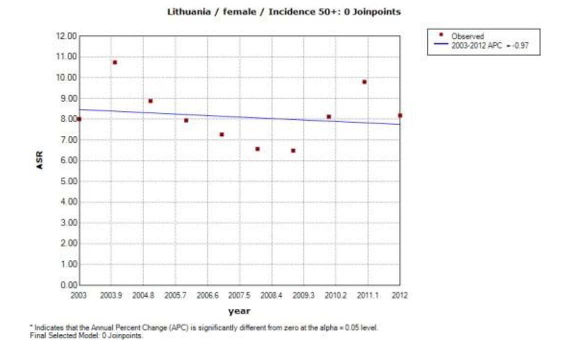

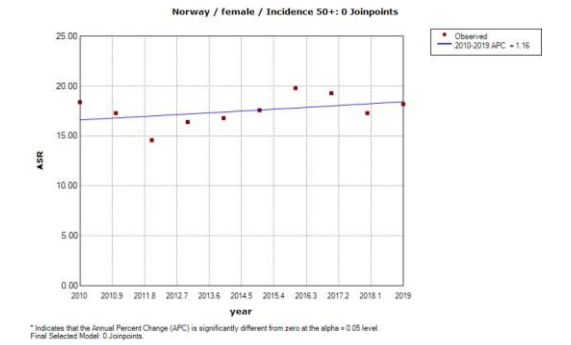

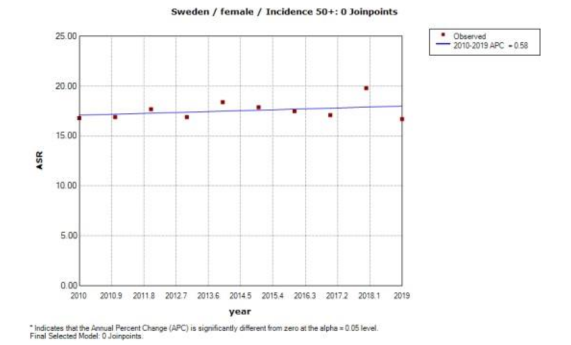

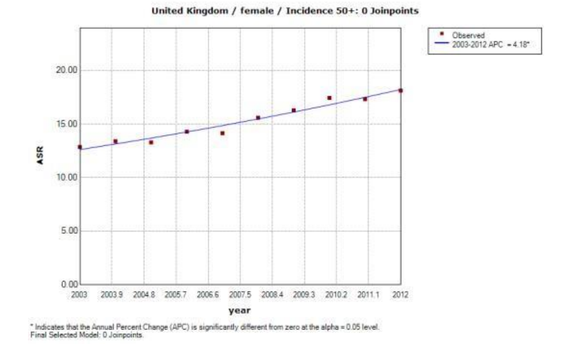

Western Europe

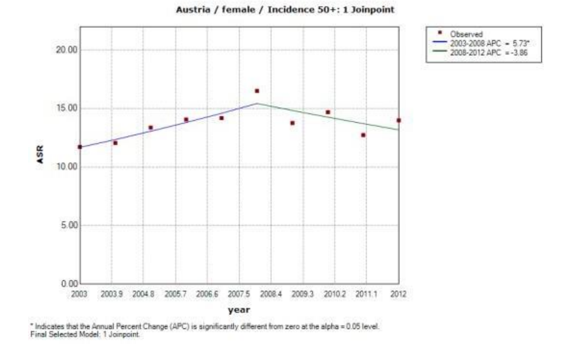

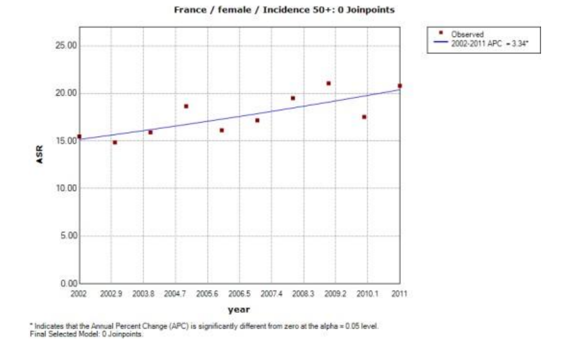

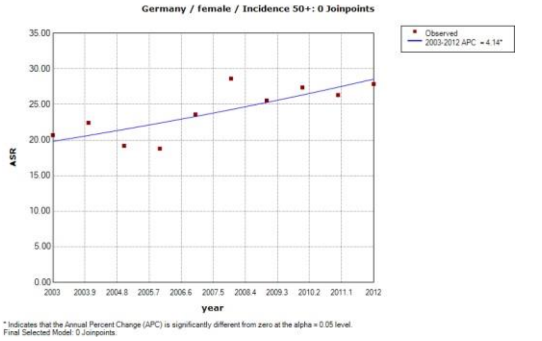

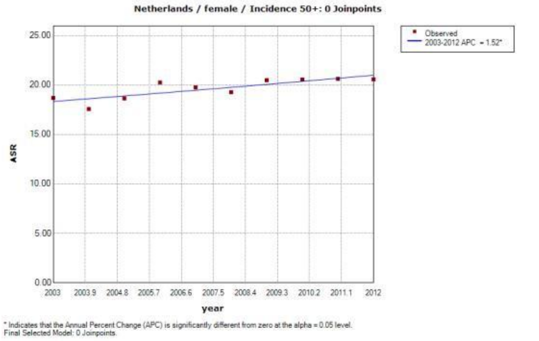

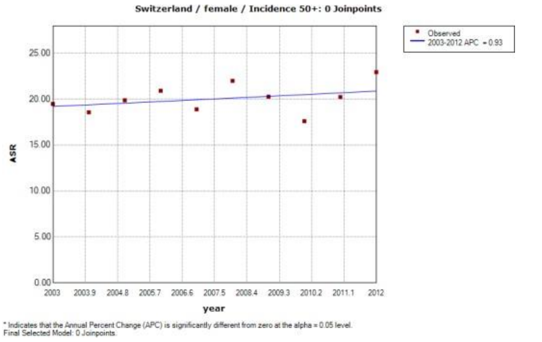

Southern Europe

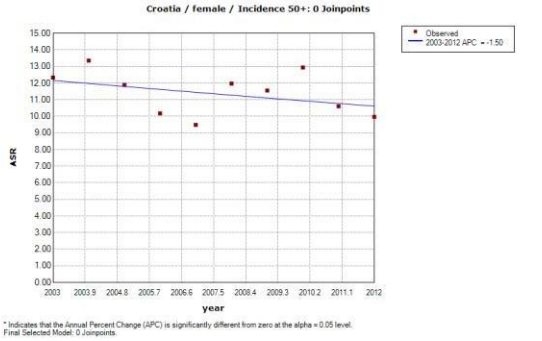

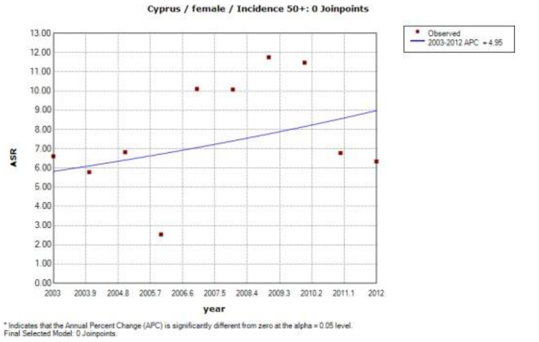

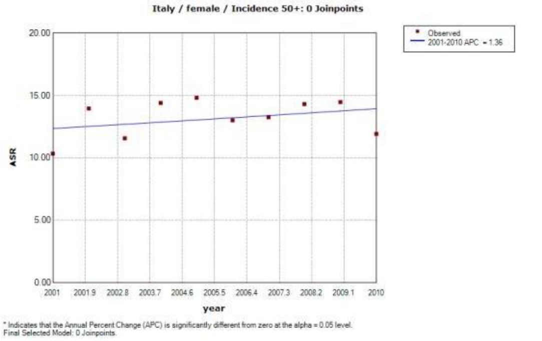

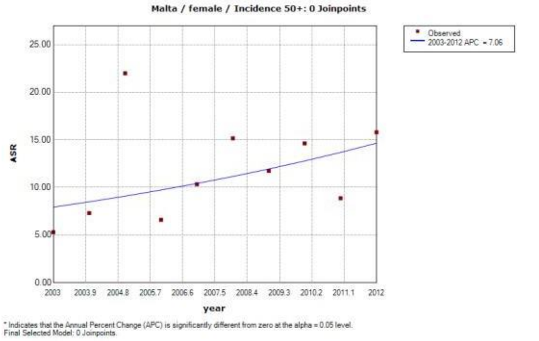

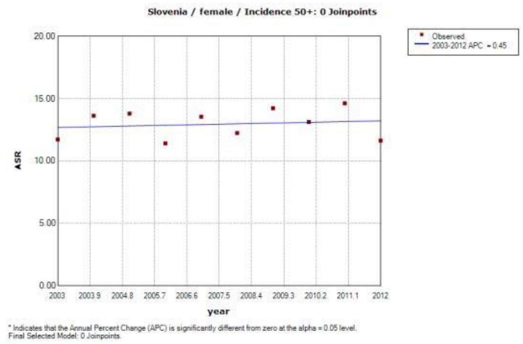

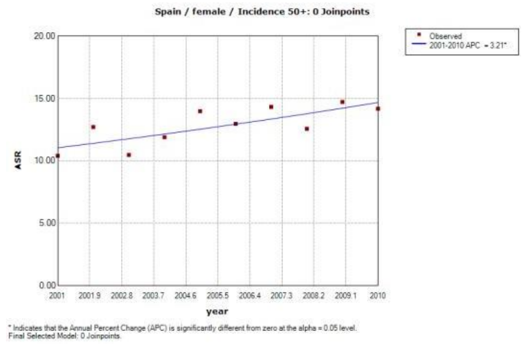

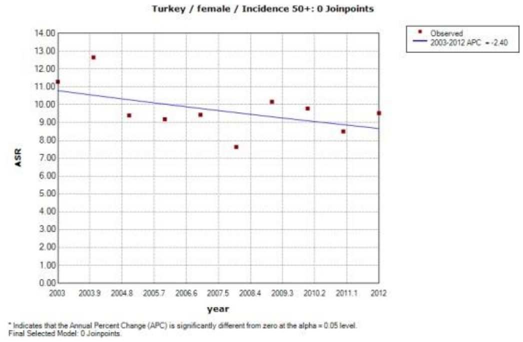

Eastern Europe

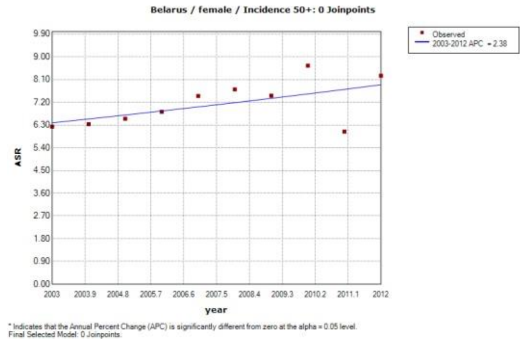

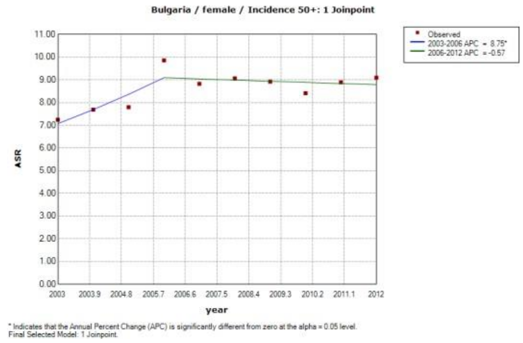

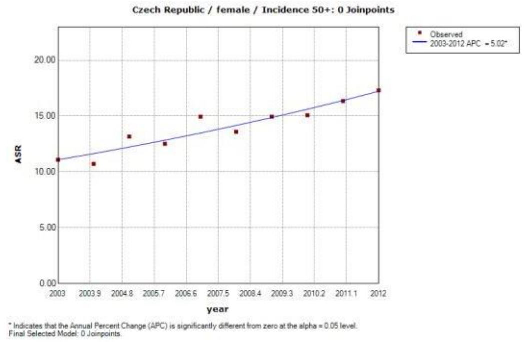

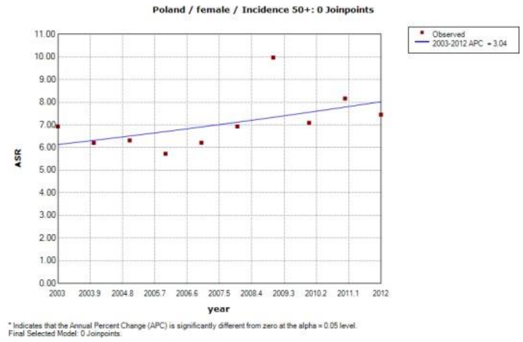

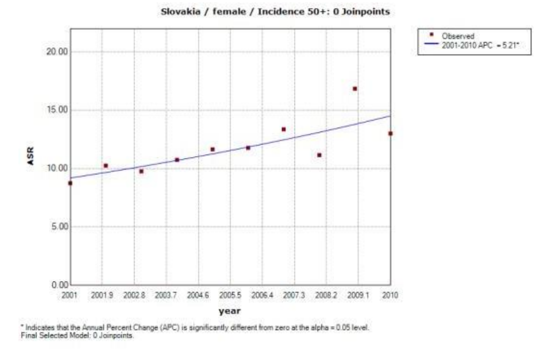
Slovakia / female / Incidence 50+: 0 Joinpoints
Observed
2001-2010 APC = 5.21*
ASR
year
* Indicates that the Annual Percent Change (APC) is significantly different from zero at the alpha = 0.05 level.
Final Selected Model: 0 Joinpoints.

Africa

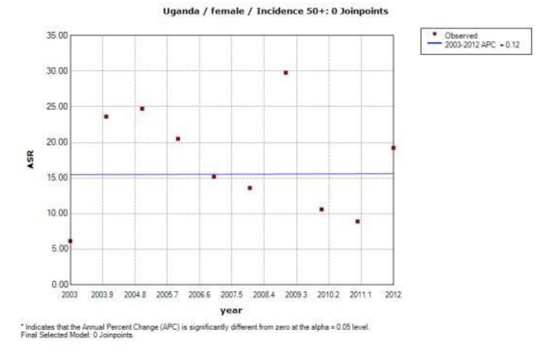
Uganda / female / Incidence 50+: 0 Joinpoints
Observed
2003-2012 APC = 0.12
ASR
year
* Indicates that the Annual Percent Change (APC) is significantly different from zero at the alpha = 0.05 level.
Final Selected Model: 0 Joinpoints.

i.) Mortality male all ages

Asia

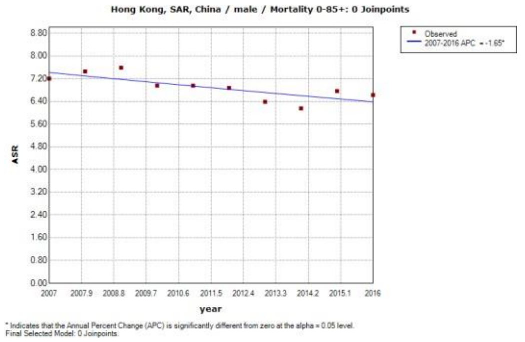

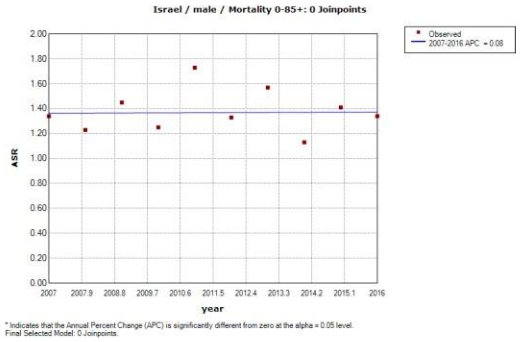

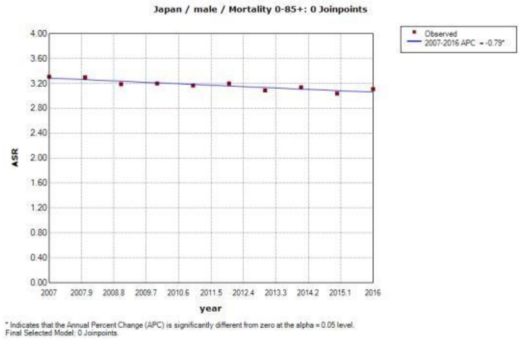

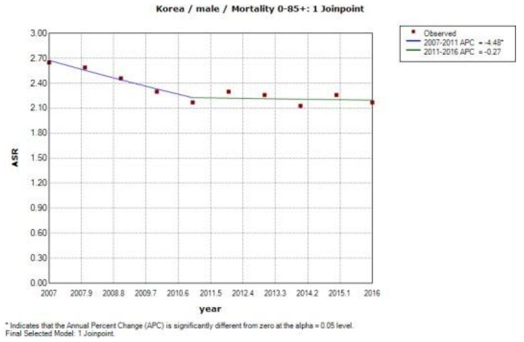

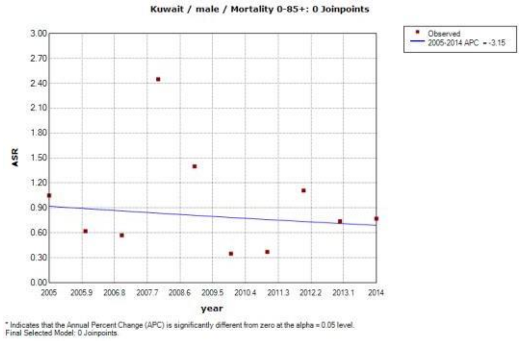

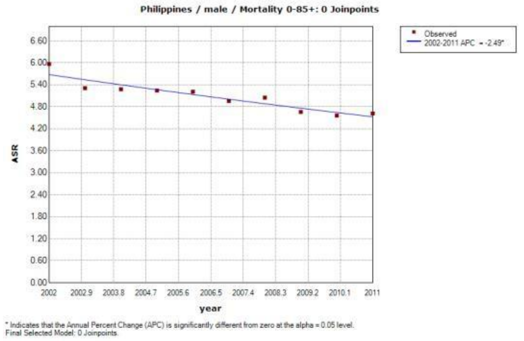

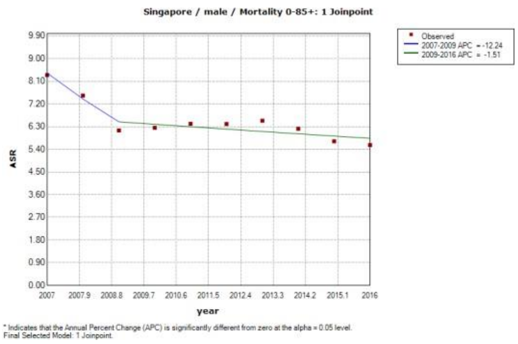

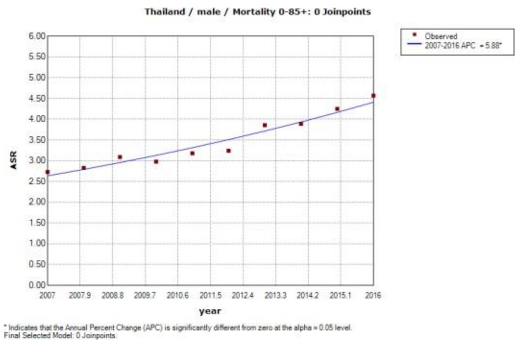

Oceania

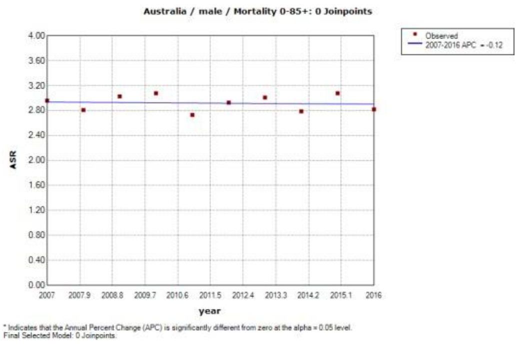

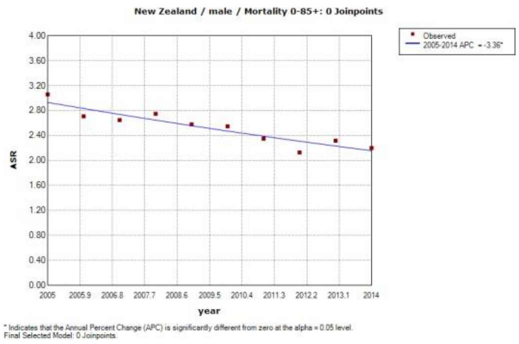

Northern America

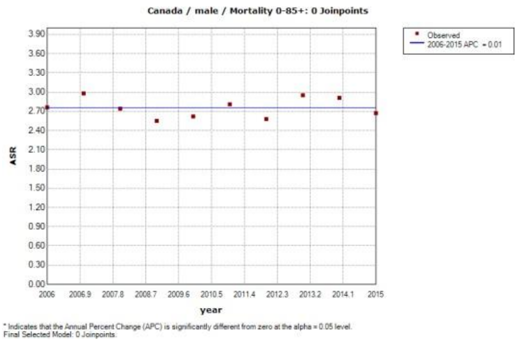

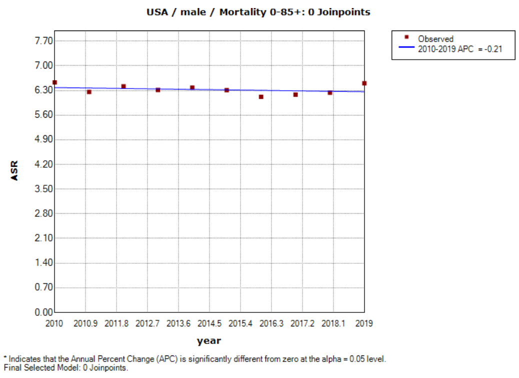

Southern America

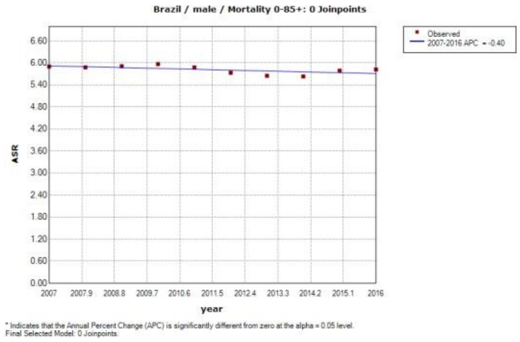

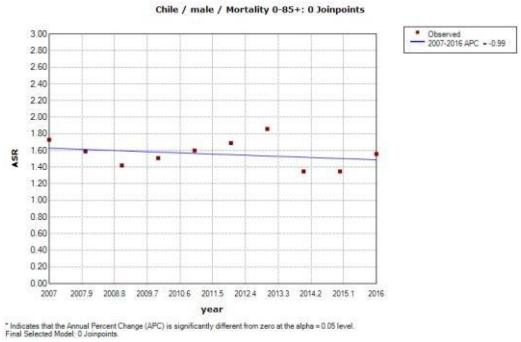

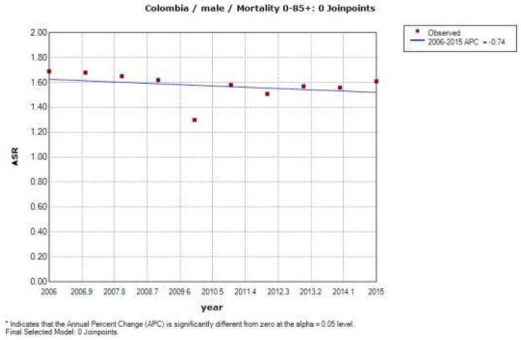

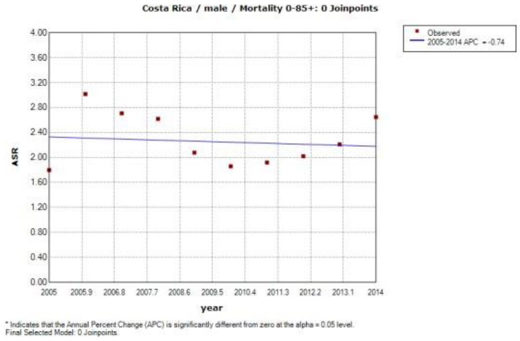

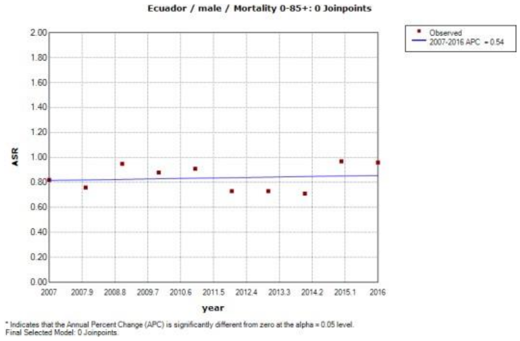

Northern Europe

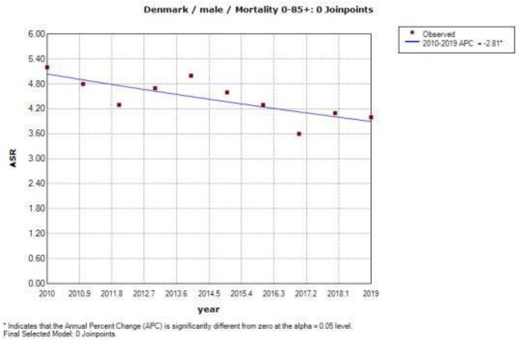

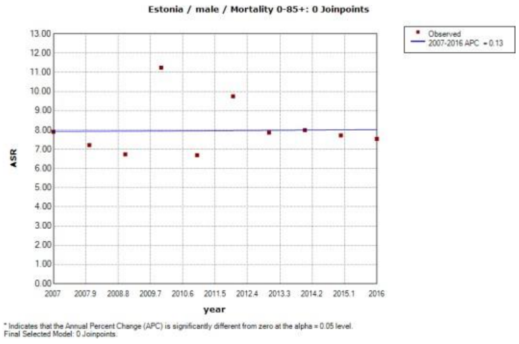

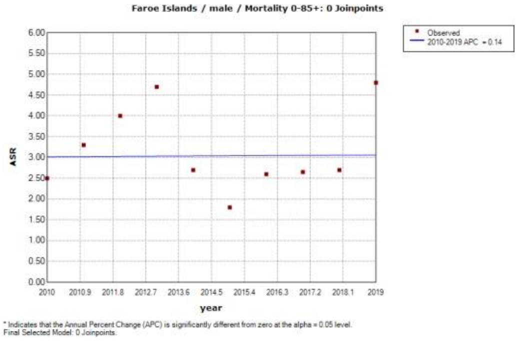

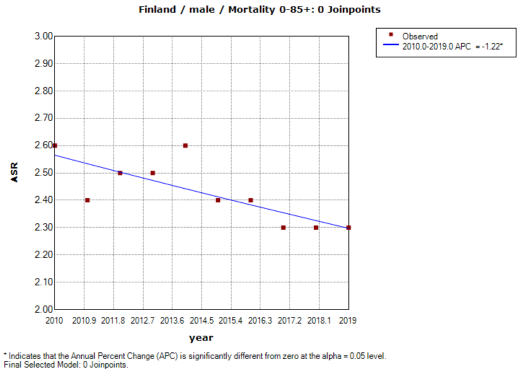

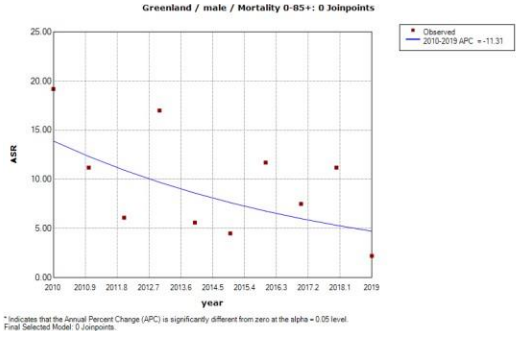

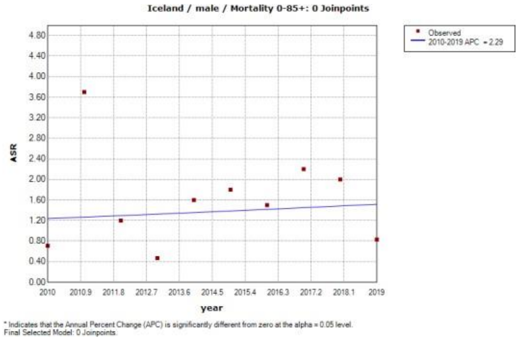

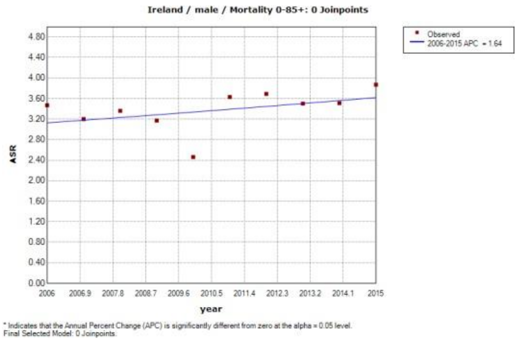

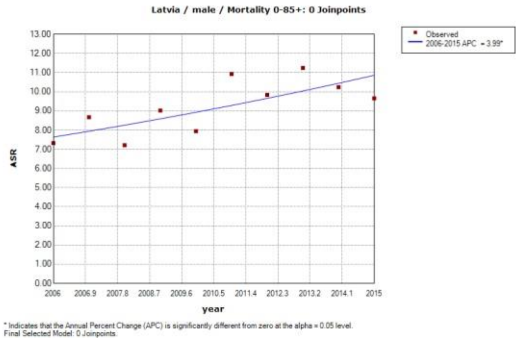

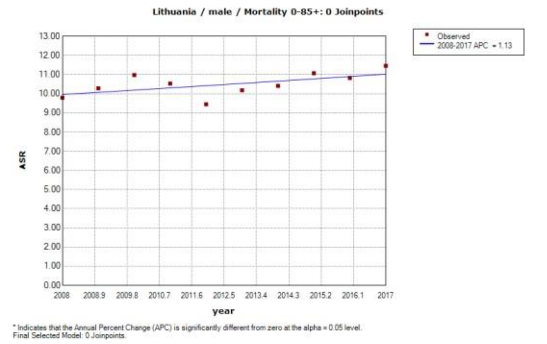

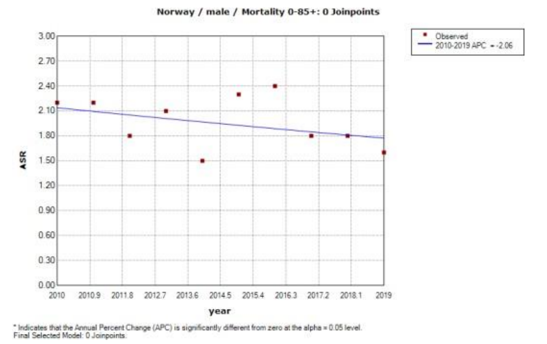

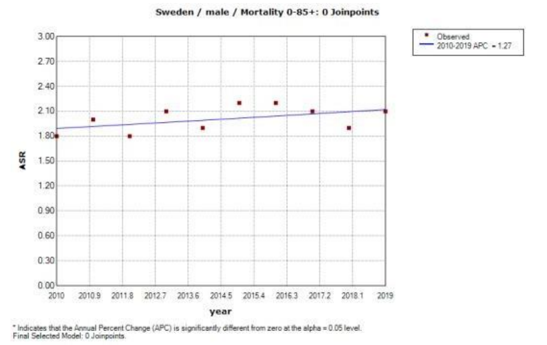

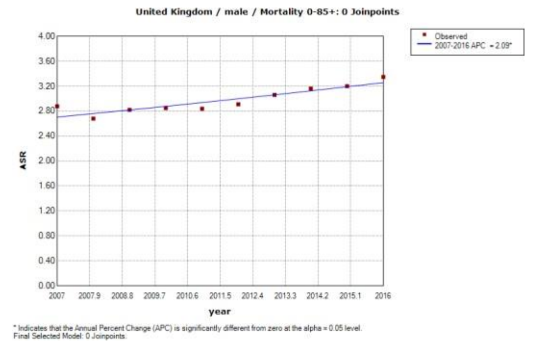

Western Europe

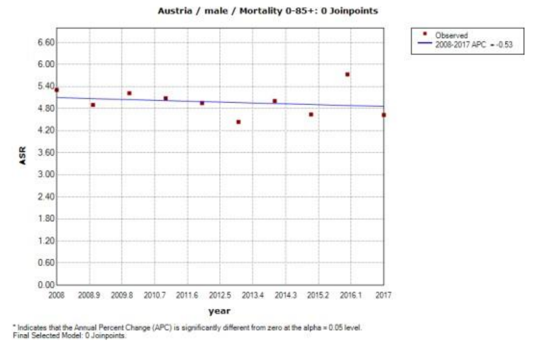

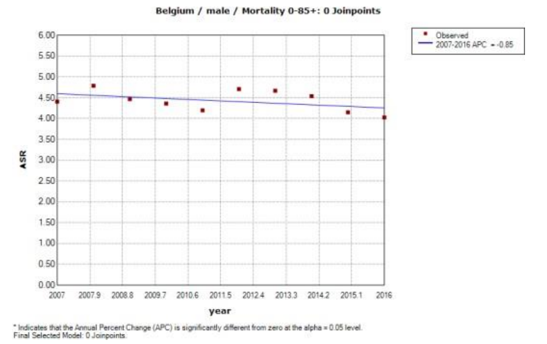

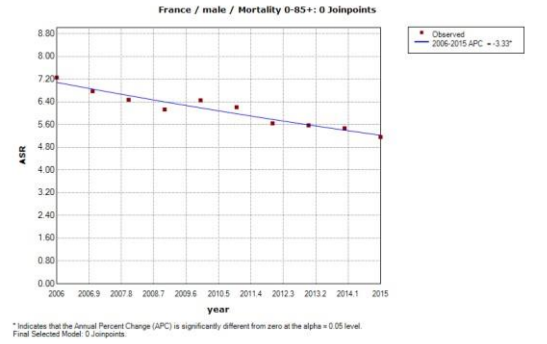

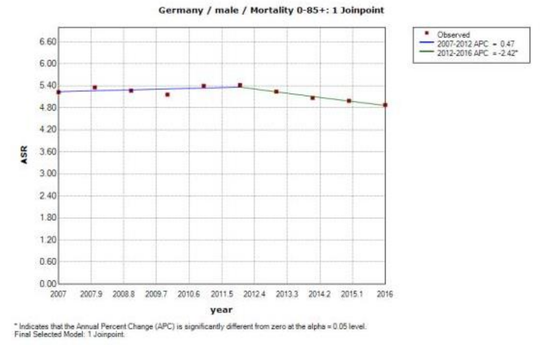

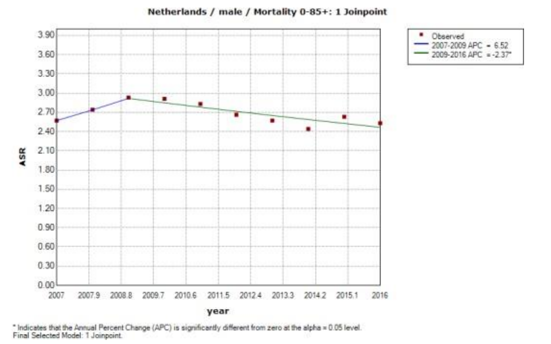

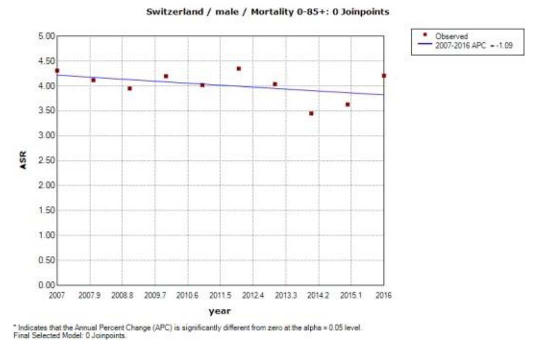

Southern Europe

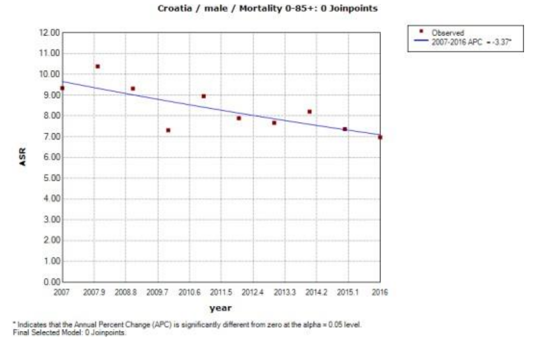

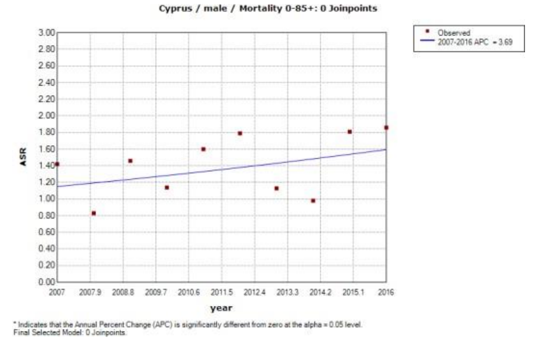

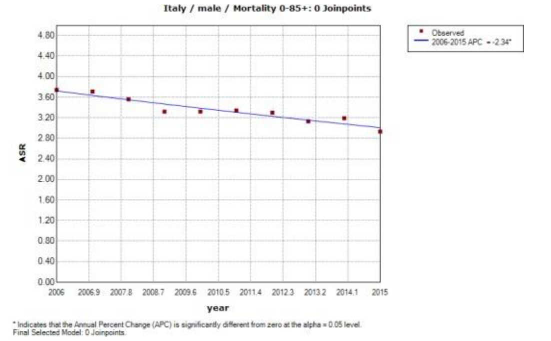

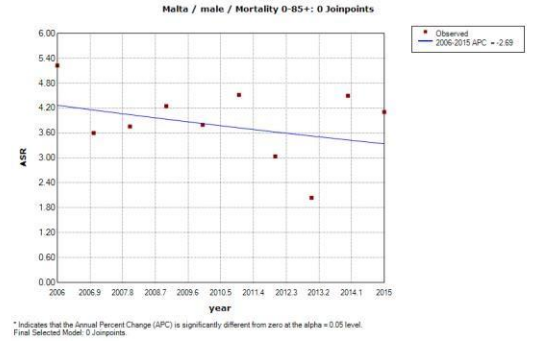

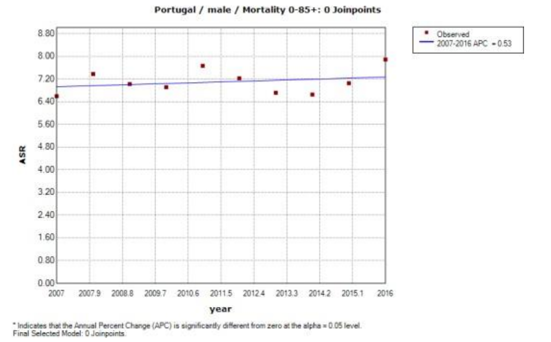

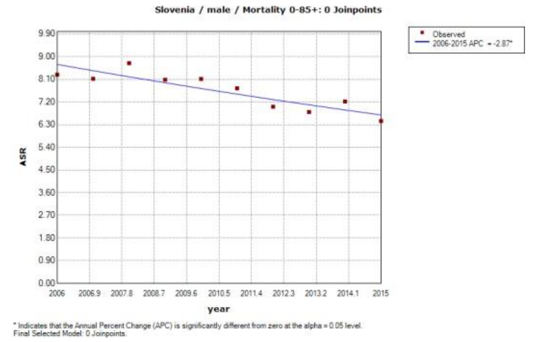

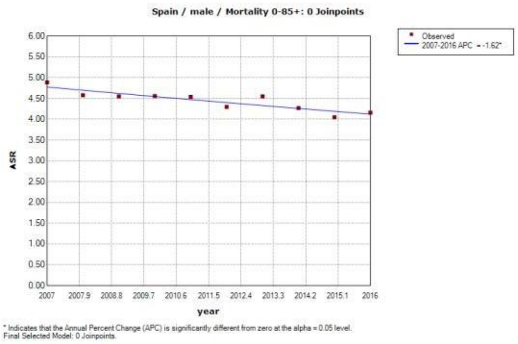

Eastern Europe

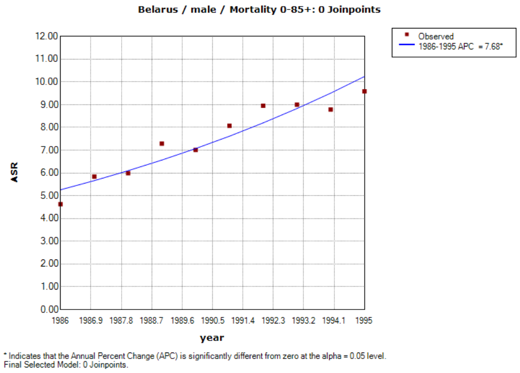

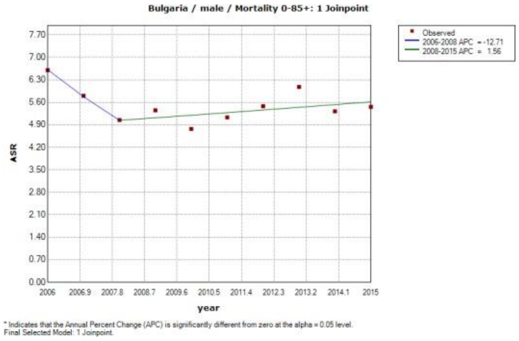

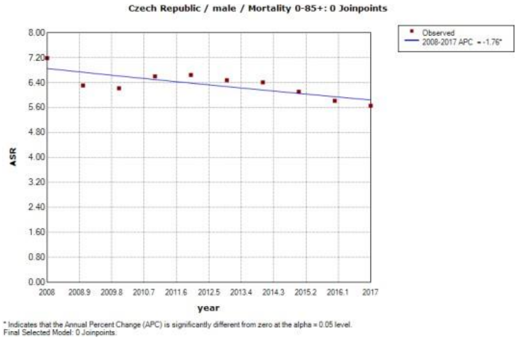

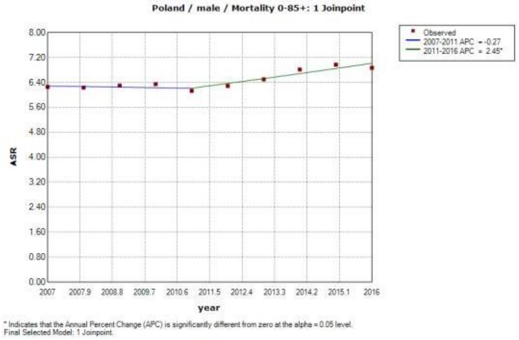

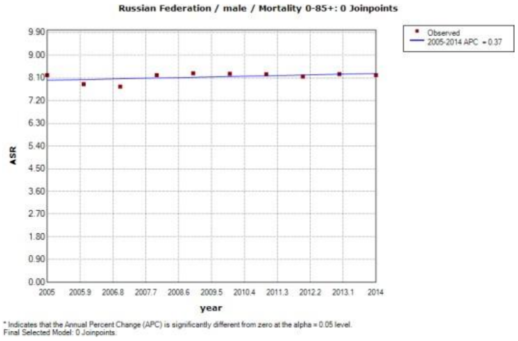

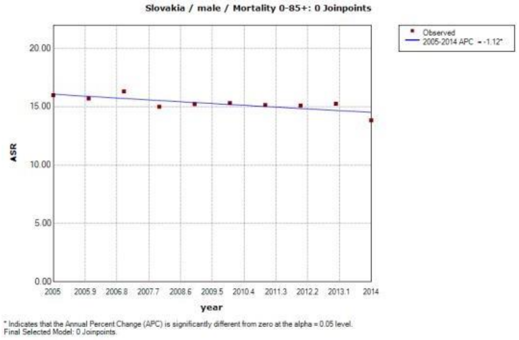

j.) Mortality female all ages

Asia

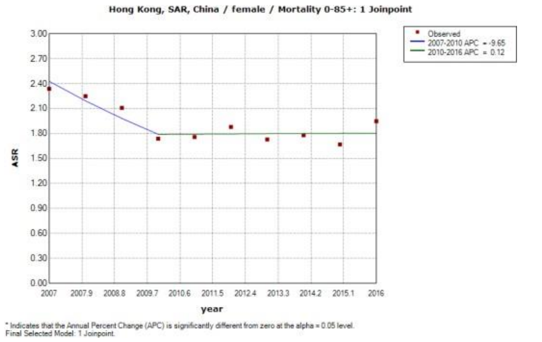

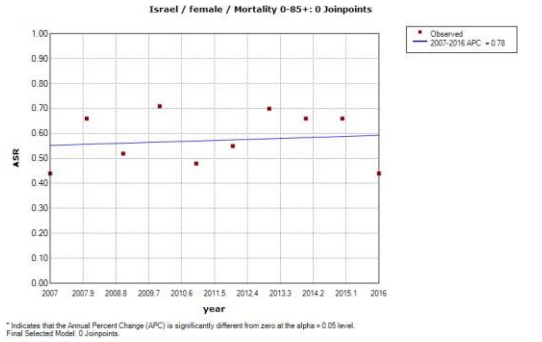

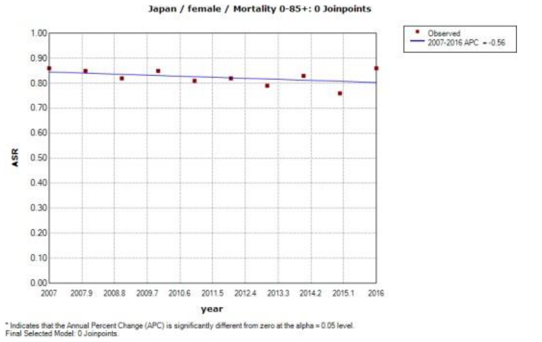

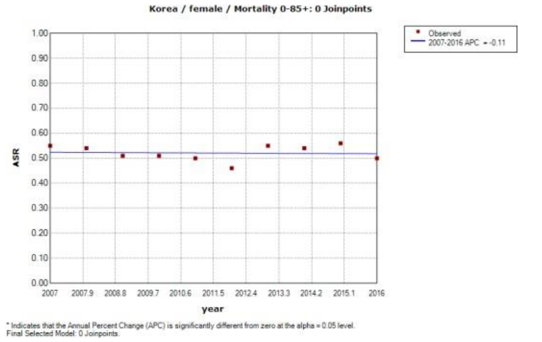

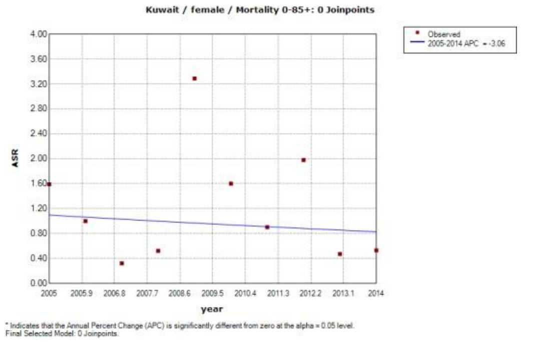

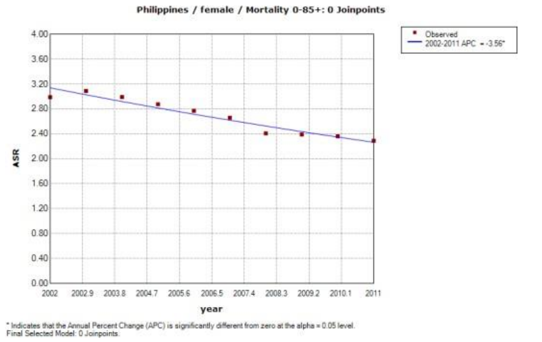

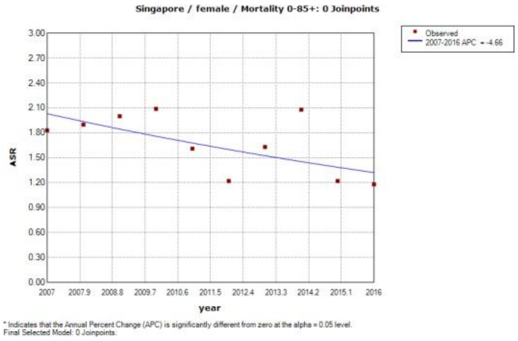

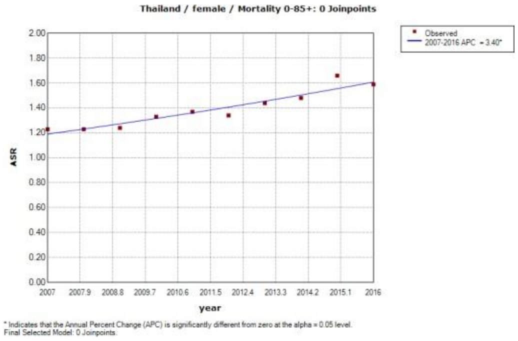

Oceania

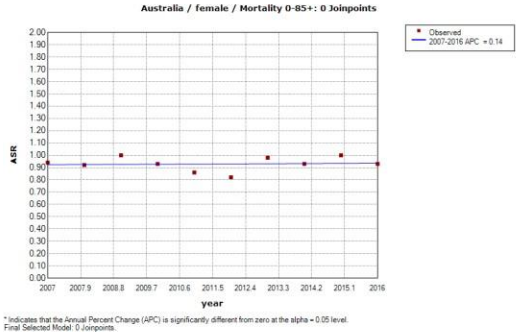

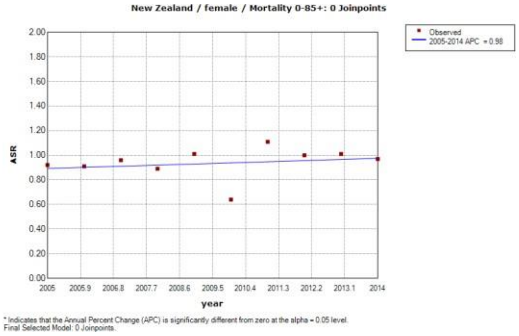

Northern America

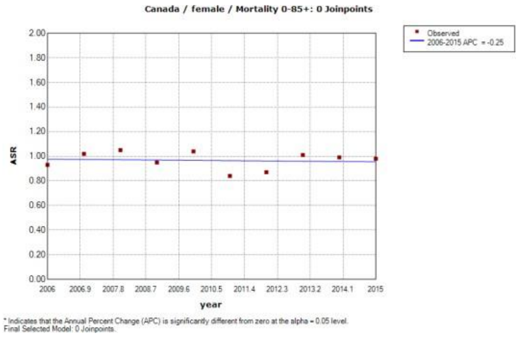

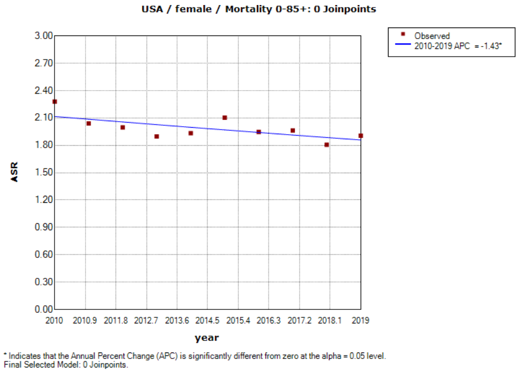

Southern America

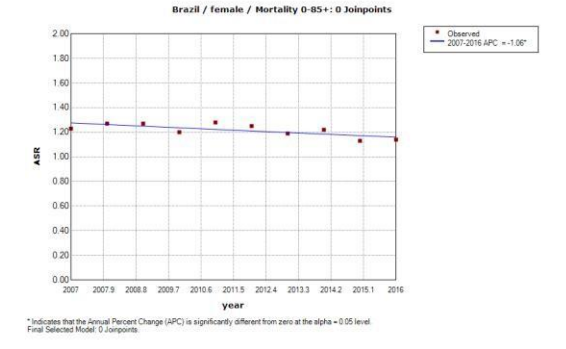

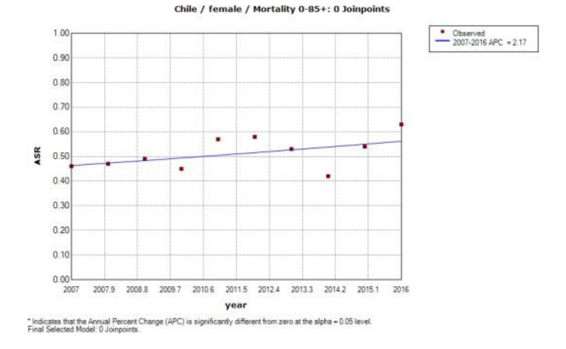

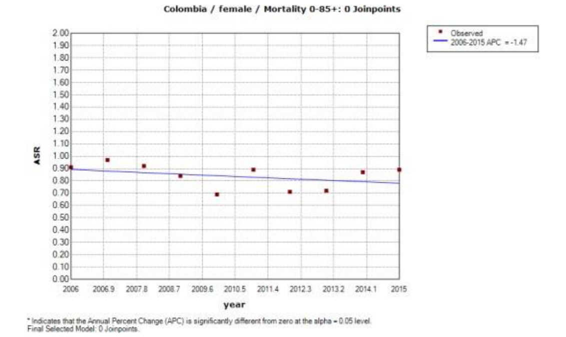

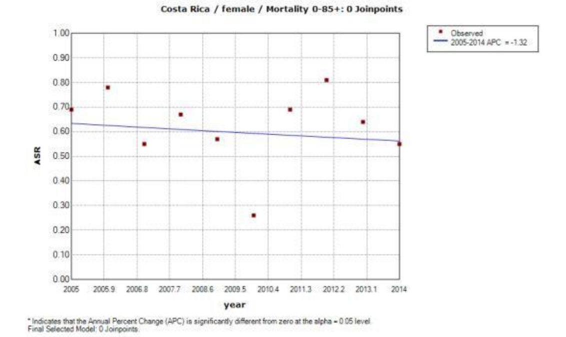

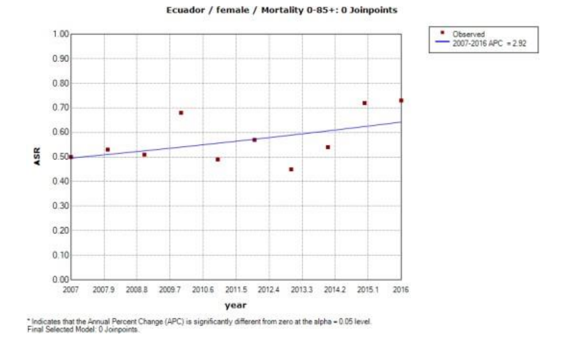

Northern Europe

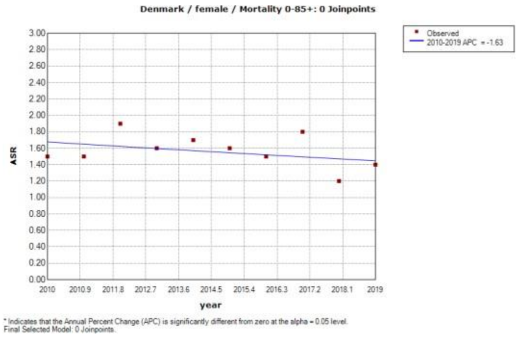

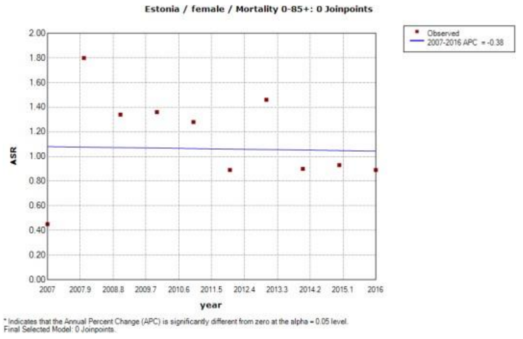

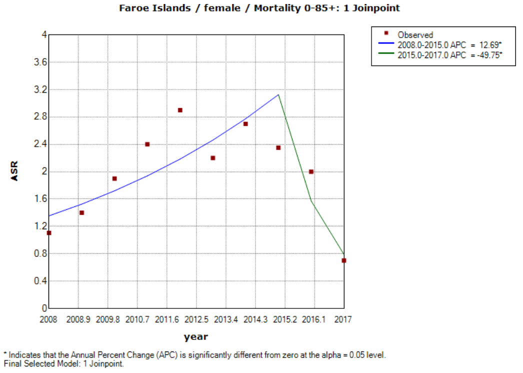

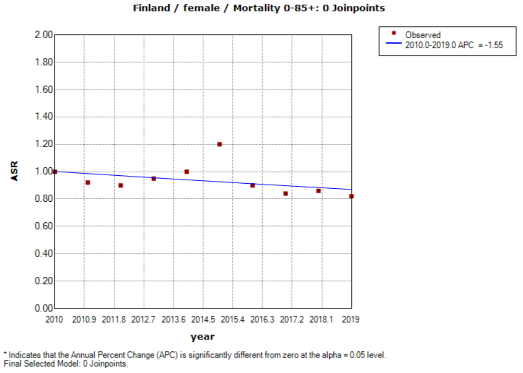

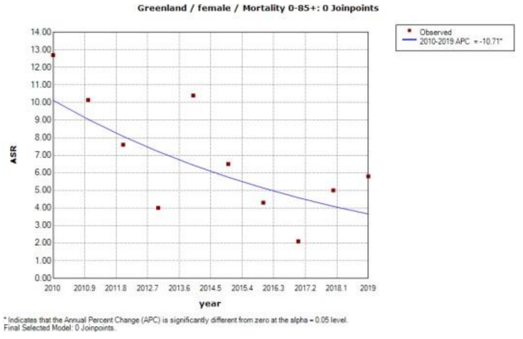

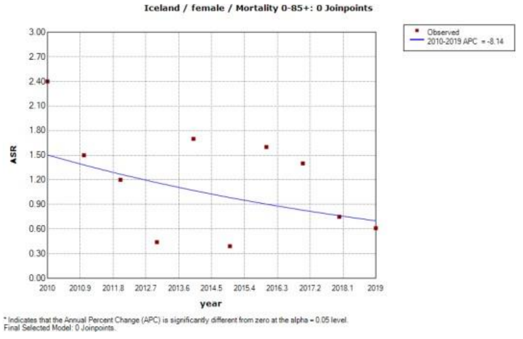

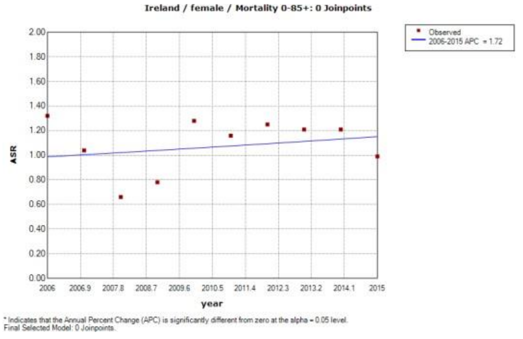

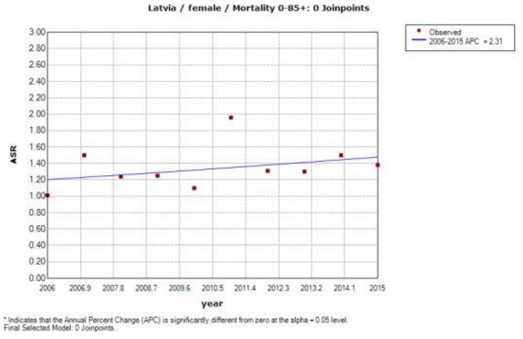

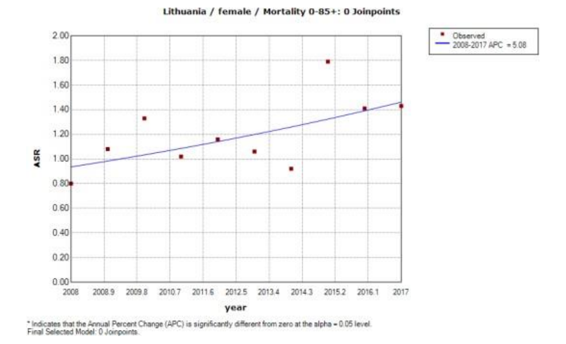

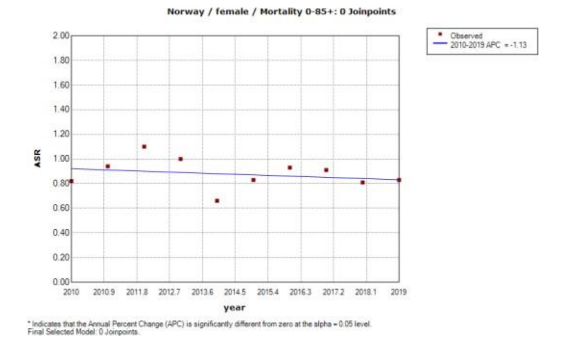

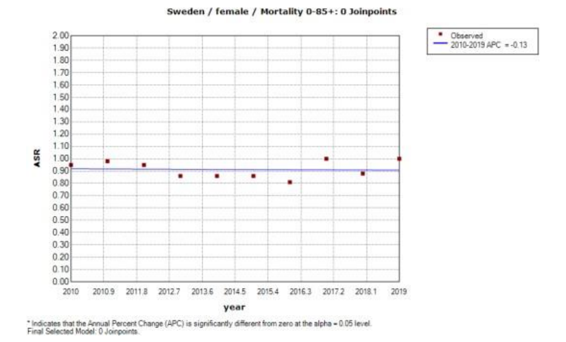

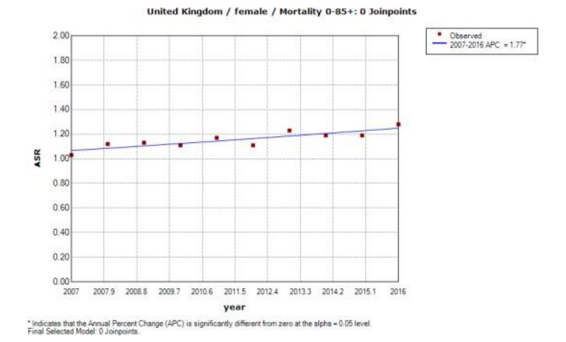

Western Europe

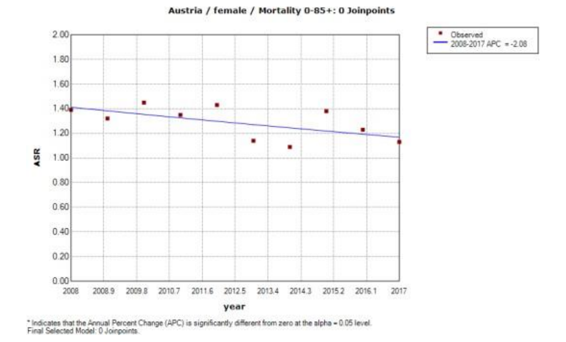

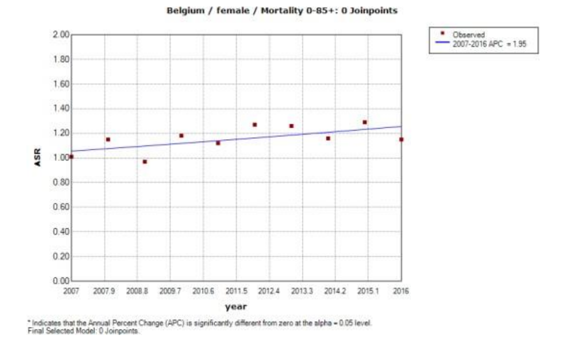

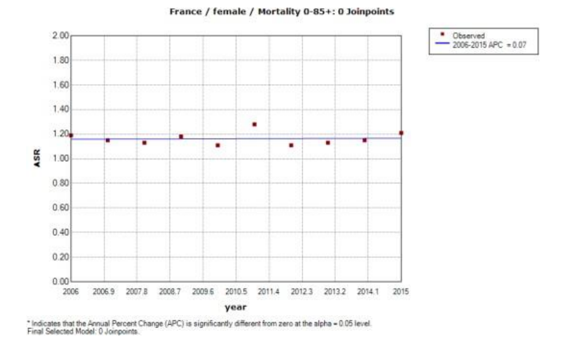

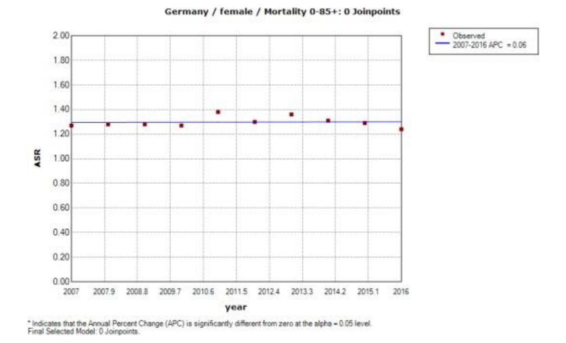

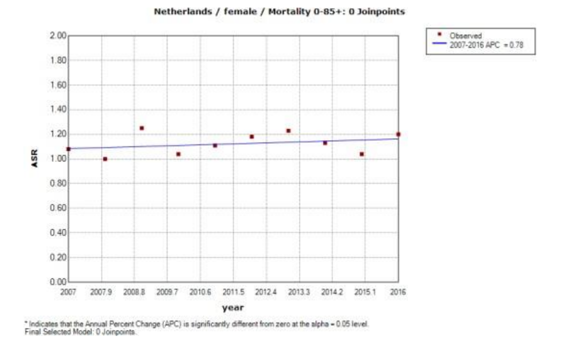

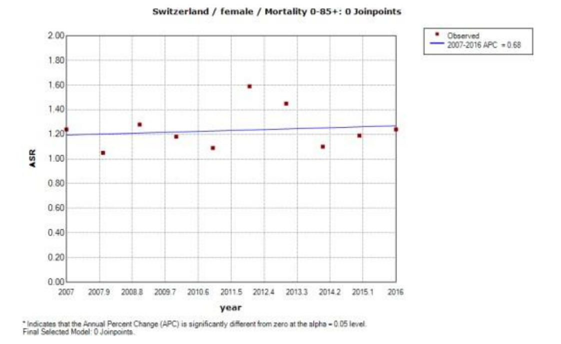

Southern Europe

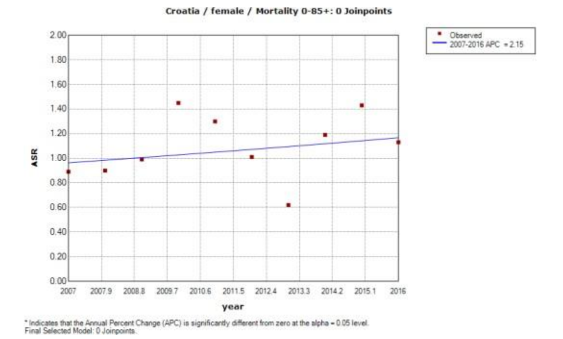

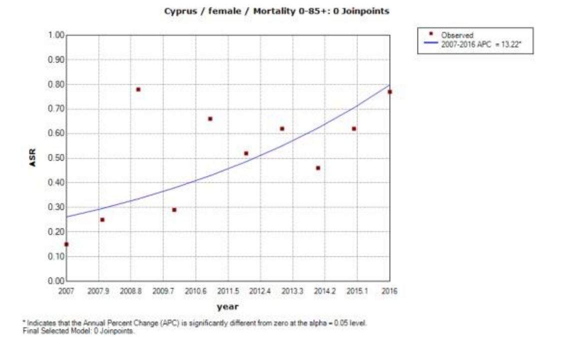

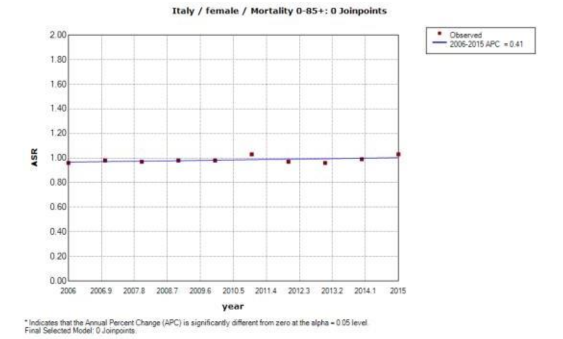

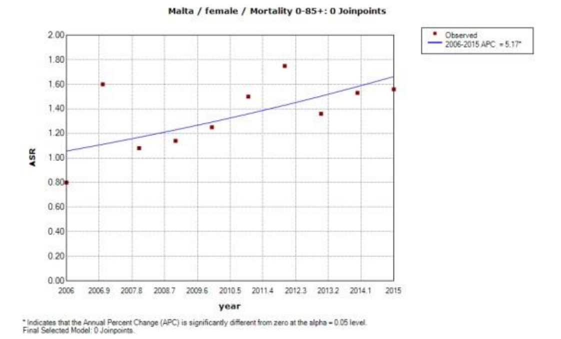

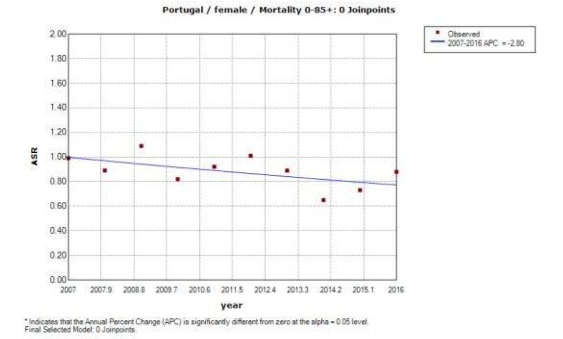

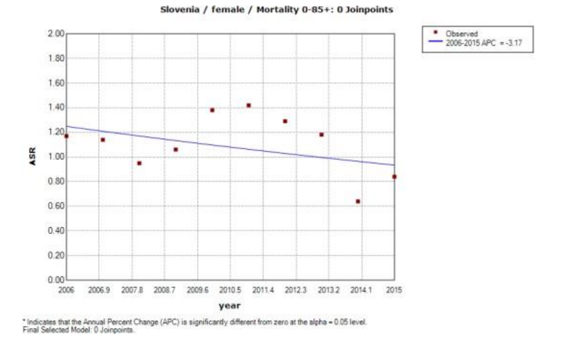

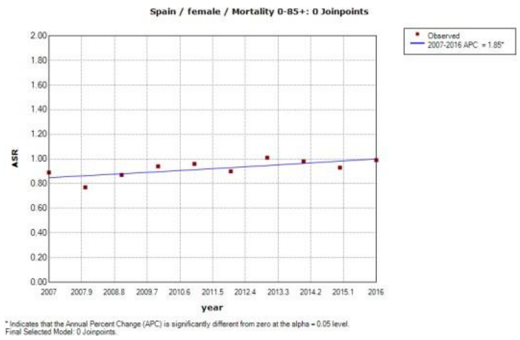

Eastern Europe

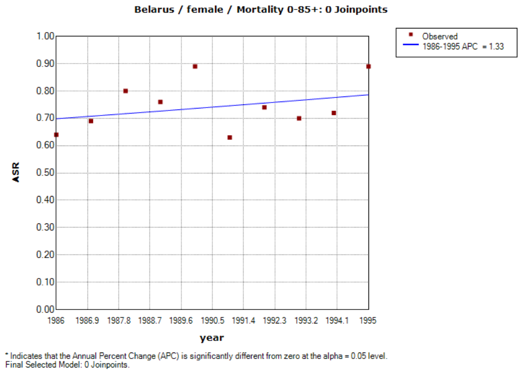

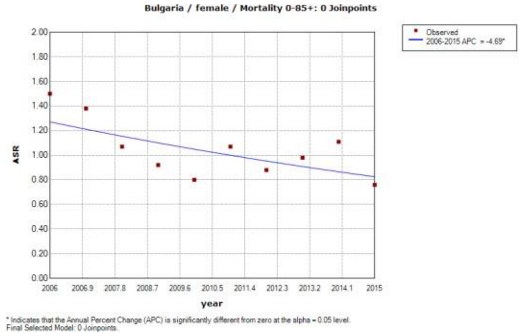

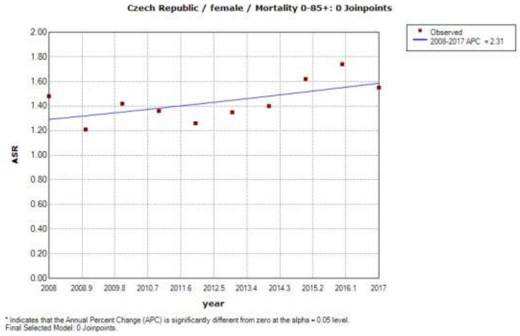

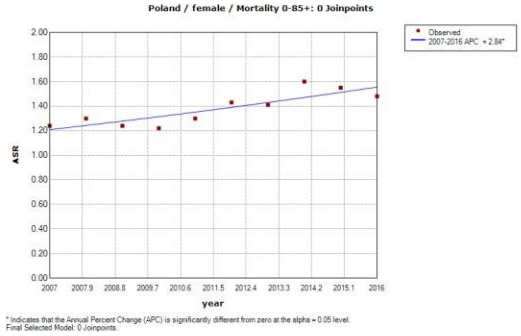

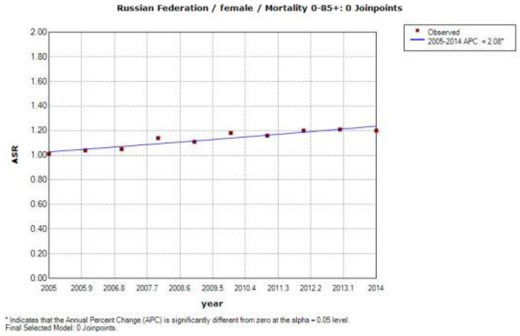

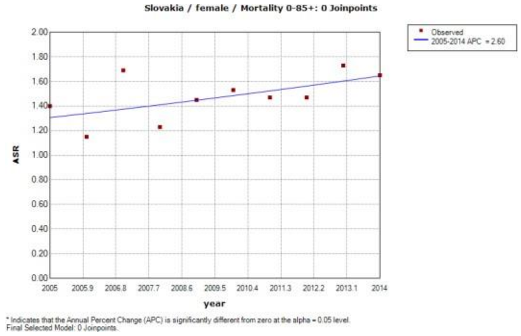

Supplementary Figure 3. AAPC of LOCP cancers incidence for individuals aged 50 years or above

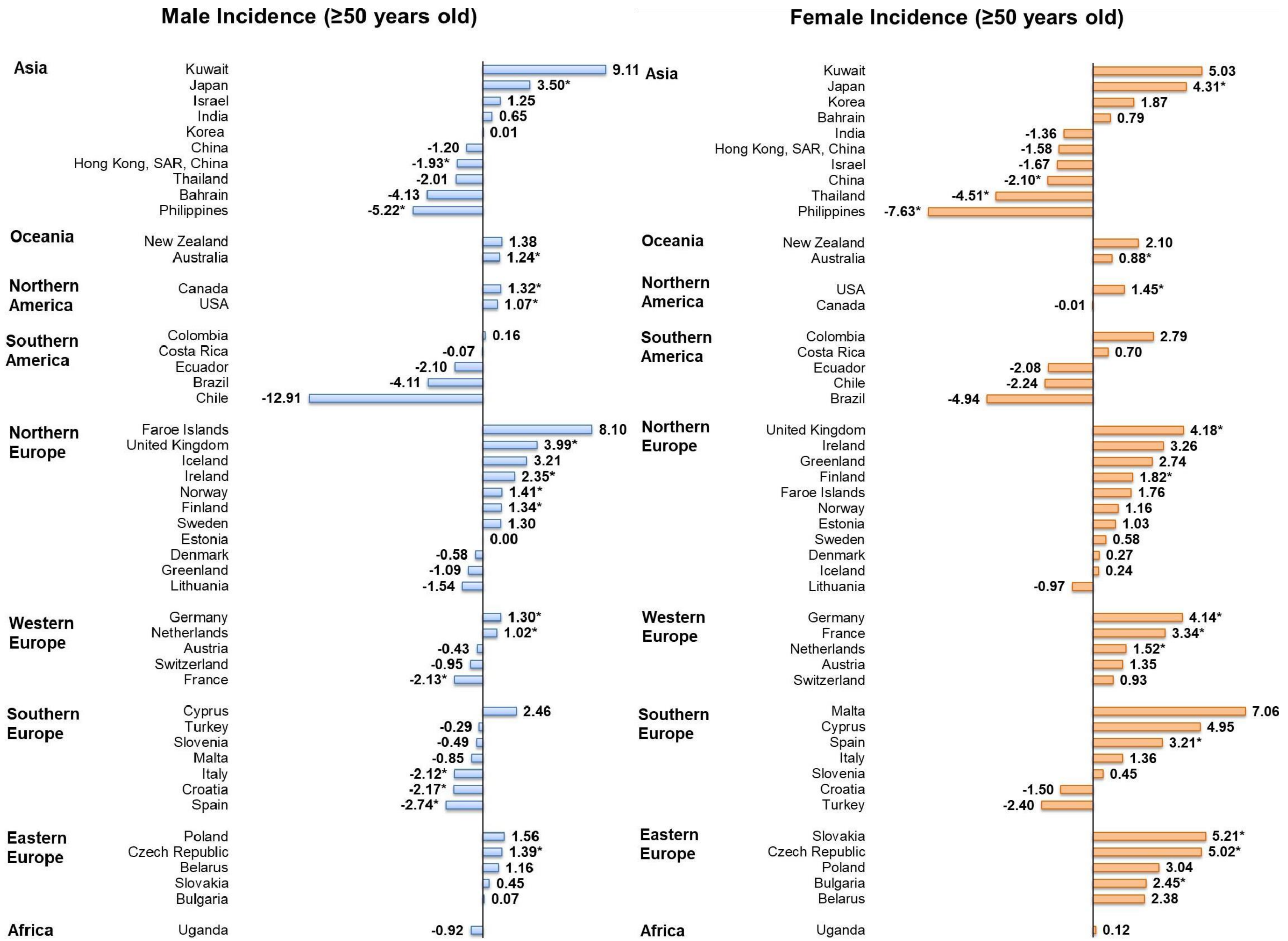

Supplementary Figure 4. AAPC of LOCP cancers incidence for individuals aged < 50 years

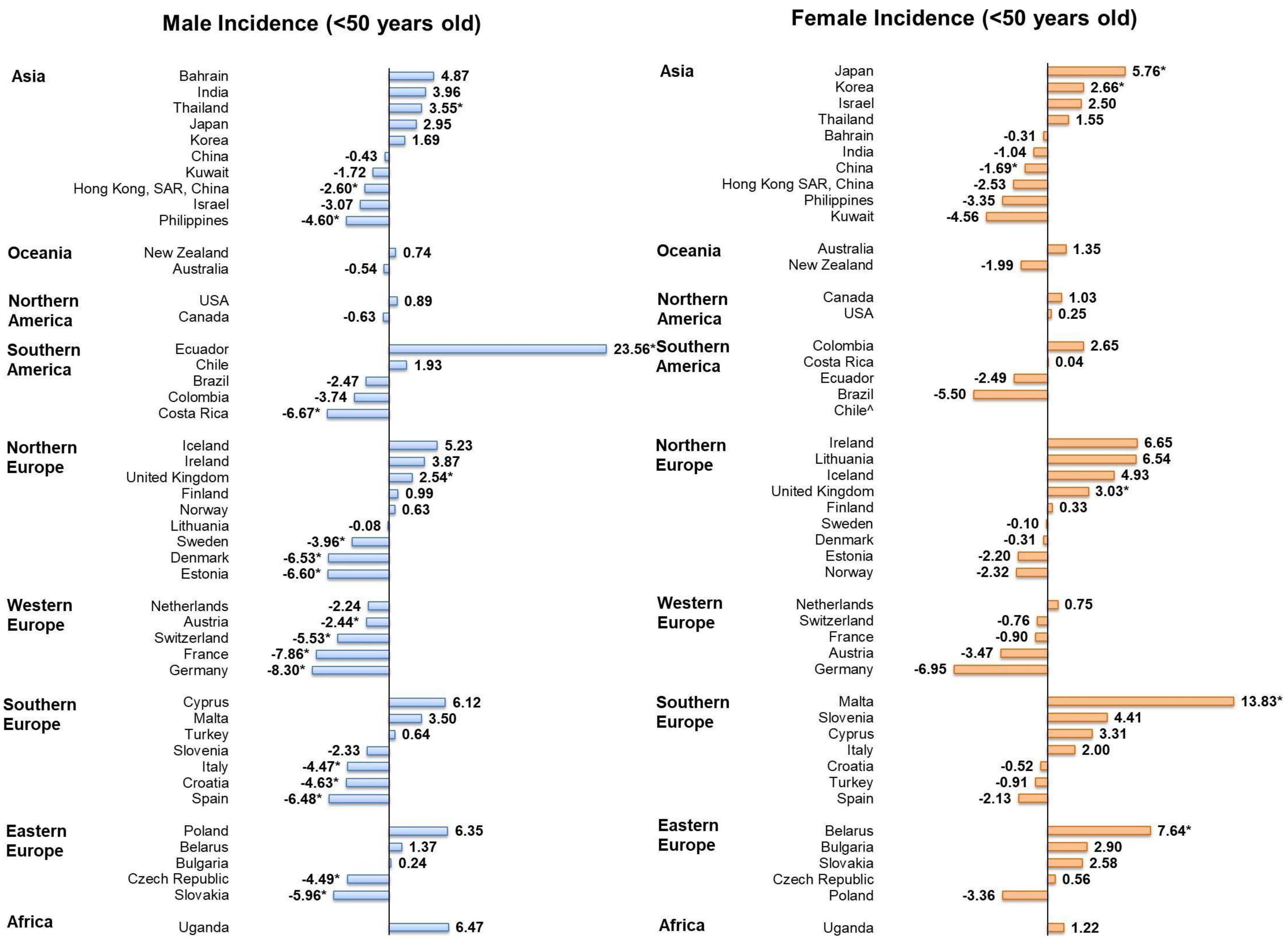

Supplementary Figure 5. AAPC of LOCP cancers incidence for individuals aged < 40 years

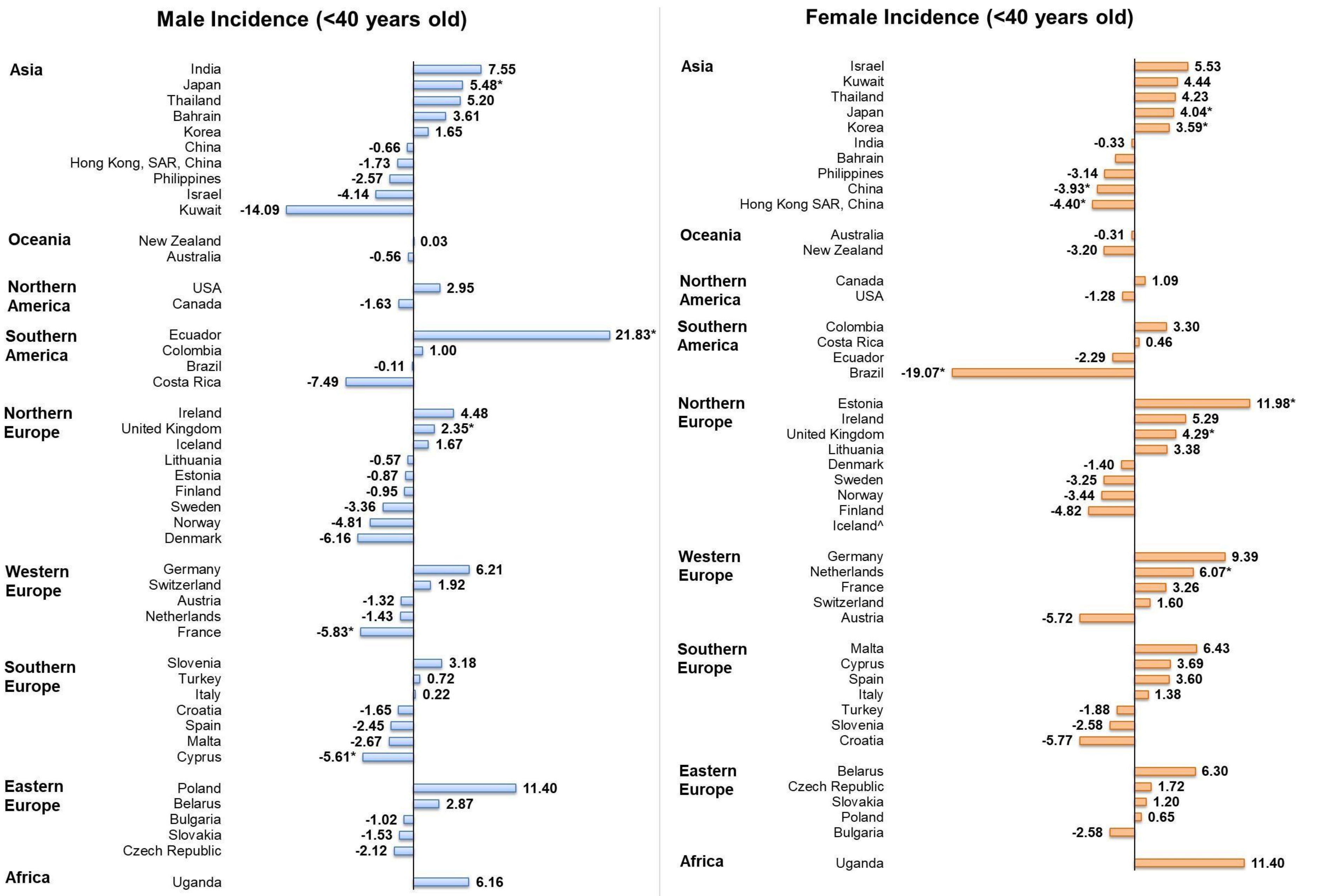

Supplementary Table 1. ASR of LOCP cancers incidence and mortality by age group and subsite

	Age Group	*Lip, oral cavity*	*Salivary glands*	*Oropharynx*	*Nasopharynx*	*Hypopharynx*	*Combined*
Incidence	0-9	0.06	0.03	0.01	0.04	0.00	0.14
	10-19	0.09	0.09	0.01	0.21	0.01	0.42
	20-29	0.74	0.19	0.04	0.41	0.05	1.4
	30-39	2.5	0.35	0.21	1.3	0.24	4.6
	40-49	5.7	0.65	1.3	2.9	1.0	11.6
	50-59	10.6	1.2	3.4	4.4	2.6	22.2
	60-69	16.6	2.2	5.3	4.8	4.5	33.4
	70-85+	20.6	3.4	5.0	3.8	5.0	37.8
	0-85+ (All ages)	4.1	0.6	1.1	1.5	0.9	8.1
Mortality	0-9	0.00	0.00	0.00	0.00	0.00	0.05
	10-19	0.04	0.02	0.00	0.09	0.00	0.15
	20-29	0.39	0.03	0.01	0.13	0.02	0.59
	30-39	1.1	0.06	0.07	0.40	0.13	1.7
	40-49	2.5	0.17	0.52	1.3	0.4	4.9
	50-59	5.0	0.49	1.5	2.6	0.97	10.6
	60-69	8.0	1.1	2.6	3.8	2.0	17.5
	70-85+	10.0	1.9	3.0	3.4	2.8	21.1
	0-85+ (All ages)	1.9	0.23	0.51	0.88	0.41	3.9

Values in age-standardized rates per 100,000 population
